# Design, Synthesis and Antitumor Activity Evaluation of Novel Dual G9a/EZH2 Inhibitors

**DOI:** 10.3390/molecules31193446

**Published:** 2026-09-27

**Authors:** Yajie Shi, Qiuyue Zhang, Maoying Zhang, Xiujuan Zhao, Shuhang Zhang, Jirui Li, Haofeng Li, Chenglin Fu, Guoliang Chen

**Affiliations:** 1 College of Pharmacy, Heze University, Heze 274000, China; xiujuan95@163.com (X.Z.); zhangshuhang@hezeu.edu.cn (S.Z.); 15562712952@163.com (J.L.); 13053971876@163.com (H.L.); 19735685615@163.com (C.F.); 2 Department of Pharmacology, Shenyang Pharmaceutical University, Shenyang 110016, China; zqy45125969@163.com; 3 College of Pharmacy, Jilin Medical University, Changchun 132013, China; 4 Key Laboratory of Structure-Based Drug Design & Discovery of Ministry of Education, School of Pharmaceutical Engineering, Shenyang Pharmaceutical University, Shenyang 110016, China; zmy742634477@163.com

**Keywords:** G9a, EZH2, dual-target inhibitors, antitumor

## Abstract

Targeting the crosstalk between histone methyltransferases G9a and EZH2 represents a promising strategy for cancer therapy. To improve upon our previously reported dual inhibitor SU08, we rationally designed and synthesized five series (SU, UA, UB, N, and L) of novel G9a/EZH2 dual inhibitors, incorporating diverse pyridone, urea, and thienopyrimidine moieties, yielding a total of 43 compounds. In vitro enzymatic screening revealed that compound **SU101** against both EZH2 (IC_50_ = 4.08 nM) and G9a (IC_50_ = 2.92 nM) was comparable to SU08. Western blot analysis confirmed that **SU101** and **SU103** effectively suppressed both H3K27me3 and H3K9me2 expression in rhabdomyosarcoma RD cells. In cellular antiproliferative assays, **SU101** showed the strongest activity (IC_50_ = 15.33 μM). Molecular docking and dynamics simulations verified stable binding interactions between **SU101** and both target proteins. Pharmacokinetic studies in rats demonstrated that **SU101** possesses excellent oral bioavailability (94.3%), moderate plasma protein binding (84.6%), and an acceptable acute toxicity profile (LD_50_ = 31.02 mg/kg in mice). Overall, these results suggest that **SU101** may serve as a promising lead compound for further optimization and support the continued exploration of dual G9a/EZH2 inhibitors as a potential therapeutic approach for rhabdomyosarcoma.

## 1. Introduction

In recent years, growing attention has been paid to the crosstalk between epigenetic targets [1,2]. Numerous studies have demonstrated that G9a [3] (also known as euchromatic histone-lysine N-methyltransferase 2, EHMT2) and EZH2 [4] (enhancer of zeste homologue 2) synergistically contribute to cancer initiation and progression [5]. Both enzymes are concomitantly upregulated in various malignancies, including breast cancer [6,7], ovarian cancer [8], melanoma [9], multiple myeloma [10,11], and testicular germ cell tumors [12]. Chiara et al. found that G9a and EZH2 (via the PRC2 complex) interact genome-wide and co-localise, sharing epigenetically silenced targets and co-regulating common genes that encode developmental regulators [13]. Later, in 2022, Fong and colleagues described the interaction mechanism between G9a and the PRC2 complex during prostate cancer progression [14]. The recently identified PRC2-associated ligand-dependent co-repressor isoform 1 (PALI1) is a PRC2 subunit that associates with the PRC2.1 complex through interaction with the core subunit SUZ12, while also being able to interact with the H3K9 methyltransferase G9a. Thus, PALI1 bridges PRC2 and G9a, generating a novel G9a–PALI1–PRC2 complex that is recruited to a subset of G9a target genes involved in the regulation of developmental processes, most notably cell differentiation. At these developmental genes, the G9a–PALI1–PRC2 complex catalyzes dual methylation at H3K9 and H3K27, leading to epigenetic silencing.

**Figure 1:**
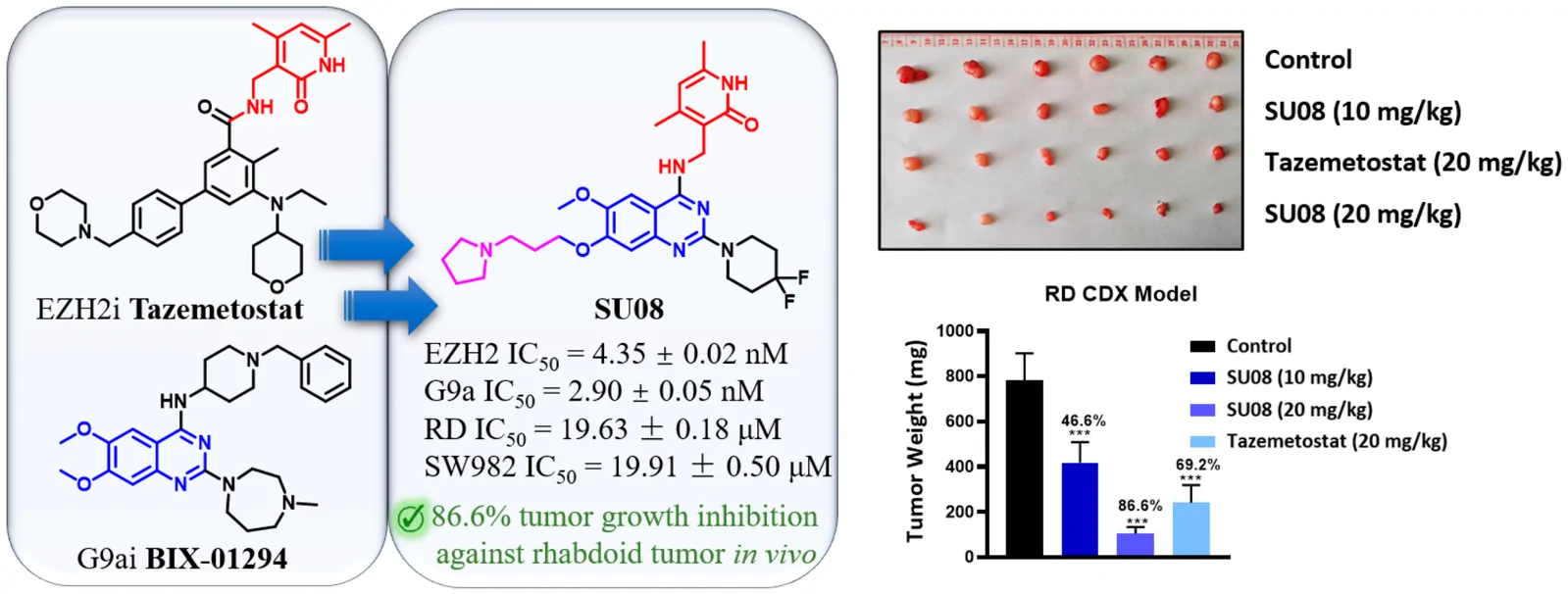
Design and biological activity evaluation of SU08. Tazemetostat (20 mg·kg^−1^) or SU08 (10, 20 mg·kg^−1^) was administered for 5 consecutive days followed by 2 rest days for 3 weeks (6 mice per group) by intraperitoneal administration in antitumor activity study. *** *p* < 0.001.

Structurally, EZH2 and G9a share homology, both containing a catalytically active SET domain, a lysine substrate-binding region, and a cofactor-binding region [15]. They can interact with each other and mutually influence histone methylation levels, synergistically promoting the development and progression of multiple cancers [16]. Therefore, designing dual inhibitors targeting both EZH2 and G9a may represent a novel direction in antitumor drug discovery. Indeed, BIX-01294 [17] has been shown to inhibit both G9a and EZH2. In 2015, Curry et al. reported HKMTi-1-005 [7], a dual inhibitor based on BIX-01294, which also exhibited activity against both targets, thereby confirming the feasibility of developing dual-target compounds. More recently, we reported a series of G9a/EZH2 dual inhibitors designed *via* a hybridisation strategy using Tazemetostat and UNC0638 as leads. Among them, compound SU08 (15 h) [18,19] maintained potent inhibitory activity against both EZH2 (IC_50_ = 4.35 ± 0.02 nM) and G9a (IC_50_ = 2.90 ± 0.05 nM) in vitro, comparable to the parent compounds Tazemetostat and UNC0638 (Figure 1). The in vivo antitumor efficacy of SU08 was evaluated in a rhabdomyosarcoma RD cell xenograft mouse model. After three weeks of treatment, SU08 (20 mg/kg, TGI: 86.6%) showed superior tumor growth inhibition compared to Tazemetostat (20 mg/kg, TGI: 69.2%). These results indicated that the G9a/EZH2 dual inhibitor SU08 outperformed the single-target EZH2 inhibitor Tazemetostat in the mouse xenograft model, and collectively suggested that SU08 is a potential drug candidate for rhabdomyosarcoma therapy, laying a foundation for the dual targeting of G9a and EZH2 as a treatment strategy for this malignancy.

On the basis of these findings, the present study employed SU08 as a lead compound for further structural optimization, leading to the design of five series of novel G9a/EZH2 dual inhibitors.

**Figure 2:**
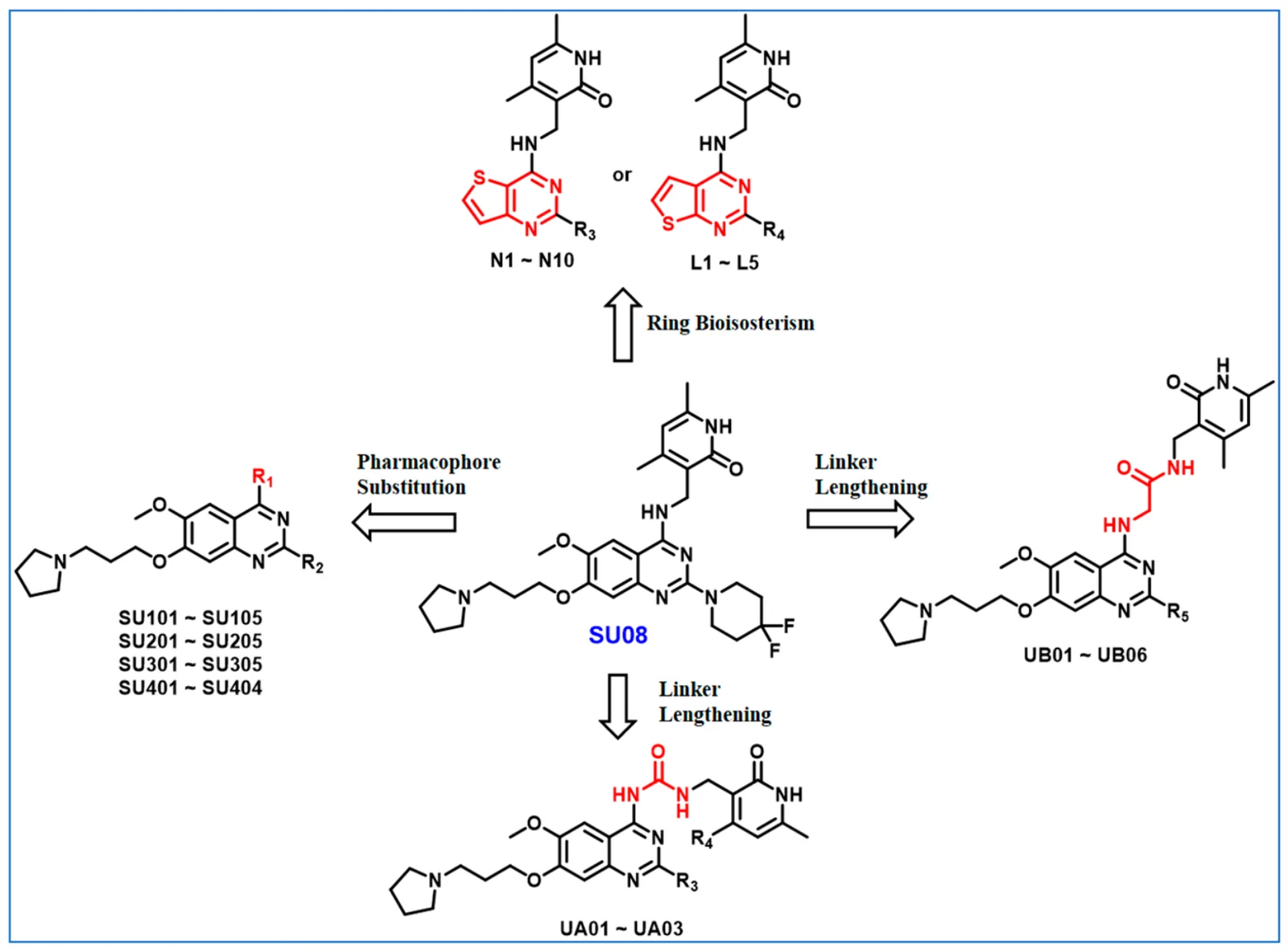
Design of SU, UA, UB, N and L series.

Given that SU08 bears considerable structural similarity to G9a inhibitors, and that both enzymatic assays and Western blot analyses indicated relatively weak EZH2 activity, we first examined the structural features of EZH2 inhibitors with the aim of enhancing EZH2 potency without compromising G9a inhibition. The pyridone moiety, which serves as a key functional group in many EZH2 inhibitors, confers strong protein-binding affinity through the formation of a hydrogen bond with Trp521, thereby anchoring the inhibitor within the SAM-binding site. The structure–activity relationships at the C-4 and C-6 positions of the pyridone ring have been extensively explored in previous reports. In the present work, we selected four favorable pyridone scaffolds as key pharmacophores and designed compounds **SU101–SU404** (Figure 2).

Further analysis of EZH2 inhibitor structures revealed that the pyridone ring is typically linked to an aromatic ring via an amide bond, and the amide carbonyl can serve as a hydrogen-bond acceptor to engage Tyr111, thereby enhancing activity. In contrast, G9a inhibitors require a secondary amine at the 4-position of the quinazoline core to act as a hydrogen-bond donor. Thus, to reconcile these opposing requirements, we hypothesized that a urea or carboxamide linker might simultaneously fulfil the hydrogen-bond donor and acceptor demands of both G9a and EZH2 inhibitors. On the basis of this rationale, the **UA** and **UB** series of compounds were designed (Figure 2).

Ring bioisosteric replacement [20] is a widely employed and highly successful strategy in drug discovery. Accordingly, we considered the thienopyrimidine ring as a bioisostere of the quinazoline scaffold and replaced the quinazoline core of the SU series with thienopyrimidine, leading to the design of the N and L series of novel G9a/EZH2 dual inhibitors (Figure 2).

## 2. Results

### 2.1. Chemistry

**Scheme 1:**
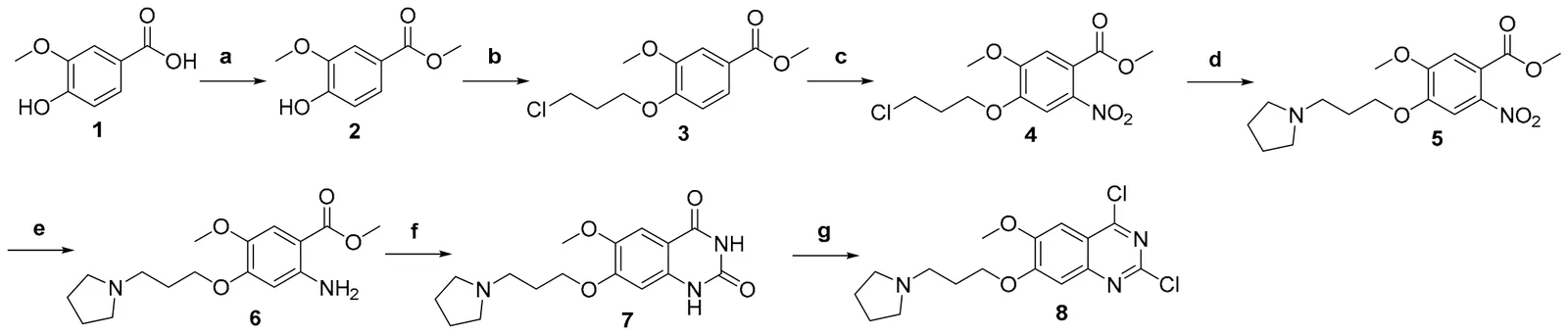
Synthetic route of **8**. Reagents and conditions: (**a**) SOCl_2_, MeOH, reflux, 7 h; (**b**) 1-bromo-3-chloropropane, K_2_CO_3_, CH_3_CN, 70 °C, 30 h; (**c**) fuming nitric acid, AcOH, Ac_2_O, 0–25 °C, 5 h; (**d**) pyrrolidine, KI, K_2_CO_3_, CH_3_CN, reflux, 10 h; (**e**) Sn, HCl (**g**), EtOH, 50 °C, 5 h; (**f**) KOCN, AcOH, H_2_O, 65 °C, 10 h; (**g**) POCl_3_, DIEA, acetone, 80 °C, 5 h.

The synthesis of key intermediate **8** was accomplished in seven steps, as outlined in Scheme 1. Commercially available vanillic acid was used as the starting material and converted to methyl ester **2** *via* esterification. Subsequent substitution with 1-bromo-3-chloropropane afforded compound **3**, which was then subjected to nitration with concentrated nitric acid, followed by ammonolysis with pyrrolidine and reduction with tin powder to furnish intermediate **6**. Thereafter, cyclization of **6** with potassium cyanate under AcOH/H_2_O conditions [21], followed by chlorination with phosphorus oxychloride, provided the quinazoline key intermediate **8** [22,23].

**Scheme 2:**
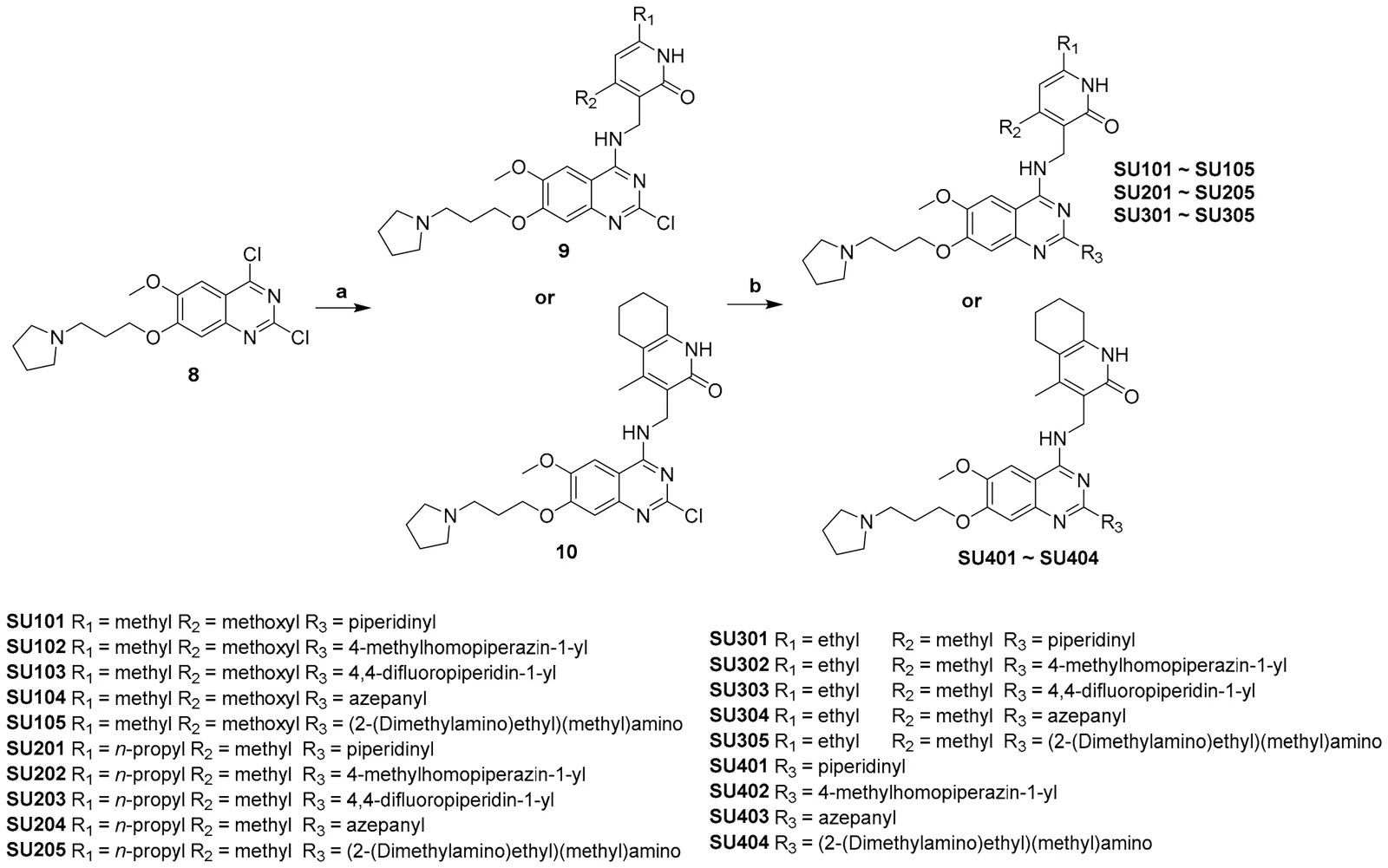
Synthetic route of **SU** compounds. Reagents and conditions: (**a**) corresponding amines, K_2_CO_3_, DMF, 70 °C, 10 h; (**b**) corresponding amines, TsOH, *n*-BuOH, Ar, 110 °C, 5–15 h.

The synthesis of the SU series of compounds was carried out as shown in Scheme 2. Starting from the quinazoline key intermediate **8**, the corresponding intermediates were obtained *via* ammonolysis, which were then refluxed with various amines under an argon atmosphere in the presence of TsOH to afford compounds **SU101–SU404**.

**Scheme 3:**
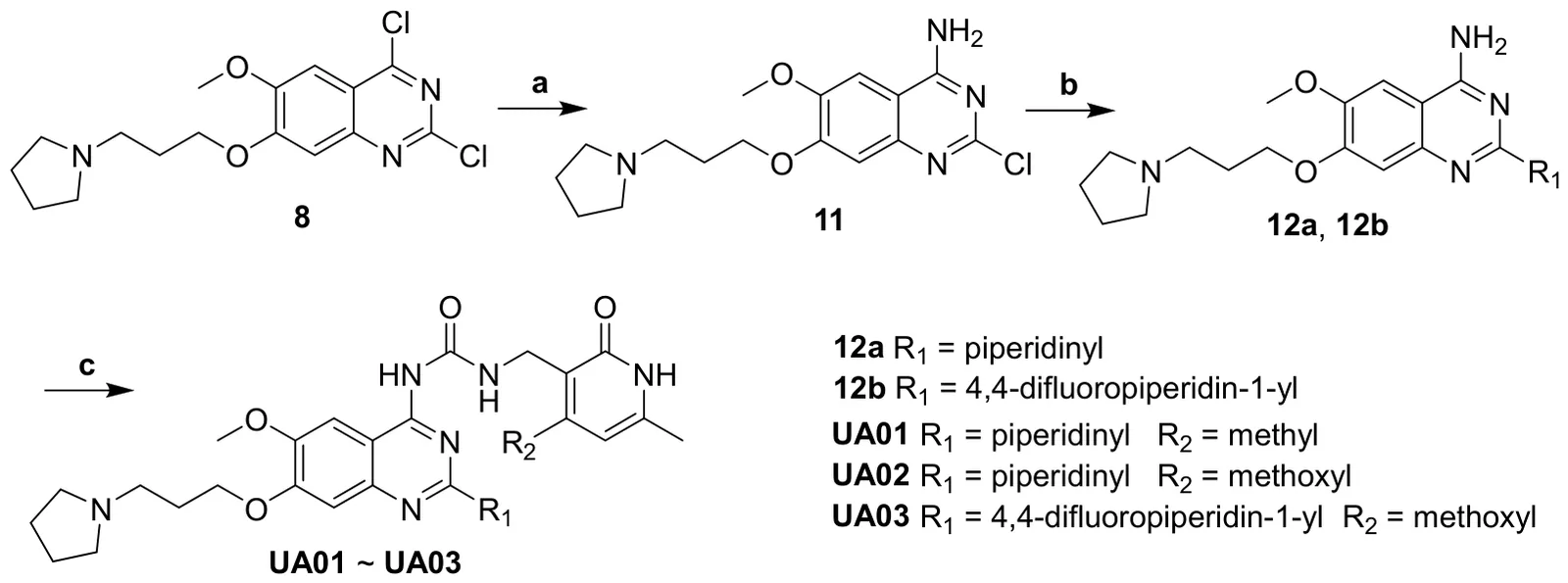
Synthetic route of **UA** compounds. Reagents and conditions: (**a**) 25% NH_3_·H_2_O, THF, r.t., 5 h; (**b**) corresponding amines, TsOH, *n*-BuOH, Ar, 110 °C, 5–15 h; (**c**) (1) BTC, TEA, Py, DCM, −80–60 °C, 2 h; (2) corresponding amines, 60 °C.

The synthesis of the UA series of compounds is illustrated in Scheme 3. Using the quinazoline key intermediate **8** as the starting material, ammonolysis with aqueous ammonia in tetrahydrofuran first afforded intermediate **11**, which was then refluxed with various amines under an argon atmosphere in the presence of TsOH to give the corresponding intermediates **12a** and **12b**. Subsequently, these intermediates were added dropwise to a solution of triphosgene in TEA/Py/DCM at −80 °C to generate the isocyanate derivatives, which were then refluxed with different amines in DCM to furnish the target compounds **UA01–UA03**.

**Scheme 4:**
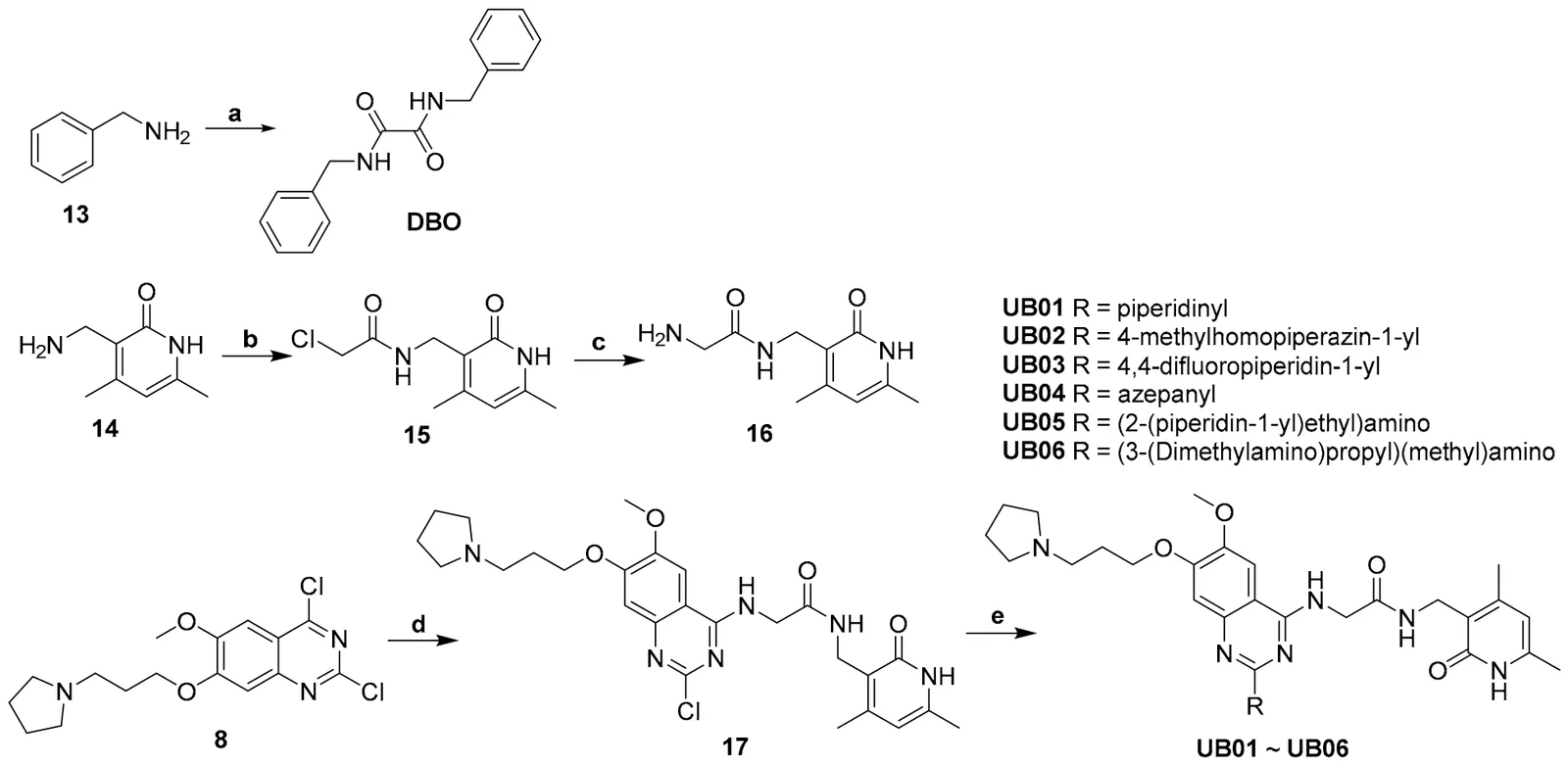
Synthetic route of **UB** compounds. Reagents and conditions: (**a**) oxalyl chloride, TEA, THF, 0 °C—r.t., 2 h; (**b**) chloroacetyl chloride, TEA, DCM, 0 °C—r.t., 2 h; (**c**) 25% NH_3_·H_2_O, EtOH, 70 °C, 7 h; (**d**) **16**, CuI, DBO, K_3_PO_4_·H_2_O, DMSO, Ar, 120 °C, 10 h; (**e**) corresponding amines, TsOH, *n*-BuOH, Ar, 110 °C, 10 h.

The synthesis of the **UB** series of compounds is illustrated in Scheme 4. First, the oxalamide ligand DBO and key intermediate **16** were synthesized for the main route. DBO was obtained by acylation of benzylamine (**13**) with oxalyl chloride in the presence of triethylamine (TEA) [24]. Intermediate **16** was prepared from **14** through two steps: acylation with chloroacetyl chloride followed by ammonolysis with 25% aqueous ammonia. In the main route, starting from quinazoline key intermediate **8**, intermediate **17** was obtained *via* an Ullmann reaction using DBO as the ligand [25,26], which was then reacted with various secondary amines under reflux in the presence of TsOH to afford **UB01–UB06**.

**Scheme 5:**
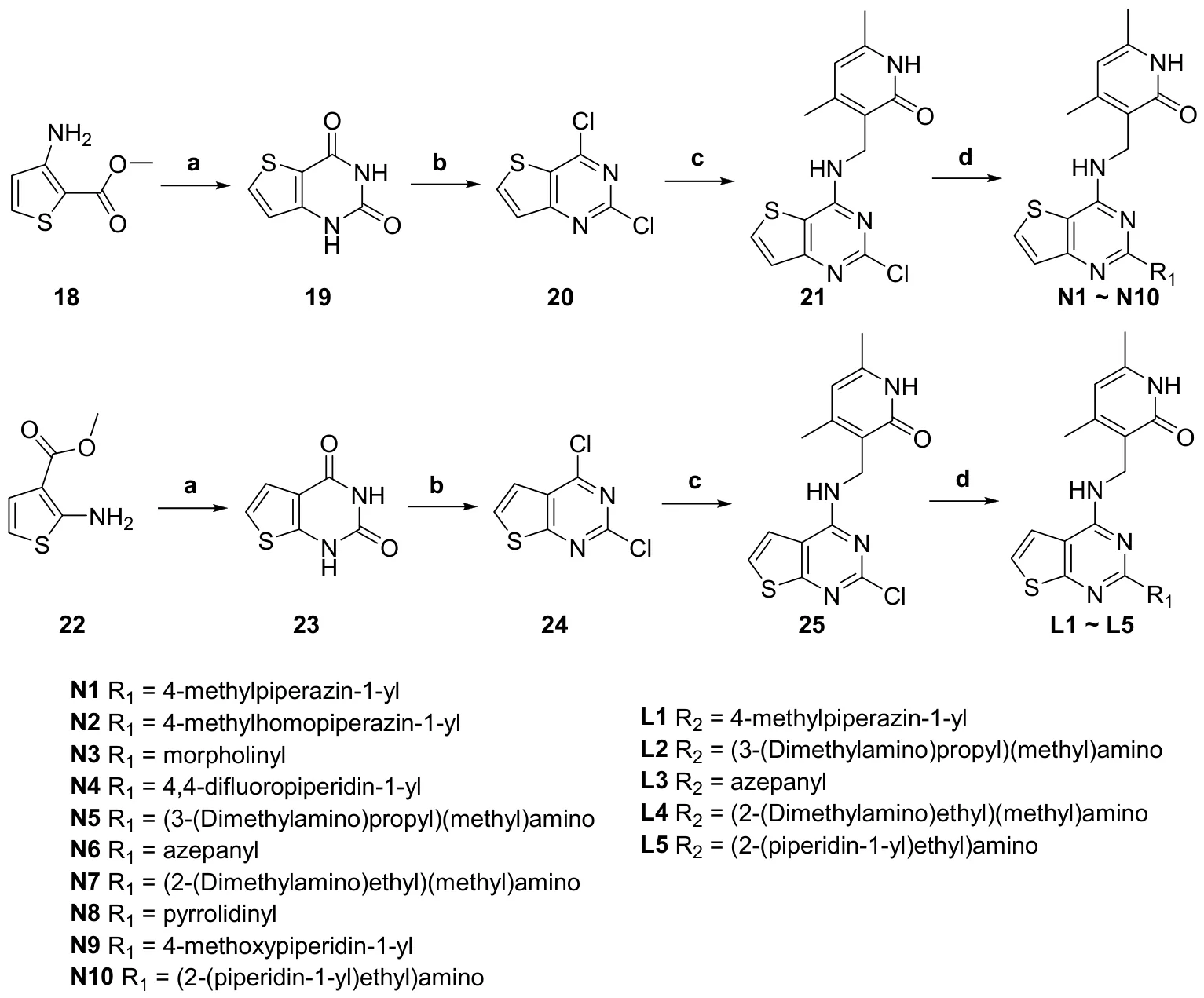
Synthetic route of **N** and **L** compounds. Reagents and conditions: (**a**) (1) urea, 200 °C, 1.5 h; (2) 20% NaOH, 100 °C, 5 h; (**b**) POCl_3_, PhNMe_2_, reflux, 4 h; (**c**) **14**, Na_2_CO_3_, DMF, Ar, 70 °C, 7 h; (**d**) corresponding amines, TsOH, *n*-BuOH, Ar, 110 °C, 24–36 h.

The synthesis of the N and L series of compounds is illustrated in Scheme 5, comprising four steps in total. Methyl 3-amino-2-thiophenecarboxylate (**18**) or methyl 2-aminothiophene-3-carboxylate (**22**) was used as the starting material and cyclized with urea under high-temperature conditions to afford intermediate **19** or **23** [27,28], which was then subjected to dichlorination with phosphorus oxychloride under reflux to give intermediate **20** or **24**. Subsequent aminolysis with pyridone intermediate **14** in the presence of potassium carbonate under basic conditions furnished **21** or **25**. Finally, the target compounds **N1–N10** and **L1–L5** were obtained by aminolysis of **21** or **25** with the corresponding secondary amines in the presence of TsOH.

### 2.2. In Vitro HMT Assay

To facilitate the preliminary screening of the large number of synthesized compounds, the inhibitory rates of EZH2i GSK126, Tazemetostat, G9ai UNC0638, the **SU**, **UA**, **UB**, **N**, and **L** series against G9a and EZH2 were first evaluated at a single concentration of 5 μM, and the results are summarized in Table 1. Overall, the **N** and **L** series exhibited relatively low inhibitory rates against both G9a and EZH2, whereas the **UA** and **UB** series showed moderate activity. Notably, the **UA** series displayed stronger EZH2 inhibition than G9a inhibition, while the **SU** series gave the best overall performance.

For the G9a target, the inhibitory activity followed the order: **SU** > **UA** > **UB** > **N** > **L**. The thienopyrimidine derivatives **N1–N10** and **L1–L5** showed weak G9a inhibition, with the **N1–N10** series being more potent than **L1–L5**, indicating that the quinazoline core is superior to thieno [3,2-*d*]pyrimidine, which in turn is superior to thieno [2,3-*d*]pyrimidine. Compounds **UA01–UA03**, likely due to the introduction of the urea moiety that increased rigidity and restricted conformational flexibility, showed reduced G9a activity. The **UB** series (**UB01–UB06**) displayed G9a inhibition similar to that of the **UA** series, suggesting that elongation of the substituent at the C-4 position of the quinazoline core did not improve activity.

For the EZH2 target, the **SU** and **UA** series showed comparable inhibitory activity, which was stronger than that of the **UB** series, followed by the **N** and **L** series. No significant difference in EZH2 inhibition was observed between the **N** and **L** series, indicating that the choice of thieno[3,2-*d*]pyrimidine versus thieno[2,3-*d*]pyrimidine had little impact on EZH2 activity. Compounds **UA01–UA03** exhibited potent EZH2 inhibition, with an average inhibitory rate higher than that of the SU series, indicating that the incorporated urea moiety enhanced EZH2 activity. **UB** series (**UB01–UB06**) showed weaker EZH2 inhibition compared to the **SU** and **UA** series, suggesting that extending the substituent at the quinazoline C-4 position led to decreased EZH2 activity.

Taken together, the structure–activity relationship analysis for both targets revealed that the **SU** series were the most promising, which retained the dual G9a/EZH2 inhibitory activity of the parent compound **SU08**. The single-concentration inhibition rate of 5 μM is only used for preliminary ranking and rapid screening of compounds. Accurate structure–activity relationship analysis is based on subsequent complete IC_50_ determination results; most compounds exhibit high inhibition rates at a single concentration, with limited differentiation of activity differences.

Based on the above screening, five promising compounds, together with the positive controls (Tazemetostat, UNC-0638, and SU08), were selected for further determination. IC_50_ values were calculated. As shown in Table 2, compound **SU101** exhibited potent inhibitory activity, comparable to that of the lead compound SU08. **UA01** displayed stronger EZH2 inhibition than G9a inhibition, whereas **UB01** showed relatively weak inhibitory activity against both EZH2 and G9a.

### 2.3. Western Blot Analysis

**Figure 3:**
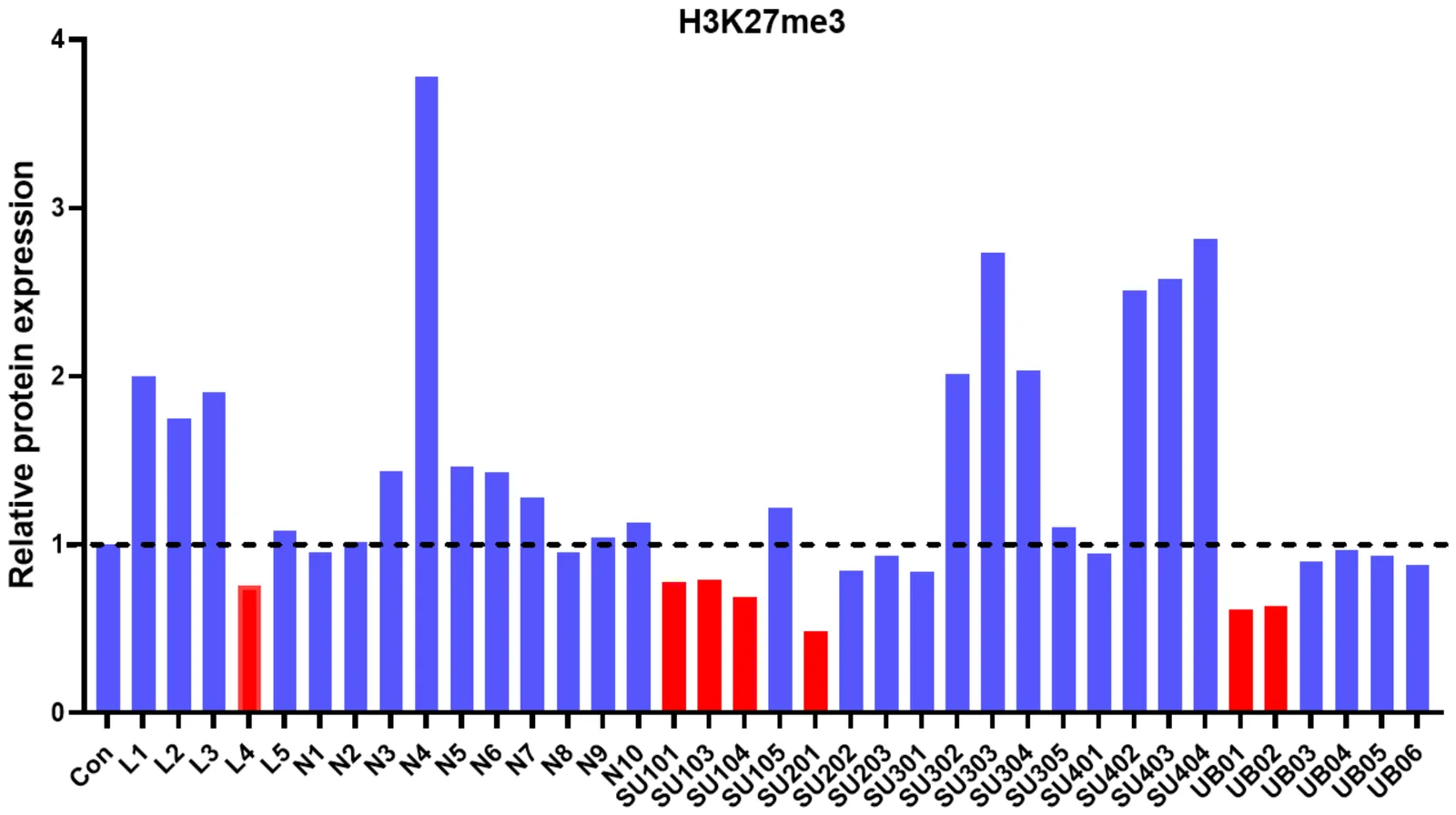
Effects of compounds (5 μM) on H3K27me3 protein expression in RD cells.

To explore the inhibitory effects of the compounds at the cellular level, we first examined the impact of most compounds on the protein expression of H3K27me3 (the EZH2-catalyzed substrate) in rhabdomyosarcoma RD cells at a single concentration. As shown in Figure 3, most of the compounds had little effect on H3K27me3 expression at the tested concentration; however, at 5 μM, H3K27me3 expression was upregulated in the presence of several compounds. This observation was not entirely consistent with the results of the enzymatic assays, which may be attributable to the fact that a concentration of 5 μM in the cellular context was insufficient for some compounds to exert their inhibitory effects, and also to differences in assay methodologies and experimental variability.

Among the tested compounds, **SU101**, **SU103**, **SU104**, **SU201**, **UB01**, **UB02**, and **L4** exhibited relatively pronounced inhibition of H3K27me3 expression in RD cells. These seven small-molecule compounds were therefore selected for further validation.

**Figure 4:**
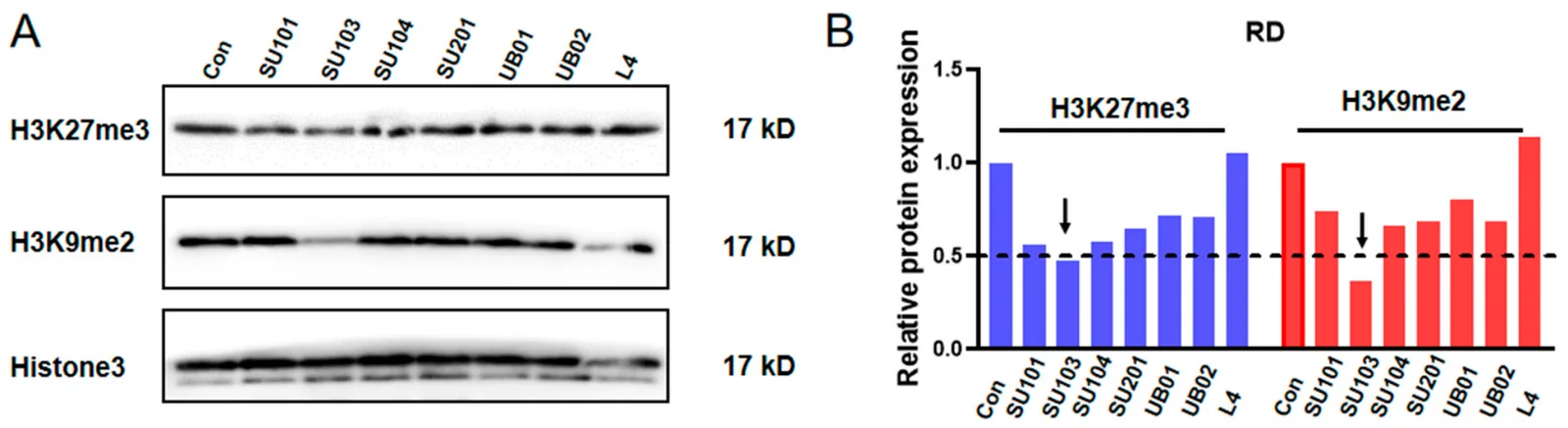
The blots (**A**) and bar chart (**B**) of effects of compounds (5 μM) on H3K27me3 and H3K9me2 protein expression in RD cells.

As shown in Figure 4, the inhibitory effects of the above seven compounds on the EZH2-catalyzed substrate H3K27me3 and the G9a-catalyzed substrate H3K9me2 in RD cells were further validated at a concentration of 5 μM. The results demonstrated that **SU101** and **SU103** exhibited relatively pronounced inhibitory effects on both H3K27me3 and H3K9me2 protein expression. The results showed a modest discrepancy compared with the HMT assay, possibly due to the effects of other methyltransferases present in cells or variations in the experimental procedures.

### 2.4. In Vitro Antiproliferative Activity Evaluation

Based on the combined results of the enzymatic activity assays and Western blot analyses, specifically including the urea-containing UA series so that the influence of the urea linker on cellular activity could be evaluated, the preferred compounds **SU101**, **SU103**, **SU104**, **UA01**, **UA02**, **UA03**, and **UB02** were selected for antiproliferative assays against RD cells to further identify active candidates, using Tazemetostat, UNC0638, and SU08 as controls. The results are presented in Table 3. Compared to the results of the HMT assay, the IC_50_ values in RD cells exhibit a significant difference in magnitude, which may be attributed to fundamental differences between the two assay systems: the biochemical assay measures direct inhibition of isolated enzymes in an optimized in vitro system, whereas the antiproliferative activity evaluation integrates membrane permeability, intracellular drug accumulation, and protein binding, assay duration (72 h), as well as possible feedback/compensatory pathways that can partially offset enzymatic inhibition in intact cells. Such discrepancies are commonly observed for epigenetic inhibitors and are not inconsistent with target engagement [29,30].

**UB02** did not exhibit appreciable cytotoxicity (IC_50_ > 100 μM), whereas the remaining compounds showed favorable activity. As shown in the tables, when the C-2 position of the quinazoline bore a piperidine group, introduction of a urea moiety at C-4 led to decreased cytotoxicity (**SU101** vs. **UA01**, **UA02**); however, when the C-2 substituent was 4,4-difluoropiperidine, the urea moiety at C-4 had little effect on cytotoxicity (**SU103** vs. **UA03**).

Compound **SU101** (IC_50_ = 15.33 ± 0.31 μM) displayed stronger antiproliferative activity against RD cells than SU08, warranting further investigation.

### 2.5. Molecular Modeling

**Figure 5:**
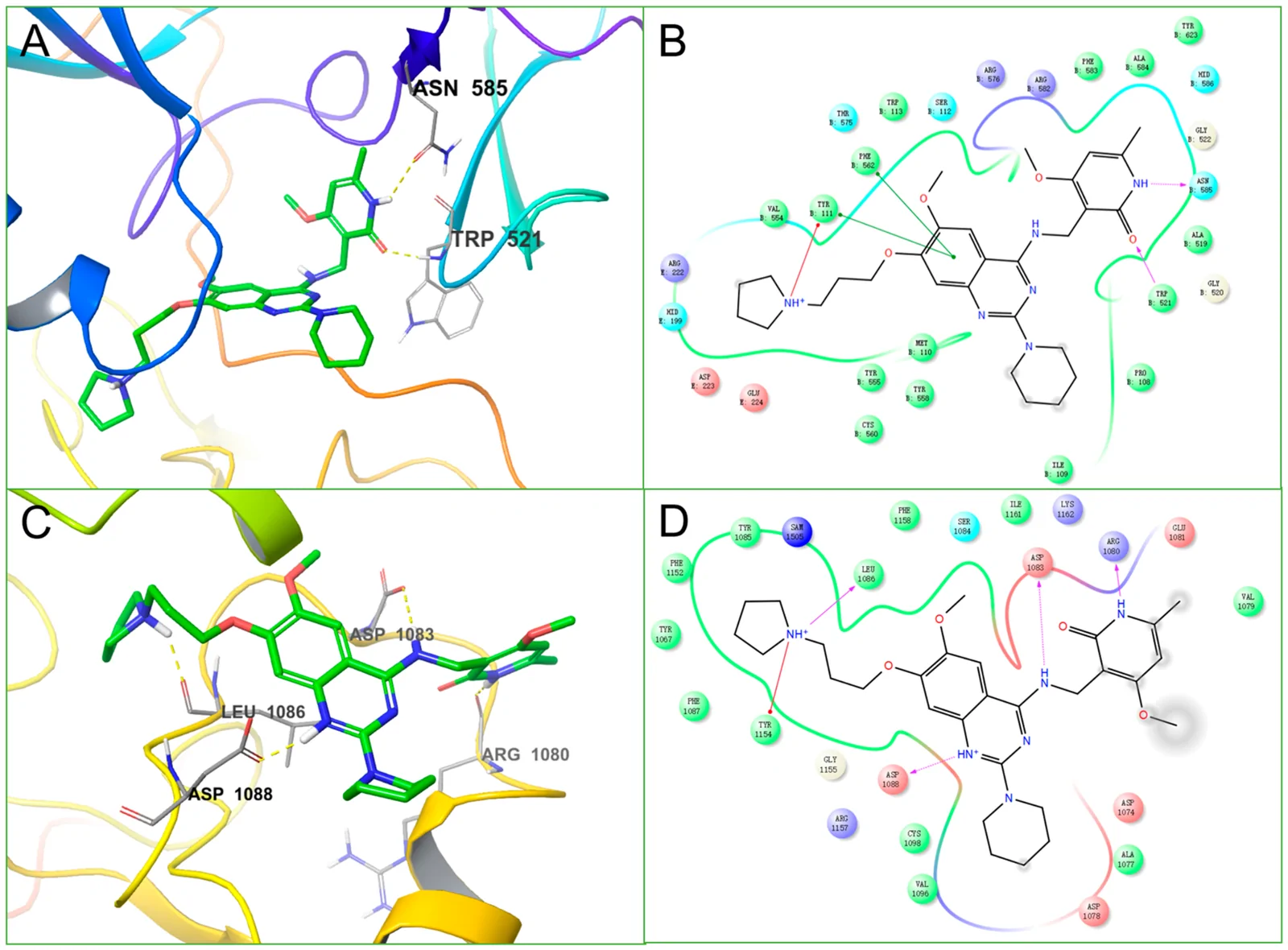
(**A**,**B**) Predicted complex of **SU101** (Green) to EZH2 (PDB ID: 4W2R). (**C**,**D**) Predicted complex of **SU101** (Green) to G9a (PDB ID: 5VSC).

To gain insight into the interactions between the most bioactive compound **SU101** and G9a/EZH2, molecular docking simulations were performed using Schrödinger 2016-2 software with G9a (PDB ID: 5VSC) [31] and EZH2 (PDB ID: 4W2R) [32]. In the predicted binding complex of **SU101** and EZH2, the pyridinone group leads to the same manner as Tazemetostat in the predicted binding complex of **SU101** and EZH2 (Figure 5A,B). Meanwhile, **SU101** forms hydrogen bonds with Arg1080, Asp1083, Leu1086, and Asp1088 in G9a (Figure 5C,D). **SU101** can be accommodated well by the cavity of G9a and EZH2, which contributes to its favorable protein binding affinity.

### 2.6. Molecular Dynamics Simulation

**Figure 6:**
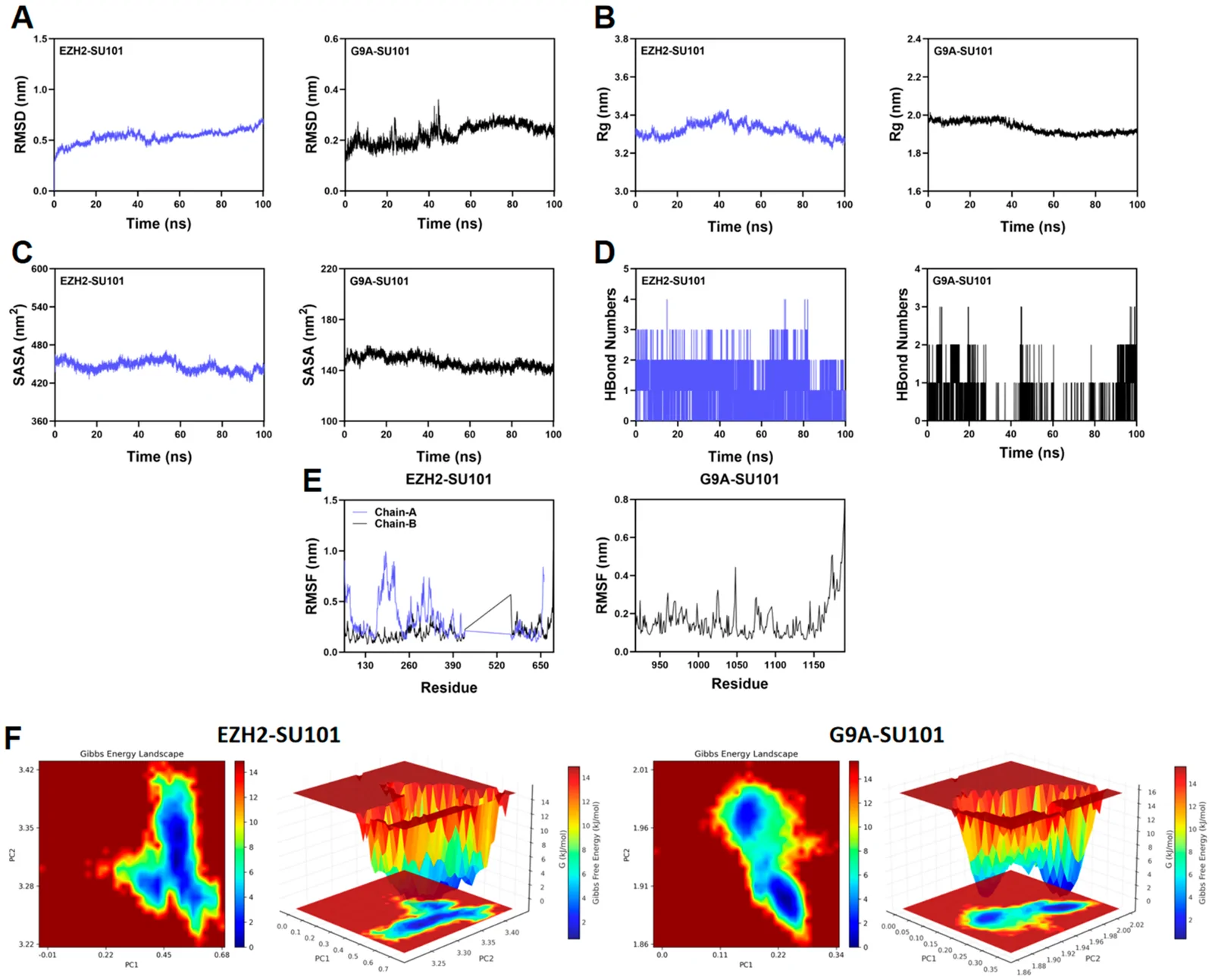
Molecular dynamics simulations of the protein–SU101 complexes (G9a PDB ID: 5VSC, EZH2 PDB ID: 4W2R). (**A**) RMSD values of the protein–ligand complexes over time; (**B**) Rg values over time; (**C**) SASA values over time; (**D**) H-bond numbers over time; (**E**) RMSF values of the protein–ligand complexes; (**F**) Free energy landscape.

The root-mean-square deviation (RMSD) is a reliable indicator of both protein–ligand conformational stability and the degree of atomic deviation from the initial positions; smaller deviations reflect greater stability. Therefore, RMSD was employed to assess the equilibration of the simulation systems. As shown in Figure 6A, the EZH2–**SU101** complex reached equilibrium after 50 ns and subsequently fluctuated around 0.1 nm. The G9a–**SU101** complex equilibrated after 60 ns and then fluctuated around 0.27 nm. These results indicate that **SU101** binds to both EZH2 and G9a with high stability.

The radius of gyration (Rg) describes global structural changes and can reflect the compactness of the protein structure. Both the EZH2–**SU101** and G9a–**SU101** complexes exhibited slight fluctuations during the simulation (Figure 6B), suggesting that conformational rearrangements occurred upon ligand binding.

The solvent-accessible surface area (SASA) is a measure of the protein surface area. The SASA values for the protein–ligand complexes were calculated throughout the simulation (Figure 6C). Both systems showed minor fluctuations, indicating that ligand binding influences the binding microenvironment and leads to moderate changes in SASA.

Hydrogen bonds play a crucial role in ligand–protein interactions. The number of hydrogen bonds formed between the small molecule and the target protein over the simulation trajectory is displayed in Figure 6D. For the EZH2–**SU101** complex, the hydrogen-bond count ranged from 0 to 4, with an average of approximately 2 bonds in most frames. For the G9a–**SU101** complex, the count ranged from 0 to 3, averaging about 1 bond. These data indicate favorable hydrogen-bonding interactions between the ligand and both target proteins.

The root-mean-square fluctuation (RMSF) reflects the flexibility of individual amino acid residues. As shown in Figure 6E, the RMSF values for the EZH2–**SU101** complex were mostly below 1 nm, while those for the G9a–**SU101** complex remained mostly below 0.5 nm, indicating that both complexes are relatively rigid.

The free energy landscape (FEL) of each complex is presented in Figure 6F, with colour mapping representing different free energy values. The X-axis denotes PC1 (RMSD, in nm), and the Y-axis denotes PC2 (Rg, in nm). Red regions correspond to higher free energy, whereas blue regions indicate lower free energy. The EZH2–**SU101** complex exhibited low free energy minima at PC1 = 0.52 nm and PC2 = 3.34 nm, and the G9a–**SU101** complex at PC1 = 0.45 nm and PC2 = 3.77 nm, suggesting that these conformations are energetically more stable.

Collectively, the molecular dynamics simulations demonstrate that both the EZH2–**SU101** and G9a–**SU101** complexes are stably bound, with favorable hydrogen-bonding networks. These findings support a strong binding interaction between the small molecule **SU101** and its target proteins.

### 2.7. Pharmacokinetic Evaluation of Compound **SU101**

To evaluate the pharmacokinetic properties of compound **SU101**, a single oral administration (*po*) at a dose of 6.25 mg/kg and a single intravenous (*iv*) bolus injection at a dose of 2.5 mg/kg were performed in Sprague–Dawley (SD) rats. The pharmacokinetic parameters are summarized in Table 4. Following oral administration, compound **SU101** reached peak plasma concentration (C_max_) of 1406.645 ng/mL at 0.167 h (T_max_). The total systemic exposure, as measured by AUC_0–24_, was 2021.51 ng·h/mL. The elimination half-life (t_1/2_) of compound **SU101** in rats was 0.94 h, and the absolute bioavailability was determined to be 94.3%, indicating that compound **SU101** is well absorbed after oral administration.

### 2.8. Plasma Protein Binding

The plasma protein binding (PPB) of compound **SU101** in Kunming mice plasma was determined using a classical equilibrium dialysis apparatus. The results showed that **SU101** exhibited moderate PPB (84.6%). Since the therapeutic efficacy of a drug is related to the concentration of free drug, the moderate %PPB may be beneficial for its in vivo antitumor activity.

### 2.9. Acute Toxicity Evaluation of SU101

To evaluate the safety profile of **SU101**, acute toxicity studies were performed using the up-and-down method [33], and the LD_50_ value was calculated using AOT425StatPgm V1.0 software. The results demonstrated that the intraperitoneal LD_50_ of **SU101** in KM mice was 31.02 mg/kg.

## 3. Materials and Methods

### 3.1. Chemistry

All reactions were carried out with magnetic stirring and in dried glassware. The reactions were monitored by thin-layer chromatography (TLC: HG/T2354-92, GF254), and compounds were visualized by TLC with UV light. All chemicals and solvents were purchased from commercial sources and used without purification treatment. Analytical TLC was carried out on 0.20 mm silica gel plates (Haiyang, Qingdao, Shandong, China) with the QF-254 UV indicator. Column chromatography was conducted using Haiyang silica gel 60 (300–400 mesh). Melting points were determined with an X-4 apparatus and were uncorrected. The nuclear magnetic resonance (NMR) spectra were recorded on a Bruker 600 MHz or 400 MHz spectrometer in CDCl_3_ using tetramethylsilane as an internal standard. Peak multiplicity of NMR signals was as follows: s, singlet; d, doublet; t, triplet; q, quartet; and m, multiplet. Chemical shift (δ): ppm relative to Me4Si (internal standard). Coupling constant: J (Hz). High-resolution mass spectra (HRMS) of all target compounds were recorded by a Waters Q-TOF Premier spectrometer with acetonitrile and water as solvents. Electrospray ionization mass spectrometry (ESI-MS) analyses were recorded in an Agilent 1100 Series MSD Trap SL (Santa Clara, CA, USA).

Synthesis of the intermediate is shown in S4 in the Supporting Materials.

***General procedure 1**** for the Synthesis of Compounds **SU101–SU404***. A solution of intermediate ***9a–9c**** or **10*** (0.42 mmol), the corresponding secondary amine (0.63 mmol), 4-methylbenzenesulfonic acid 0.144 g (0.84 mmol) in *n*-BuOH (10 mL) was degassed and purged with Ar 3 times and the mixture was stirred at 110 °C for 5–15 h. After being cooled to room temperature, *n*-BuOH (10 mL) was added to the mixture. The mixture was washed with saturated NaHCO_3_ solution, water, and brine, dried over MgSO_4_, filtered and concentrated *in vacuum* to give crude product.

*4-methoxy-3-(((6-methoxy-2-(piperidin-1-yl)-7-(3-(pyrrolidin-1-yl)propoxy)quinazolin-4-yl)amino)methyl)-6-methylpyridin-2(1H)-one (**SU101**)* was synthesized using intermediate **9a** and piperidine as starting materials. The crude product was purified by prep-HPLC (*Method 1* described in S5 in Supporting Material) to afford pure compound as white solid (80 mg; 35%). **m.p.** 145–146 °C. **^1^H NMR** (400 MHz, CDCl_3_): δ 13.01 (s, 1H), 6.97 (s, 1H), 6.88 (s, 1H), 5.86 (s, 1H), 4.69 (d, *J* = 4.8 Hz, 2H), 4.14 (t, *J* = 6.7 Hz, 2H), 3.81 (s, 10H), 2.62 (t, *J* = 7.4 Hz, 2H), 2.51 (s, 4H), 2.28 (s, 3H), 2.12–2.05 (m, 2H), 1.81–1.73 (m, 4H), 1.63 (s, 6H). **^13^C NMR** (101 MHz, CDCl_3_): *δ* 166.4, 166.3, 159.0, 153.8, 145.9, 145.2, 107.3, 106.5, 103.5, 102.7, 94.6, 67.2, 56.6, 56.0, 54.2, 53.0, 44.9, 34.6, 28.6, 26.1, 25.1, 23.5, 19.5. **HRMS** (ESI) calcd for C_29_H_40_N_6_O_4_ [M + H]^+^: 537.3189, found 537.31909.

*4-methoxy-3-(((6-methoxy-2-(4-methyl-1,4-diazepan-1-yl)-7-(3-(pyrrolidin-1-yl)propoxy)quinazolin-4-yl)amino)methyl)-6-methylpyridin-2(1H)-one (**SU102**)* was synthesized using intermediate **9a** and N-methylhomopiperazine as starting materials. The crude product was purified by prep-HPLC (*Method 2* described in S5 in Supporting Material) to afford pure compound as white solid (100 mg; 42%). **m.p.** 120–121 °C. **^1^H NMR** (400 MHz, CDCl_3_): *δ* 12.40 (s, 1H), 6.93 (s, 1H), 6.86 (s, 1H), 6.78 (s, 1H), 5.93 (s, 1H), 4.72 (d, *J* = 5.0 Hz, 2H), 4.16 (t, *J* = 6.8 Hz, 2H), 4.06 (brs, 2H), 3.92 (t, *J* = 6.3 Hz, 2H), 3.87 (s, 3H), 3.84 (s, 3H), 2.75 (t, *J* = 4.2 Hz, 2H), 2.63 (t, *J* = 7.5 Hz, 2H), 2.59 (t, *J* = 5.4 Hz, 2H), 2.53 (brs, 4H), 2.39 (s, 3H), 2.33 (s, 3H), 2.14–2.09 (m, 2H), 2.06–2.03 (m, 2H), 1.78 (brs, 4H). **^13^C NMR** (101 MHz, CDCl_3_): *δ* 166.7, 165.8, 160.5, 158.6, 153.9, 146.2, 140.6, 120.4, 106.4, 103.2, 100.3, 94.8, 67.1, 58.4, 57.1, 56.7, 56.2, 54.1, 52.8, 46.5, 35.1, 27.8, 27.1, 23.5, 19.7. **ESI-MS** *m*/*z*: 566.2 [M + H]^+^.

*3-(((2-(4,4-Difluoropiperidin-1-yl)-6-methoxy-7-(3-(pyrrolidin-1-yl)propoxy)quinazolin-4-yl)amino)methyl)-4-methoxy-6-methylpyridin-2(1H)-one (**SU103**)* was synthesized using intermediate **9a** and 4,4-difluoropiperidine hydrochloride as starting materials. The crude product was purified by prep-HPLC (*Method 3* described in S5 in Supporting Material) to afford pure compound as white solid (123 mg; 51%). **m.p.** 98–100 °C. **^1^H NMR** (400 MHz, CDCl_3_): *δ* 12.38 (s,1H), 6.87 (s,3H), 5.91 (s,1H), 4.70 (d, *J* = 5.2 Hz, 2H),4.15 (t, *J* = 6.7 Hz, 2H), 4.04 (t, *J* = 5.5 Hz, 4H), 3.86 (s, 3H), 3.84 (s, 3H), 2.69–2.66 (m, 2H), 2.58 (s, 4H), 2.32 (s, 3H), 2.15–2.08 (m, 2H), 2.05–1.95 (m, 4H), 1.80 (s, 4H). **^13^C NMR** (101 MHz, CDCl_3_): *δ* 165.2, 165.1, 158.2, 157.3, 157.2, 152.9, 144.6, 122.0 (t, *J* = 252 Hz), 106.3, 105.9, 102.9, 101.3, 93.6, 66.1, 55.6, 55.1, 53.1, 51.9, 40.0 (t, *J* = 4.3 Hz), 33.6, 33.0 (t, *J* = 21.9 Hz), 28.7, 27.3, 22.5, 18.5. **ESI-MS** *m*/*z*: 573.1 [M + H]^+^.

*3-(((2-(Azepan-1-yl)-6-methoxy-7-(3-(pyrrolidin-1-yl)propoxy)quinazolin-4-yl)amino)methyl)-4-methoxy-6-methylpyridin-2(1H)-one (**SU104**)* was synthesized using intermediate **9a** and hexamethyleneimine as starting materials. The crude product was purified by prep-HPLC (*Method 4* described in S5 in Supporting Material) to afford pure compound as white solid (106 mg; 46%). **m.p.** 110–112 °C. **^1^H NMR** (400 MHz, CDCl_3_): *δ* 12.74 (s, 1H), 7.07 (brs, 2H), 5.90 (s, 1H), 4.67 (s, 2H), 4.16 (t, *J* = 6.6 Hz, 2H), 3.82 (brs, 10H), 2.64 (t, *J* = 7.4 Hz, 2H), 2.54 (s, 4H), 2.31 (s, 3H), 2.13–2.06 (m, 4H), 1.80–1.78 (m, 4H), 1.54 (s, 4H). **^13^C NMR** (101 MHz, CDCl_3_): *δ* 166.5, 166.2, 158.7, 153.9, 146.1, 145.2, 107.0, 106.8, 103.3, 103.1, 94.7, 67.3, 56.8, 56.0, 54.2, 53.0, 47.2, 34.7, 28.5, 28.3, 27.3, 23.5, 19.5. **ESI-MS** *m*/*z*: 551.2 [M + H]^+^.

*3-(((2-((2-(Dimethylamino)ethyl)(methyl)amino)-6-methoxy-7-(3-(pyrrolidin-1-yl)propoxy)quinazolin-4-yl)amino)methyl)-4-methoxy-6-methylpyridin-2(1H)-one (**SU105**)* was synthesized using intermediate **9a** and *N,N,N’*-trimethylethylenediamine as starting materials. The crude product was purified by prep-HPLC (*Method 5* described in S5 in Supporting Material) to afford pure compound as white solid (88 mg; 38%). **m.p.** 144–146 °C. **^1^H NMR** (400 MHz, CDCl_3_): *δ* 12.80 (s, 1H), 7.06 (s, 1H), 6.98 (s, 2H), 5.91 (s, 1H), 4.68 (d, *J* = 4.5 Hz, 2H), 4.15 (t, *J* = 6.7 Hz, 2H), 3.88–3.83 (m, 5H), 3.82 (s, 3H), 3.23 (s, 3H), 2.66 (t, *J* = 7.4 Hz, 2H), 2.61–2.56 (m, 6H), 2.35 (s, 6H), 2.32 (s, 3H), 2.14–2.07 (m, 2H), 1.81–1.78 (m, 4H). **^13^C NMR** (101 MHz, CDCl_3_): *δ* 166.4, 166.3, 158.9, 158.2, 153.9, 146.1, 145.2, 106.8, 106.1, 103.3, 103.1, 96.7, 67.2, 56.9, 56.8, 56.0, 54.2, 53.0, 47.4, 45.8, 35.6, 34.9, 28.4, 23.5, 19.5. **ESI-MS** *m*/*z*: 554.3 [M + H]^+^.

*3-(((6-Methoxy-2-(piperidin-1-yl)-7-(3-(pyrrolidin-1-yl)propoxy)quinazolin-4-yl)amino)methyl)-4-methyl-6-propylpyridin-2(1H)-one (**SU201**)* was synthesized using intermediate **9b** and piperidine as starting materials. The crude product was purified by prep-HPLC (*Method 1* described in S5 in Supporting Material) to afford pure compound as white solid (127 mg; 55%). **m.p.** 109–111 °C. **^1^H NMR** (400 MHz, CDCl_3_): *δ* 12.02 (s, 1H), 6.89 (s, 1H), 6.81 (s, 1H), 6.73 (t, *J* = 5.4 Hz, 1H), 5.95 (s, 1H), 4.78 (d, *J* = 5.4 Hz, 2H), 4.17 (t, *J* = 6.7 Hz, 2H), 4.05 (t, *J* = 5.4 Hz, 4H), 3.85 (s, 3H), 2.65 (t, *J* = 7.3 Hz, 2H), 2.55–2.49 (m, 5H), 2.37 (s, 3H), 2.15–2.10 (m, 2H), 2.08–1.97 (m, 4H), 1.79 (s, 4H), 1.76–1.67 (m, 2H), 1.26 (s, 2H), 0.92 (t, *J* = 7.3 Hz, 3H). **^13^C NMR** (101 MHz, CDCl_3_): *δ* 165.5, 159.1, 158.3, 154.1, 149.9, 149.1, 146.8, 145.9, 122.9, 109.1, 107.0, 103.6, 102.0, 67.3, 56.6, 54.2, 53.0, 41.2, 37.1, 35.0, 34.0, 29.7, 28.4, 23.5, 22.0, 19.7, 13.6. **ESI-MS** *m*/*z*: 549.3 [M + H]^+^.

*3-(((6-Methoxy-2-(4-methyl-1,4-diazepan-1-yl)-7-(3-(pyrrolidin-1-yl)propoxy)quinazolin-4-yl)amino)methyl)-4-methyl-6-propylpyridin-2(1H)-one (**SU202**)* was synthesized using intermediate **9b** and N-methylhomopiperazine as starting materials. The crude product was purified by prep-HPLC (*Method 2* described in S5 in Supporting Material) to afford pure compound as white solid (99 mg; 41%). **m.p.** 133–135 °C. **^1^H NMR** (400 MHz, CDCl_3_): *δ* 10.47 (s, 1H), 6.87 (s, 1H), 5.94 (s, 1H), 4.75 (d, *J* = 5.4 Hz, 2H), 4.20 (t, *J* = 6.4 Hz, 2H), 4.11 (s, 2H), 3.97 (s, 2H), 3.86 (s, 3H), 2.85 (s, 4H), 2.78 (s, 2H), 2.70 (s, 4H), 2.49 (t, *J* = 7.5 Hz, 2H), 2.45 (s, 3H), 2.34 (s, 3H), 2.24–2.20 (m, 2H), 2.14 (s, 2H), 1.89 (s, 4H), 1.72–1.66 (m, 2H). **ESI-MS** *m*/*z*: 578.3 [M + H]^+^.

*3-(((2-(4,4-Difluoropiperidin-1-yl)-6-methoxy-7-(3-(pyrrolidin-1-yl)propoxy)quinazolin-4-yl)amino)methyl)-4-methyl-6-propylpyridin-2(1H)-one (**SU203**)* was synthesized using intermediate **9b** and 4,4-difluoropiperidine hydrochloride as starting materials. The crude product was purified by prep-HPLC (*Method 3* described in S5 in Supporting Material) to afford pure compound as white solid (76 mg; 31%). **m.p.** 125–127 °C. **^1^H NMR** (400 MHz, CDCl_3_): *δ* 11.71 (s, 1H), 6.89 (s, 1H), 6.81 (s, 1H), 6.77 (s, 1H), 5.94 (s, 1H), 4.78 (d, *J* = 5.4 Hz, 2H), 4.17 (t, *J* = 6.5 Hz, 2H), 4.05 (t, *J* = 5.6 Hz, 4H), 3.85 (s, 3H), 2.76–2.74 (m, 2H), 2.68 (s, 4H), 2.50 (t, *J* = 7.5 Hz, 2H), 2.37 (s, 3H), 2.21–2.14 (m, 2H), 2.07–1.97 (m, 4H), 1.85 (s, 4H), 1.75–1.66 (m, 2H). **^13^C NMR** (101 MHz, CDCl_3_): *δ* 165.3, 159.1, 158.2, 154.0, 149.9, 149.0, 146.6, 145.9, 123.0 (t, *J* = 240 Hz), 109.1, 107.0, 103.7, 102.0, 67.0, 56.6, 54.1, 53.0, 41.2, 37.2, 35.0, 34.0 (t, *J* = 22.8 Hz), 27.9, 23.5, 21.9, 19.7, 14.0, 13.6. **ESI-MS** *m*/*z*: 585.2 [M + H]^+^.

*3-(((2-(Azepan-1-yl)-6-methoxy-7-(3-(pyrrolidin-1-yl)propoxy)quinazolin-4-yl)amino)methyl)-4-methyl-6-propylpyridin-2(1H)-one (**SU204**)* was synthesized using intermediate **9b** and hexamethyleneimine as starting materials. The crude product was purified by prep-HPLC (*Method 4* described in S5 in Supporting Material) to afford pure compound as white solid (104 mg; 44%). **m.p.** 136–138 °C. **^1^H NMR** (400 MHz, CDCl_3_): *δ* 11.87 (s, 1H), 6.92 (s, 1H), 6.79 (s, 1H), 6.61 (s, 1H), 5.94 (s, 1H), 4.80 (d, *J* = 5.4 Hz, 2H), 4.16 (t, *J* = 6.8 Hz, 2H), 3.87–3.83 (m, 7H), 2.63 (t, *J* = 7.4 Hz, 2H), 2.53–2.49 (m, 6H), 2.36 (s, 3H), 2.14–2.07 (m, 2H), 1.83 (brs, 4H), 1.80–1.78 (m, 4H), 1.74–1.67 (m, 2H), 1.57 (brs, 4H), 0.94 (t, *J* = 7.3 Hz, 3H). **^13^C NMR** (101 MHz, CDCl_3_): *δ* 165.5, 158.6, 154.7, 154.0, 150.1, 146.6, 145.0, 123.0, 109.1, 103.0, 102.4, 67.3, 56.7, 54.2, 53.0, 47.0, 37.0, 35.0, 28.6, 28.5, 27.5, 23.5, 22.0, 19.8, 13.6. **ESI-MS** *m*/*z*: 563.3 [M + H]^+^.

*3-(((2-((2-(Dimethylamino)ethyl)(methyl)amino)-6-methoxy-7-(3-(pyrrolidin-1-yl)propoxy)quinazolin-4-yl)amino)methyl)-4-methyl-6-propylpyridin-2(1H)-one (**SU205**)* was synthesized using intermediate **9b** and *N,N,N’*-trimethylethylenediamine as starting materials. The crude product was purified by prep-HPLC (*Method 5* described in S5 in Supporting Material) to afford pure compound as white solid (88 mg; 37%). **m.p.** 150–151 °C. **^1^H NMR** (400 MHz, CDCl_3_): *δ* 12.2 (s, 1H), 6.90 (s, 1H), 6.80 (s, 1H), 6.61 (s, 1H), 5.95 (s, 1H), 4.79 (d, *J* = 5.2 Hz, 2H), 4.16 (t, *J* = 6.7 Hz, 2H), 3.89–3.82 (m, 5H), 3.25 (s, 3H), 2.64 (t, *J* = 7.4 Hz, 2H), 2.59 (t, *J* = 7.5 Hz, 2H), 2.53–2.49 (m, 6H), 2.35 (s, 3H), 2.34 (s, 6H), 2.14–2.07 (m, 2H), 1.79–1.78 (m, 4H), 1.74–1.66 (m, 2H), 0.93 (t, *J* = 7.3 Hz, 3H). **^13^C NMR** (101 MHz, CDCl_3_): *δ* 165.5, 158.8, 154.0, 150.2, 146.8, 145.2, 122.8, 109.1, 106.6, 103.1, 102.3, 67.2, 57.0, 56.7, 54.2, 53.0, 47.5, 45.9, 37.1, 35.6, 35.0, 28.4, 23.5, 21.9, 19.9, 13.6. **ESI-MS** *m*/*z*: 566.3 [M + H]^+^.

*6-Ethyl-3-(((6-methoxy-2-(piperidin-1-yl)-7-(3-(pyrrolidin-1-yl)propoxy)quinazolin-4-yl)amino)methyl)-4-methylpyridin-2(1H)-one (**SU301**)* was synthesized using intermediate **9c** and piperidine as starting materials. The crude product was purified by prep-HPLC (*Method 1* described in S5 in Supporting Material) to afford pure compound as white solid (74 mg; 33%). **m.p.** 161–163 °C. **^1^H NMR** (400 MHz, CDCl_3_): *δ* 12.3 (s, 1H), 6.93 (s, 1H), 6.80 (s, 1H), 6.70 (s, 1H), 5.94 (s, 1H), 4.79 (d, *J* = 5.6 Hz, 2H), 4.15 (t, *J* = 6.7 Hz, 2H), 3.88–3.87 (m, 4H), 3.83 (s, 3H), 2.65 (t, *J* = 7.4 Hz, 2H), 2.61–2.55 (m, 6H), 2.38 (s, 3H), 2.14–2.07 (m, 2H), 1.80–1.77 (m, 4H), 1.65 (brs, 6H), 1.27 (t, *J* = 7.6 Hz, 3H). **^13^C NMR** (101 MHz, CDCl_3_): *δ* 165.5, 158.8, 158.7, 154.0, 150.1, 148.0, 145.4, 123.1, 108.2, 106.7, 103.3, 102.2, 67.2, 56.6, 54.2, 53.0, 45.2, 37.1, 28.4, 26.2, 26.1, 25.1, 23.5, 19.8, 12.7. **ESI-MS** *m*/*z*: 535.1 [M + H]^+^.

*6-Ethyl-3-(((6-methoxy-2-(4-methyl-1,4-diazepan-1-yl)-7-(3-(pyrrolidin-1-yl)propoxy)quinazolin-4-yl)amino)methyl)-4-methylpyridin-2(1H)-one (**SU302**)* was synthesized using intermediate **9c** and N-methylhomopiperazine as starting materials. The crude product was purified by prep-HPLC (*Method 2* described in S5 in Supporting Material) to afford pure compound as white solid (71 mg; 30%). **m.p.** 147–149 °C. **^1^H NMR** (400 MHz, CDCl_3_): *δ* 12.85 (s, 1H), 6.89 (s, 2H), 6.73 (s, 1H), 5.92 (s, 1H), 4.78 (s, 2H), 4.14 (s, 2H), 4.05 (s, 2H), 3.93 (s, 2H), 3.79 (s, 3H), 2.74 (s, 2H), 2.60 (s, 4H), 2.50 (s, 5H), 2.38 (s, 2H), 2.35 (s, 2H), 2.06 (s, 4H), 1.77 (s, 4H), 1.23 (s, 3H). **^13^C NMR** (101 MHz, CDCl_3_): *δ* 165.6, 158.7, 158.6, 153.9, 150.3, 149.4, 148.3, 145.1, 122.8, 108.1, 106.7, 103.2, 102.4, 67.2, 59.1, 57.5, 56.6, 54.1, 52.9, 46.7, 46.1, 45.7, 36.9, 28.5, 27.9, 26.0, 23.5, 19.8, 12.7. **ESI-MS** *m*/*z*: 564.2 [M + H]^+^.

*3-(((2-(4,4-Difluoropiperidin-1-yl)-6-methoxy-7-(3-(pyrrolidin-1-yl)propoxy)quinazolin-4-yl)amino)methyl)-6-ethyl-4-methylpyridin-2(1H)-one (**SU303**)* was synthesized using intermediate **9c** and 4,4-difluoropiperidine hydrochloride as starting materials. The crude product was purified by prep-HPLC (*Method 3* described in S5 in Supporting Material) to afford pure compound as white solid (103 mg; 43%). **m.p.** 131–132 °C. **^1^H NMR** (400 MHz, CDCl_3_): *δ* 12.78 (s, 1H), 6.89 (s, 1H), 6.88 (s, 1H), 6.80 (t, *J* = 5.2 Hz, 1H), 5.94 (s, 1H), 4.77 (d, *J* = 5.2Hz, 2H), 4.16 (t, *J* = 6.7 Hz, 2H), 4.04 (t, *J* = 5.2 Hz, 4H), 3.82 (s, 3H), 2.64–2.60 (m, 2H), 2.58–2.56 (m, 2H), 2.52 (s, 4H), 2.36 (s, 3H), 2.13–2.06 (m, 2H), 2.03–1.98 (m, 4H), 1.78 (s, 3H). **^13^C NMR** (101 MHz, CDCl_3_): *δ* 165.7, 159.1, 158.3, 154.1, 150.2, 149.2, 148.3, 145.8, 122.8 (t, *J* = 240 Hz), 108.2, 106.9, 103.6, 102.1, 67.3, 56.6, 54.2, 52.9, 41.2, 37.1, 34.0 (t, *J* = 17.3 Hz), 28.5, 26.1, 23.5, 19.7, 12.7. **ESI-MS** *m*/*z*: 571.2 [M + H]^+^.

*3-(((2-(Azepan-1-yl)-6-methoxy-7-(3-(pyrrolidin-1-yl)propoxy)quinazolin-4-yl)amino)methyl)-6-ethyl-4-methylpyridin-2(1H)-one (**SU304**)* was synthesized using intermediate **9c** and hexamethyleneimine as starting materials. The crude product was purified by prep-HPLC (*Method 4* described in S5 in Supporting Material) to afford pure compound as white solid (83 mg; 36%). **m.p.** 145–146 °C. **^1^H NMR** (400 MHz, CDCl_3_): *δ* 12.07 (s, 1H), 7.02 (s, 1H), 6.82 (s, 2H), 5.95 (s, 1H), 4.79 (d, *J* = 5.2 Hz, 2H), 4.17 (t, *J* = 6.7 Hz, 2H), 3.86 (t, *J* = 5.8 Hz, 4H), 3.83 (s, 3H), 2.66 (t, *J* = 7.4 Hz, 2H), 2.61–2.55 (m, 6H), 2.36 (s, 3H), 2.15–2.08 (m, 2H), 1.83–1.79 (m, 8H), 1.57 (brs, 4H), 1.27 (t, *J* = 7.5 Hz, 3H). **^13^C NMR** (101 MHz, CDCl_3_): *δ* 165.4, 158.6, 154.0, 148.0, 145.2, 122.9, 108.1, 106.4, 103.1, 102.5, 94.3, 67.3, 56.7, 54.2, 53.0, 47.1, 37.1, 28.5, 28.4, 27.5, 26.2, 23.5, 19.8, 12.7. **ESI-MS** *m*/*z*: 549.3 [M + H]^+^.

*3-(((2-((2-(Dimethylamino)ethyl)(methyl)amino)-6-methoxy-7-(3-(pyrrolidin-1-yl)propoxy)quinazolin-4-yl)amino)methyl)-6-ethyl-4-methylpyridin-2(1H)-one (**SU305**)* was synthesized using intermediate **9c** and *N,N,N’*-trimethylethylenediamine as starting materials. The crude product was purified by prep-HPLC (*Method 5* described in S5 in Supporting Material) to afford pure compound as white solid (111 mg; 48%). **m.p.** 158–160 °C. **^1^H NMR** (400 MHz, CDCl_3_): *δ* 11.2 (s, 1H), 7.00 (s, 1H), 6.83 (s, 2H), 5.95 (s, 1H), 4.78 (d, *J* = 5.0 Hz, 2H), 4.17 (t, *J* = 6.6 Hz, 2H), 3.91–3.83 (m, 5H), 3.27 (s, 3H), 2.74 (brs, 2H), 2.64–2.54 (m, 8H), 2.36 (s, 6H), 2.35 (s, 3H), 2.20–2.12 (m, 2H), 1.83 (s, 4H), 1.27 (t, *J* = 7.6 Hz, 3H). **^13^C NMR** (101 MHz, CDCl_3_): *δ* 165.0, 158.7, 153.9, 150.4, 147.7, 145.5, 122.8, 108.1, 106.2, 103.1, 102.2, 67.1, 56.9, 56.6, 54.1, 53.0, 47.5, 45.8, 37.3, 35.8, 29.7, 26.2, 23.5, 19.9, 12.6. **ESI-MS** *m*/*z*: 552.2 [M + H]^+^.

*3-(((6-Methoxy-2-(piperidin-1-yl)-7-(3-(pyrrolidin-1-yl)propoxy)quinazolin-4-yl)amino)methyl)-4-methyl-5,6,7,8-tetrahydroquinolin-2(1H)-one (**SU401**)* was synthesized using intermediate **10** and piperidine as starting materials. The crude product was purified by prep-HPLC (*Method 1* described in S5 in Supporting Material) to afford pure compound as white solid (92 mg; 39%). **m.p.** 130–132 °C. **^1^H NMR** (400 MHz, CDCl_3_): *δ* 12.6 (s, 1H), 6.93 (s, 1H), 6.86 (s, 2H), 4.79 (d, *J* = 5.4 Hz, 2H), 4.15 (t, *J* = 6.7 Hz, 2H), 3.88–3.86 (m, 4H), 3.82 (s, 3H), 2.95 (s, 2H), 2.64 (t, *J* = 7.4 Hz, 2H), 2.55 (brs, 4H), 2.41 (brs, 2H), 2.24 (s, 3H), 2.14–2.07 (m, 2H), 1.80–1.77 (m, 4H), 1.72 (brs, 4H), 1.65 (brs, 6H). **^13^C NMR** (101 MHz, CDCl_3_): *δ* 163.8, 158.9, 153.9, 150.9, 145.3, 140.2, 122.2, 114.7, 106.6, 103.4, 102.5, 67.2, 56.6, 54.2, 53.0, 45.2, 36.7, 28.4, 27.5, 26.1, 25.1, 24.9, 23.5, 22.3, 16.8. **ESI-MS** *m*/*z*: 561.2 [M + H]^+^.

*3-(((6-Methoxy-2-(4-methyl-1,4-diazepan-1-yl)-7-(3-(pyrrolidin-1-yl)propoxy)quinazolin-4-yl)amino)methyl)-4-methyl-5,6,7,8-tetrahydroquinolin-2(1H)-one (**SU402**)* was synthesized using intermediate **10** and N-methylhomopiperazine as starting materials. The crude product was purified by prep-HPLC (*Method 2* described in S5 in Supporting Material) to afford pure compound as white solid (82 mg; 33%). **m.p.** 139–140 °C. **^1^H NMR** (400 MHz, CDCl_3_): *δ* 12.3 (s, 1H), 7.00 (s, 1H), 6.86 (s, 2H), 4.78 (d, *J* = 5.1 Hz, 2H), 4.17 (t, *J* = 6.6 Hz, 2H), 4.07 (brs, 2H), 3.94 (t, *J* = 6.1 Hz, 2H), 3.83 (s, 3H), 2.89 (s, 2H), 2.78 (brs, 2H), 2.69 (t, *J* = 7.3 Hz, 2H), 2.65–2.62 (m, 2H), 2.59 (brs, 4H), 2.42 (s, 5H), 2.25 (s, 3H), 2.16–2.11 (m, 2H), 2.09–2.07 (m, 2H), 1.81 (s, 4H), 1.73 (s, 4H). **^13^C NMR** (101 MHz, CDCl_3_): *δ* 163.7, 158.7, 154.1, 150.9, 145.4, 140.2, 122.0, 114.7, 106.4, 103.3, 102.6, 67.2, 59.0, 57.4, 56.7, 54.2, 53.0, 46.6, 46.1, 45.6, 36.8, 28.3, 27.6, 27.5, 25.0, 23.5, 22.3, 16.9. **ESI-MS** *m*/*z*: 590.2 [M + H]^+^.

*3-(((2-(Azepan-1-yl)-6-methoxy-7-(3-(pyrrolidin-1-yl)propoxy)quinazolin-4-yl)amino)methyl)-4-methyl-5,6,7,8-tetrahydroquinolin-2(1H)-one (**SU403**)* was synthesized using intermediate **10** and hexamethyleneimine as starting materials. The crude product was purified by prep-HPLC (*Method 4* described in S5 in Supporting Material) to afford pure compound as white solid (101 mg; 42%). **m.p.** 124–126 °C. **^1^H NMR** (400 MHz, CDCl_3_): *δ* 12.85 (s, 1H), 7.02 (s, 1H), 6.92 (s, 2H), 4.78 (d, *J* = 5.0 Hz, 2H), 4.16 (t, *J* = 6.7 Hz, 2H), 3.85 (t, *J* = 5.8 Hz, 4H), 3.80 (s, 3H), 2.88 (s, 2H), 2.64 (t, *J* = 7.4 Hz, 2H), 2.54 (s, 4H), 2.40 (s, 2H), 2.24 (s, 3H), 2.14–2.07 (m, 2H), 1.82–1.75 (m, 8H), 1.70 (brs, 4H), 1.58–1.56 (m, 4H). **^13^C NMR** (101 MHz, CDCl_3_): *δ* 163.8, 158.7, 154.0, 151.0, 145.1, 140.5, 121.8, 114.8, 106.2, 103.1, 102.9, 67.3, 56.8, 54.2, 53.0, 47.1, 36.7, 28.5, 28.4, 27.5, 24.9, 23.5, 22.3, 22.3, 16.8. **ESI-MS** *m*/*z*: 575.2 [M + H]^+^.

*3-(((2-((2-(Dimethylamino)ethyl)(methyl)amino)-6-methoxy-7-(3-(pyrrolidin-1-yl)propoxy)quinazolin-4-yl)amino)methyl)-4-methyl-5,6,7,8-tetrahydroquinolin-2(1H)-one (**SU404**)* was synthesized using intermediate **10** and *N,N,N’*-trimethylethylenediamine as starting materials. The crude product was purified by prep-HPLC (*Method 5* described in S5 in Supporting Material) to afford pure compound as white solid (85 mg; 35%). **m.p.** 143–145 °C. **^1^H NMR** (400 MHz, CDCl_3_): *δ* 11.98 (s, 1H), 7.14 (s, 1H), 6.95 (s, 1H), 4.76 (d, *J* = 4.3 Hz, 2H), 4.19 (t, *J* = 6.4 Hz, 2H), 3.89–3.85 (m, 2H), 3.83 (s, 3H), 3.28 (s, 3H), 2.85 (s, 4H), 2.75 (s, 4H), 2.65 (t, *J* = 6.6 Hz, 2H), 2.43 (s, 2H), 2.38 (s, 6H), 2.26 (s, 3H), 2.22–2.18 (m, 2H), 1.88 (s, 4H), 1.72 (s, 4H). **^13^C NMR** (101 MHz, CDCl_3_): *δ* 163.4, 158.6, 153.9, 151.4, 145.7, 140.5, 121.2, 114.8, 105.4, 103.1, 102.8, 66.9, 56.9, 56.7, 54.0, 52.9, 47.6, 45.7, 37.2, 36.1, 29.7, 27.6, 24.9, 23.5, 22.2, 22.2, 17.0. **ESI-MS** *m*/*z*: 578.3 [M + H]^+^.

***General procedure 2**** for the Synthesis of Compounds **UA01~UA03***.

To a 50 mL two-necked flask were added triphosgene (BTC, 0.19 g, 0.65 mmol) and dry dichloromethane (4 mL). The flask was placed in a cold bath and cooled to −80 °C. A solution of **12a** or **12b** (1.3 mmol) in triethylamine (4 mL), pyridine (4 mL), and dichloromethane (4 mL) was added dropwise, while the internal temperature was maintained below −70 °C. After the addition was complete, stirring was continued for 5 min, and the mixture was then allowed to warm to room temperature slowly. The mixture was subsequently heated in an oil bath at 60 °C for 2 h. Then, intermediate **14** (0.2 g, 1.3 mmol) was added to the flask, and the reaction was stirred at 60 °C overnight. The next day, the reaction was complete, although some starting material remained. The solvent was removed under reduced pressure, and the residue was poured into water (50 mL). The aqueous phase was extracted with dichloromethane (30 mL × 3). The combined organic layers were washed with water (50 mL) and brine, then dried over anhydrous magnesium sulfate. Evaporation of the solvent afforded the crude product.

*1-((4,6-dimethyl-2-oxo-1,2-dihydropyridin-3-yl)methyl)-3-(6-methoxy-2-(piperidin-1-yl)-7-(3-(pyrrolidin-1-yl)propoxy)quinazolin-4-yl)urea (**UA01**)*. The crude product was purified by prep-HPLC (*Method 6* described in S5 in Supporting Material) to afford pure compound as pale yellow solid (79 mg; 11%). m.p. 153–155 °C. **^1^H NMR** (400 MHz, CDCl_3_) *δ* 12.28 (s, 1H), 9.71 (t, *J* = 4.8 Hz, 1H), 8.48 (s, 1H), 7.20 (s, 1H), 6.86 (s, 1H), 5.89 (s, 1H), 4.54 (d, *J* = 4.8 Hz, 2H), 4.16 (t, *J* = 6.6 Hz, 2H), 3.96 (s, 3H), 3.55 (s, 4H), 2.67 (t, *J* = 7.3 Hz, 2H), 2.58 (s, 4H), 2.31 (s, 3H), 2.25 (s, 3H), 2.16–2.09 (m, 2H), 1.80 (s, 4H), 1.50 (s, 6H). **^13^C NMR** (101 MHz, CDCl_3_) *δ* 164.7, 156.9, 155.6, 155.3, 155.1, 151.5, 150.6, 146.7, 143.2, 121.8, 109.0, 106.6, 102.6, 101.1, 67.3, 56.5, 54.1, 52.9, 45.4, 44.8, 35.7, 28.2, 25.7, 23.5, 19.5, 18.7. **ESI-MS** *m*/*z*: 564.2 [M + H]^+^.

*1-(6-Methoxy-2-(piperidin-1-yl)-7-(3-(pyrrolidin-1-yl)propoxy)quinazolin-4-yl)-3-((4-methoxy-6-methyl-2-oxo-1,2-dihydropyridin-3-yl)methyl)urea (**UA02**).* The crude product was purified by prep-HPLC (*Method 1* described in S5 in Supporting Material) to afford pure compound as pale yellow solid (50 mg; 6%). m.p. 164–166 °C. **^1^H NMR** (400 MHz, CDCl_3_) *δ* 12.82 (s, 1H), 9.53 (s, 1H), 8.41 (s, 1H), 7.20 (s, 1H), 6.85 (s, 1H), 5.87 (s, 1H), 4.50 (s, 2H), 4.16 (s, 2H), 3.97 (s, 3H), 3.83 (s, 3H), 3.53 (s, 4H), 2.61 (t, *J* = 6.6 Hz, 2H), 2.51 (s, 4H), 2.30 (s, 3H), 2.11 (t, *J* = 6.4 Hz, 2H), 1.77 (s, 4H), 1.51 (s, 2H),1.45 (s, 4H). **^13^C NMR** (101 MHz, CDCl_3_) *δ* 167.2, 165.6, 157.0, 155.7, 155.3, 155.1, 150.4, 146.7, 129.9, 106.6, 106.4, 102.7, 101.1, 93.9, 67.5, 56.6, 56.1, 54.2, 52.9, 45.3, 33.2, 28.5, 25.7, 24.8, 23.5, 19.4. **ESI-MS** *m*/*z*: 580.2 [M + H]^+^.

*1-(2-(4,4-Difluoropiperidin-1-yl)-6-methoxy-7-(3-(pyrrolidin-1-yl)propoxy)quinazolin-4-yl)-3-((4-methoxy-6-methyl-2-oxo-1,2-dihydropyridin-3-yl)methyl)urea (**UA03**).* The crude product was purified by prep-HPLC (*Method 1* described in S5 in Supporting Material) to afford pure compound as pale yellow solid (62 mg; 8%). m.p. 143–145 °C. **^1^H NMR** (400 MHz, CDCl_3_) *δ* 12.73 (s, 1H), 9.47 (s, 1H), 8.91 (s, 1H), 7.38 (s, 1H), 6.83 (s, 1H), 5.90 (s, 1H), 4.50 (d, *J* = 3.1 Hz, 2H), 4.15 (t, *J* = 6.6 Hz, 2H), 4.01 (s, 3H), 3.83 (s, 3H), 3.72 (s, 4H), 2.66 (t, *J* = 7.2 Hz, 2H), 2.57 (s, 4H), 2.34 (s, 3H), 2.15–2.08 (m, 2H), 1.89–1.84 (m, 4H), 1.79 (s, 4H). **^13^C NMR** (101 MHz, CDCl_3_) *δ* 167.1, 165.6, 156.1, 156.0, 155.3, 155.2, 150.1, 147.2, 146.7, 122.3 (t, *J* = 240.3 Hz), 106.6, 106.5, 103.4, 101.4, 93.9, 67.3, 56.6, 56.1, 54.1, 52.9, 41.5, 33.7 (t, *J* = 22.7 Hz), 33.2, 29.7, 28.2, 23.5, 19.4. **ESI-MS** *m*/*z*: 616.2 [M + H]^+^.

*N-((4,6-Dimethyl-2-oxo-1,2-dihydropyridin-3-yl)methyl)-2-((6-methoxy-2-(piperidin-1-yl)-7-(3-(pyrrolidin-1-yl)propoxy)quinazolin-4-yl)amino)acetamide (**UB01**)* was synthesized using intermediate **17** and piperidine as starting materials according to **General procedure 1**. The crude product was purified by prep-HPLC (*Method 2* described in S5 in Supporting Material) to afford pure compound as white solid (77 mg; 32%). **m.p.** 137–140 °C. **^1^H NMR** (400 MHz, CDCl_3_): *δ* 12.29 (s, 1H), 7.57 (s, 1H), 7.01 (s, 1H), 6.90 (s, 1H), 6.84 (s, 1H), 5.82 (s, 1H), 4.34 (d, *J* = 5.1 Hz, 2H), 4.17 (d, *J* = 3.8 Hz, 2H), 4.06 (t, *J* = 6.4 Hz, 2H), 3.76 (s, 3H), 3.68 (s, 4H), 2.61 (t, *J* = 7.3 Hz, 2H), 2.53 (s, 4H), 2.27 (s, 3H), 2.07–2.04 (m, 5H), 1.77 (s, 4H), 1.57–1.56 (m, 2H), 1.53 (s, 4H). **^13^C NMR** (101 MHz, CDCl_3_) *δ* 170.1, 164.9, 158.7, 158.4, 153.9, 150.5, 149.1, 145.4, 143.0, 121.7, 109.7, 106.37, 103.0, 101.8, 67.2, 56.2, 54.1, 52.9, 45.0, 35.6, 28.4, 25.9, 25.0, 23.4, 19.5, 18.5. **ESI-MS**: 578.2 [M + H]^+^.

*N-((4,6-Dimethyl-2-oxo-1,2-dihydropyridin-3-yl)methyl)-2-((6-methoxy-2-(4-methyl-1,4-diazepan-1-yl)-7-(3-(pyrrolidin-1-yl)propoxy)quinazolin-4-yl)amino)acetamide (**UB02**)* was synthesized using intermediate **17** and N-methylhomopiperazine as starting materials according to **General procedure 1**. The crude product was purified by prep-HPLC (*Method 4* described in S5 in Supporting Material) to afford pure compound as white solid (99 mg; 39%). **m.p.** 139–141 °C. **^1^H NMR** (400 MHz, CDCl_3_): *δ* 12.37 (s, 1H), 7.55 (t, *J* = 5.3 Hz, 1H), 6.94 (s, 1H), 6.83 (s, 1H), 6.62 (brs, 1H), 5.83 (s, 1H), 4.35 (d, *J* = 5.6 Hz, 2H), 4.15 (d, *J* = 4.9 Hz, 2H), 4.09 (t, *J* = 6.7 Hz, 2H), 3.84 (brs, 2H), 3.76–3.73 (m, 5H), 2.65–2.64 (m, 2H), 2.60 (t, *J* = 7.4 Hz, 2H), 2.51 (brs, 6H), 2.32 (s, 3H), 2.29 (s, 3H), 2.10–2.03 (m, 5H), 1.94 (brs, 2H), 1.78–1.76 (m, 4H). **^13^C NMR** (101 MHz, CDCl_3_) *δ* 170.0, 164.9, 158.6, 158.1, 154.0, 150.2, 149.4, 145.3, 142.9, 121.8, 109.6, 106.5, 102.9, 58.9, 57.4, 56.3, 54.2, 52.9, 46.6, 46.0, 45.5, 45.2, 35.6, 28.5, 27.6, 23.5, 19.5, 18.5. **ESI-MS**: 607.3 [M + H]^+^.

*N-((4,6-Dimethyl-2-oxo-1,2-dihydropyridin-3-yl)methyl)-2-((2-(4,4-difluoropiperidin-1-yl)-6-methoxy-7-(3-(pyrrolidin-1-yl)propoxy)quinazolin-4-yl)amino)acetamide (**UB03**)* was synthesized using intermediate **17** and N-methylhomopiperazine 4,4-difluoropiperidine hydrochloride as starting materials according to **General procedure 1**. The crude product was purified by prep-HPLC (*Method 3* described in S5 in Supporting Material) to afford pure compound as white solid (67 mg; 26%). **m.p.** 136–139 °C. **^1^H NMR** (400 MHz, CDCl_3_) *δ* 11.66 (s, 1H), 7.52 (s, 1H), 6.85 (s, 1H), 5.87 (s, 1H), 4.35 (d, J = 5.1 Hz, 2H), 4.14–4.10 (m, 4H), 3.86 (s, 4H), 3.79 (s, 3H), 2.82–2.80 (m, 2H), 2.75 (s, 4H), 2.30 (s, 3H), 2.19–2.17 (m, 2H), 2.14 (s, 3H), 1.95–1.88 (m, 8H). **^13^C NMR** (101 MHz, CDCl_3_) *δ* 169.6, 164.6, 158.9, 157.5, 154.0, 150.2, 148.8, 146.0, 142.6, 122.0 (t, J = 300 Hz), 109.7, 106.5, 103.3, 101.5, 67.0, 56.3, 54.1, 52.9, 45.1, 41.0, 35.8, 33.9 (t, J = 23.6 Hz), 29.7, 23.5, 19.5, 18.7. **ESI-MS**: 614.2 [M + H]^+^.

*N-((4,6-Dimethyl-2-oxo-1,2-dihydropyridin-3-yl)methyl)-2-((2-(azepan-1-yl)-6-methoxy-7-(3-(pyrrolidin-1-yl)propoxy)quinazolin-4-yl)amino)acetamide (**UB04**)* was synthesized using intermediate **17** and hexamethyleneimine as starting materials according to **General procedure 1**. The crude product was purified by prep-HPLC (*Method 5* described in S5 in Supporting Material) to afford pure compound as white solid (72 mg; 29%). **m.p.** 127–129 °C. **^1^H NMR** (400 MHz, CDCl_3_) *δ* 12.12 (s, 1H), 7.69 (s, 1H), 7.10 (s, 1H), 5.81 (s, 1H), 4.32 (brs, 2H), 4.17 (s, 2H), 4.06 (brs, 2H), 3.81 (s, 3H), 3.68 (brs, 4H), 2.68–2.66 (m, 2H), 2.62 (s, 4H), 2.28 (s, 3H), 2.09–2.07 (m, 5H), 1.82 (brs, 4H), 1.74 (s, 4H), 1.48 (s, 4H). **^13^C NMR** (101 MHz, CDCl_3_) *δ* 169.8, 164.7, 158.7, 154.0, 150.2, 145.7, 142.8, 121.9, 109.4, 102.7, 67.1, 56.6, 54.1, 52.8, 47.5, 45.3, 35.6, 29.7, 28.1, 27.2, 23.5, 19.5, 18.6. **ESI-MS**: 592.2 [M + H]^+^.

*N-((4,6-dimethyl-2-oxo-1,2-dihydropyridin-3-yl)methyl)-2-((2-((dimethylamino)ethyl)(methyl)amino)-6-methoxy-7-(3-(pyrrolidin-1-yl)propoxy)quinazolin-4-yl)amino)acetamide (**UB05**)* was synthesized using intermediate **17** and N,N,N’-trimethylethylenediamine as starting materials according to **General procedure 1**. The crude product was purified by prep-HPLC (*Method 1* described in S5 in Supporting Material) to afford pure compound as white solid (50 mg; 20%). **m.p.** 135–136 °C. **^1^H NMR** (400 MHz, CDCl_3_) *δ* 12.29 (s, 1H), 7.87 (t, *J* = 5.1 Hz, 1H), 7.16 (brs, 1H), 7.06 (s, 1H), 6.81 (s, 1H), 5.82 (s, 1H), 4.32 (d, *J* = 5.5 Hz, 2H), 4.16 (d, *J* = 3.0 Hz, 2H), 4.05 (t, *J* = 6.6 Hz, 2H), 3.76 (s, 3H), 3.62 (t, *J* = 7.1 Hz, 2H), 3.09 (s, 3H), 2.63 (t, *J* = 7.4 Hz, 2H), 2.54 (s, 4H), 2.48 (t, *J* = 7.3 Hz, 2H), 2.28 (s, 3H), 2.27 (s, 6H), 2.10–2.03 (m, 5H), 1.78 (s, 4H). **^13^C NMR** (101 MHz, CDCl_3_) *δ* 170.2, 164.8, 158.7, 157.8, 153.9, 150.5, 148.1, 145.4, 143.0, 121.8, 109.6, 105.6, 102.9, 102.1, 67.1, 56.6, 56.3, 54.1, 52.9, 47.4, 45.5, 45.0, 35.6, 35.5, 28.3, 23.4, 19.5, 18.5. **ESI-MS**: 595.2 [M + H]^+^.

*N-((4,6-dimethyl-2-oxo-1,2-dihydropyridin-3-yl)methyl)-2-((2-((3-(dimethylamino)propyl)(methyl)amino)-6-methoxy-7-(3-(pyrrolidin-1-yl)propoxy)quinazolin-4-yl)amino)acetamide (**UB06**)* was synthesized using intermediate **17** and N,N,N’-trimethylpropane-1,3-diamine as starting materials according to **General procedure 1**. The crude product was purified by prep-HPLC (*Method 6* described in S5 in Supporting Material) to afford pure compound as white solid (57 mg; 22%). **m.p.** 131–133 °C. **^1^H NMR** (400 MHz, CDCl_3_) *δ* 7.72 (t, *J* = 5.6 Hz, 1H), 6.99 (s, 2H), 6.77 (s, 1H), 5.77 (s, 1H), 4.29 (d, *J* = 5.5 Hz, 2H), 4.11 (s, 2H), 4.01 (t, *J* = 6.6 Hz, 2H), 3.70 (s, 3H), 3.48 (t, *J* = 7.0 Hz, 2H), 3.01 (s, 3H), 2.55 (t, *J* = 7.4 Hz, 2H), 2.46 (s, 4H), 2.27–2.25 (m, 2H), 2.23 (s, 3H), 2.16 (s, 6H), 2.04–1.97 (m, 5H), 1.72–1.65 (m, 6H). **^13^C NMR** (101 MHz, CDCl_3_) *δ* 170.4, 164.8, 158.6, 158.4, 153.9, 150.4, 149.1, 145.1, 142.9, 121.8, 109.6, 106.0, 102.9, 102.0, 67.1, 57.0, 56.2, 54.1, 52.9, 47.4, 45.3, 45.1, 35.5, 35.1, 28.4, 25.8, 23.4, 19.5, 18.5. **ESI-MS**: 609.2 [M + H]^+^.

***General procedure 6**** for the Synthesis of **N1-N10, L1-L5**.* To a 25 mL round-bottomed flask were added **21** or **25** (0.08 mmol), corresponding amines (0.088 mmol), *p*-toluenesulfonic acid (0.027 g, 0.16 mmol), and *n*-butanol (3 mL). The flask was purged with argon three times and then heated in an oil bath at 110 °C. After 22.5 h, the reaction was complete. The solvent was removed under reduced pressure, and the residue was poured into water (20 mL). The aqueous layer was extracted with dichloromethane (20 mL × 4). The combined organic layers were washed with brine, dried over anhydrous magnesium sulfate, and concentrated to give crude product. This crude product was purified by column chromatography to afford the target compound.

*4,6-dimethyl-3-(((2-(4-methylpiperazin-1-yl)thieno[3,2-d]pyrimidin-4-yl)amino)methyl)pyridin-2(1H)-one (**N1**)* was synthesized using intermediate **21** and *N*-methylpiperazine as starting materials as white solid (58 mg; 36%). **m.p.** 251–254 °C. **^1^H NMR** (400 MHz, CDCl_3_), *δ* 12.45 (s, 1H), 7.49 (d, *J* = 5.3 Hz, 1H), 7.10 (d, *J* = 5.3 Hz, 1H), 6.24 (s, 1H), 5.95 (s, 1H), 4.76 (d, *J* = 5.7 Hz, 2H), 3.93 (s, 4H), 2.54 (s, 4H), 2.38 (s, 3H), 2.37 (s, 3H), 2.36 (s, 3H). **^13^C NMR** (101 MHz, CDCl_3_): *δ* 165.5, 161.6, 161.0, 157.1, 149.8, 142.8, 130.2, 124.2, 122.5, 109.7, 106.6, 55.1, 46.1, 44.2, 36.8, 19.6, 18.9. **ESI-MS** *m*/*z*: 385.2 [M + H]^+^.

*4,6-Dimethyl-3-(((2-(4-methyl-1,4-diazepan-1-yl)thieno[3,2-d]pyrimidin-4-yl)amino)methyl)pyridin-2(1H)-one (**N2**)* was synthesized using intermediate **21** and *N*-methylhomopiperazine as starting materials as white solid (40 mg; 24%). **m.p.** 153–155 °C. **^1^H NMR** (400 MHz, CDCl_3_), *δ* 13.11 (s, 1H), 7.46 (d, *J* = 5.3 Hz, 1H), 7.10 (d, *J* = 5.3 Hz, 1H), 6.26 (t, *J* = 5.5 Hz, 1H), 5.95 (s, 1H), 4.78 (d, *J* = 5.6 Hz, 2H), 4.03 (t, *J* = 4.6 Hz, 2H), 3.90 (t, *J* = 6.3 Hz, 2H), 2.74 (t, *J* = 4.6 Hz, 2H), 2.60 (t, *J* = 5.3 Hz, 2H), 2.39 (s, 3H), 2.36 (s, 3H), 2.34 (s, 3H), 2.07–2.01 (m, 2H). **^13^C NMR** (101 MHz, CDCl_3_): *δ* 165.7, 162.0, 160.9, 156.9, 150.0, 143.1, 129.8, 124.3, 122.4, 109.8, 105.6, 58.9, 57.5, 46.7, 46.4, 46.1, 36.6, 27.8, 19.6, 18.9. **ESI-MS** *m*/*z*: 399.2 [M + H]^+^.

*4,6-Dimethyl-3-(((2-morpholinothieno[3,2-d]pyrimidin-4-yl)amino)methyl)pyridin-2(1H)-one (**N3**)* was synthesized using intermediate **21** and morpholine as starting materials as white solid (46 mg; 30%). **m.p.** 97–100 °C. **^1^H NMR** (400 MHz, CDCl_3_), *δ* 12.82 (s, 1H), 7.51 (d, *J* = 5.3 Hz, 1H), 7.15 (d, *J* = 5.3 Hz, 1H), 6.37 (s, 1H), 5.97 (s, 1H), 4.77 (d, *J* = 5.5 Hz, 2H), 3.86–3.80 (m, 8H), 2.35 (s, 6H). **^13^C NMR** (101 MHz, CDCl_3_): δ 165.6, 157.1, 150.1, 143.2, 135.0, 130.4, 124.1, 122.2, 116.7, 109.9, 106.9, 67.0, 45.1, 36.9, 19.6, 18.9. **ESI-MS** *m*/*z*: 372.1 [M + H]^+^.

*3-(((2-(4,4-Difluoropiperidin-1-yl)thieno[3,2-d]pyrimidin-4-yl)amino)methyl)-4,6-dimethylpyridin-2(1H)-one (**N4**)* was synthesized using intermediate **21** and *4,4-difluoropiperidine hydrochloride* as starting materials as white solid (50 mg; 29%). **m.p.** 198–200 °C. **^1^H NMR** (400 MHz, CDCl_3_), *δ* 12.80 (s, 1H), 7.51 (d, *J* = 5.3 Hz, 1H), 7.15 (s, 1H), 6.42 (s, 1H), 5.97 (s, 1H), 4.04 (t, *J* = 5.2 Hz, 4H), 2.36 (s, 6H), 2.07–1.97 (m, 4H). **^13^C NMR** (101 MHz, CDCl_3_): *δ* 165.6, 161.3, 157.2, 150.0, 143.1, 130.8, 130.5, 124.5 122.7 (t, *J* = 245 Hz), 109.8, 106.8, 66.9, 41.6, 36.9, 33.9 (t, *J* = 22.5 Hz), 19.6, 18.9. **ESI-MS** *m*/*z*: 406.2 [M + H]^+^.

*3-(((2-((3-(Dimethylamino)propyl)(methyl)amino)thieno[3,2-d]pyrimidin-4-yl)amino)methyl)-4,6-dimethylpyridin-2(1H)-one (**N5**)* was synthesized using intermediate **21** and *N,N,N’-Trimethylpropane-1,3-diamine* as starting materials as white solid (55 mg; 33%). **m.p.** 161–163 °C. **^1^H NMR** (400 MHz, CDCl_3_), *δ* 13.13 (s, 1H), 7.45 (d, *J* = 5.3 Hz, 1H), 7.10 (d, *J* = 5.3 Hz, 1H), 6.27 (t, *J* = 5.4 Hz, 1H), 5.96 (s, 1H), 4.79 (d, *J* = 5.6 Hz, 2H), 3.75 (t, *J* = 7.2 Hz, 2H), 3.21 (s, 3H), 2.38–2.34 (m, 8H), 2.26 (s, 6H), 1.89–1.82 (m, 2H). **^13^C NMR** (101 MHz, CDCl_3_): *δ* 165.7, 161.9, 161.2, 156.9, 150.0, 143.1, 129.7, 124.3, 122.4, 109.8, 105.5, 57.4, 47.8, 45.5, 36.6, 35.6, 25.9, 19.7, 18.9. **ESI-MS** *m*/*z*: 401.3 [M + H]^+^.

*3-(((2-(Azepan-1-yl)thieno[3,2-d]pyrimidin-4-yl)amino)methyl)-4,6-dimethylpyridin-2(1H)-one (**N6**)* was synthesized using intermediate **21** and hexamethyleneimine as starting materials as white solid (65 mg; 40%). **m.p.** 113–115 °C. **^1^H NMR** (400 MHz, CDCl_3_), *δ* 12.94 (s, 1H), 7.45 (d, *J* = 5.3 Hz, 1H), 7.15 (d, *J* = 4.6 Hz, 1H), 6.27 (s, 1H), 5.96 (s, 1H), 4.79 (d, *J* = 5.6 Hz, 2H), 3.83 (t, *J* = 5.9 Hz, 3H), 2.35 (s, 6H), 1.84–1.82 (m, 4H), 1.58–1.56 (m, 4H). **^13^C NMR** (101 MHz, CDCl_3_): *δ* 165.7, 156.9, 150.1, 143.0, 130.8, 129.7, 124.4, 124.2, 122.4, 109.8, 105.4, 47.3, 36.6, 28.4, 27.5, 19.6, 18.9. **ESI-MS** *m*/*z*: 384.2 [M + H]^+^.

*3-(((2-((2-(Dimethylamino)ethyl)(methyl)amino)thieno[3,2-d]pyrimidin-4-yl)amino)methyl)-4,6-dimethylpyridin-2(1H)-one (**N7**)* was synthesized using intermediate **21** and *N,N,N’*-Trimethylethylenediamine as starting materials as white solid (45 mg; 28%). **m.p.** 206–207 °C. **^1^H NMR** (400 MHz, CDCl_3_), *δ* 12.99 (s, 1H), 7.47 (d, *J* = 5.3 Hz, 1H), 7.10 (d, *J* = 5.3 Hz, 1H), 6.25 (s, 1H), 5.96 (s, 1H), 4.79 (d, *J* = 5.4 Hz, 2H), 3.85 (t, *J* = 7.3 Hz, 2H), 3.23 (s, 3H), 2.61 (t, *J* = 7.2 Hz, 2H), 2.35–2.34 (m, 12H). **^13^C NMR** (101 MHz, CDCl_3_): *δ* 165.6, 161.9, 161.1, 157.0, 150.1, 143.1, 129.8, 124.3, 122.3, 109.8, 105.7, 56.8, 47.7, 45.8, 36.7, 35.9, 19.7, 18.9. **ESI-MS** *m*/*z*: 387.2 [M + H]^+^.

*4,6-Dimethyl-3-(((2-(pyrrolidin-1-yl)thieno[3,2-d]pyrimidin-4-yl)amino)methyl)pyridin-2(1H)-one (**N8**)* was synthesized using intermediate **21** and pyrrolidine as starting materials as white solid (59 mg; 40%). **m.p.** 145–147 °C. **^1^H NMR** (400 MHz, CDCl_3_), *δ* 12.04 (s, 1H), 7.46 (d, *J* = 5.3 Hz, 1H), 7.18 (s, 1H), 6.24 (s, 1H), 5.94 (s, 1H), 4.78 (d, *J* = 5.8 Hz, 2H), 3.66 (t, *J* = 6.5 Hz, 4H), 2.40 (s, 3H), 2.34 (s, 3H), 2.00–1.96 (m, 4H). **^13^C NMR** (101 MHz, CDCl_3_): *δ* 165.4, 156.9, 149.7, 142.5, 129.7, 124.2, 122.9, 109.6, 105.7, 46.9, 36.6, 25.6, 19.7, 18.9. **ESI-MS** *m*/*z*: 356.1 [M + H]^+^.

*3-(((2-(4-Methoxypiperidin-1-yl)thieno[3,2-d]pyrimidin-4-yl)amino)methyl)-4,6-dimethylpyridin-2(1H)-one (**N9**)* was synthesized using intermediate **21** and 4-methoxypiperidine as starting materials as white solid (71 mg; 42%). **m.p.** 153–155 °C. **^1^H NMR** (400 MHz, CDCl_3_), *δ* 12.89 (s, 1H), 7.48 (d, *J* = 5.3 Hz, 1H), 7.12 (s, 1H), 6.30 (s, 1H), 5.94 (s, 1H), 4.77 (d, *J* = 5.7 Hz, 2H), 4.48–4.43 (m, 2H), 3.51–3.42 (m, 1H), 3.40 (s, 3H), 3.38–3.34 (m, 2H), 2.36 (s, 3H), 2.34 (s, 3H), 2.00–1.96 (m, 2H), 1.63–1.54 (m, 2H). **^13^C NMR** (101 MHz, CDCl_3_): *δ* 165.7, 157.1, 149.9, 143.0, 130.1, 124.1, 122.5, 109.8, 106.1, 55.6, 42.2, 36.8, 30.9, 19.6, 18.9. **ESI-MS** *m*/*z*: 400.1 [M + H]^+^.

*4,6-Dimethyl-3-(((2-((2-(piperidin-1-yl)ethyl)amino)thieno[3,2-d]pyrimidin-4-yl)amino)methyl)pyridin-2(1H)-one (**N10**)* was synthesized using intermediate **21** and 1-(2-aminoethyl)piperidine as starting materials as white solid (66 mg; 38%). **m.p.** 127–129 °C. **^1^H NMR** (400 MHz, CDCl_3_), *δ* 12.81 (s, 1H), 7.47 (d, *J* = 5.3 Hz, 1H), 7.08 (d, *J* = 5.3 Hz, 1H), 6.31 (t, *J* = 5.5 Hz, 1H), 5.95 (s, 1H), 5.40 (s, 1H), 4.73 (d, *J* = 5.7 Hz, 2H), 3.56 (t, *J* = 5.6 Hz, 2H), 2.62–2.59 (m, 2H), 2.47 (brs, 4H), 2.42 (s, 3H), 2.35 (s, 3H),1.62–1.60 (m, 4H), 1.47–1.43 (m, 2H). **^13^C NMR** (101 MHz, CDCl_3_): *δ* 165.6, 161.4, 161.4, 157.4, 150.1, 142.9, 130.0, 124.0, 122.4, 109.8, 106.8, 57.8, 54.4, 38.6, 37.0, 25.9, 24.4, 19.7, 18.9. **ESI-MS** *m*/*z*: 413.2 [M + H]^+^.

*4,6-Dimethyl-3-(((2-(4-methylpiperazin-1-yl)thieno[2,3-d]pyrimidin-4-yl)amino)methyl)pyridin-2(1H)-one (**L1**)* was synthesized using intermediate **25** and *N*-methylpiperazine as starting materials as white solid (48 mg; 30%). **m.p.** 221–223 °C. **^1^H NMR** (400 MHz, CDCl_3_), *δ* 11.53 (s, 1H), 6.91 (d, *J* = 6.0 Hz, 1H), 6.75 (d, *J* = 5.9 Hz, 1H), 6.44 (d, *J* = 5.3 Hz, 1H), 5.93 (s, 1H), 4.73 (d, *J* = 5.7 Hz, 2H), 3.97 (s, 4H), 2.59 (s, 4H), 2.42 (s, 3H), 2.35 (s, 3H), 2.29 (s, 3H). **^13^C NMR** (101 MHz, CDCl_3_): *δ* 169.2, 165.2, 159.4, 157.0, 149.9, 142.3, 122.9, 117.2, 116.4, 109.7, 109.5, 54.9, 45.9, 43.8, 36.6, 19.7, 18.9. **ESI-MS** *m*/*z*: 385.2 [M + H]^+^.

*3-(((2-((3-(Dimethylamino)propyl)(methyl)amino)thieno[2,3-d]pyrimidin-4-yl)amino)methyl)-4,6-dimethylpyridin-2(1H)-one (**L2**)* was synthesized using intermediate **25** and *N,N,N’*-trimethylpropane-1,3-diamine as starting materials as white solid (63 mg; 37%). **m.p.** 146–148 °C. **^1^H NMR** (400 MHz, CDCl_3_), *δ* 12.43 (s, 1H), 6.93 (d, *J* = 6.0 Hz, 1H), 6.68 (d, *J* = 5.9 Hz, 1H), 6.46 (t, *J* = 5.4 Hz, 1H), 5.93 (s, 1H), 4.76 (d, *J* = 5.6 Hz, 2H), 3.75–3.71 (t, *J* = 7.2 Hz, 2H), 3.60 (q, *J* = 7.3 Hz, 2H), 3.20 (s, 3H), 2.40–2.36 (m, 2H), 2.34 (s, 3H), 2.27 (s, 9H). **^13^C NMR** (101 MHz, CDCl_3_): *δ* 169.5, 165.4, 159.9, 156.9, 150.2, 142.8, 122.7, 117.4, 115.2, 109.8, 108.6, 57.3, 47.6, 45.4, 36.3, 35.6, 25.8, 19.7, 18.8. **ESI-MS** *m*/*z*: 401.2 [M + H]^+^.

*3-(((2-(Azepan-1-yl)thieno[2,3-d]pyrimidin-4-yl)amino)methyl)-4,6-dimethylpyridin-2(1H)-one (**L3**)* was synthesized using intermediate **25** and hexamethyleneimine as starting materials as white solid (57 mg; 35%). **m.p.** 152–153 °C. **^1^H NMR** (400 MHz, CDCl_3_), *δ* 11.09 (s, 1H), 6.92 (s, 1H), 6.77 (s, 1H), 5.95 (s, 1H), 4.74 (d, *J* = 4.1 Hz, 2H), 3.85 (s, 4H), 2.35 (s, 3H), 2.29 (s, 3H), 1.84 (s, 4H), 1.58 (s, 4H). **ESI-MS** *m*/*z*: 384.2 [M + H]^+^.

*3-(((2-((2-(Dimethylamino)ethyl)(methyl)amino)thieno[2,3-d]pyrimidin-4-yl)amino)methyl)-4,6-dimethylpyridin-2(1H)-one (**L4**)* was synthesized using intermediate **25** and N,N,N’-trimethylethylenediamine as starting materials as white solid (55 mg; 34%). **m.p.** 167–169 °C. **^1^H NMR** (400 MHz, CDCl_3_), *δ* 11.97 (s, 1H), 6.91 (d, *J* = 4.0 Hz, 1H), 6.70 (d, *J* = 4.0 Hz, 1H), 6.37 (s, 1H), 5.94 (s, 1H), 4.75 (d, *J* = 3.7 Hz, 2H), 3.85 (t, *J* = 4.8 Hz, 2H), 3.22 (s, 3H), 2.63 (s, 2H), 2.38 (s, 5H), 2.34 (s, 3H), 2.29 (s, 3H). **^13^C NMR** (101 MHz, CDCl_3_): *δ* 169.5, 165.3, 159.8, 157.0, 150.1, 142.5, 122.7, 117.2, 115.5, 109.7, 108.8, 77.3, 77.0, 76.7, 56.8, 47.4, 45.6, 36.5, 35.8, 19.7, 18.9. **ESI-MS** *m*/*z*: 387.2 [M + H]^+^.

*4,6-Dimethyl-3-(((2-((2-(piperidin-1-yl)ethyl)amino)thieno[2,3-d]pyrimidin-4-yl)amino)methyl)pyridin-2(1H)-one (**L5**)* was synthesized using intermediate **25** and 1-(2-aminoethyl)piperidine as starting materials as white solid (76 mg; 44%). **m.p.** 198–200 °C. **^1^H NMR** (400 MHz, CDCl_3_), *δ* 11.41 (s, 1H), 6.92 (d, J = 6.0 Hz, 1H), 6.73 (d, J = 6.0 Hz, 1H), 6.46 (s, 1H), 5.92 (s, 1H), 5.48 (s, 1H), 4.69 (d, J = 5.7 Hz, 2H), 3.60 (s, 2H), 2.64 (s, 2H), 2.52 (s, 2H), 2.42 (s, 3H), 2.28 (s, 3H), 1.65 (s, 6H), 1.48 (s, 2H). **^13^C NMR** (101 MHz, CDCl_3_): *δ* 169.1, 165.1, 160.0, 157.3, 150.2, 142.2, 123.0, 117.4, 115.8, 109.7, 108.4, 57.7, 54.4, 47.9, 47.8, 38.2, 36.8, 19.8, 19.0. **ESI-MS** *m*/*z*: 413.2 [M + H]^+^.

### 3.2. In Vitro HMT Assay

In vitro methyltransferase assays were performed using an MTase-Glo Kit (Promega, catalog no. V7601) according to the manufacturer’s instructions [34]. Compounds were incubated with 0.5 ng/well recombinant EHMT2 (G9a) protein (Active motif, catalog no. 31410) or recombinant PRC2 complex (Active motif, catalog no. 31387), 30 ng/well recombinant human histone H3 (Abcam, catalog no. ab198757), and 2 μmol/L S-adenosyl methionine (SAM) in the reaction buffer (50 mmol/L Tris-HCl, pH 8.1, 5 mmol/L NaCl, 5 mmol/L MgCl_2_, 1 mmol/L DTT, 1 mmol/L PMSF, and 1% DMSO) for 1 h at room temperature. After stopping the reaction, the luminescence readout was measured using a microplate luminescence detector (Centro xs^3^ LB 960, Berthold Technologies, Bad Wildbad, Gemany). Data analysis was performed using GraphPad Prism 10.5.0 software, and the IC_50_ values were calculated using the “log (inhibitor) vs. response—Variable slope (four parameters)” nonlinear regression model, with three independent replicates (*n* = 3).

### 3.3. Western Blot Analysis

RD cells were incubated with target compounds (5 μM) or 0.1% DMSO for 6 days. Cell samples were collected in ice-cold lysis buffer. Lysates were cleared by centrifugation at 12,000 rpm for 5 min at 4 °C, and supernatants were removed and assayed for protein concentration using the BCA Protein Assay Kit (Thermo Fisher Scientific, Waltham, MA, USA). Cell lysates were loaded to 10–15% SDS-PAGE and separated by electrophoresis. Separated proteins were then electrically transferred to polyvinylidene difluoride membranes (Millipore IPVH00010), which were blocked with 5% bovine serum albumin/TBST for 1 h. Membranes were hybridized with the following primary antibodies: Anti-H3K9me2 antibody (Cell Signalling Technology), Anti-H3K27me3 antibody (Cell Signalling Technology).

### 3.4. In Vitro Antiproliferative Activity Evaluation

**Cell Culture.** RD cells were obtained from China Center for Type Culture Collection (CCTCC), cultured in DMEM (Dulbecco’s modified Eagle’s medium, Gbico) with 10% FBS and 1% penicillin-streptomycin (Gibco). All the cells were maintained in a humidified atmosphere of 5% CO_2_ at 37 °C.

**MTT Assay** [35]. RD cells (3 × 10^3^/well) were seeded in 96-well plates and then treated with vehicle alone or with the tested compounds for 72 h. Then, 10 μL of MTT (5 mg/mL, in PBS) was added to each well, and the samples were incubated for a further 4 h. The MTT formazan formed by viable cells was dissolved in DMSO (100 μL), and the absorbance was measured using a multi-mode plate reader (Molecular Devices, San Jose, CA, USA) (492 nm).

### 3.5. Molecular Modeling

The X-ray crystal structures of G9a (PDB ID: 5VSC) and EZH2 (PDB ID: 4W2R) were retrieved from the Protein Data Bank [36]. The protein structures were prepared using the Protein Preparation Wizard module. Hydrogen atoms were added, water molecules in the entire system were removed, followed by energy minimization and optimization using the OPLS_2005 force field. Grids of G9a and EZH2 were generated using Glide 2016-2, following the standard procedure recommended by Schrödinger software. The Ligand Preparation Wizard module was employed to prepare the compounds for molecular docking. To prepare ligand structures, hydrogens were added, and 3D geometries, ionization, and tautomeric states were generated. Finally, the ligand structures were minimized using the OPLS 2005 force field [37]. The conformational ensembles were docked flexibly using Glide with the standard settings in both the standard-precision modes. Only poses with low-energy conformations and good hydrogen-bond geometries were considered.

### 3.6. Molecular Dynamics Simulation

Molecular dynamics (MD) simulations were performed using GROMACS 2022.2 [38,39,40]. The force field parameters for the proteins were generated using the pdb2gmx tool within GROMACS 2022.2. For the ligands, topology files were generated using the GAFF2 force field [41] via sobtop_1.0 (dev3.1), and partial atomic charges were assigned using the RESP method to ensure physicochemical consistency. The AMBER14SB force field [42] was applied to the receptor proteins. During the simulations, systems were solvated with the TIP3P water model, maintaining a minimum distance of 1.0 nm between the protein atoms and the box edges to ensure adequate solvation. To achieve system electroneutrality, Na^+^ and Cl^−^ ions were added at a concentration of 0.15 M NaCl using the gmx genion tool of GROMACS 2022.2, mimicking physiological ionic strength. Long-range electrostatic interactions were treated with the Particle Mesh Ewald (PME) method using a cutoff distance of 1.0 nm. Bond constraints were handled using the LINCS algorithm.

Prior to the production runs, the systems underwent energy minimization: first with 3000 steps of the steepest descent algorithm, followed by 2000 steps of conjugate gradient minimization. Simulations were performed under the NPT ensemble at 310 K using a Nosé–Hoover thermostat with a coupling constant of 0.1 ps, and at 1 bar using a Parrinello–Rahman barostat with a coupling constant of 2.0 ps. Each simulation was run for 100 ns with an integration time step of 2 fs. The root-mean-square deviation (RMSD), root-mean-square fluctuation (RMSF), number of hydrogen bonds, radius of gyration (Rg), solvent-accessible surface area (SASA), and free energy landscape were calculated using the gmx rmsd, gmx rmsf, gmx hbond, gmx gyrate, gmx sasa, and gmx sham tools in GROMACS 2022.2, respectively, to assess system stability, conformational changes, and solvent effects.

### 3.7. Pharmacokinetics Study In Vivo

SD rats (6 females, 6 males, and 7 weeks old, 180–200 g) were used in this study and purchased from Liaoning Changsheng Biotechnology Co., Ltd. (Benxi, Liaoning, China) (License No.: SCXK (Liao) 2020-0001). The SD rats were raised under sterile conditions (18–22 °C, humidity 35%−65%, and ventilation) under daily cycles of light/darkness with a 12 h interval. They went through a period of 3 days of acclimatization before experimental procedure. 12 SD rats were assigned to 2 groups randomly. **SU101** formulated in 0.5% CMC-Na solution was administered orally at the indicated doses. The combination of **SU101** in a ratio of 5% DMSO, 5% Tween-80 and 90% normal saline was chosen for the intravenous injection formulation. The SD rats were administered compound **SU101** *via* iv or *po* routes with different doses of 2.5 and 6.25 mg/kg. SD rats were weighed to calculate the required volume before each administration. Blood samples were obtained at indicated time points (*iv*: 0, 0.083, 0.167, 0.25, 0.5, 1, 2, 4, 8, 12, 24 h; *po*: 0, 0.083, 0.167, 0.25, 0.5, 1, 2, 4, 8, 12, 24 h). Blood samples were taken from the orbital vein at indicated time points, collected in Eppendorf tubes containing anticoagulant EDTA-K_2_, and centrifuged at 4500 rpm for 5 min. After that, each plasma sample (100 μL) was mixed with internal standard **SU08** in MeCN:H_2_O (3:7, *v*/*v*) (2 μg/mL, 50 μL) and EA (500 μL). The resulting mixture was vortexed for 2 min, then centrifuged at 4000 rpm for 5 min. Then, supernatant was reconstituted with MeCN:H_2_O (3:7, *v*/*v*) (100 μL), and resulting concentrations of compounds were respectively analyzed by HPLC (Schimadzu LC-20A with a Shimadzu Inertsil-SP C18 (5 µm; 150 mm × 4.6 mm)). All the pharmacokinetic parameters were calculated using the non-compartmental analysis (NCA) model with Phoenix WinNonlin 8.1.0 software.

### 3.8. Percentage Plasma Protein Binding (%PPB)

Kunming mice (5 males, 20–22 g) were purchased from Liaoning Changsheng Biotechnology Co., Ltd. (Liaoning, China) (License No.: SCXK (Liao) 2020-0001). dialysis bag was purchased from viskase (MD3428). Orbital blood collection was performed to collect blood samples in Eppendorf tubes containing anticoagulant EDTA-K_2_, then centrifuged at 4500 rpm for 5 min to get blood plasma samples. PBS solution in external dialysate: Na_2_HPO_4_ 6 g, NaH_2_PO_4_ 0.4 g, NaCl 9 g in distilled water 1 L (pH = 7.4, 0.02 mol/L). Three 25 mL beakers contained 15 mL of 5 μg/mL, 1 μg/mL, and 0.2 μg/mL **SU101** in external dialysate, respectively. Three dialysis bags, each containing 300 μL of blank mouse plasma, were clamped, and the beakers were sealed with ziplock bags. The beakers were equilibrated in a 37 °C water bath for 24 h. Concentrations of external dialysate and internal dialysate were analyzed by HPLC (Schimadzu LC-20A with a Shimadzu Inertsil-SP C18 (5 µm; 150 mm × 4.6 mm)). The %PPB value of the compound was calculated by following equation: % PPB = 100 × ([Donor]_24 h_ − [Receiver]_24 h_)/[Donor]_24 h_. Data are expressed as the mean from three concentrations.

### 3.9. Acute Toxicity Study

Kunming mice (6 males, 20–22 g) were purchased from Liaoning Changsheng Biotechnology Co., Ltd. (Liaoning, China) (License No.: SCXK (Liao) 2020-0001). **SU101** was administered by intraperitoneal administration. Mice were dosed sequentially according to the table (S6 in Supporting Information) and observed for 14 days.

## 4. Conclusions

In summary, a series of 43 novel dual G9a/EZH2 inhibitors belonging to five structural series (**SU**, **UA**, **UB**, **N**, and **L**) were designed and synthesized based on the lead compound SU08. Structure–activity relationship studies revealed that the quinazoline core is essential for maintaining potent dual inhibition, while urea modifications enhanced EZH2 activity but reduced G9a potency, and thienopyrimidine bioisosteres led to diminished activity. Among all compounds, **SU101** emerged as the most promising, with low nanomolar enzymatic inhibitory activity, robust cellular target engagement, and superior antiproliferative efficacy against RD cells. Molecular modeling and MD simulations corroborated its favourable binding modes with both EZH2 and G9a. Moreover, **SU101** exhibited excellent oral bioavailability, moderate plasma protein binding, and manageable acute toxicity, indicating favourable druggability. These results highlight **SU101** as a potential therapeutic agent for rhabdomyosarcoma and offer valuable insights for the rational design of next-generation dual epigenetic inhibitors targeting G9a and EZH2.

## Figures and Tables

**Table 1:** Inhibitory effects of compounds against G9a and EZH2.

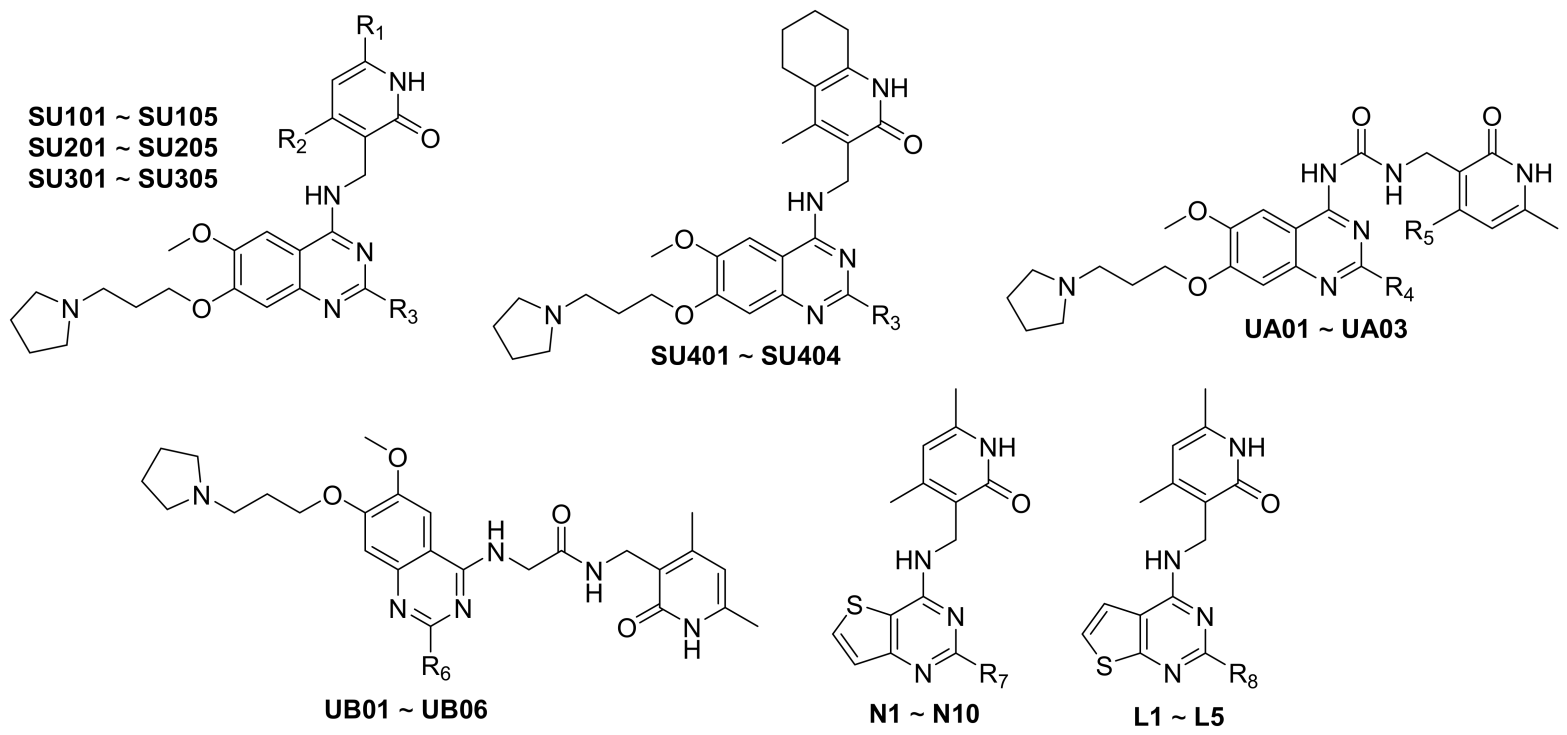			
Compound	% Inhibition (@5 μM)		cLogP
	EZH2	G9a	
GSK126	97.65	-	2.279
Tazemetostat	98.10	-	3.506
UNC0638	-	99.32	5.284
N1	75.62	86.24	2.679
N2	79.11	85.17	2.742
N3	80.67	82.36	2.41
N4	71.49	85.14	3.544
N5	76.31	83.35	2.923
N6	78.62	81.60	4.096
N7	80.58	80.49	2.861
N8	88.14	79.39	3.183
N9	79.25	76.58	3.639
N10	74.38	81.04	3.425
L1	76.24	77.16	2.45
L2	80.15	76.24	2.512
L3	81.02	78.05	2.694
L4	89.95	74.62	3.866
L5	76.53	77.50	2.631
SU101	95.92	93.18	3.557
SU102	94.15	90.94	2.659
SU103	95.69	92.85	3.461
SU104	91.52	92.37	4.013
SU105	93.71	91.56	2.778
SU201	94.52	89.95	5.295
SU202	92.55	91.64	4.398
SU203	95.03	91.08	5.199
SU204	91.58	93.97	5.751
SU205	93.24	89.51	4.517
SU301	95.48	90.54	4.839
SU302	90.15	89.69	3.941
SU303	93.66	90.31	4.743
SU304	92.10	93.74	5.295
SU305	93.47	91.36	4.06
SU401	91.03	92.16	5.37
SU402	90.87	90.46	4.472
SU403	92.11	90.80	5.826
SU404	90.09	91.43	4.591
UA01	95.97	86.32	3.623
UA02	92.00	88.95	2.864
UA03	93.35	85.73	2.768
UB01	87.50	86.52	3.375
UB02	85.33	83.55	2.477
UB03	81.59	82.96	3.279
UB04	86.42	84.72	3.831
UB05	87.91	81.28	2.597
UB06	80.54	83.19	2.659

**Table 2:** In Vitro EZH2 and G9a Inhibitory Activities of Compounds ^a^.

Compound	IC_50_ ± SD (nM)	
	EZH2	G9a
UNC0638	-	3.88 ± 0.34
Tazemetostat	2.76 ± 0.25	-
SU08	4.49 ± 0.33	3.01 ± 0.31
SU101	4.08 ± 0.21	2.92 ± 0.21
SU103	4.20 ± 0.39	3.52 ± 0.32
SU104	4.68 ± 0.32	4.12 ± 0.41
UA01	4.27 ± 0.49	17.79 ± 1.66
UB01	25.37 ± 1.95	15.76 ± 1.60

^a^ IC_50_ values for enzymatic inhibition of EZH2 and G9a; data are expressed as the mean ± SD from the dose–response curves of three independent experiments.

**Table 3:** Antiproliferative activities of selected compounds against RD cell line ^a^.

Compound	Structure	IC_50_ ± SD (μM)
Tazemetostat	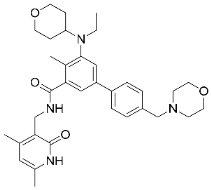	191.98 ± 1.02
UNC0638	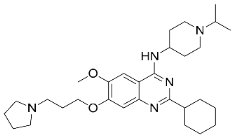	23.76 ± 0.53
SU08	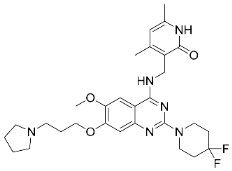	20.72 ± 0.64
SU101	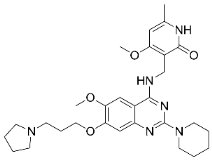	15.33 ± 0.31
SU103	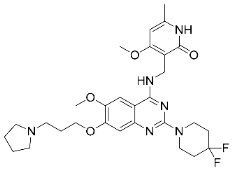	38.25 ± 0.72
SU104	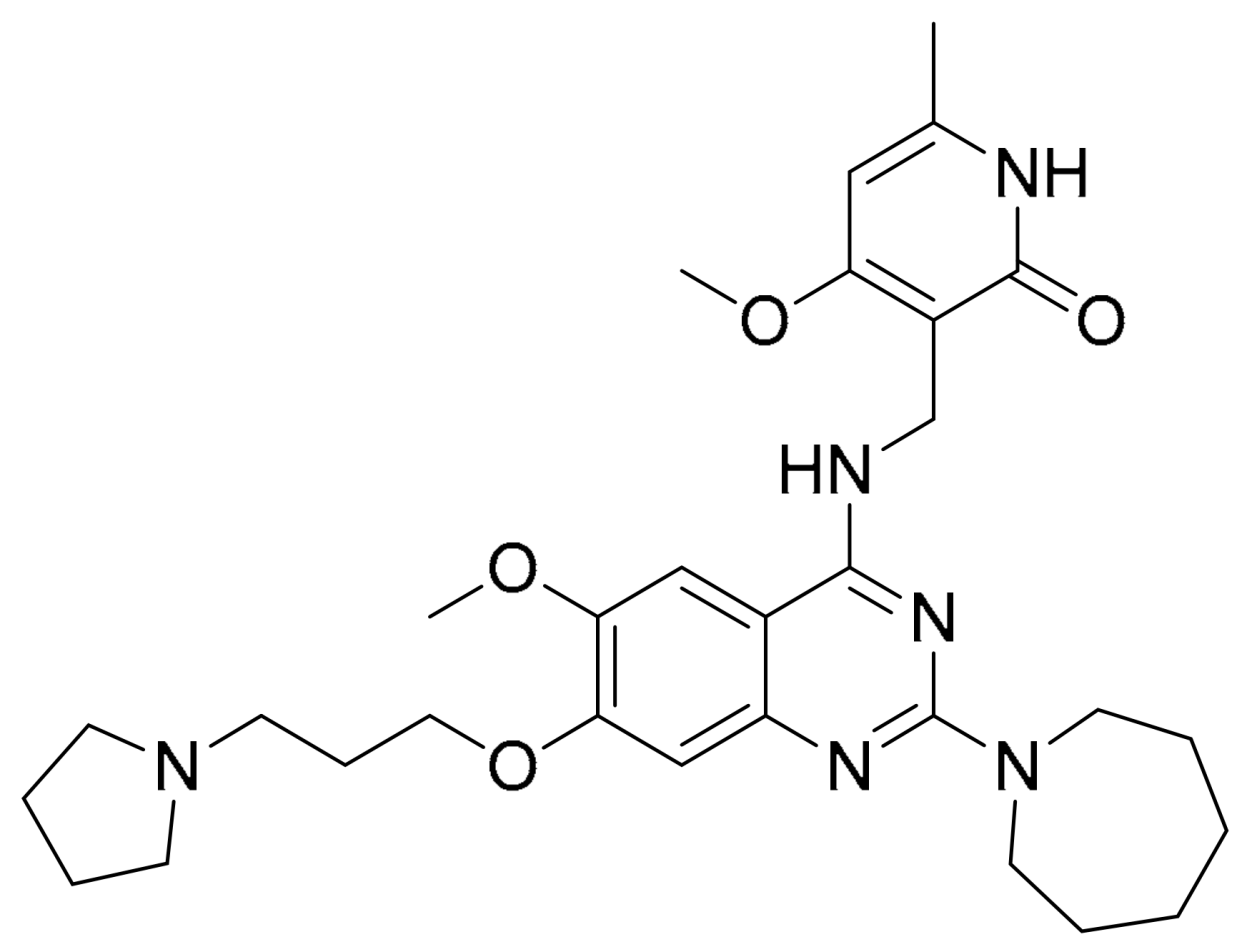	88.35 ± 1.30
UA01	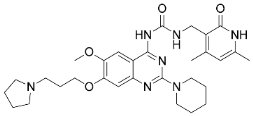	45.15 ± 0.59
UA02	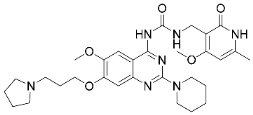	85.03 ± 0.66
UA03	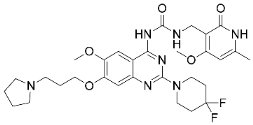	39.92 ± 0.48
UB02	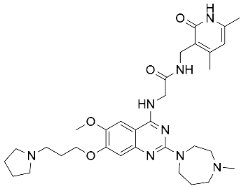	146.47 ± 0.95

^a^ Cell viability was determined by the MTT assay after treatment for 72 h.

**Table 4:** Pharmacokinetics of **SU101** in Rats following Intravenous and Oral Administration ^a^.

Route	T_max_ (h)	C_max_ (ng/mL)	t_1/2_ (h)	CL (mL/h/kg)	V_z_ (mL/kg)	AUC_(0–24)_ (ng·h/mL)	AUC_(0–∞)_ (ng·h/mL)	*F* (%)
*iv* (2.5 mg/kg)	0.083 ± 0.000	2828.869 ± 385.656	0.24 ± 0.03	2831.97 ± 202.35	989.16 ± 161.08	857.47 ± 65.98	886.76 ± 66.99	
*po* (6.25 mg/kg)	0.167 ± 0.000	1406.645 ± 82.788	0.94 ± 0.08	3128.09 ± 247.38	4254.01 ± 445.29	2021.51 ± 163.49	2129.60 ± 164.41	94.3

^a^ Species: SD rats; After administration, blood samples were collected at different time (*iv*: 0, 0.083, 0.167, 0.25, 0.5, 1, 2, 4, 8, 12, 24 h; *po*: 0, 0.083, 0.167, 0.25, 0.5, 1, 2, 4, 8, 12, 24 h); Compounds formulated in 0.5% CMC-Na solution was administered orally at the indicated doses; The combination of compounds in a ratio of 5% DMSO, 5% Tween-80 and 90% normal saline was chosen for the intravenous injection formulation. F (%) calculated as [AUC_(0–24)__po/Dose_po]/[AUC_(0–24)__iv/Dose_iv] × 100.

## Data Availability

The data presented in this study are available within the article and Supplementary Materials. Additional data are available from the corresponding authors upon reasonable request.

## Supplementary Materials

The following supporting information can be downloaded at https://www.mdpi.com/article/10.3390/molecules31193446/s1: Figure S1. Effects of compounds (5 μM) on H3K27me3 and H3K9me2 protein expressions in RD cells; Figure S2. Antiproliferative Activities of compounds against RD cell line; Figure S3. HMT assays of compounds against EZH2/G9a; S4. Synthesis procedures; S5. Protocols for preparative HPLC purification methods; S6. Acute toxicity test of **SU101**; S7. NMR spectra of compounds; S8. MS Spectra of compounds.
