# Supplementary material for: Design, Synthesis and Antitumor Activity Evaluation of Novel Dual G9a/EZH2 Inhibitors
Source: Molecules. 2026 Sep 27;31(19):3446. doi: 10.3390/molecules31193446 (PMC13666074; doi:10.3390/molecules31193446)
Supplement: Supplementary file 1 [file molecules-31-03446-s001.zip › molecules-4582524-supplementary.pdf]

Supplementary Materials

Design, Synthesis and Antitumor Activity Evaluation of Novel Dual G9a/EZH2 Inhibitors

Yajie Shi[1,*], Qiuyue Zhang[2,4], Maoying Zhang[3], Xiujuan Zhao[1], Shuhang Zhang[1], Jirui Li[1], Haofeng Li[1], Chenglin Fu[1], Guoliang Chen[3,*]

[1]*College of pharmacy, Heze University, Heze 274000, China*

[2]*Department of Pharmacology, Shenyang Pharmaceutical University, Shenyang 110016, China*

[3]*Key Laboratory of Structure-Based Drug Design & Discovery of Ministry of Education, School of Pharmaceutical Engineering, Shenyang Pharmaceutical University, Shenyang 110016, China*

[4]*College of pharmacy, Jilin Medical University, Jilin 132013, China.*

* Corresponding authors at: College of pharmacy, Heze University, Heze 274000, PR China; Key Laboratory of Structure-Based Drug Design & Discovery of Ministry of Education, School of Pharmaceutical Engineering, Shenyang Pharmaceutical University, Shenyang 110016, PR China

E-mail addresses: shiyajie1002@163.com (Y. Shi), chenguoliang@syphu.edu.cn (G. Chen).

Figure S1. Effects of compounds (5 μM) on H3K27me3 and H3K9me2 protein expressions in RD cells. 3

Figure S2. Antiproliferative Activities of compounds against RD cell line. 4

Figure S3. HMT assays of compounds against EZH2/G9a. 5

S4. Synthesis procedures. 6

S5. Protocols for preparative HPLC purification methods 12

S6. Acute toxicity test of **SU101**. 13

S7. NMR spectra of compounds 14

S8. MS Spectra of compounds 61

References 112

Figure S1. Effects of compounds (5 μM) on H3K27me3 and H3K9me2 protein expressions in RD cells.

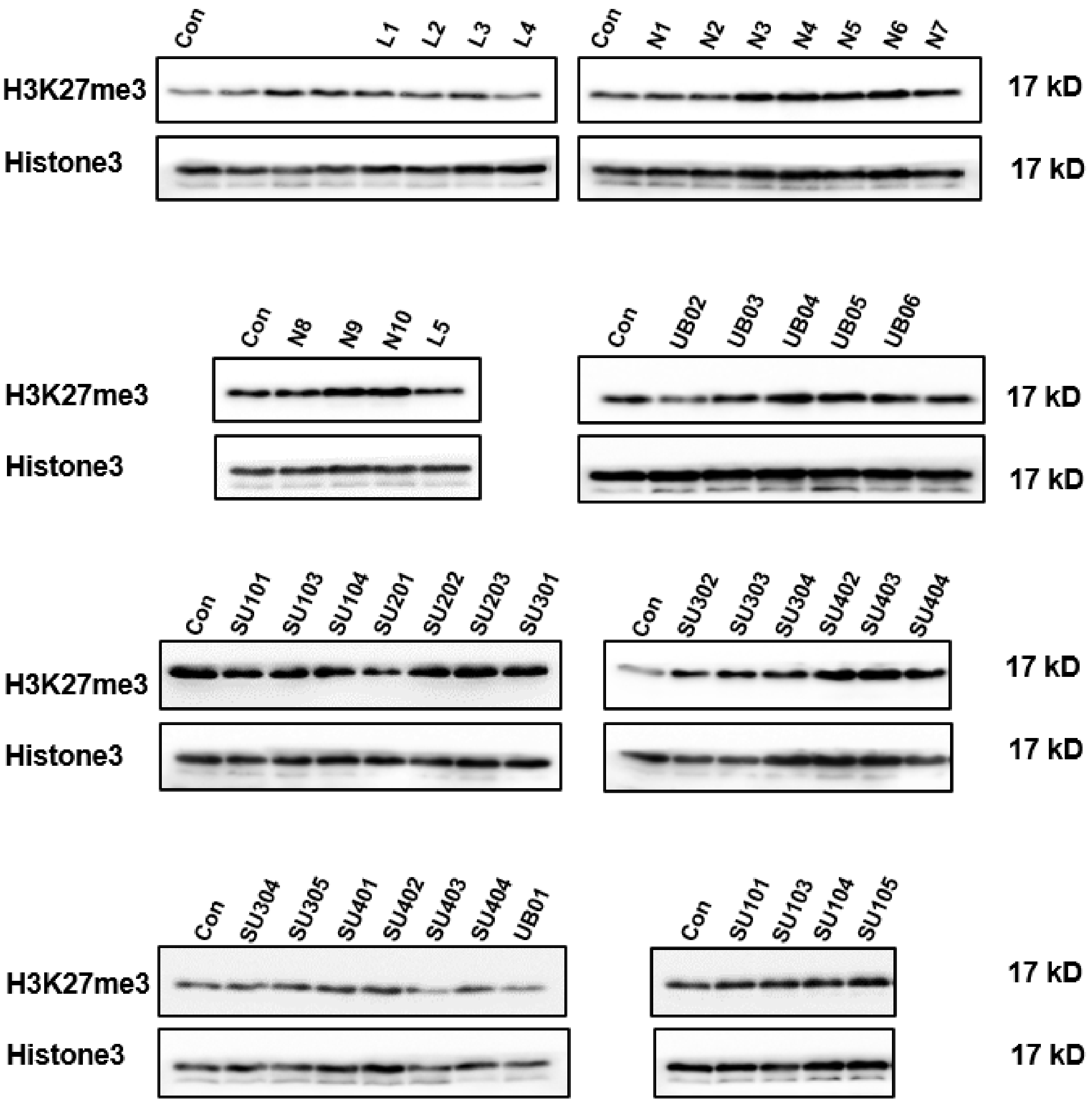

Figure S2. Antiproliferative Activities of compounds against RD cell line.

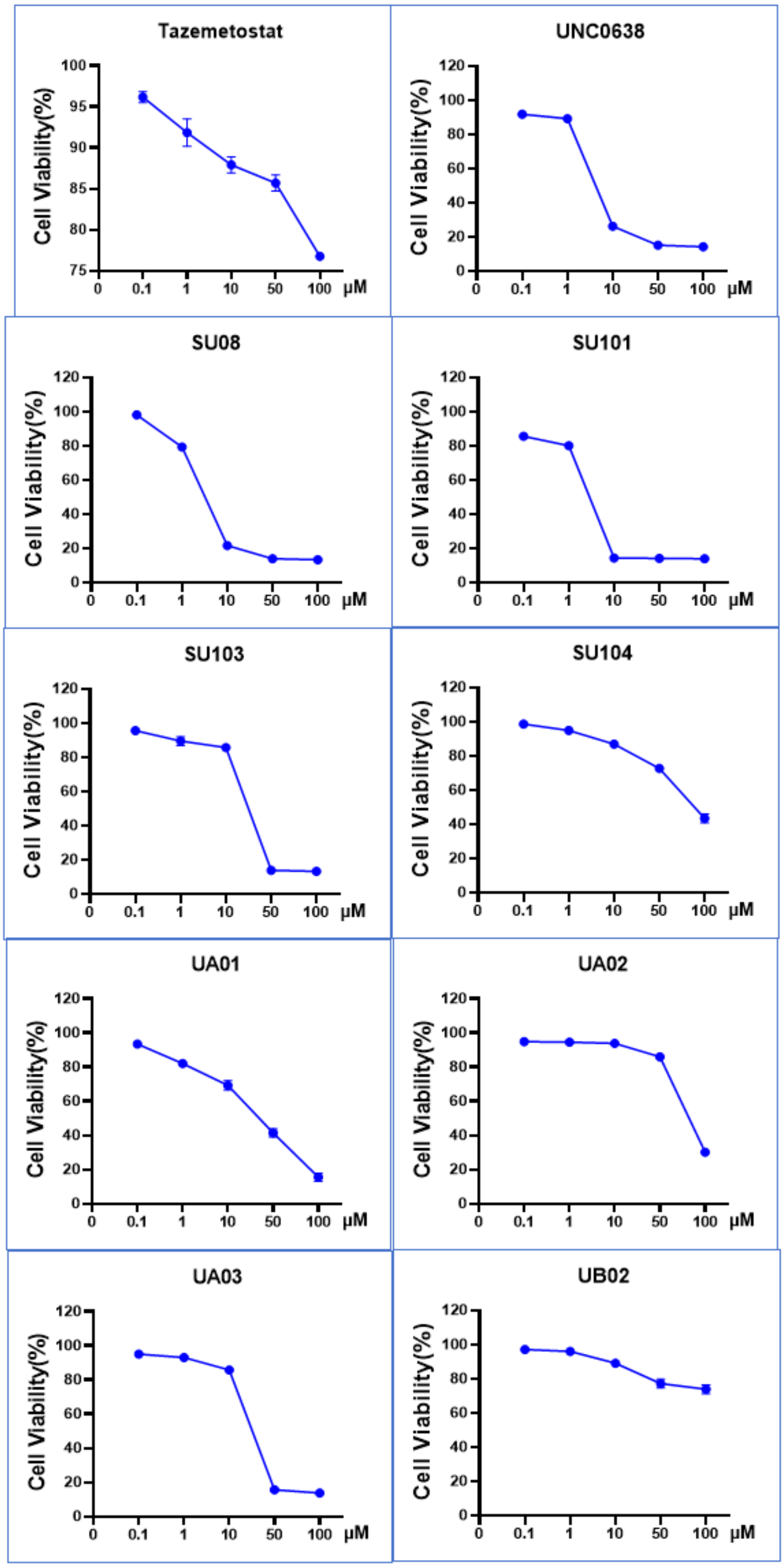

Figure S3. HMT assays of compounds against EZH2/G9a.

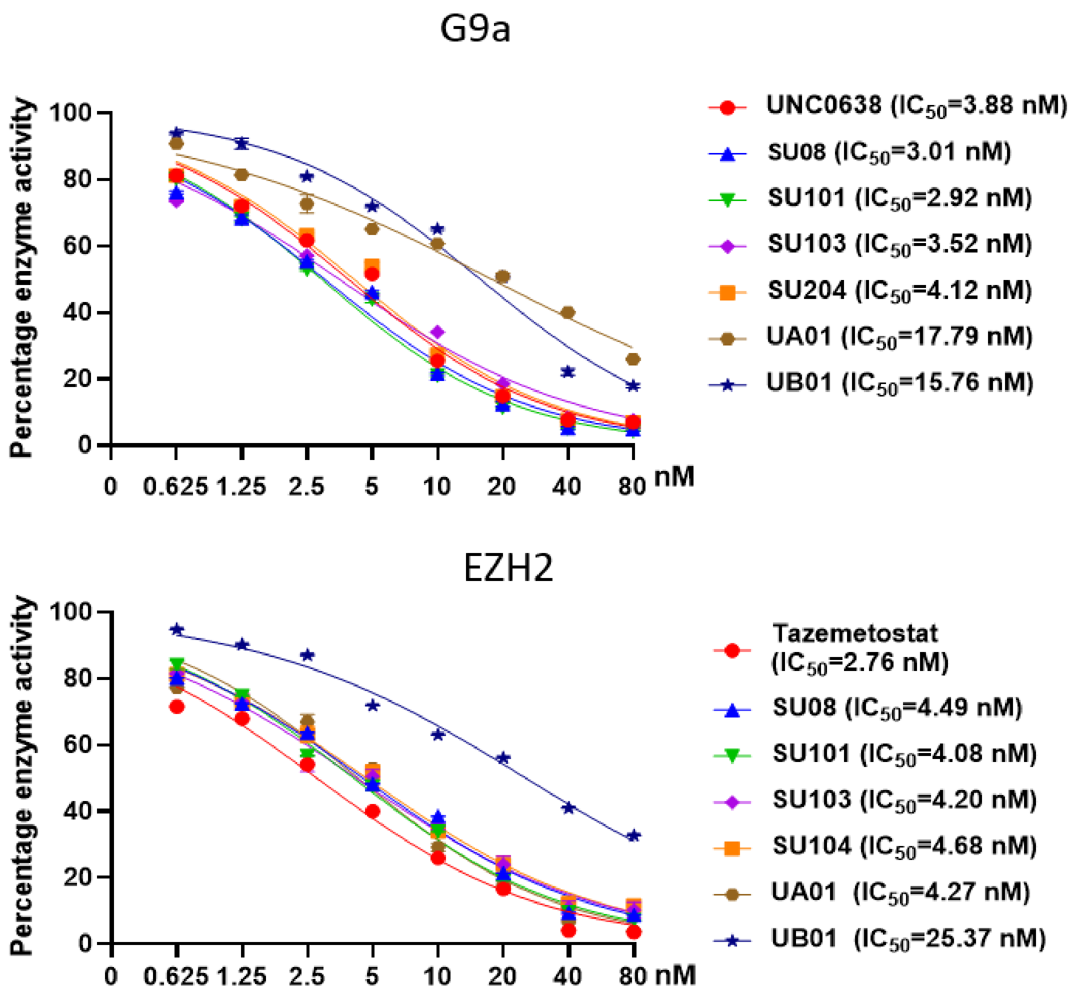

S4. Synthesis procedures.

*Methyl 4-hydroxy-3-methoxybenzoate (**2**)*. To a solution of vanillic acid 40.0 g (0.24 mol) in methanol (200 mL) was added $SOCl_2$ 20.8 mL (0.28 mol) dropwise over 1 h at 0 ºC, Upon the completion of addition, the mixture was stirred at 65 ºC for 7 h. After being concentrated *in vacuum*, the residue was dissolved in water (200 mL), extracted with DCM (200 mL × 3). The combined organic phase was washed with saturated $NaHCO_3$ twice, brine, dried over $MgSO_4$, filtered and concentrated *in vacuum* to give **2** as pale white solid 41.6 g, yield 96%.

*Methyl 4-(3-chloropropoxy)-3-methoxybenzoate (**3**)*. A mixture of intermediate **2** 31.0g (0.17 mol), 1-bromo-3-chloropropane 54.55 g (0.26 mol), K_2CO_3 70.49 g (0.51 mol), CH_3CN (300 mL) was stirred at 50 ºC for 30 h, Then, the mixture was filtered to give filtrate. After being concentrated under reduced pressure, the residue was dissolved in water (200 mL), extracted with DCM (200 mL × 3). The combined organic phase was washed with water, brine, dried over $MgSO_4$, filtered and concentrated *in vacuum* to give pale red solid 45.3 g. The crude product was recrystallized with acetone to give **3** as white crystal 37.3 g, yield 85%. **m.p.** 103−104ºC. **^{1}H NMR** (600 MHz, $CDCl_3$): *δ* 7.66 (dd, *J* = 2.6 Hz, 1H), 7.55 (d, *J* = 2.0 Hz, 1H), 6.92 (d, *J* = 8.4 Hz, 1H), 4.24 (t, *J* = 6.0 Hz, 2H), 3.90 (s, 3H), 3.89 (s, 3H), 3.78 (t, *J* = 6.2 Hz, 2H), 2.34−2.28 (m, 2H).

*Methyl 4-(3-chloropropoxy)-5-methoxy-2-nitrobenzoate (**4**)*. To a solution of intermediate **3** 30 g (0.12 mol), HOAc (30 mL), Ac_2O (30 mL) was added fuming nitric acid 9.3 g (0.14 mol) dropwise over 1 h at 0 ºC. Upon the completion of addition, the mixture was stirred at room temperature for 5 h. Then the reaction mixture was poured into ice-water (300 mL) under stirring and precipitate was collected by filtration. Compound **4** 34.0 g was obtained as yellow solid, yield 97%. **m.p.** 63−65 ºC. **^{1}H NMR** (600 MHz, $CDCl_3$): *δ* 7.48 (s, 1H), 7.07 (s, 1H), 4.26 (t, *J* = 5.9 Hz, 2H), 3.95 (s, 3H), 3.91 (s, 3H), 3.78 (t, *J* = 6.2 Hz, 2H), 2.35 (m, 2H). **ESI-MS** m/z: 325.27$[M + Na]^+$.

*Methyl 5-methoxy-2-nitro-4-(3-(pyrrolidin-1-yl)propoxy)-benzoate (**5**)*. A solution of intermediate **4** 11.12 g (0.037 mol), KI 6.14 g (0.037 mol), CH_3CN (60 mL) was stirred at reflux for 30 min. After being cooled, K_2CO_3 15.34 g (0.11 mol) and pyrrolidine 2.74 g (0.039 mol) was added. Then the reaction mixture was stirred at 80 ºC for 10 h. After being filtered, the filtrate was concentrated under reduced pressure. The residue was dissolved in water 100 mL, extracted with EA (100 mL × 3). The combined organic phase was washed with water, brine, dried over $MgSO_4$, filtered and concentrated *in vacuum* to give brown oil. The crude product was purified by silica gel column chromatography (EA: PE: TEA = 1:3:0.005) to afford intermediate **5** 8.3 g as yellow oil, yield 66%. **^{1}H NMR** (600 MHz, $CDCl_3$): *δ* 7.50 (s, 1H), 7.07 (s, 1H), 4.20 (t, *J* = 6.6 Hz, 2H), 3.95 (s, 3H), 3.91 (s, 3H), 2.68−2.59 (m, 6H), 2.15−2.09 (m,

2H), 1.82 (brs, 4H). **ESI-MS** m/z: 339.32 $[M + H]^+$.

*Methyl 2-amino-5-methoxy-4-(3-(pyrrolidin-1-yl)propoxy) benzoate (**6**) [1]*. To a solution of intermediate **5** 4.5 g (13.3 mmol), Sn powder 4.8 g (26.6 mmol), EtOH (45 mL) flowed into HCl gas for 5 h, After being filtered, the filter cake was dissolved in water (10 mL). EA (50 mL) was added, followed by dropping 25% $NH_3 \cdot H_2O$ under ice bath till pH = 9. The organic phase was separated, washed with water, brine, dried over $MgSO_4$, filtered and concentrated *in vacuum* to give **6** 3.2 g as yellow solid, yield 85%. **m.p.** 73−74 °C。 **^{1}H NMR** (600 MHz, $CDCl_3$): δ 7.30 (s, 1H), 6.19 (s, 1H), 5.55 (s, 2H), 4.10 (t, J = 6.6 Hz, 2H), 3.85 (s, 3H), 3.80 (s, 3H), 2.76−2.67 (m, 6H), 2.19−2.11 (m, 2H), 1.88-1.84 (m, 4H). **ESI-MS** m/z: 308.17 $[M + H]^+$.

6-Methoxy-7-(3-(pyrrolidin-1-yl)propoxy)quinazoline-2,4(1H,3H)-dione (7) [2,3]. A solution of intermediate **6** 4.5 g (14.59 mmol), NaOCN 1.4 g (21.88 mmol), HOAc 5 mL, water (10 mL) was stirred at 65 °C for 10 h. After being cooled to room temperature, methanol (10 mL) and water (20 mL) was added to the mixture, NaOH solid was added in batches until pH = 9. Then the reaction mixture was stirred at 65 °C for 2 h. Methanol was removed under reduced pressure. The residue was filtered and the solid was washed with water for twice, dried to afford intermediate **7** 4.13 g as white solid, yield 89%. **ESI-MS** m/z: 320.4 $[M + H]^+$.

*2,4-Dichloro-6-methoxy-7-(3-(pyrrolidin-1-yl)propoxy)- quinazoline (**8**)*. To a solution of intermediate **7** 4.13 g (12.9 mmol) and acetone (20 mL) under ice bath, was dropped $POCl_3$ 5.93 g (38.7 mmol), followed by addition of DIPEA 1.67 g (12.9 mmol) dropwise. After addition, the reaction mixture was stirred at 80 °C for 5 h. Then the solution was concentrated under reduced pressure, the residue was dissolved in DCM (50 mL). After being poured into ice-water (50 mL), 30% NaOH solution was dropped to the mixture till pH = 9. The DCM phase was separated, water phase was extracted with DCM (30 mL × 2). The combined organic phase was washed with water, brine, dried over $MgSO_4$, filtered and concentrated *in vacuum* to give yellow solid 3.92 g. The crude product was purified by silica gel column chromatography (MeOH: DCM: TEA = 1:20:0.005) to afford intermediate **8** 3.01 g as white solid, yield 66%. **m.p.** 95−97 °C **^{1}H NMR** (600 MHz, $CDCl_3$): δ 7.34 (s, 1H), 7.29 (s, 1H), 4.29 (t, J = 8.0 Hz, 2H), 4.05 (s, 3H), 2.69 (t, J = 4.0 Hz, 2H), 2.56−2.47 (m, 4H), 2.19−2.12 (m, 2H), 1.81−1.79 (m, 4H). **ESI-MS** m/z: 356.2 $[M + H]^+$.

*General procedure for the synthesis of intermediates **9a–9c** and **10***. To a 25 mL round-bottom flask were added intermediate **8** (1.0 g, 2.80 mmol), corresponding amine (3.00 mmol), finely ground potassium carbonate (1.90 g, 14.0 mmol), and DMF (2 mL). The flask was purged with argon three times and heated in an oil bath at 70 °C. After 10 h, the reaction was stopped, and water was added. The mixture was filtered under suction to afford the target compound.

3-(((2-chloro-6-methoxy-7-(3-(pyrrolidin-1-yl)propoxy)quinazolin-4-yl)amino)methyl)-4-

*methoxy-6-methylpyridin-2(1H)-one (**9a**)* was obtained as a yellow solid (1.1 g, yield 85%).

*3-(((2-chloro-6-methoxy-7-(3-(pyrrolidin-1-yl)propoxy)quinazolin-4-yl)amino)methyl)-4-methyl-6-propylpyridin-2(1H)-one (**9b**)* was obtained as a yellow solid (1.2 g, yield 86%).

*3-(((2-chloro-6-methoxy-7-(3-(pyrrolidin-1-yl)propoxy)quinazolin-4-yl)amino)methyl)-6-ethyl-4-methylpyridin-2(1H)-one (**9c**)* was obtained as a yellow solid (1.1 g, yield 81%).

*3-(((2-chloro-6-methoxy-7-(3-(pyrrolidin-1-yl)propoxy)quinazolin-4-yl)amino)methyl)-4-methyl-5,6,7,8-tetrahydroquinolin-2(1H)-one (**10**)* was obtained as a yellow solid (1.2 g, yield 83%).

2-chloro-6-methoxy-7-(3-(pyrrolidin-1-yl)propoxy)quinazolin-4-amine (11)

To a 250 mL round-bottom flask were added intermediate **8** (9 g, 0.025 mol), THF (50 mL), 25% aqueous ammonia (50 mL), and finely ground potassium carbonate (10.5 g, 0.076 mol). The mixture was stirred at room temperature. After 5 h, the reaction was complete. THF was removed under reduced pressure, and the residue was poured into water (50 mL). The aqueous layer was extracted with DCM (100 mL × 3). The combined organic layers were washed once with water and once with brine, then dried over anhydrous magnesium sulfate overnight. On the following day, the mixture was filtered, and the filtrate was concentrated to afford the crude product as a yellow solid (7.8 g, yield 91.5%). **ESI-MS** m/z: 337.1 $[M+H]^+$.

6-methoxy-2-(piperidin-1-yl)-7-(3-(pyrrolidin-1-yl)propoxy)quinazolin-4-amine (12a)

Using 2-chloro-6-methoxy-7-(3-(pyrrolidin-1-yl)propoxy)quinazolin-4-amine (**11**) and piperidine as starting materials, **12a** was synthesized according to **General procedure 1**. The work-up was modified to silica gel column chromatography, eluting with a gradient of MeOH/DCM/TEA (1:50:0.02) to (1:20:0.02), to afford **12a** as a yellow solid (0.26 g, yield 38%).

2-(4,4-difluoropiperidin-1-yl)-6-methoxy-7-(3-(pyrrolidin-1-yl)propoxy)quinazolin-4-amine (12b)

Using 2-chloro-6-methoxy-7-(3-(pyrrolidin-1-yl)propoxy)quinazolin-4-amine (**11**) and 4,4-difluoropiperidine hydrochloride as starting materials, **12b** was synthesized according to **General procedure 1**. The work-up was modified to silica gel column chromatography, eluting with a gradient of MeOH/DCM/TEA (1:50:0.02) to (1:20:0.02), to afford **12b** as a yellow solid (1.04 g, yield 83%). **ESI-MS** m/z: 422.2 $[M+H]^+$.

*N,N'-dibenzyloxalamide (**DBO**) [4].* To a 100 mL three-necked flask were added benzylamine (1.1 g, 10.3 mmol), triethylamine (1.78 g, 12.88 mmol), and dry THF (6 mL). The flask was placed in a cold bath and cooled to 0 °C. A solution of oxalyl chloride (0.65 g, 5.15 mmol) in

dry THF (5 mL) was added dropwise while maintaining the internal temperature at 0–10 °C. After the addition, the mixture was allowed to warm to room temperature. After 2 h, TLC indicated completion of the reaction. The mixture was concentrated under reduced pressure, and the residue was poured into water (30 mL). After stirring for 3 min, the precipitate was collected by filtration under suction. The filter cake was washed once with water and twice with diethyl ether (4 mL each), then dried to afford **DBO** as a white solid (1.05 g, yield 79.5%). **ESI-MS** m/z: 291.1 $[M+Na]^+$.

*2-chloro-N-((4,6-dimethyl-2-oxo-1,2-dihydropyridin-3-yl)methyl)acetamide (**15**).*To a 100 mL three-necked flask were added intermediate **14** (2 g, 13.2 mmol), dichloromethane (20 mL), and triethylamine (2.6 g, 26.4 mmol). The flask was cooled to 0 °C in a cold bath, and chloroacetyl chloride (1.56 g, 13.86 mmol) was added dropwise while maintaining the temperature at 0–5 °C. After addition, the mixture was allowed to warm to room temperature. After 2 h, the reaction was complete. The mixture was concentrated, and the residue was poured into water (50 mL). The precipitated solid was collected by filtration under suction to give **15** as a white solid (1.63 g, yield 54%). **ESI-MS** m/z: 229.1 $[M+H]^+$.

*2-amino-N-((4,6-dimethyl-2-oxo-1,2-dihydropyridin-3-yl)methyl)acetamide (**16**).* To a 100 mL three-necked flask were added **15** (0.3 g, 1.24 mmol), ethanol (6 mL), and 25% aqueous ammonia (3 mL). The flask was heated in an oil bath at 70 °C. After 5 h, additional aqueous ammonia (1 mL) was added, and stirring was continued for another 2 h until the reaction was complete. The solvent was removed under reduced pressure, and the crude product **16** was used directly in the next step without further purification.

*2-((2-chloro-6-methoxy-7-(3-(pyrrolidin-1-yl)propoxy)quinazolin-4-yl)amino)-N-((4,6-dimethyl-2-oxo-1,2-dihydropyridin-3-yl)methyl)acetamide (**17**) [5,6].* To a 100 mL three-necked flask were added intermediate **8** (50 mg, 0.14 mmol), intermediate **16** (44 mg, 0.21 mmol), CuI (1.3 mg, 0.007 mmol), DBO (3.8 mg, 0.014 mmol), and $K_3PO_4 \cdot H_2O$ (37 mg, 0.14 mmol). The flask was purged with argon three times, and then DMSO (1.5 mL) was added via syringe. The mixture was heated in an oil bath at 120 °C overnight. After completion, the reaction mixture was poured into water (10 mL) and extracted with dichloromethane (10 mL × 3). The combined organic layers were washed once with water and once with brine, then dried over anhydrous magnesium sulfate. Filtration and concentration under reduced pressure gave a crude product (65 mg). This residue was adsorbed onto 90 mg of silica gel and chromatographed over 1 g of silica gel, eluting with MeOH/DCM (1:100) containing a trace of TEA, to afford **17** as a white solid (20 mg, yield 27%).**^{1}H NMR** (400 MHz, DMSO-d_6): δ 11.45 (s, 1H), 8.62 (t, J = 5.8 Hz, 1H), 7.97 (t, J = 5.0 Hz, 1H), 7.66 (s, 1H), 7.08 (s, 1H), 5.84 (s, 1H), 4.19–4.14 (m, 4H), 4.07 (d, J = 5.7 Hz, 2H), 3.89 (s, 3H), 2.83–2.80 (m, 6H), 2.13 (s, 3H), 2.10 (s, 3H), 2.06–2.01 (m, 2H), 1.79 (s, 4H). **ESI-MS**: 529.2 $[M+H]^+$.

***General procedure 3** for the Synthesis of **19** and **23** [7,8].* To a 100 mL three-necked flask were added **18** or **22** (0.064 mol) and urea (38.4 g, 0.64 mol). The flask was heated in a sand bath at 230 °C to maintain an internal temperature of 200 °C. After 1.5 h, TLC indicated complete consumption of the starting material. A 20% NaOH solution (100 mL) was slowly added, and the mixture was heated in an oil bath at 100 °C. After 4 h, activated carbon (1 g) was added, and stirring was continued at 100 °C for another 1 h. The reaction was then stopped, and the mixture was filtered under suction. The filter cake was washed twice with water and discarded. The filtrate was adjusted to pH 4 with 6 N HCl, upon which a large amount of off-white solid precipitated. The solid was collected by filtration under suction to afford target compound.

*Thieno[3,2-d]pyrimidine-2,4(1H,3H)-dione (**19**)* was obtained as off-white solid (9.42 g, crude yield 88%).

*thieno[2,3-d]pyrimidine-2,4(1H,3H)-dione (**23**)* was obtained as brown solid (12.4 g, yield 116%).

***General procedure 4** for the Synthesis of **20** and **24**.* To a 100 mL round-bottomed flask were added **19** or **23** (13.5 mmol) and phosphorus oxychloride (8.18 g, 67.5 mmol). The flask was cooled in an ice-water bath, and N,N-dimethylaniline (2.06 g, 13.5 mmol) was added dropwise. After stirring for 10 min, the mixture was heated to reflux. After 6 h, the reaction was complete. Most of the phosphorus oxychloride was removed by distillation under reduced pressure. The residue was dissolved in THF (5 mL) while hot and then slowly poured into ice-water (30 mL). The pH of the mixture was adjusted to 9 by dropwise addition of 30% aqueous NaOH solution under ice-bath cooling. The precipitate was collected by filtration under suction, and the filter cake was washed twice with water to afford a dark red solid (2.53 g). This crude product was purified by flash column chromatography (EA/PE = 1:10) to give the title compound as a white solid.

*2,4-Dichlorothieno[3,2-d]pyrimidine (**20**)* was obtained as white solid (7.2 g, crude yield 59%). **^{1}H NMR** (400 MHz, $CDCl_3$), δ 8.13 (d, J = 5.5 Hz, 1H), 7.55 (d, J = 5.5 Hz, 1H). **ESI-MS** m/z: 205.1 $[M+H]^+$.

*2,4-Dichlorothieno[2,3-d]pyrimidine (**24**)* was obtained as white solid (6.76 g, crude yield 55%).**^{1}H NMR** (400 MHz, $CDCl_3$), δ 7.63 (d, J = 6.0 Hz, 1H), 7.44 (d, J = 6.0 Hz, 1H).

***General procedure 5** for the Synthesis of **21** and **25**.* To a 50 mL round-bottomed flask were added **20** or **24** (8.06 mmol), 3-(aminomethyl)-4,6-dimethylpyridin-2(1*H*)-one (1.36 g, 8.90 mmol), and potassium carbonate powder (3.18 g, 40.3 mmol) in DMF (10 mL). The flask was purged with argon three times and then heated in an oil bath at 70 °C. After 7.5 h, the reaction was complete. The reaction mixture was poured into water (50 mL) and extracted with ethyl acetate (20 mL × 3). The combined organic layers were washed twice with water and once with

brine, then dried over anhydrous magnesium sulfate. The solvent was removed under reduced pressure to afford the product as a pale yellow solid (2.54 g, yield 84.4%).

3-(((2-Chlorothieno[3,2-d]pyrimidin-4-yl)amino)methyl)-4,6-dimethylpyridin-2(1H)-one ***(21)*** was obtained as white solid (6.3 g, crude yield 109%).^{1}**H NMR** (400 MHz, $CDCl_3$), δ 11.52 (s, 1H), 8.42 (t, J = 4.1 Hz, 1H), 8.12 (d, J = 5.4 Hz, 1H), 7.31 (d, J = 5.3 Hz, 1H), 5.89 (s, 1H), 4.44 (d, J = 4.5 Hz, 2H), 2.25 (s, 3H), 2.13 (s, 3H). **ESI-MS** m/z: 321.0, 323.0 $[M+H]^+$.

3-(((2-Chlorothieno[2,3-d]pyrimidin-4-yl)amino)methyl)-4,6-dimethylpyridin-2(1H)-one ***(25)*** was obtained as white solid (5.8 g, crude yield 95%).

S5. Protocols for preparative HPLC purification methods

Reverse phase HPLC was carried out on a Waters prep 150 LC with a Daisogel C18, 10 mm, 100 Å, 250*30.0 mmI.D and UV detection. Solvent A: water with 0.075% trifluoroacetic acid; Solvent B: acetonitrile.

Method 1: A: B = 77:23 (*v*/*v*)

Method 2: A: B = 75:25 (*v*/*v*)

Method 3: Gradient: 20% of B to 40% of B within 10 minutes; then 40% of B for 5 min, then 40% of B to 90% of B within 5 minutes, then 90% B over 5 minutes.

Method 4: A: B = 70:30 (*v*/*v*)

Method 5: A: B = 80:20 (*v*/*v*)

Method 6: Gradient: 20% of B to 30% of B within 10 minutes; then 30% of B for 5 min, then 30% of B to 90% of B within 5 minutes, then 90% B over 5 minutes.

S6. Acute toxicity test of **SU101**.

Species: Kunming mice (6 males, 20-22 g) were purchased from Liaoning Changsheng Biotechnology Co., Ltd. (Liaoning, China) (License No.: SCXK (Liao) 2020-0001).

SU101 was administered by intraperitoneal administration. Mice were dosed sequentially according to the table and observed for 14 days.

Table S1. AOT425statpgm (Version: 1.0) Test Results and Recommendations.

Test Seq.	Animal ID	Dose (mg/kg)	Short-term Result	Long-term Result
**1**	1	55	X	X
**2**	2	17.5	O	O
**3**	3	55	X	X
**4**	4	17.5	O	O
**5**	5	55	X	X
**6**	6	17.5	O	O

(X = Died, O = Survived)

Dose Recommendation: The main test is complete.

Stopping criteria met: 5 reversals in 6 tests. LR criterion.

Statistical Estimate based on long term outcomes:

Estimated LD_{50} = 31.02 (Based on an assumed sigma of 0.5).

Approximate 95% confidence interval is 17.5 to 55.

S7. NMR spectra of compounds

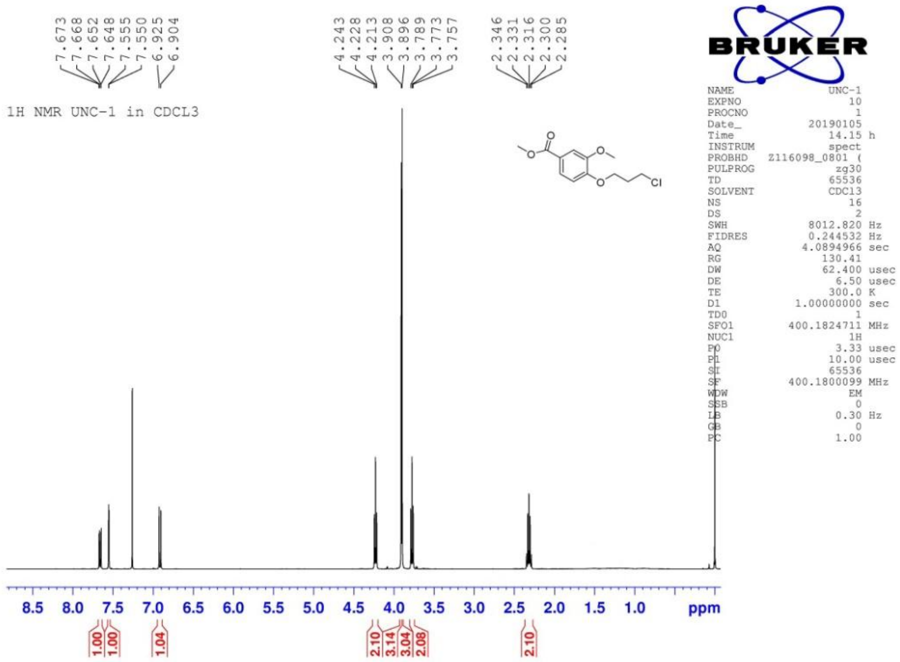

^{1}H NMR Spectrum of 3

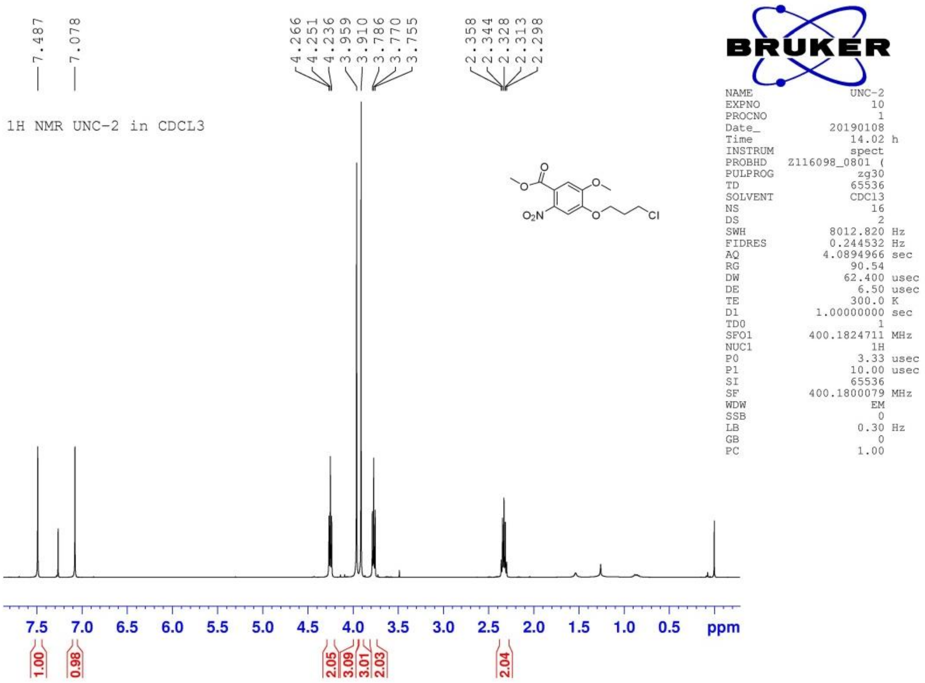

^{1}H NMR Spectrum of 4

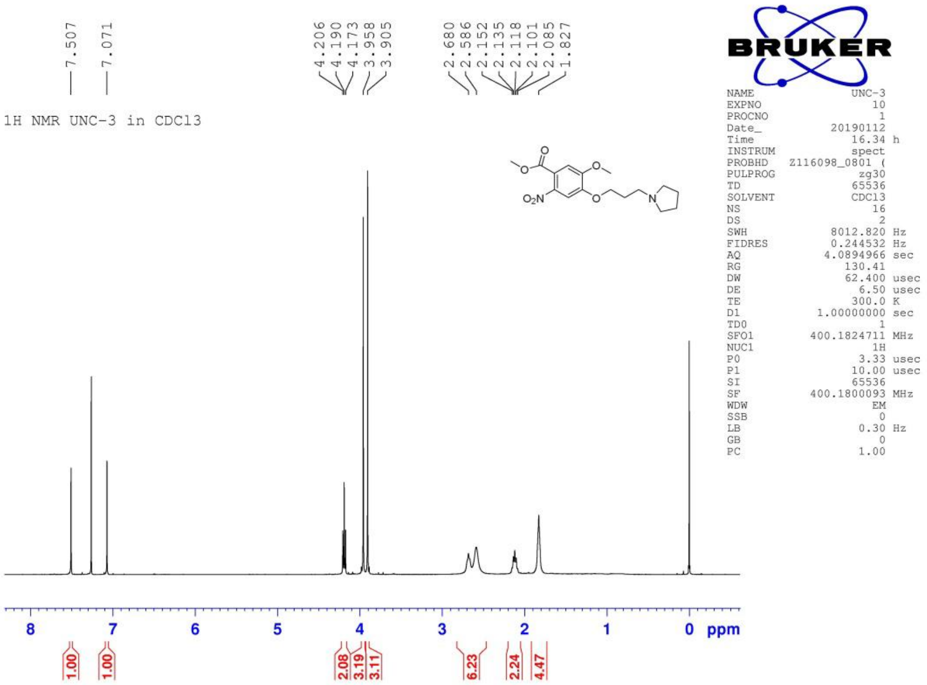

^{1}H NMR Spectrum of 5

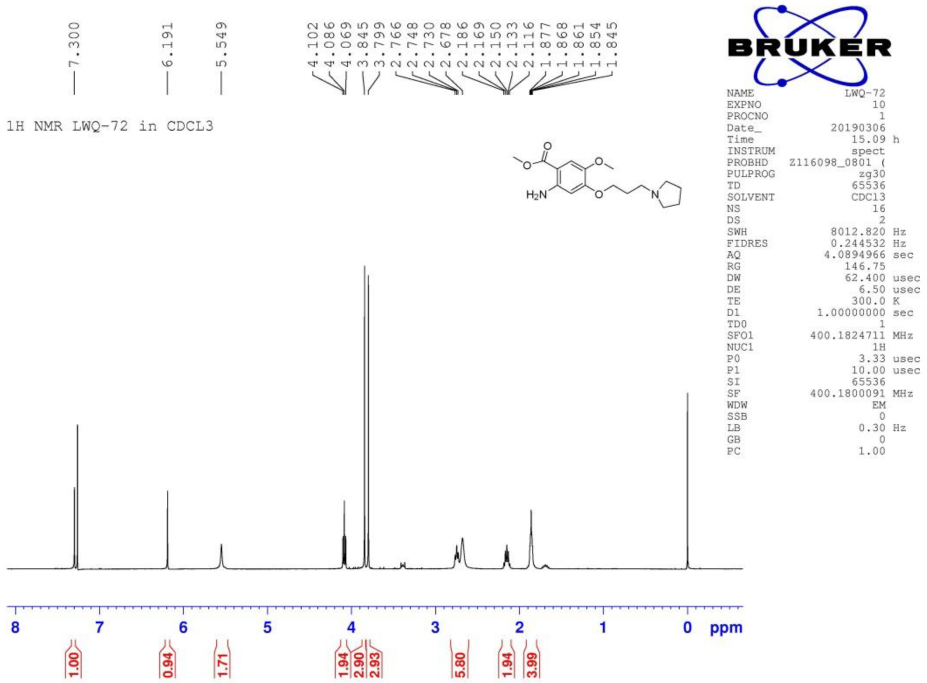

^{1}H NMR Spectrum of 6

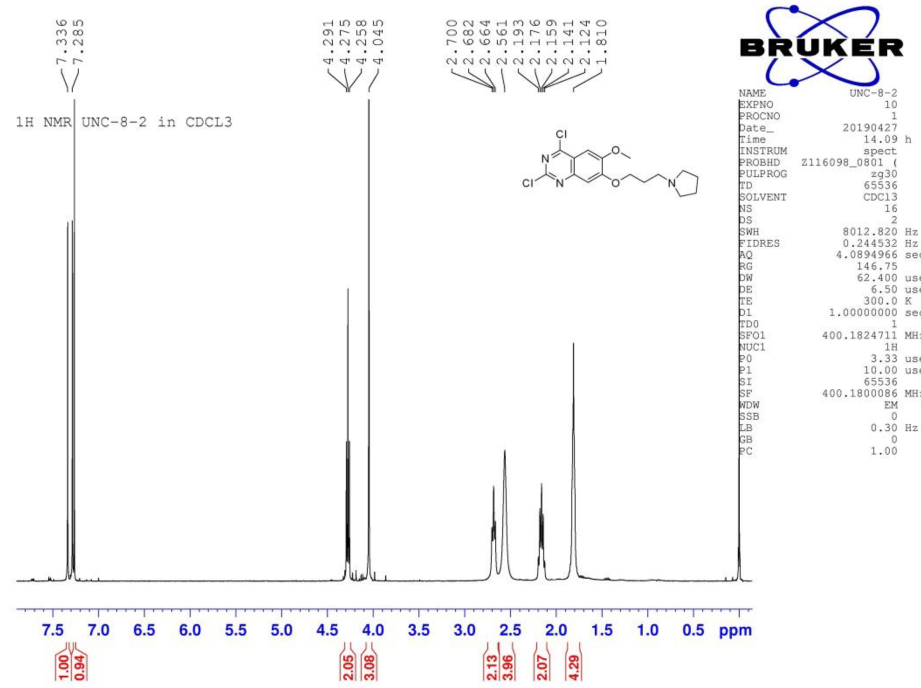

^{1}H NMR Spectrum of 8

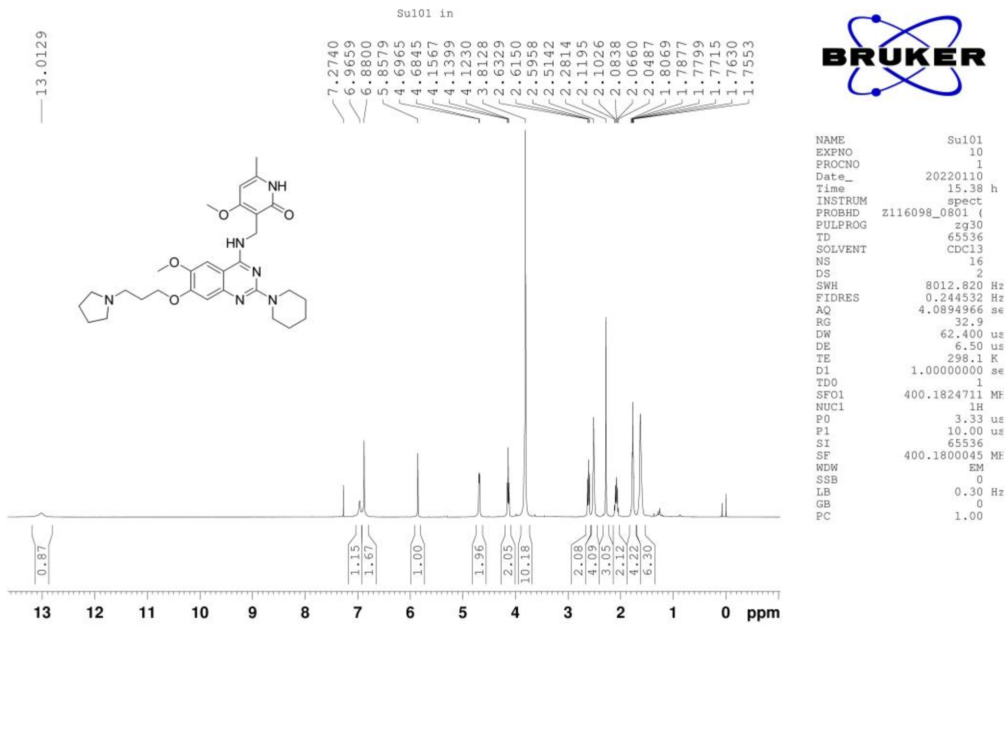

^{1}H NMR Spectrum of SU101

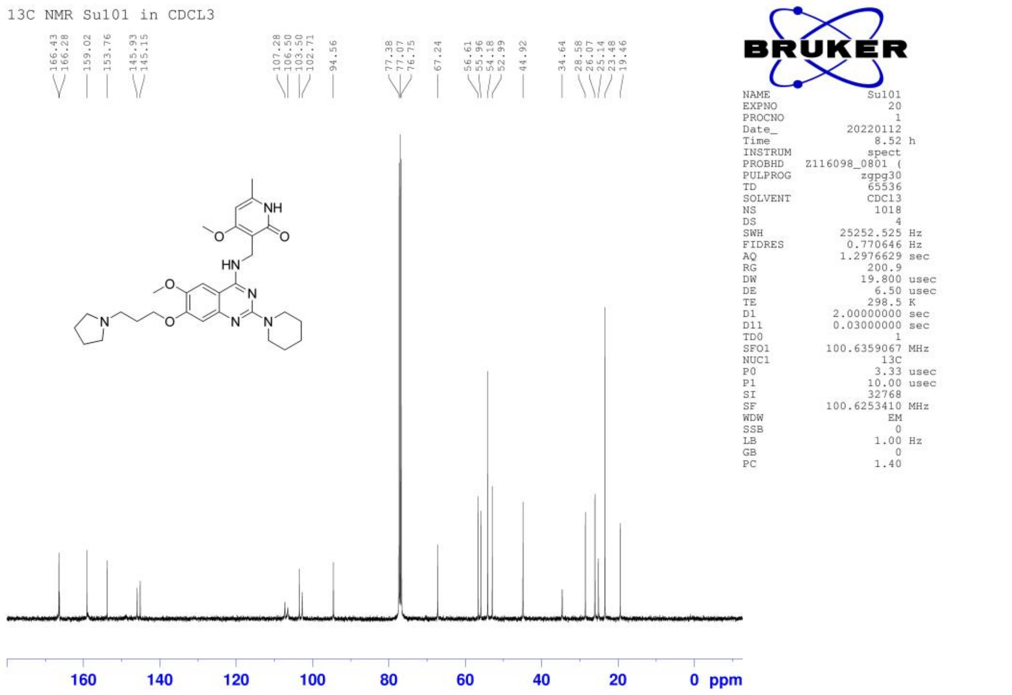

^{13}C NMR Spectrum of SU101

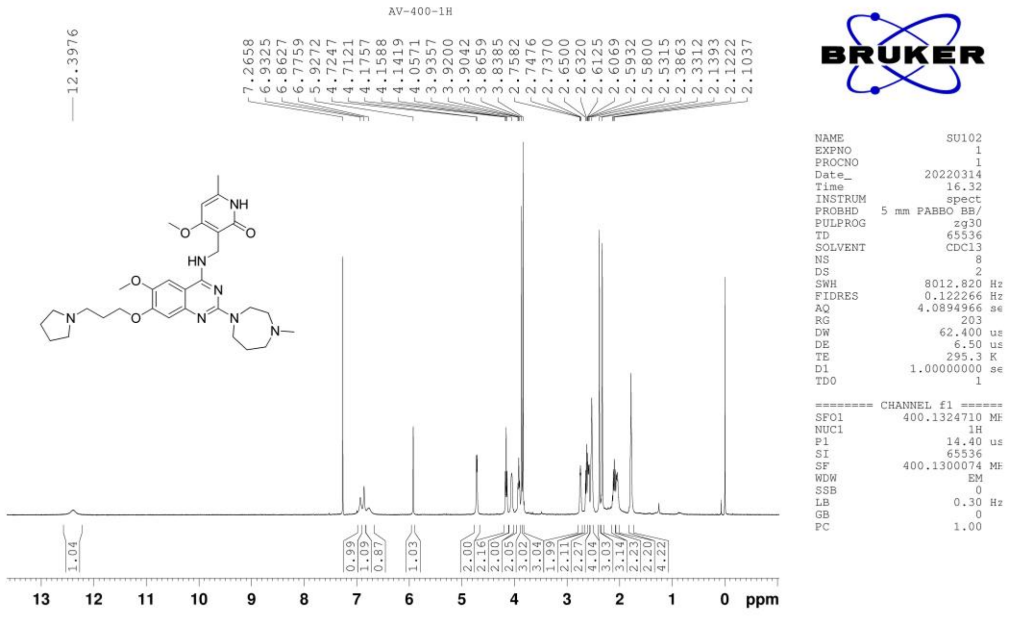

^{1}H NMR Spectrum of SU102

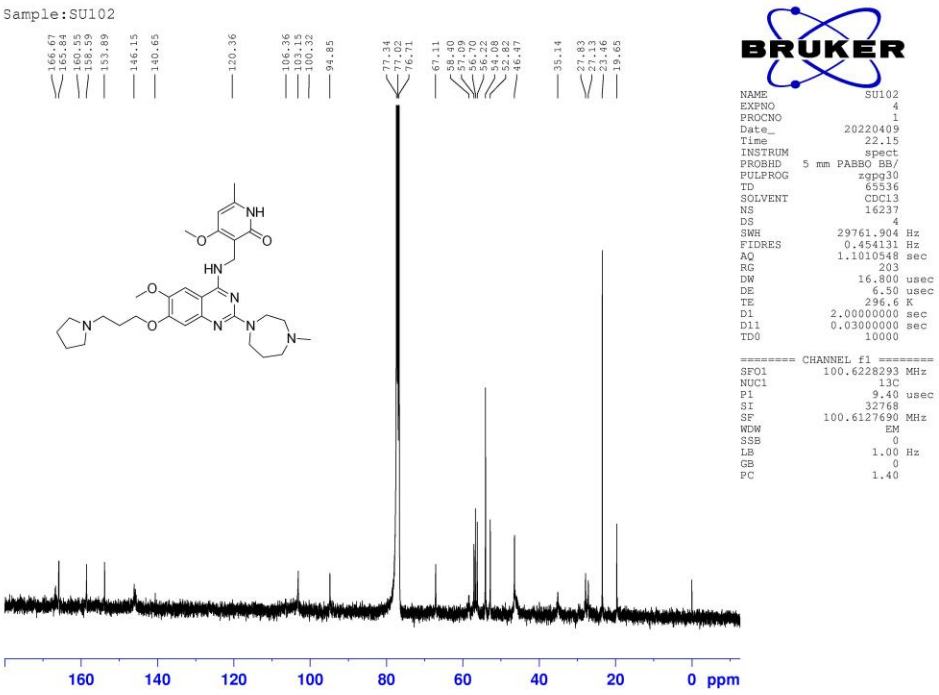

^{13}C NMR Spectrum of SU102

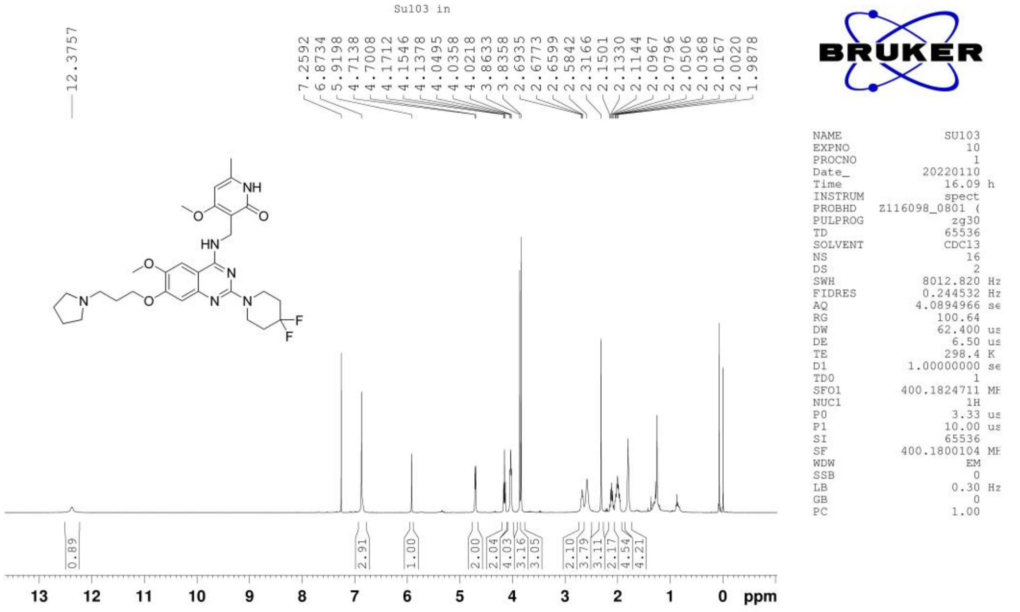

^{1}H NMR Spectrum of SU103

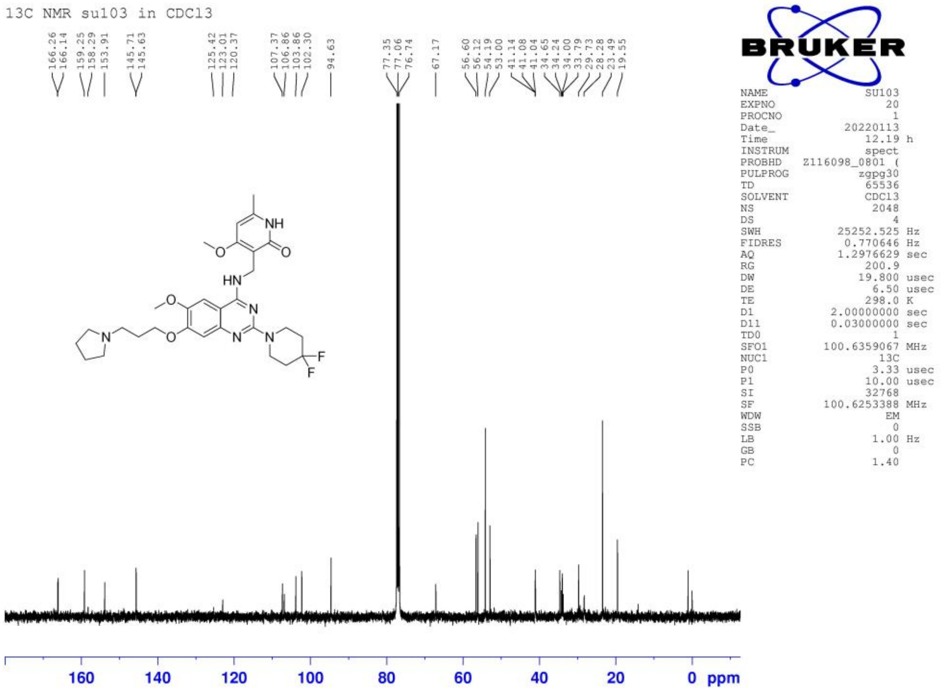

^{13}C NMR Spectrum of SU103

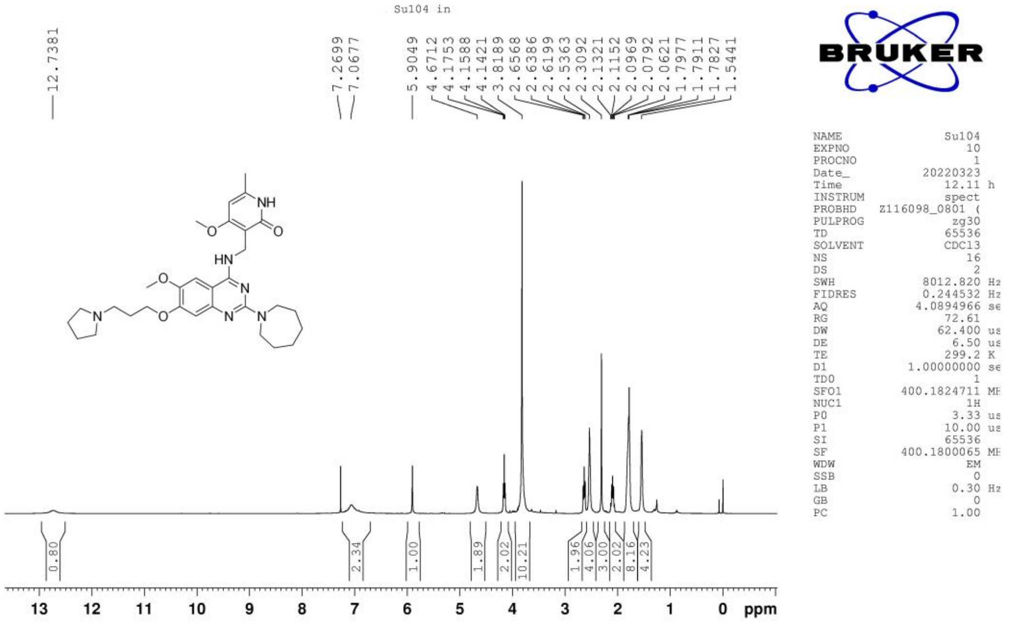

^{1}H NMR Spectrum of SU104

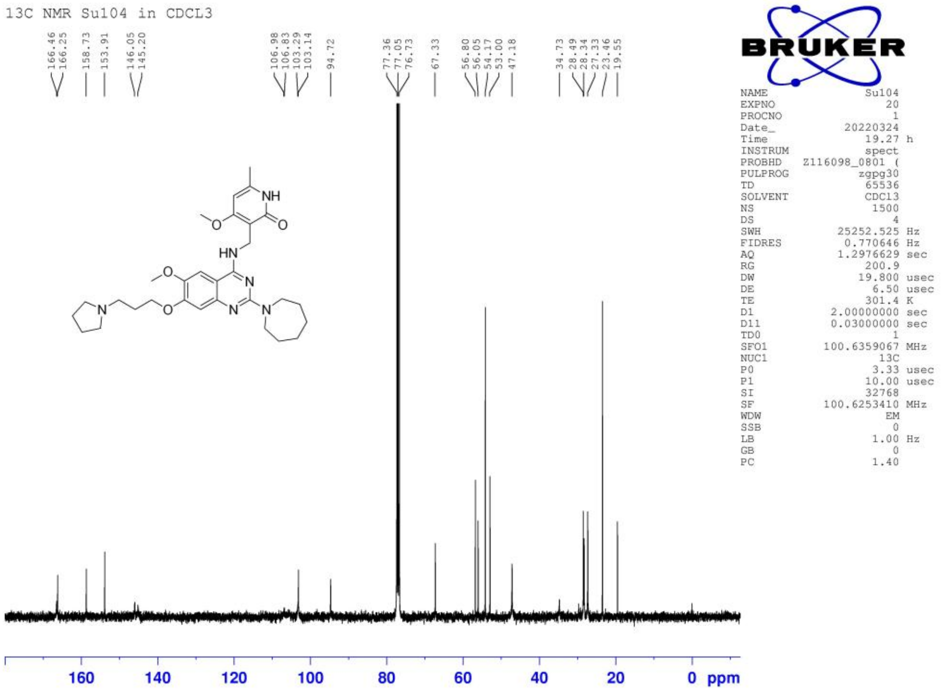

^{13}C NMR Spectrum of SU104

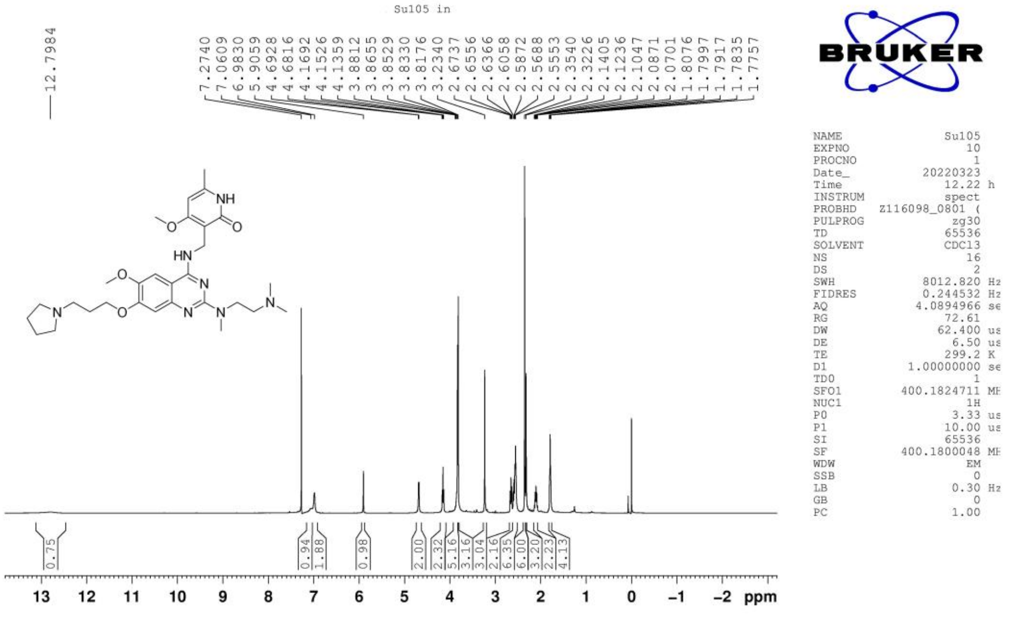

^{1}H NMR Spectrum of SU105

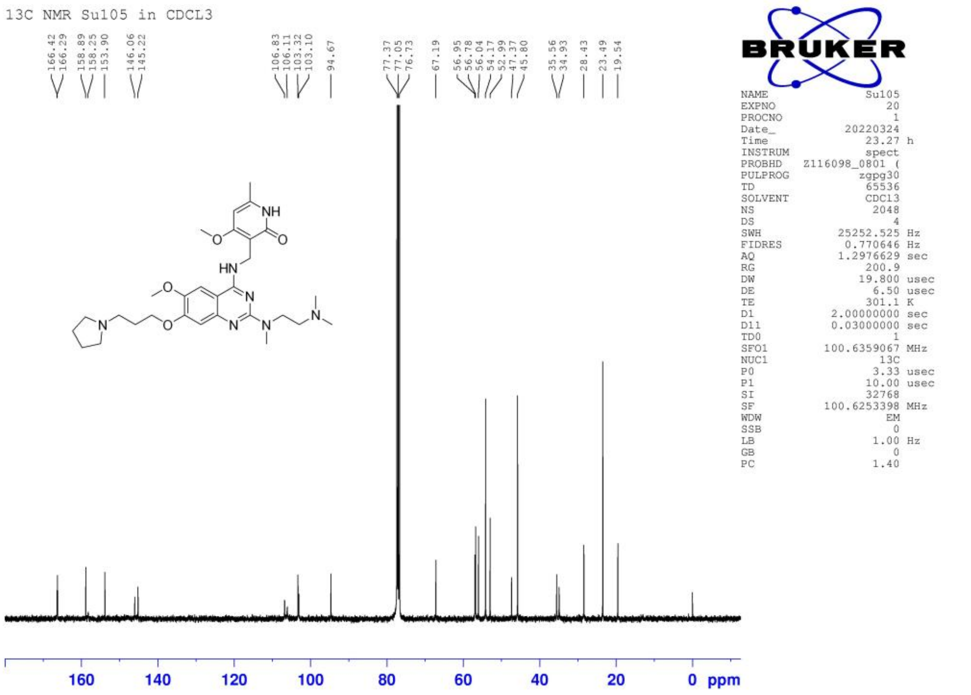

^{13}C NMR Spectrum of SU105

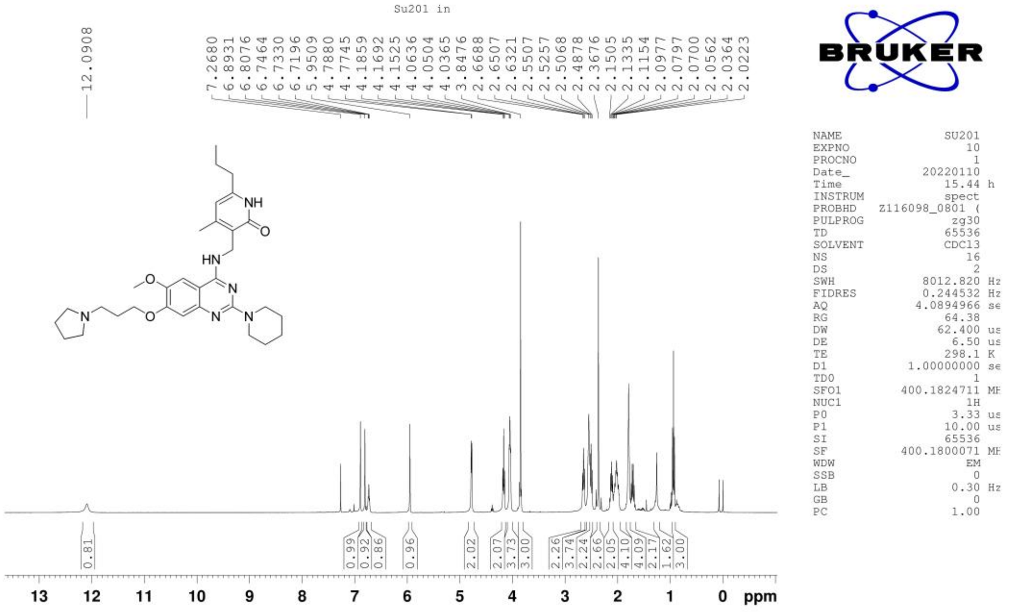

^{1}H NMR Spectrum of SU201

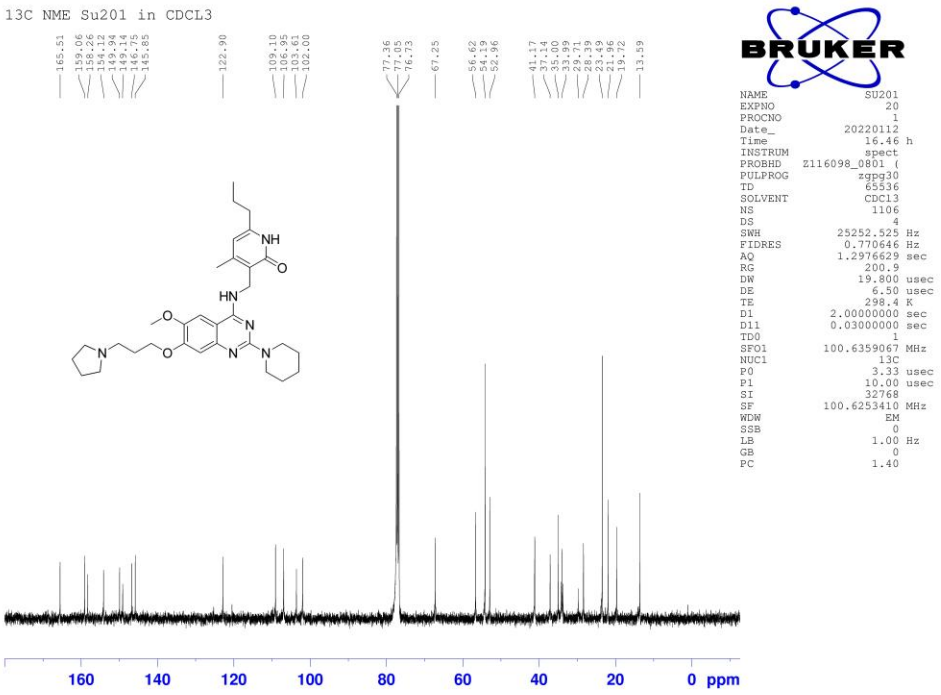

^{13}C NMR Spectrum of SU201

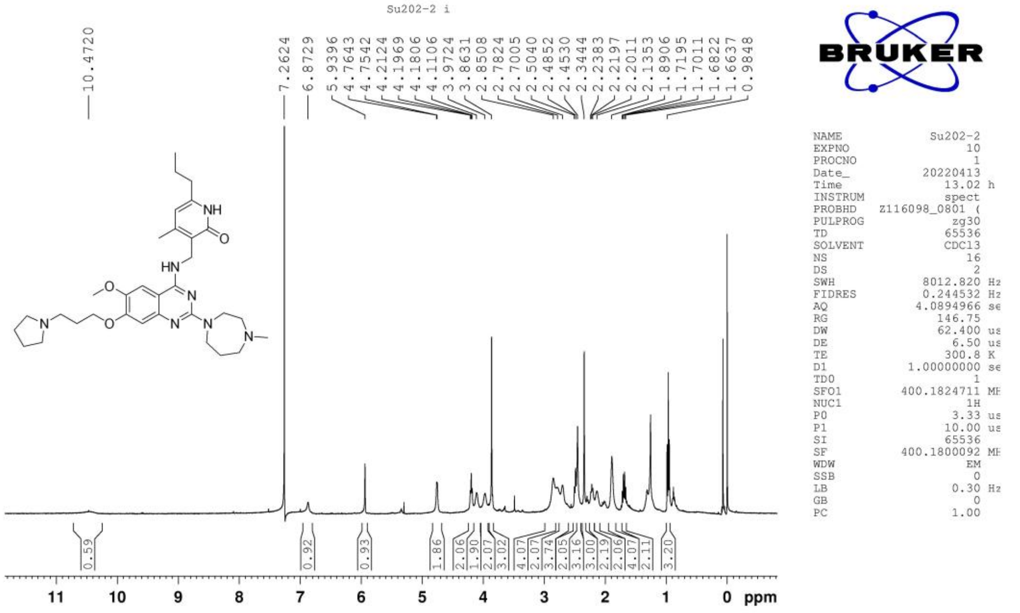

^{1}H NMR Spectrum of SU202

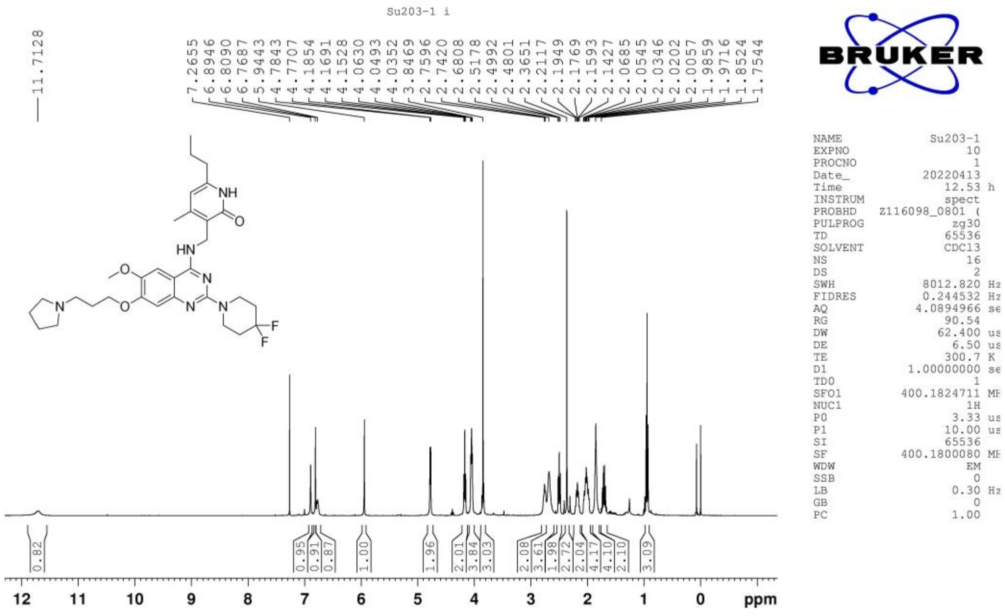

^{1}H NMR Spectrum of SU203

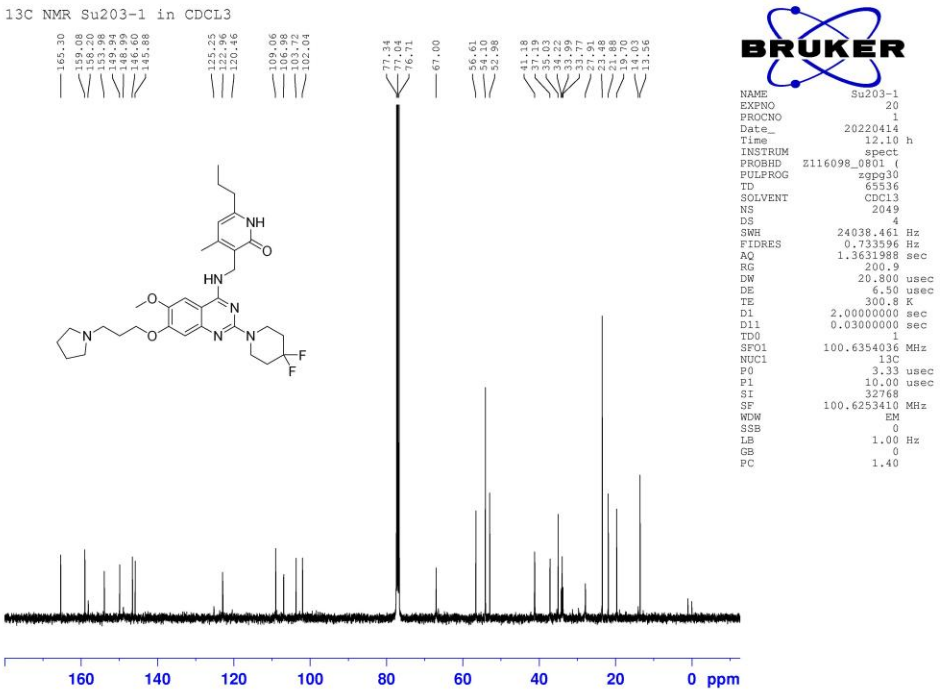

^{13}C NMR Spectrum of SU203

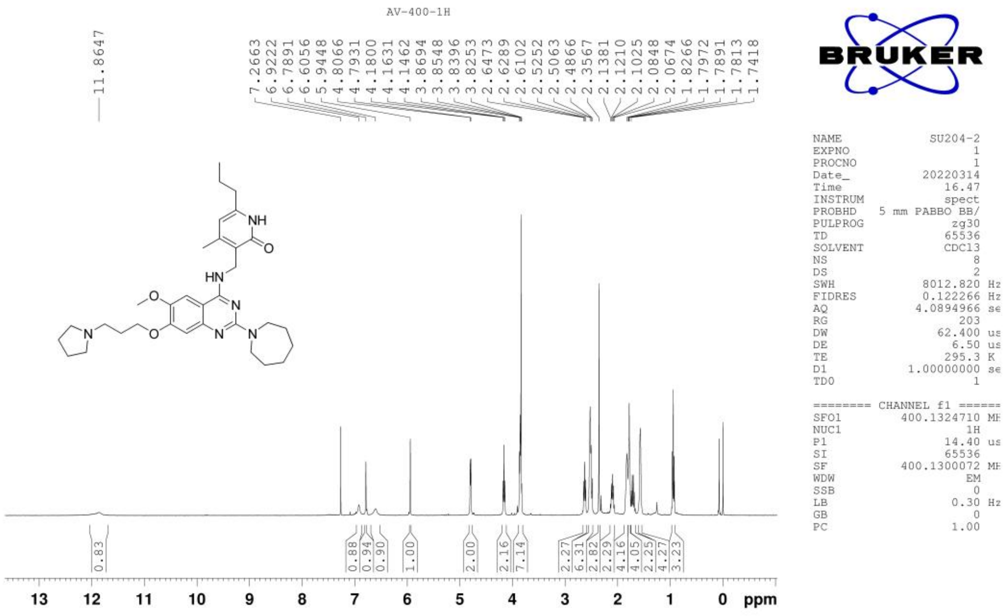

^{1}H NMR Spectrum of SU204

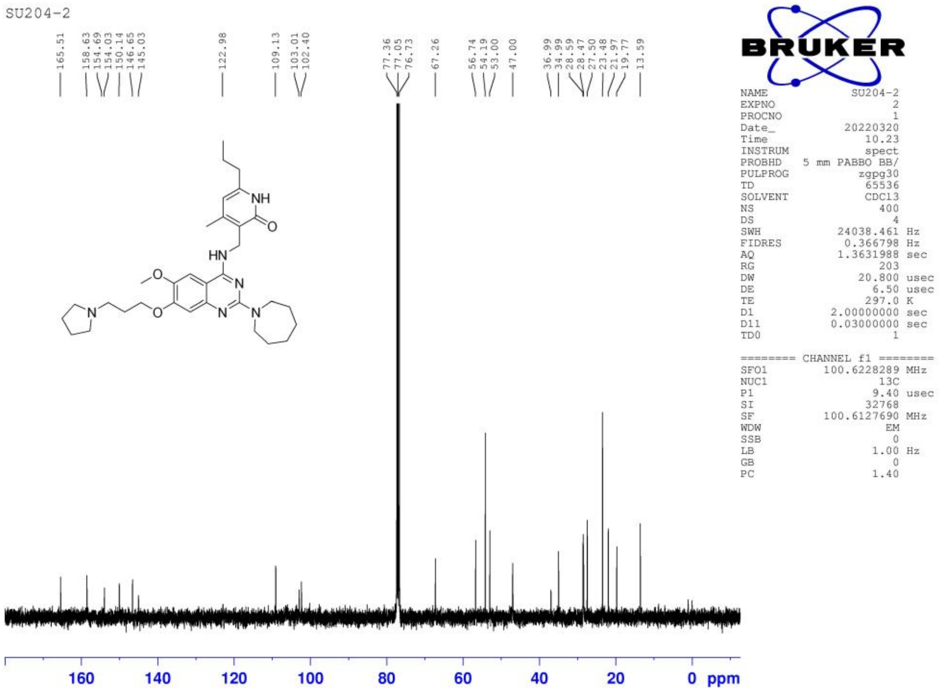

^{13}C NMR Spectrum of SU204

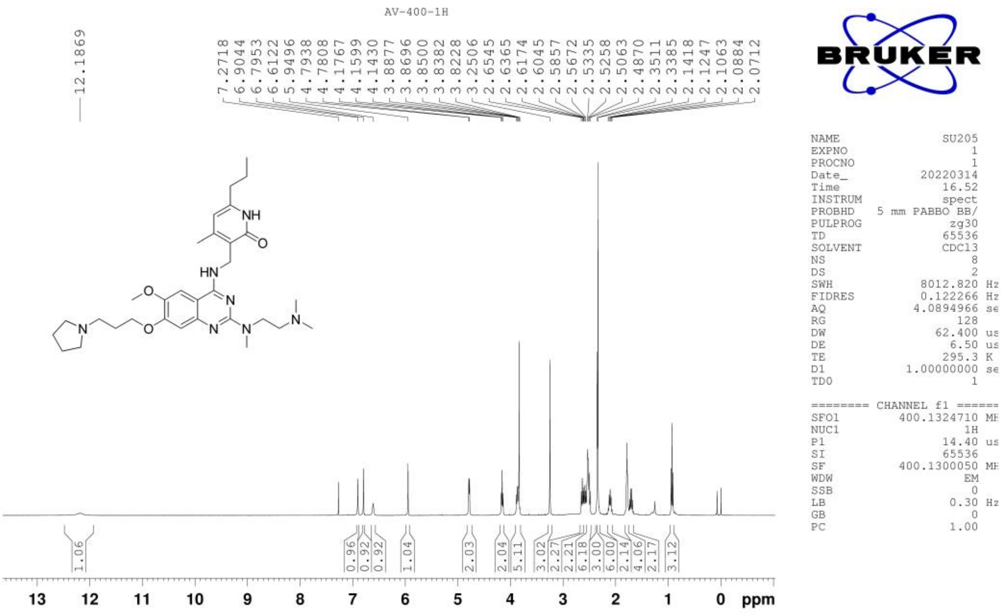

^{1}H NMR Spectrum of SU205

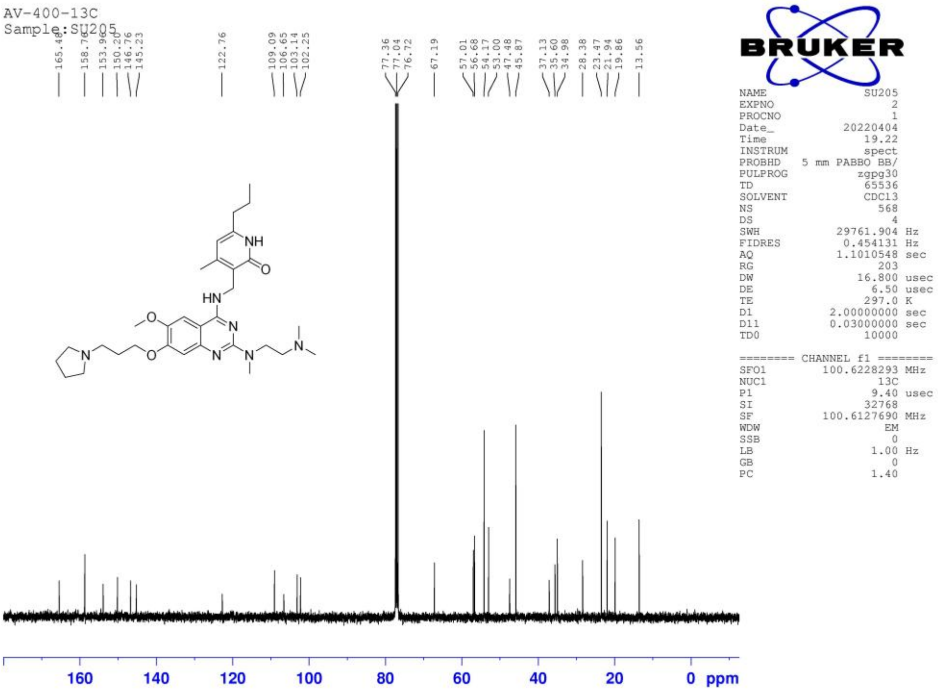

^{13}C NMR Spectrum of SU205

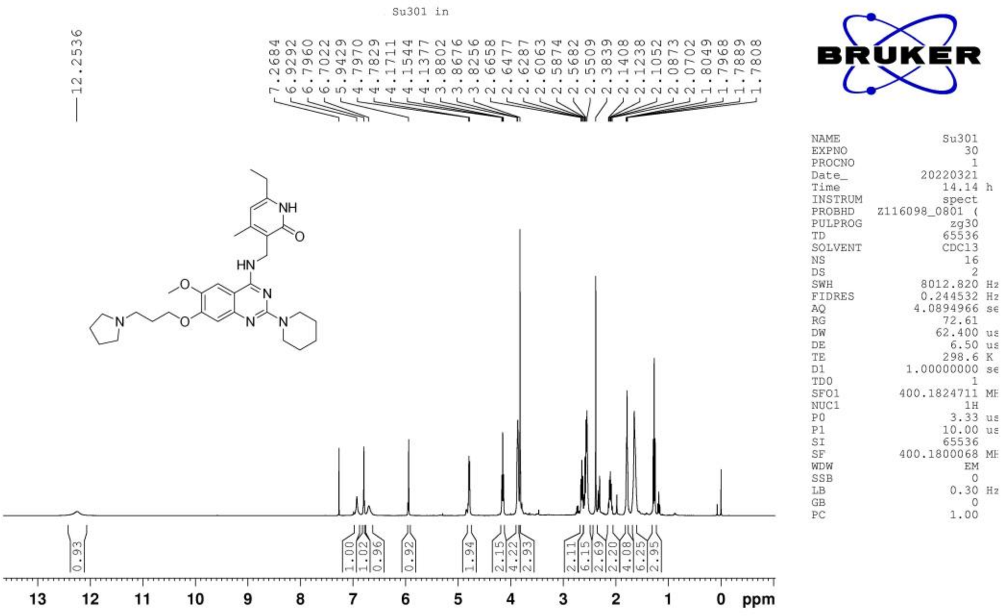

^{1}H NMR Spectrum of SU301

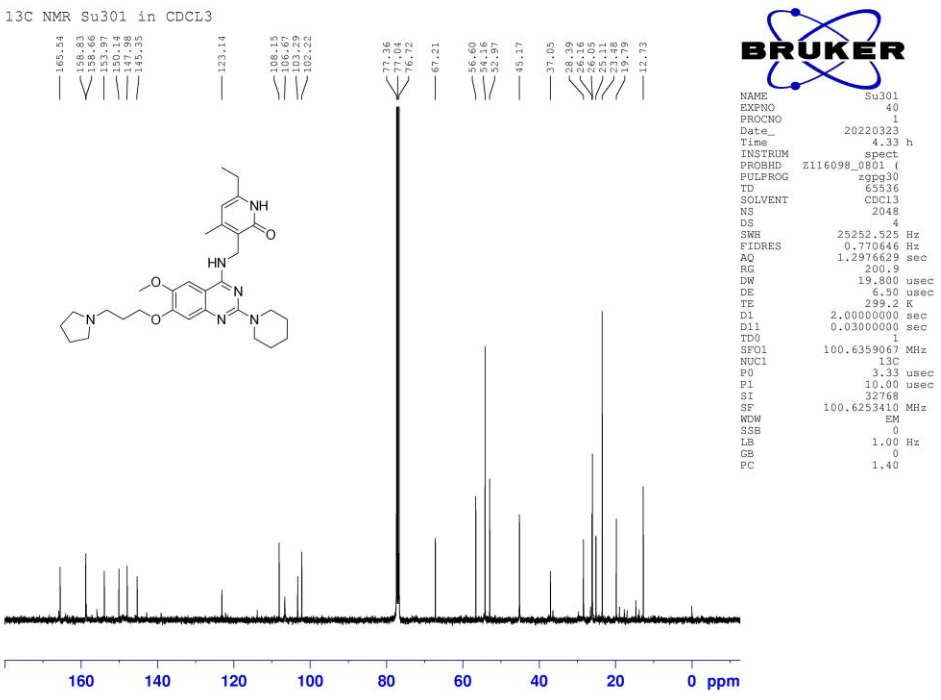

^{13}C NMR Spectrum of SU301

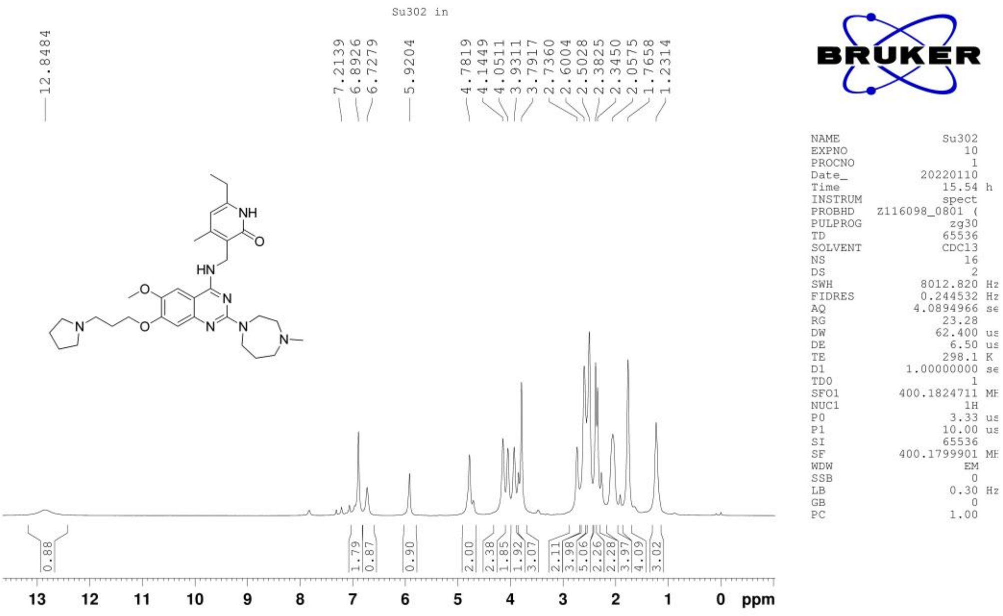

^{1}H NMR Spectrum of SU302

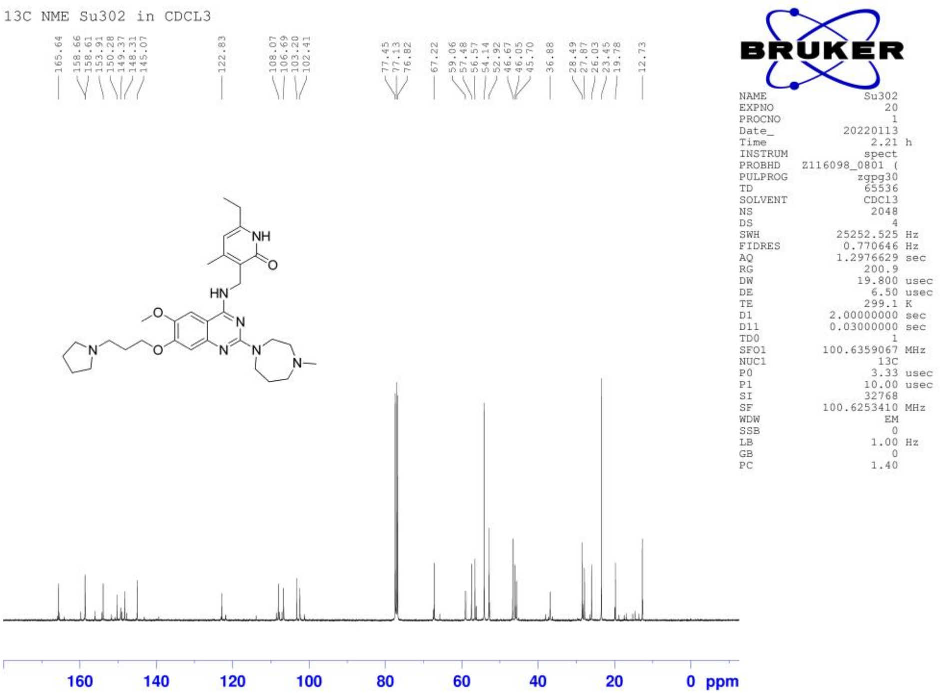

^{13}C NMR Spectrum of SU302

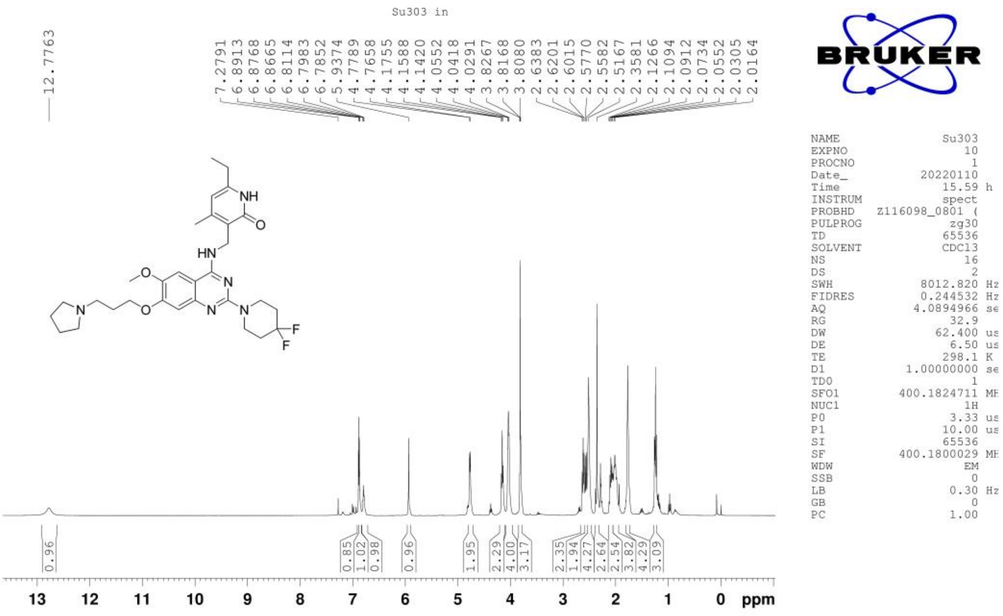

^{1}H NMR Spectrum of SU303

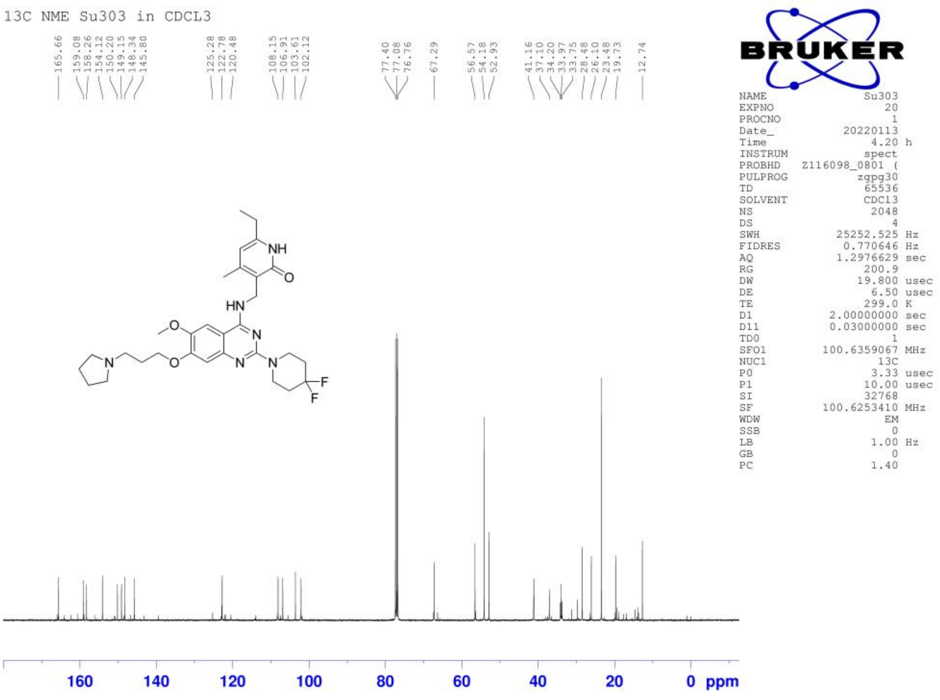

^{13}C NMR Spectrum of SU303

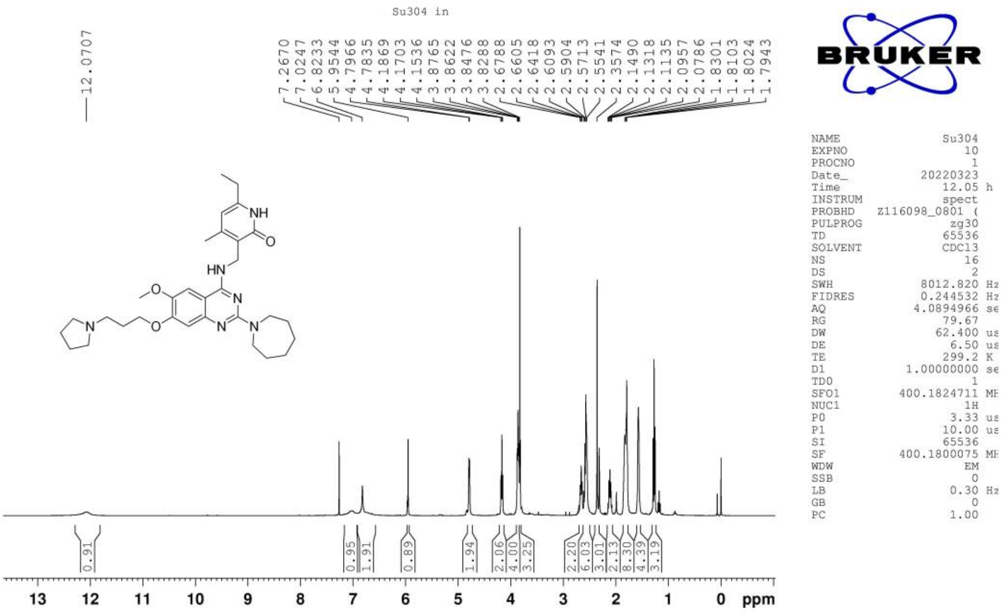

^{1}H NMR Spectrum of SU304

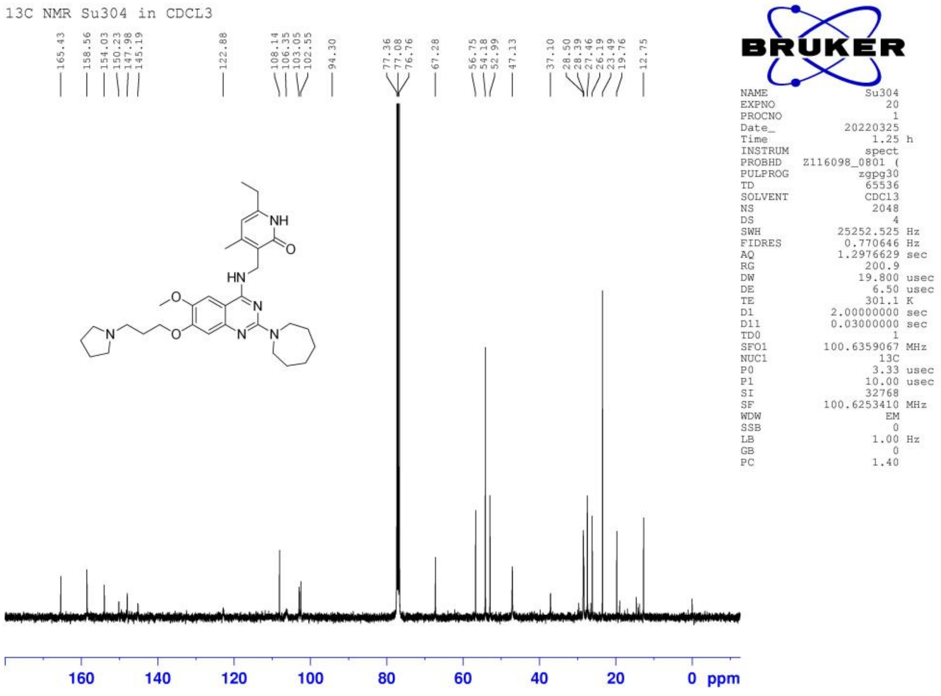

^{13}C NMR Spectrum of SU304

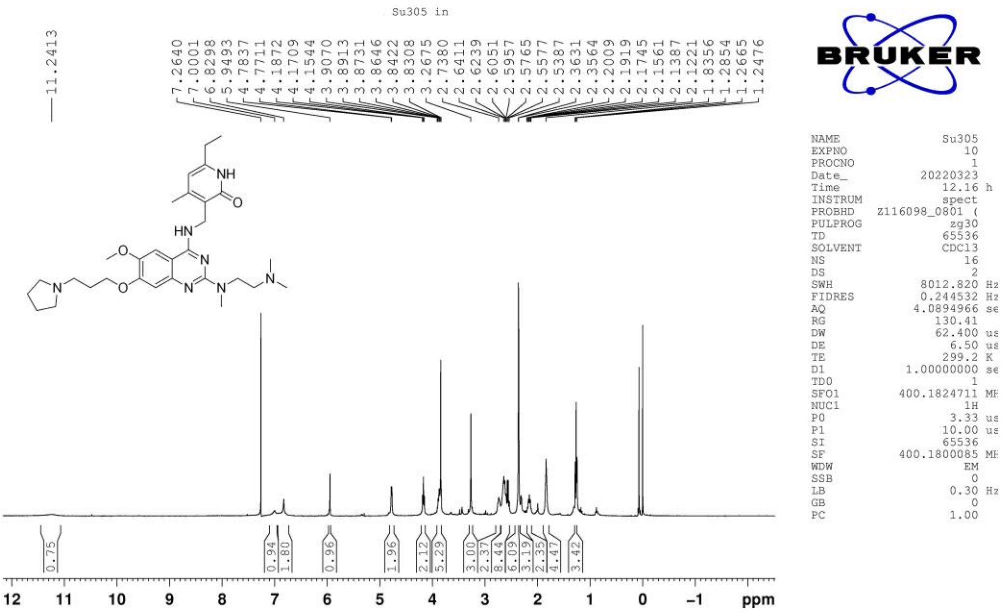

^{1}H NMR Spectrum of SU305

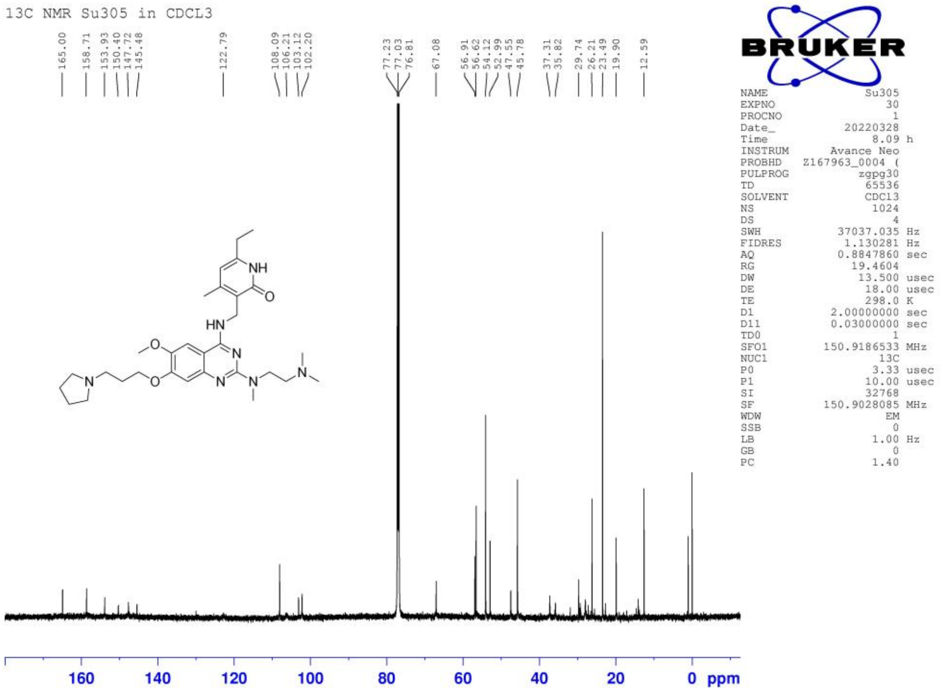

^{13}C NMR Spectrum of SU305

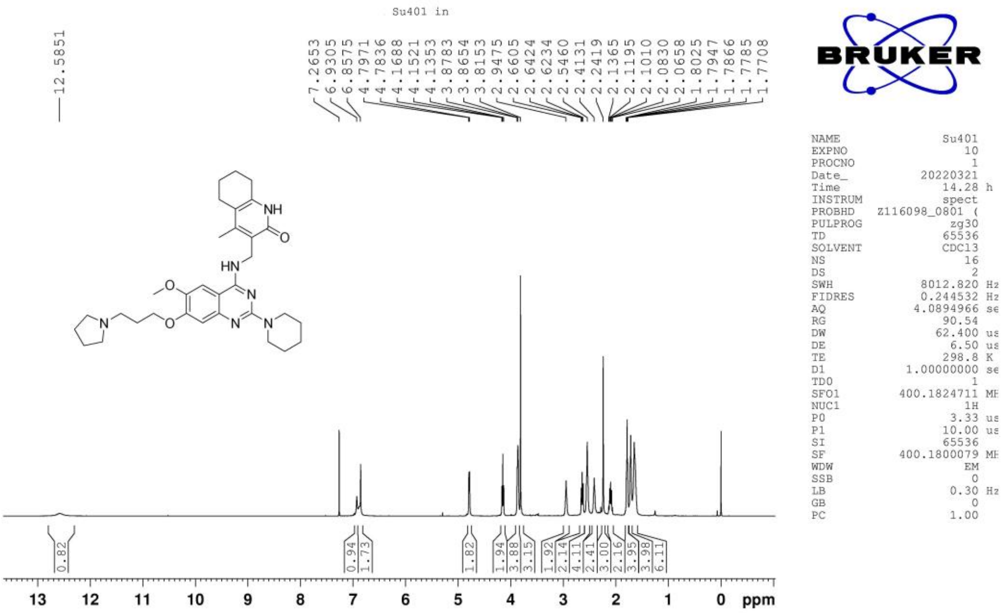

^{1}H NMR Spectrum of SU401

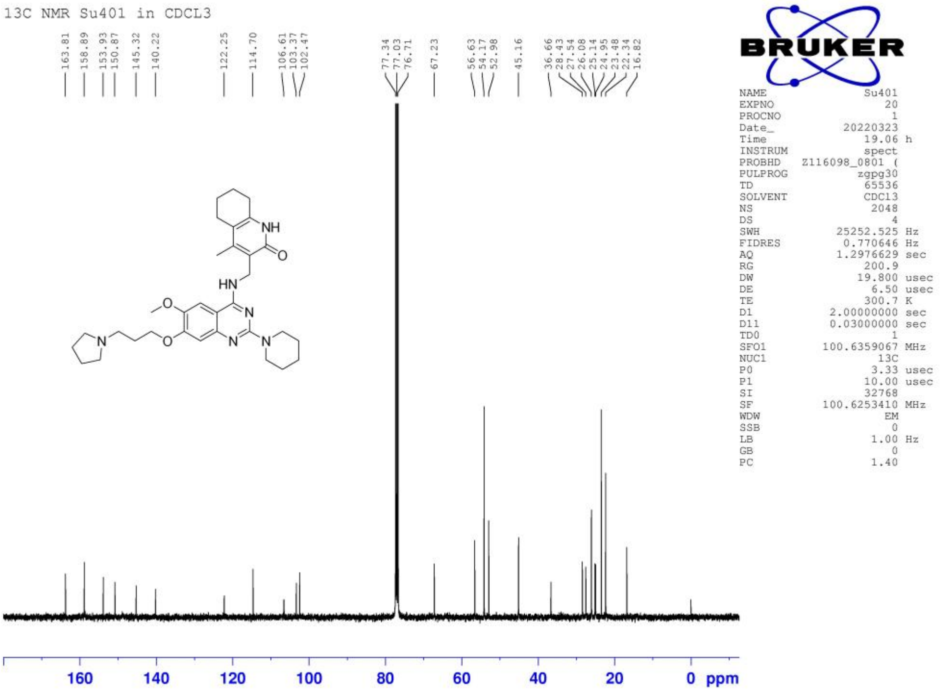

^{13}C NMR Spectrum of SU401

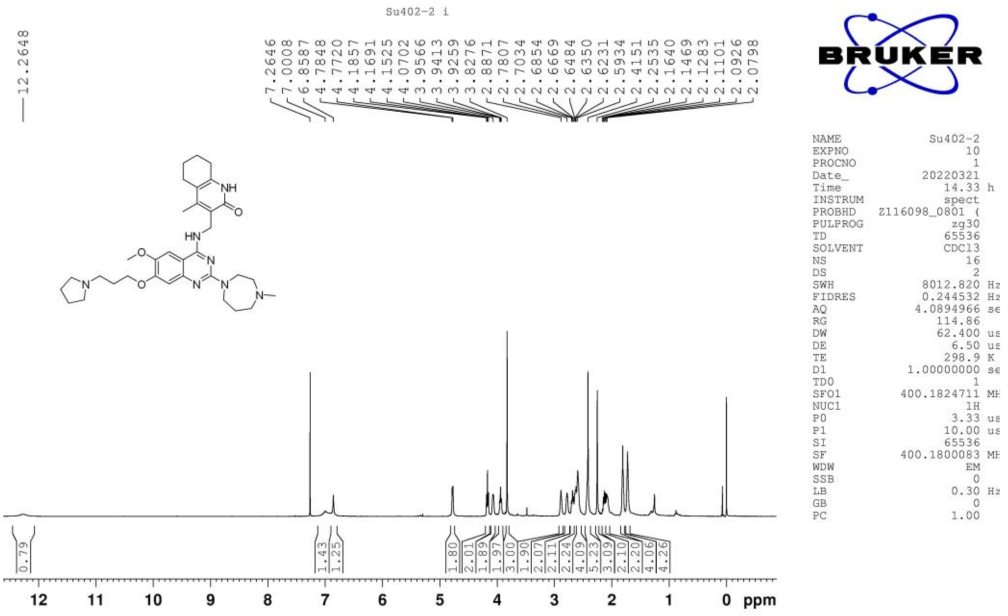

^{1}H NMR Spectrum of SU402

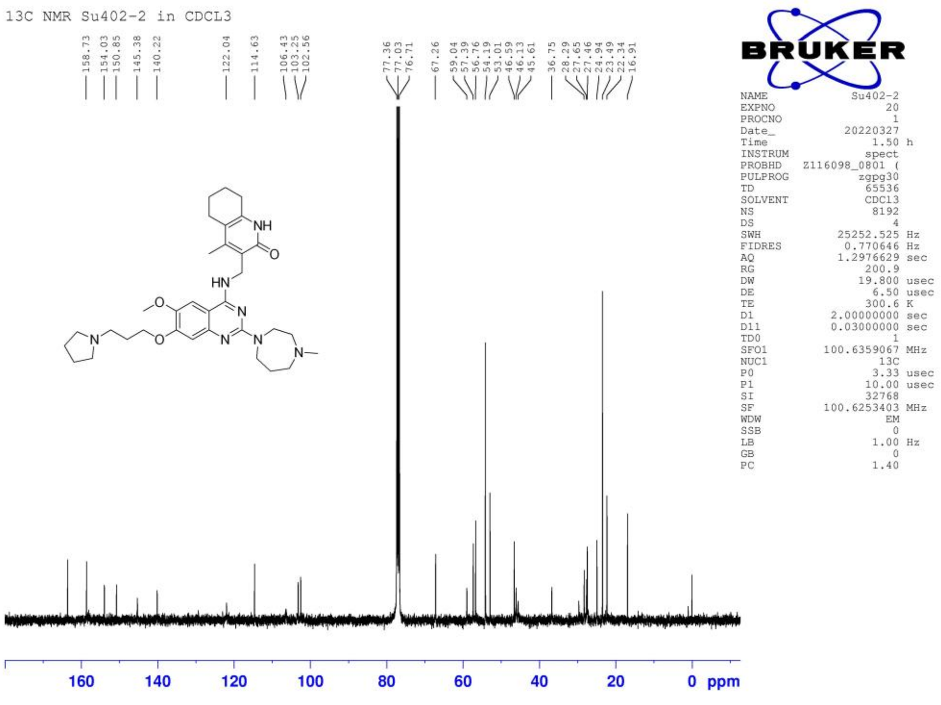

^{13}C NMR Spectrum of SU402

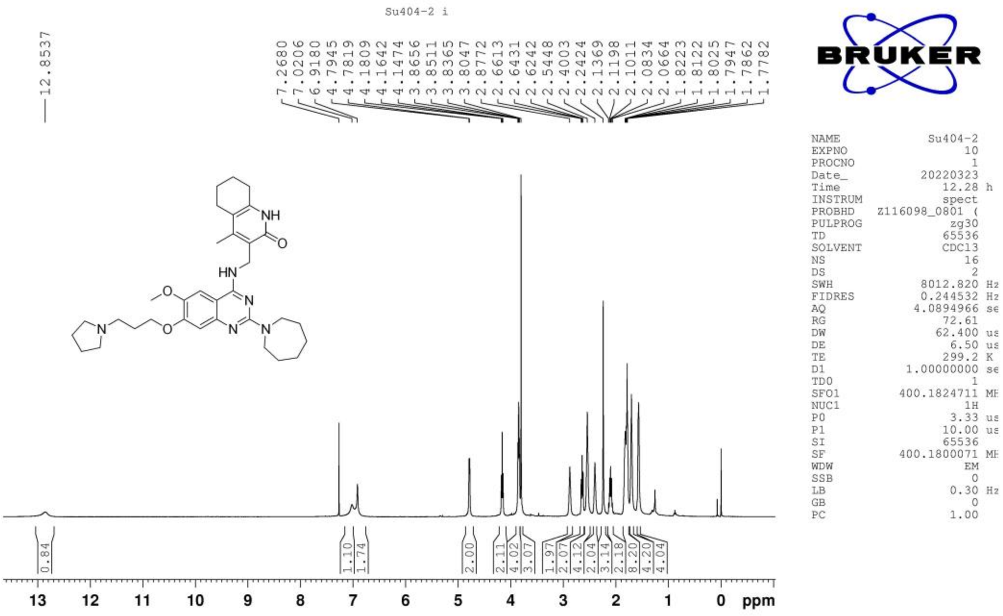

^{1}H NMR Spectrum of SU403

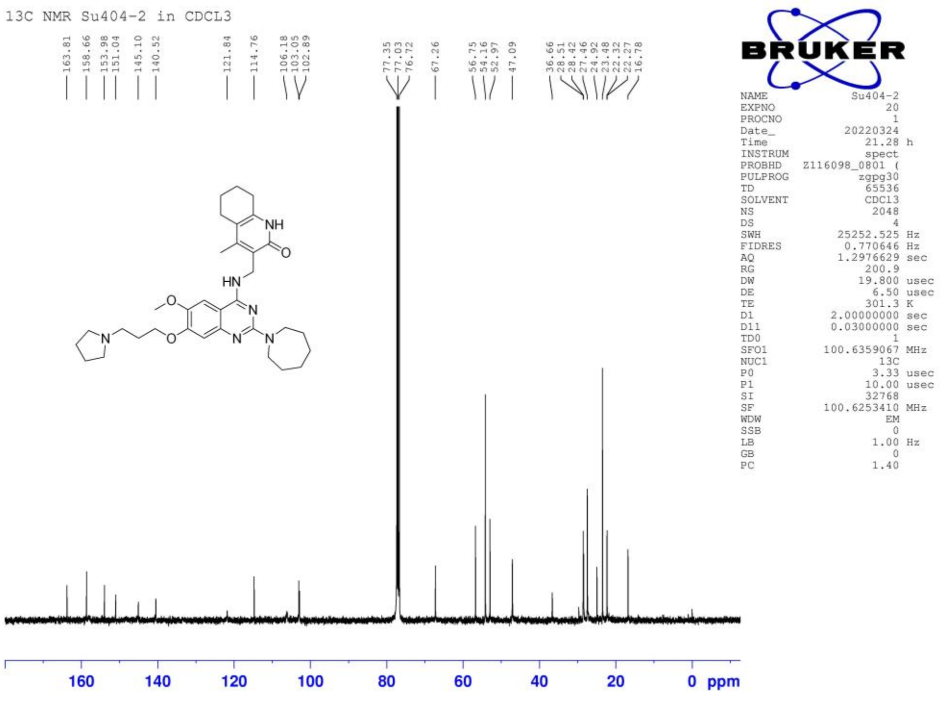

^{13}C NMR Spectrum of SU403

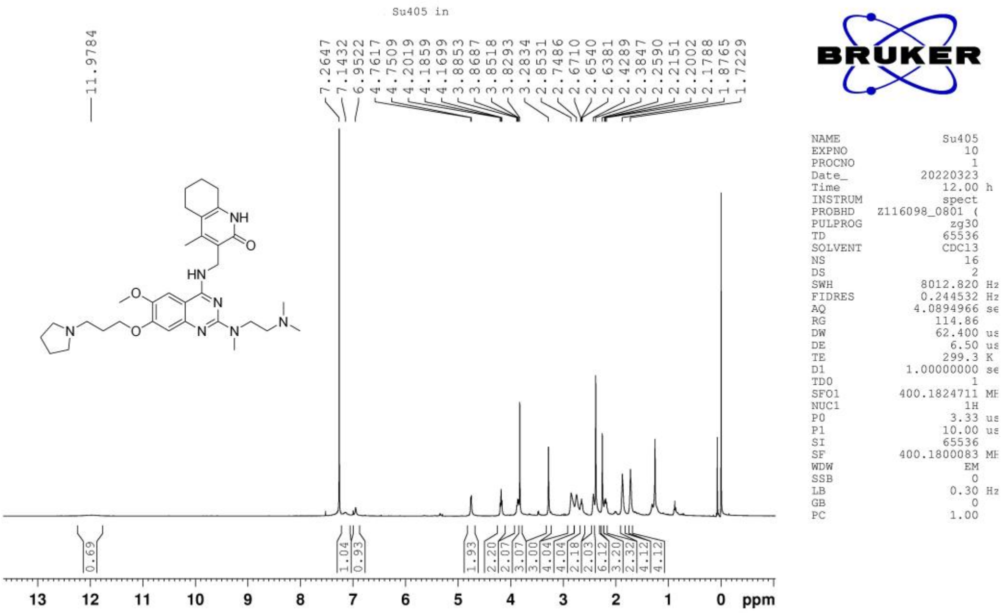

^{1}H NMR Spectrum of SU404

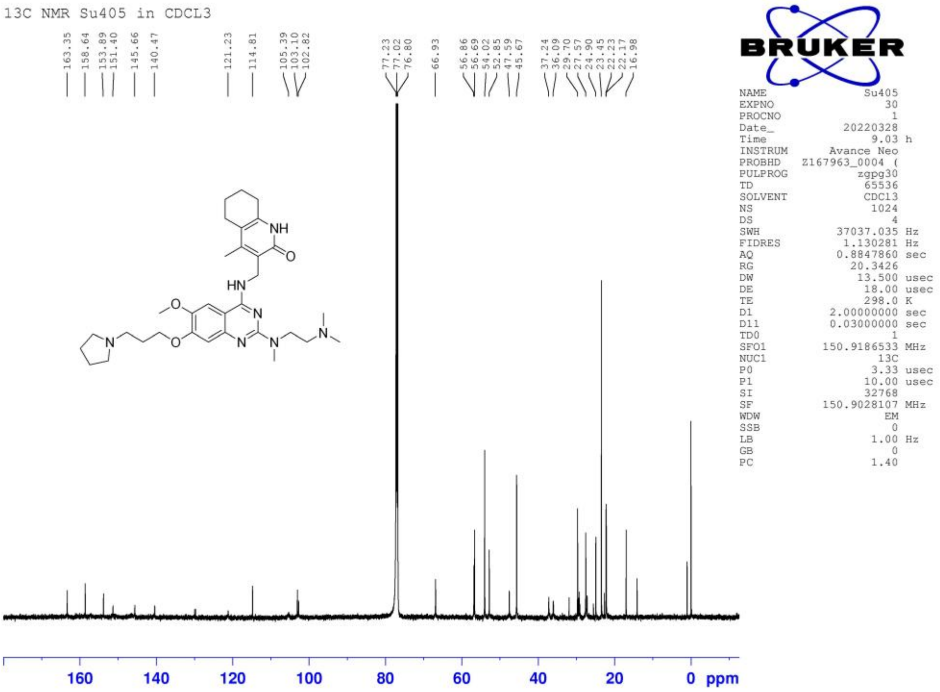

^{13}C NMR Spectrum of SU404

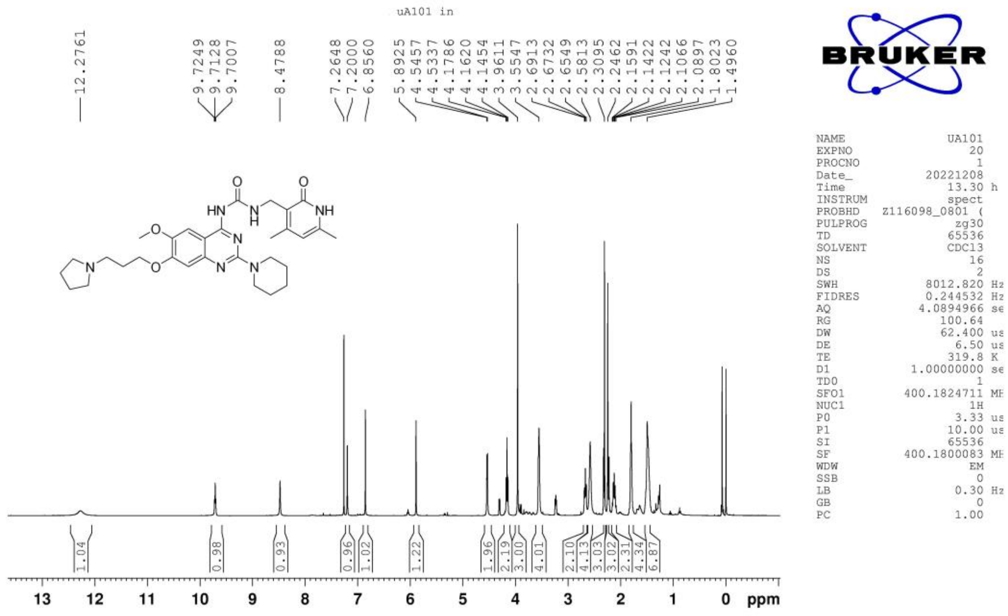

^{1}H NMR Spectrum of UA01

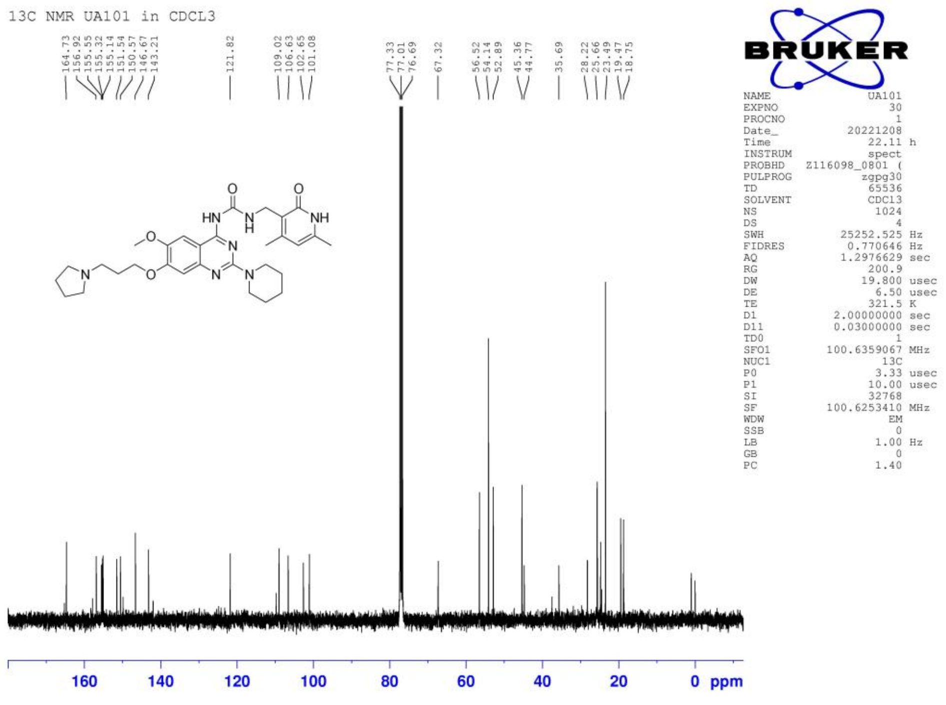

^{13}C NMR Spectrum of UA01

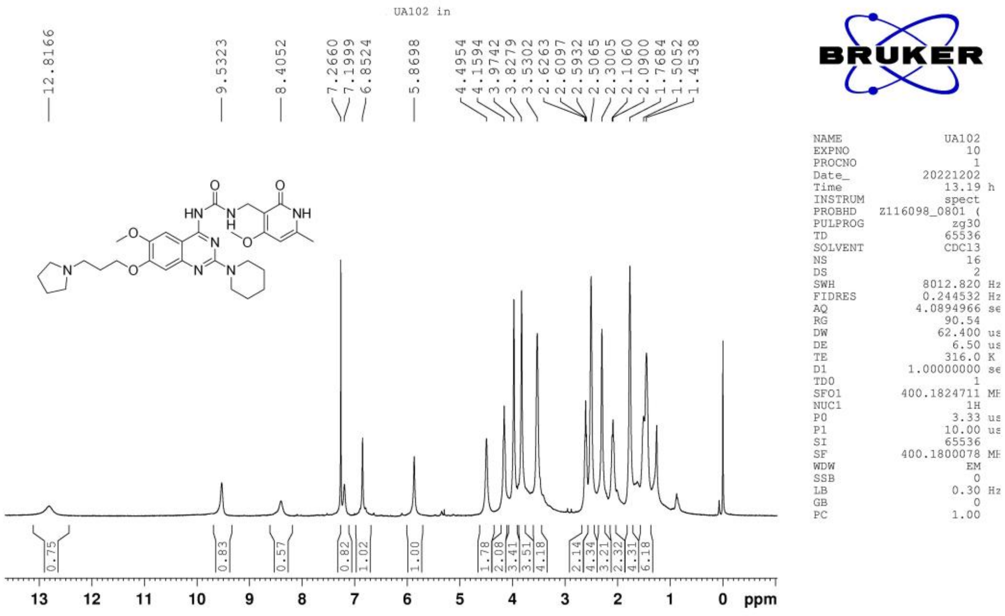

^{1}H NMR Spectrum of UA02

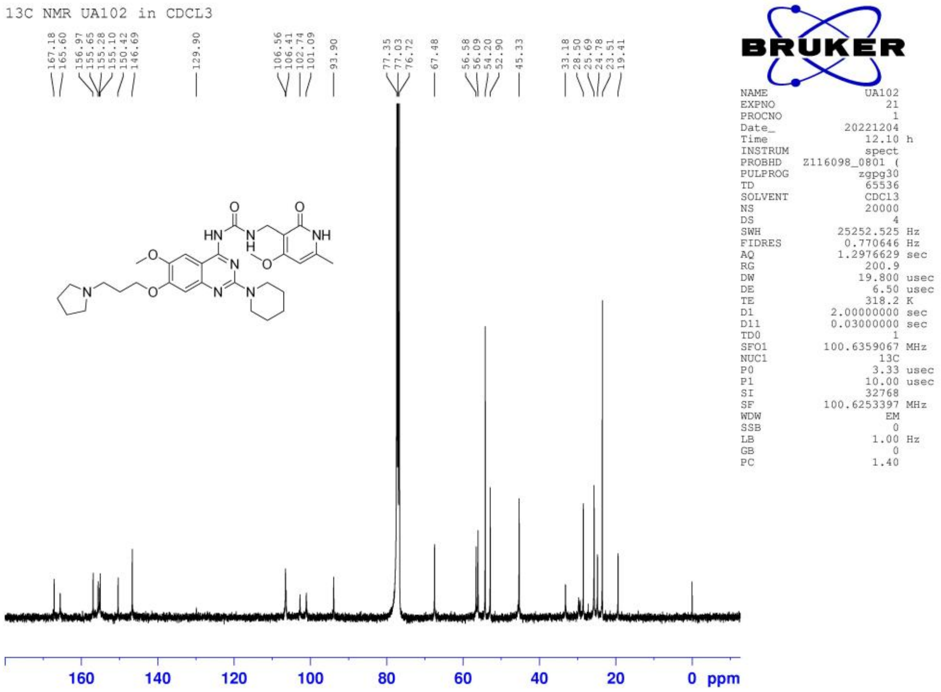

^{13}C NMR Spectrum of UA02

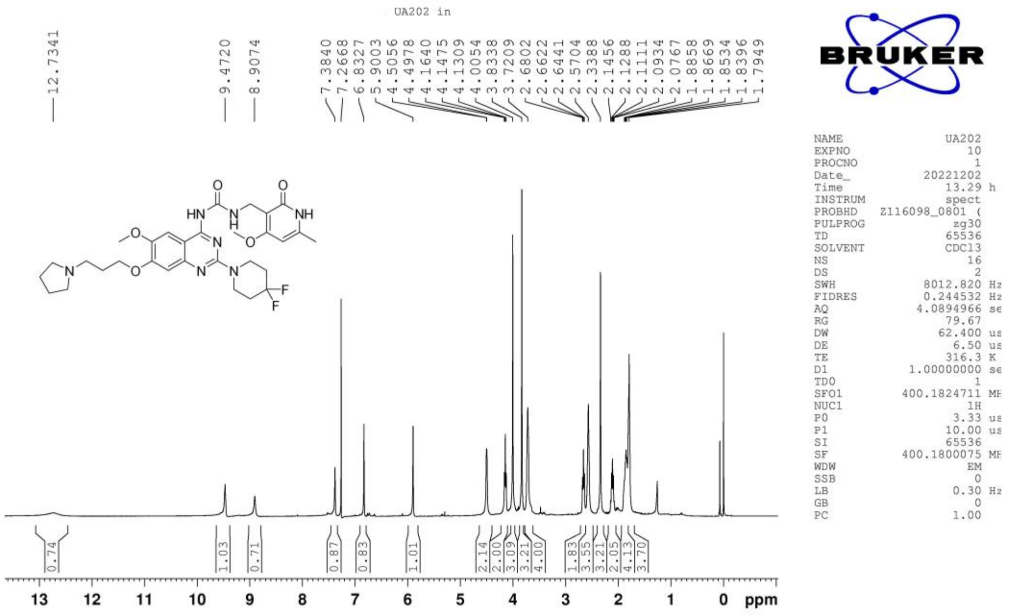

^{1}H NMR Spectrum of UA03

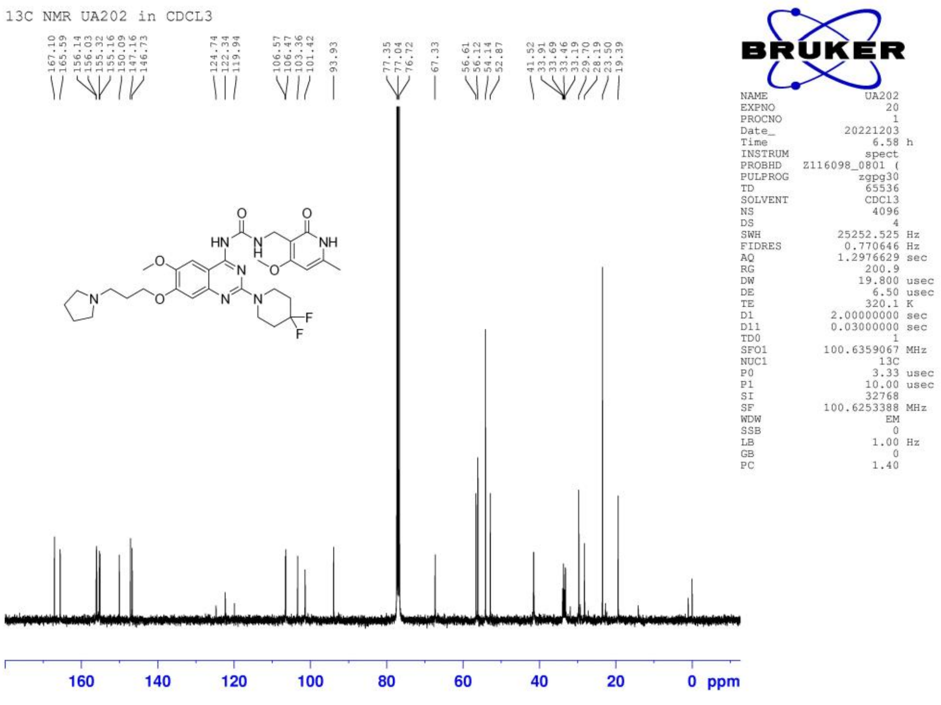

^{13}C NMR Spectrum of UA03

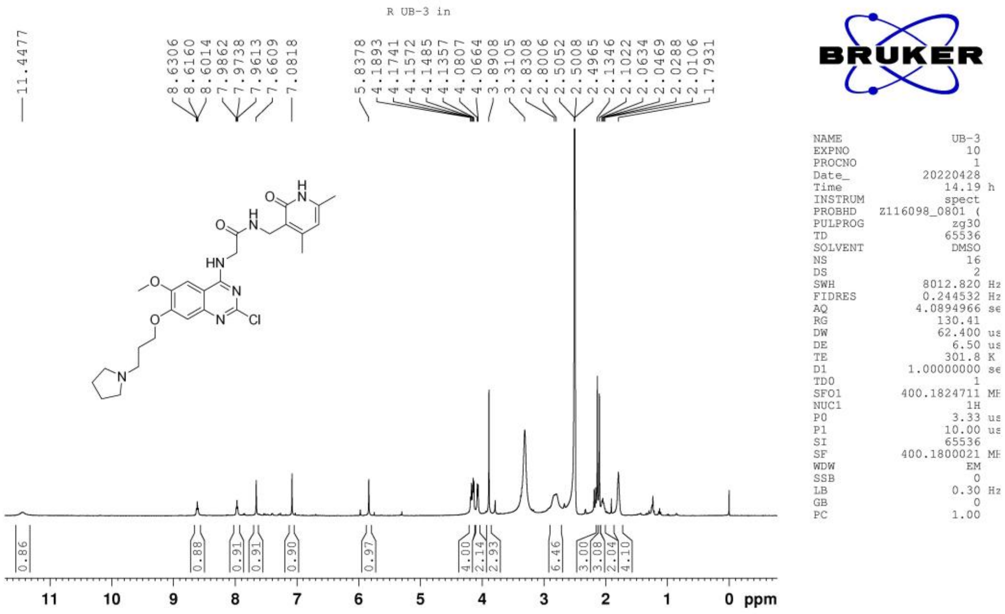

^{1}H NMR Spectrum of 17

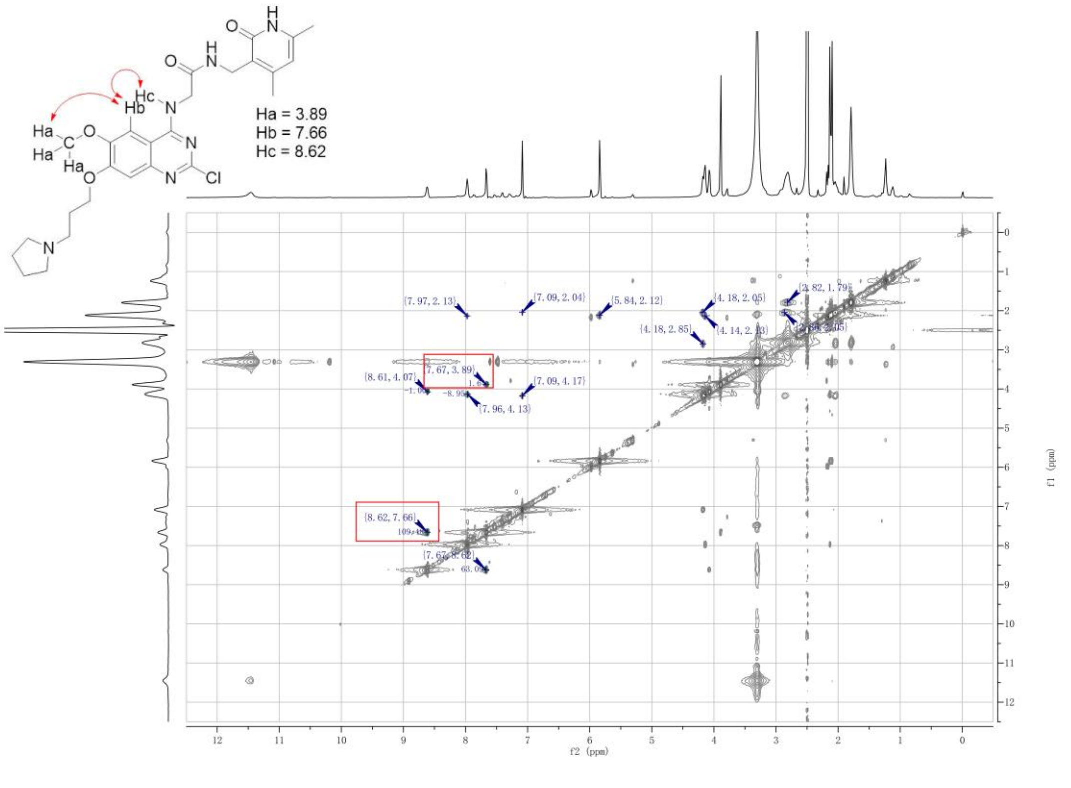

^{1}H-^{1}H NOESY Spectrum of 17

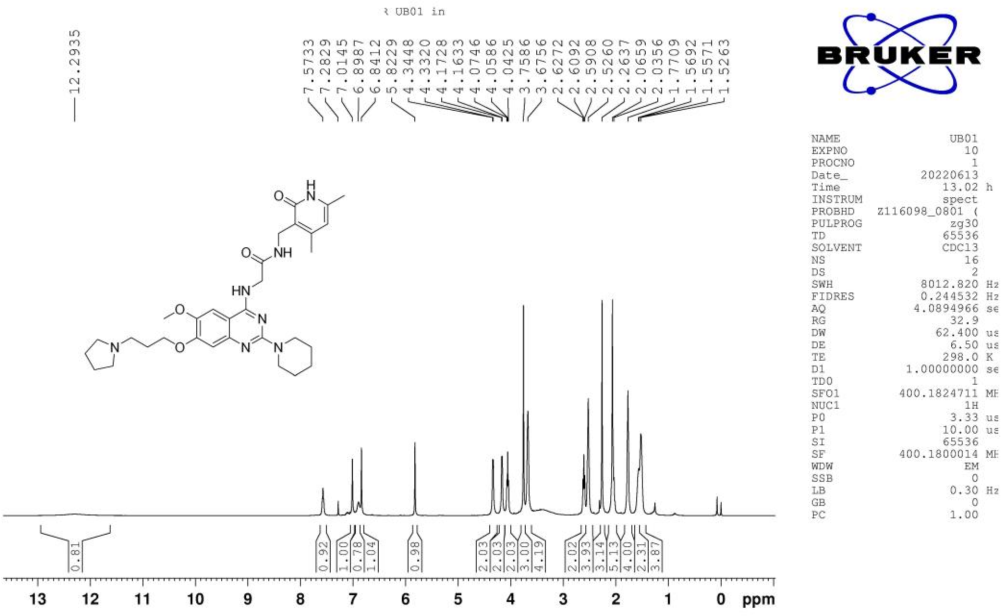

^{1}H NMR Spectrum of UB01

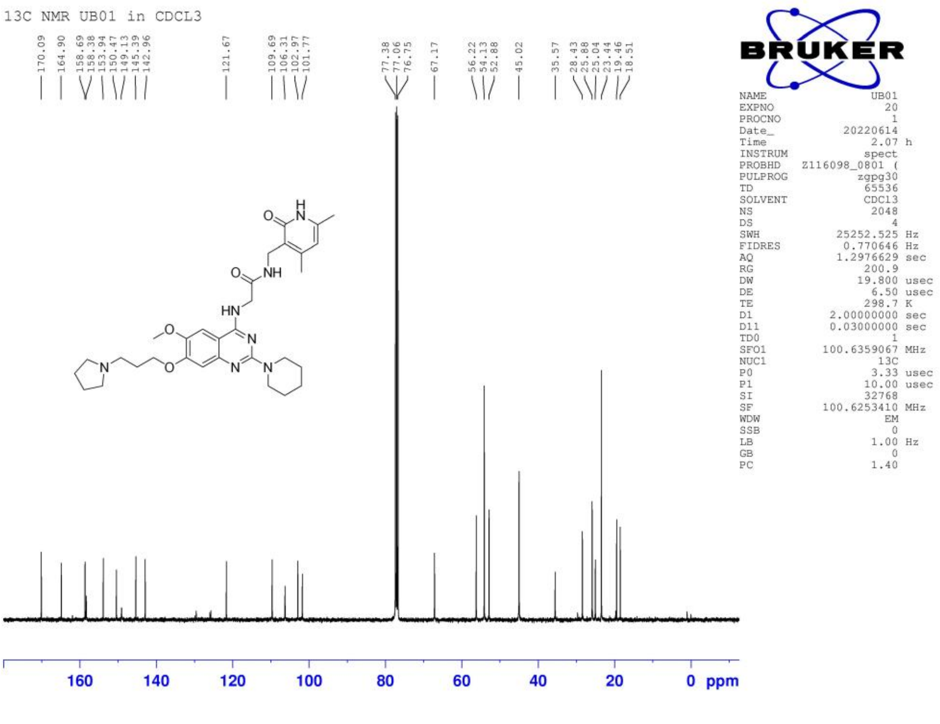

^{13}C NMR Spectrum of UB01

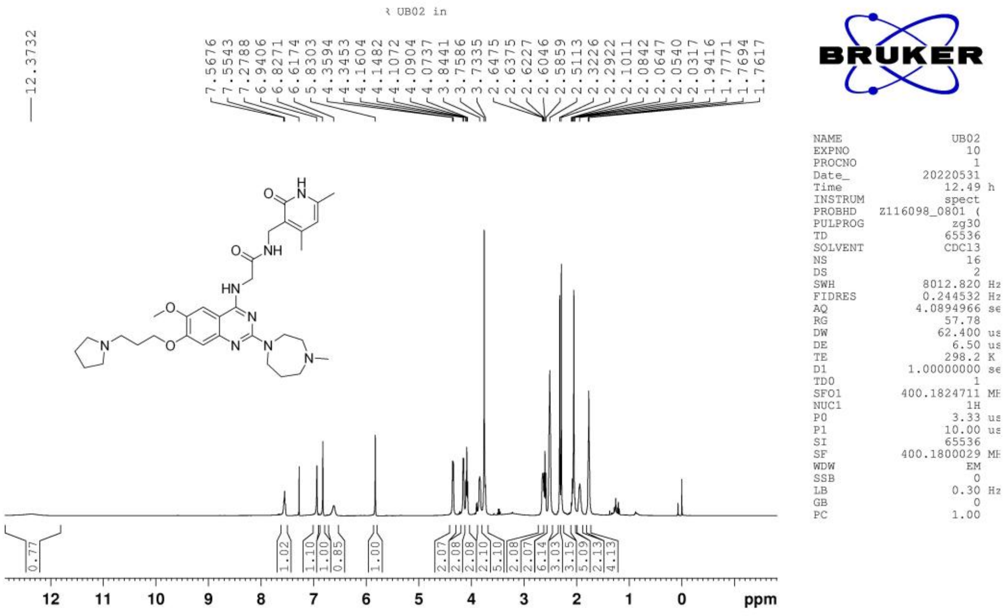

^{1}H NMR Spectrum of UB02

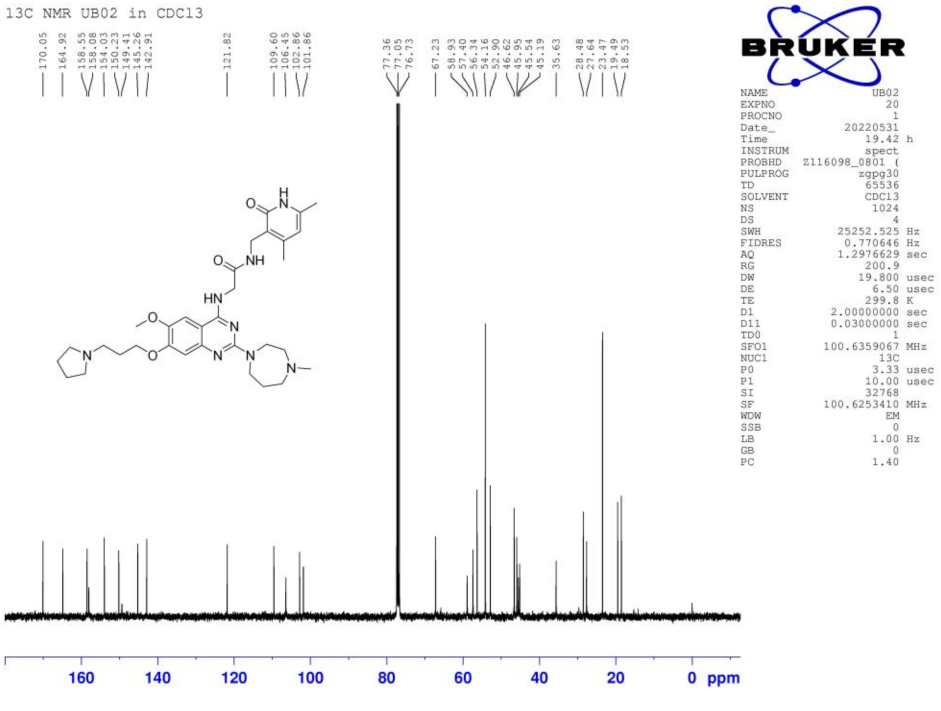

^{13}C NMR Spectrum of UB02

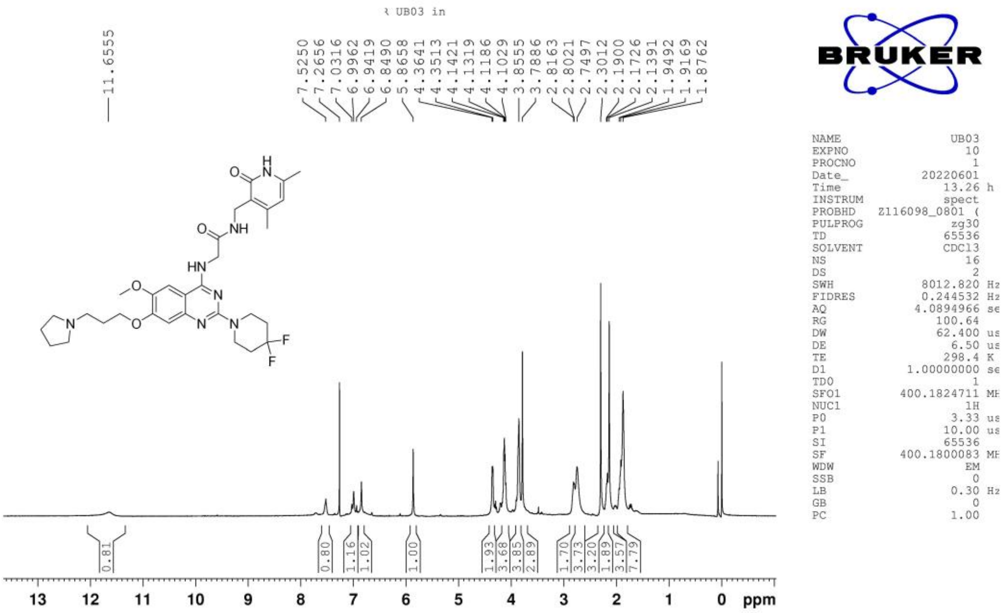

^{1}H NMR Spectrum of UB03

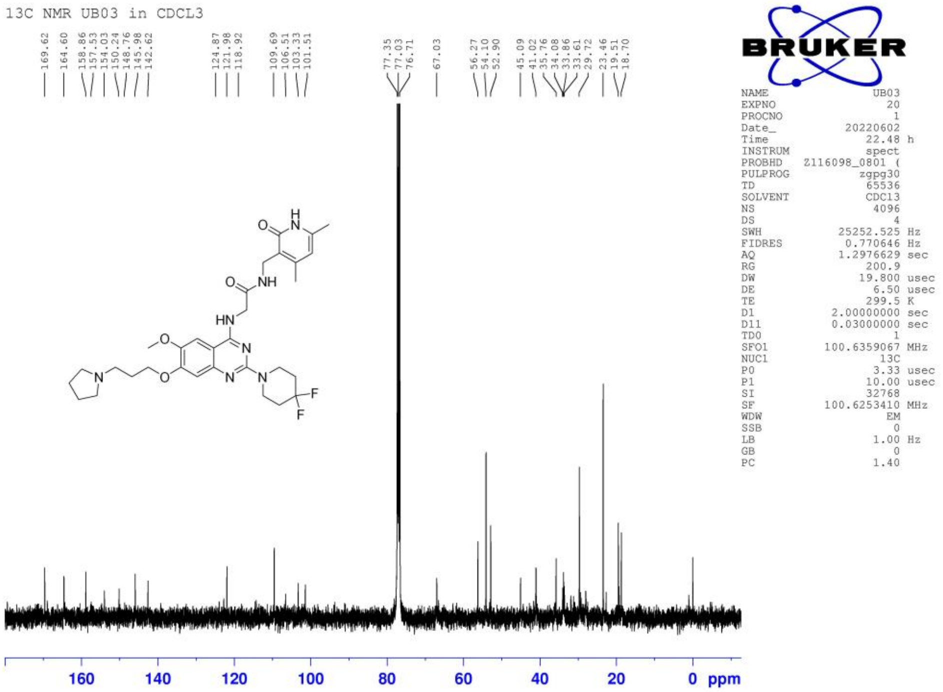

^{13}C NMR Spectrum of UB03

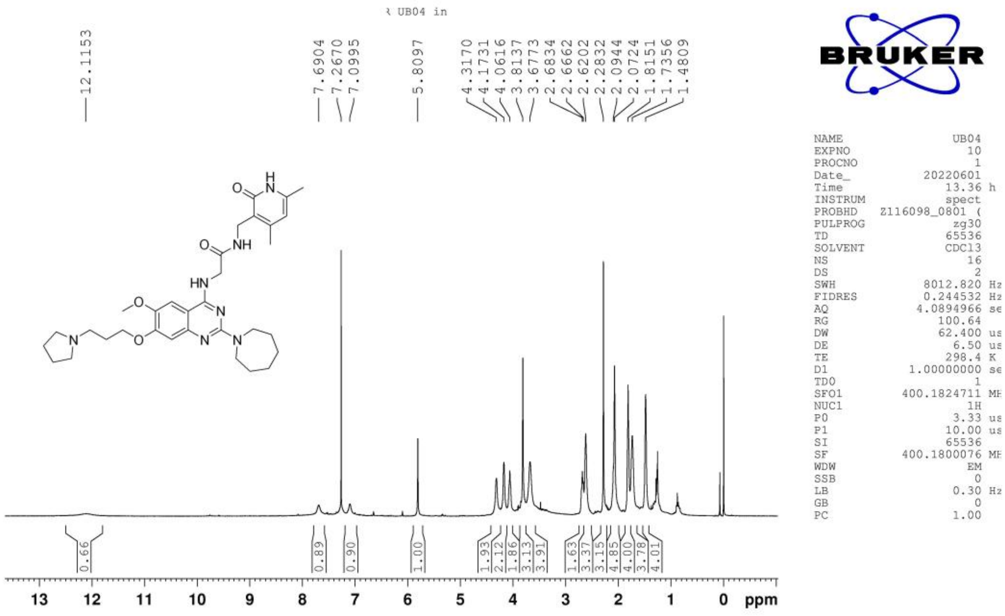

^{1}H NMR Spectrum of UB04

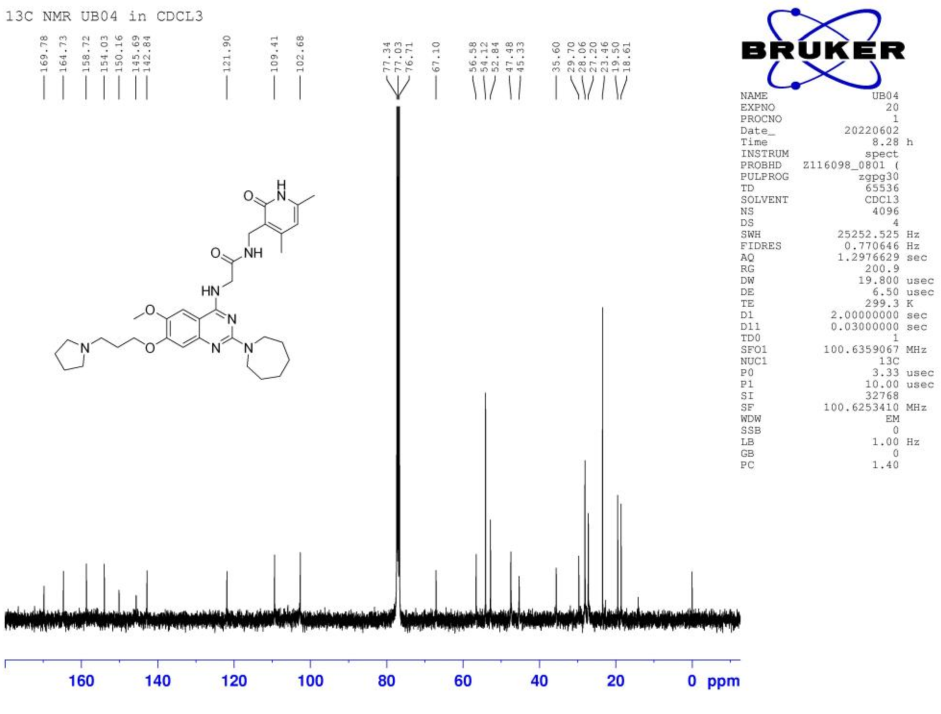

^{13}C NMR Spectrum of UB04

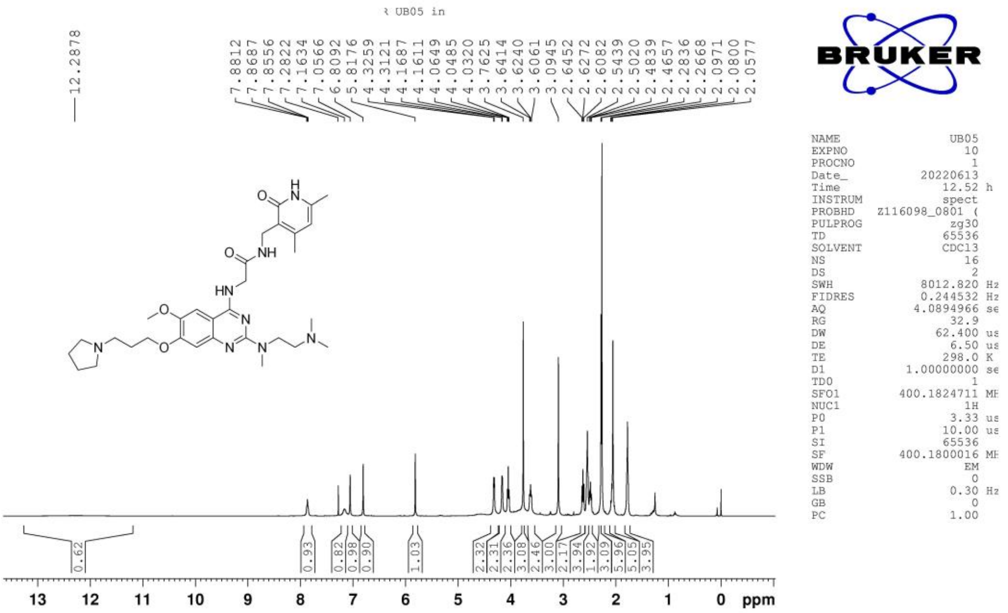

^{1}H NMR Spectrum of UB05

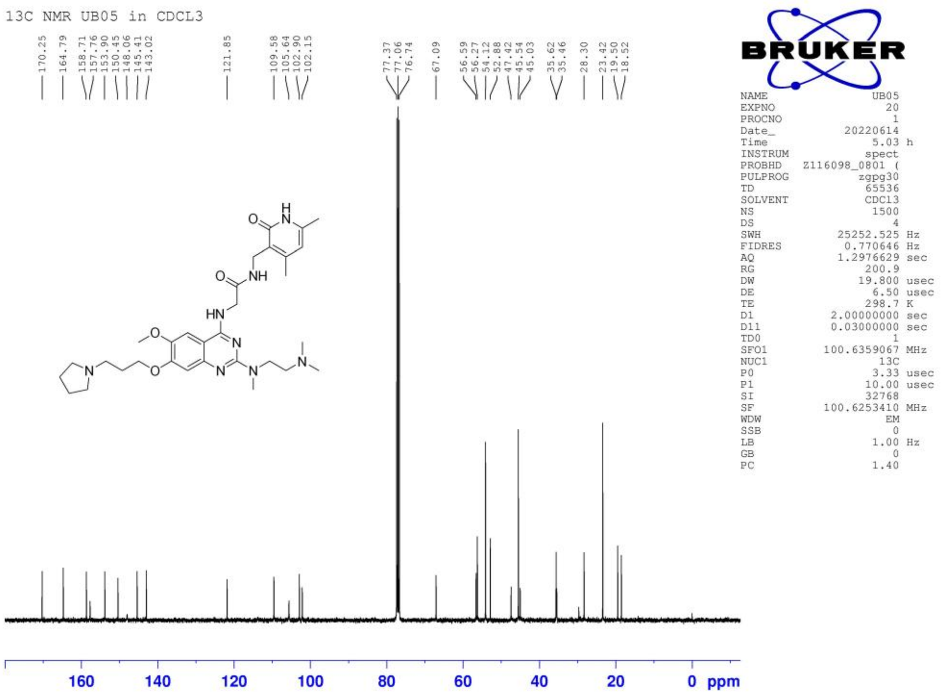

^{13}C NMR Spectrum of UB05

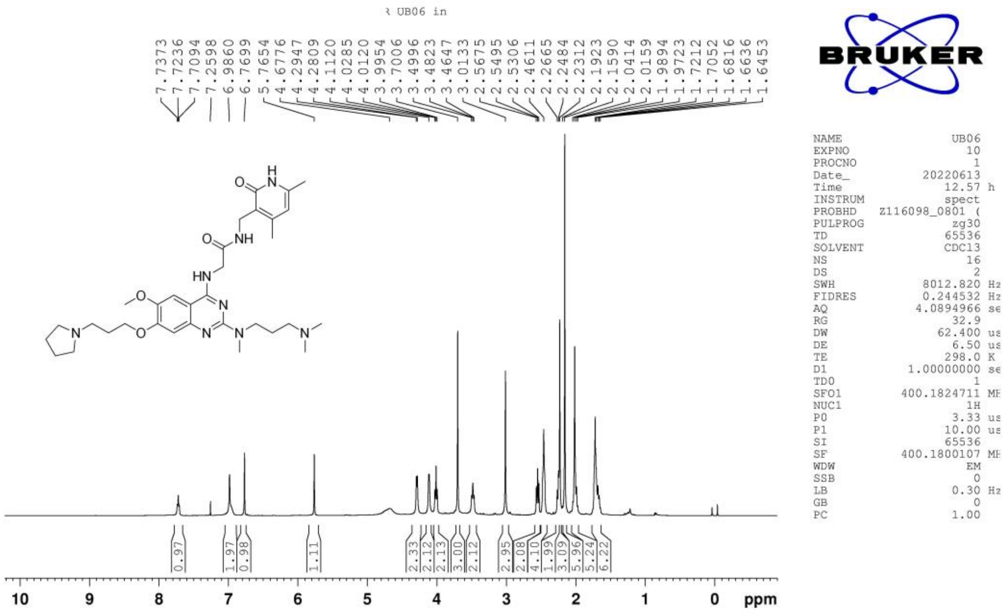

^{1}H NMR Spectrum of UB06

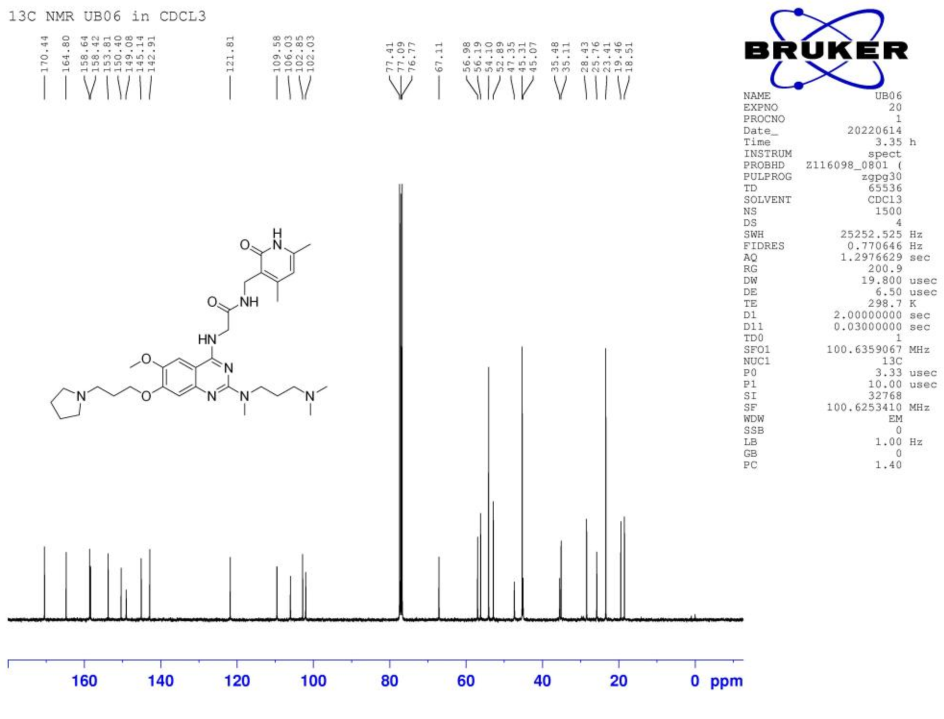

^{13}C NMR Spectrum of UB06

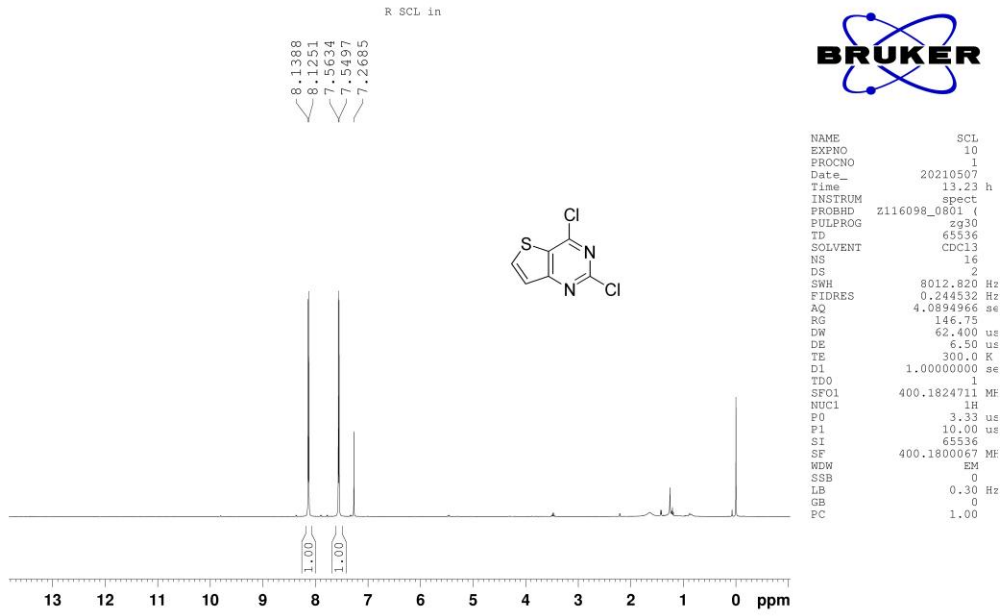

^{1}H NMR Spectrum of 20

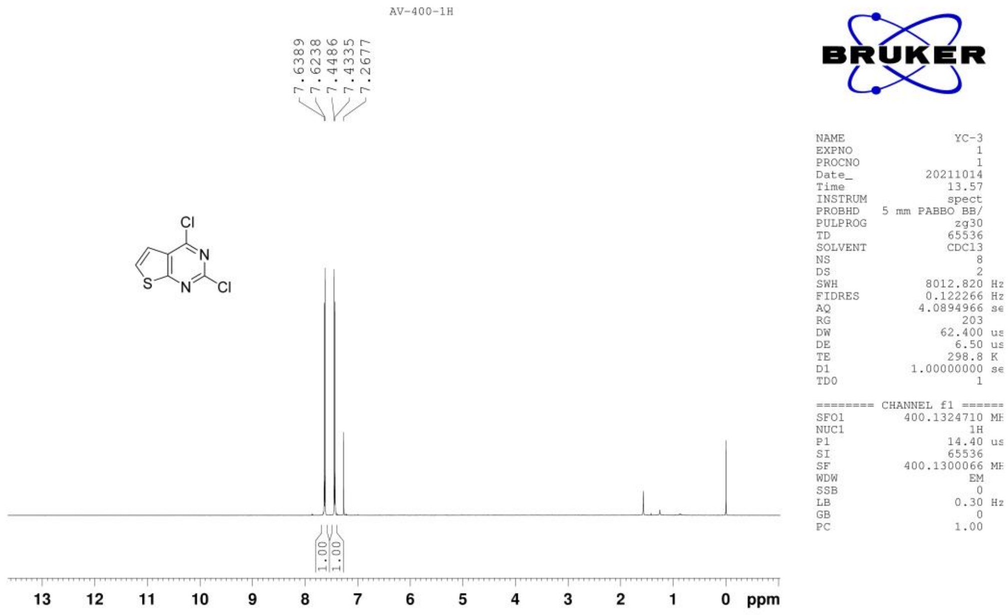

^{1}H NMR Spectrum of 24

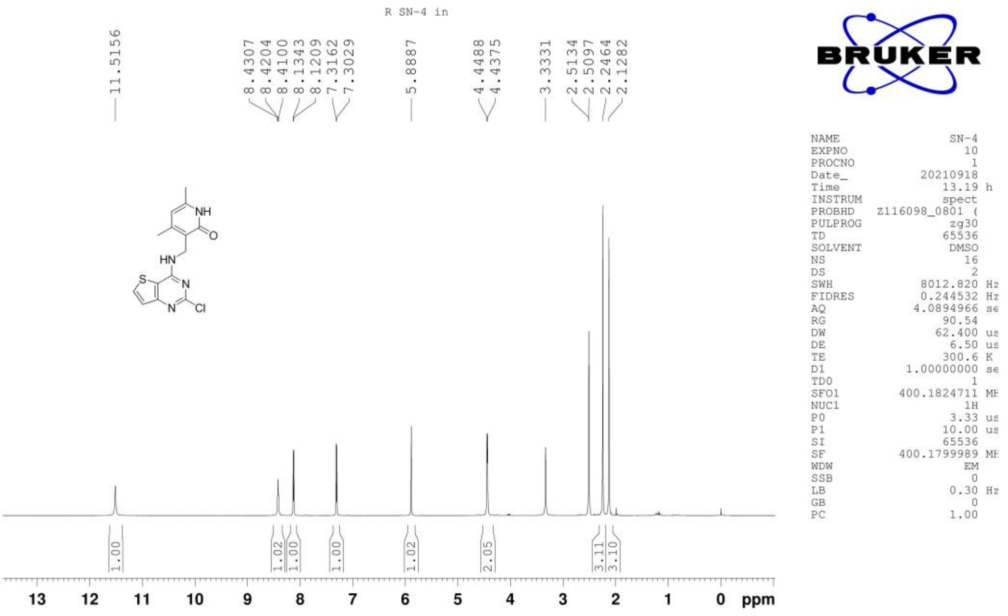

^{1}H NMR Spectrum of 21

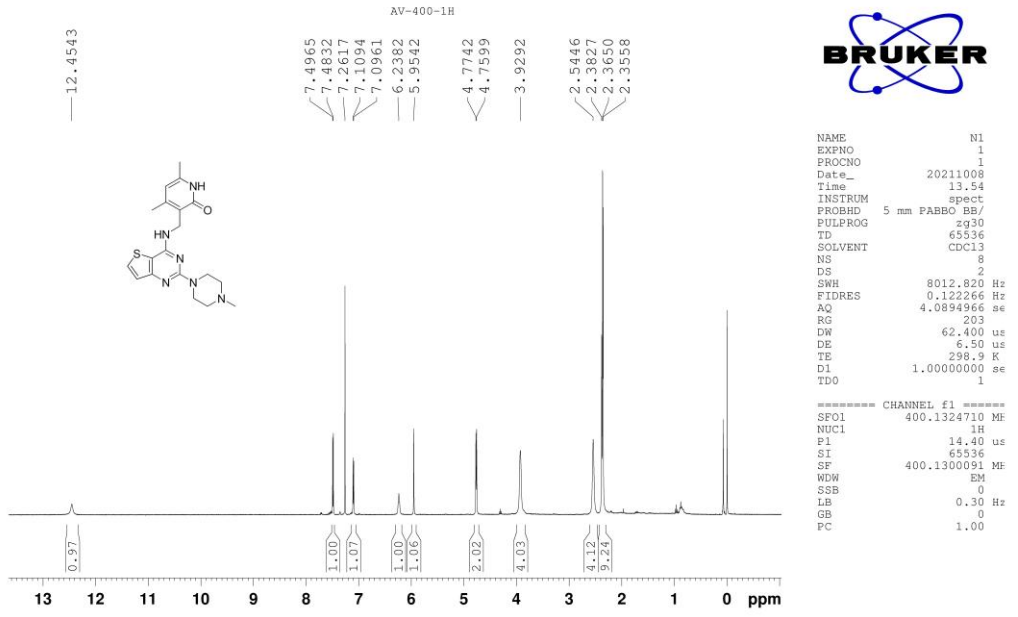

^{1}H NMR Spectrum of N1

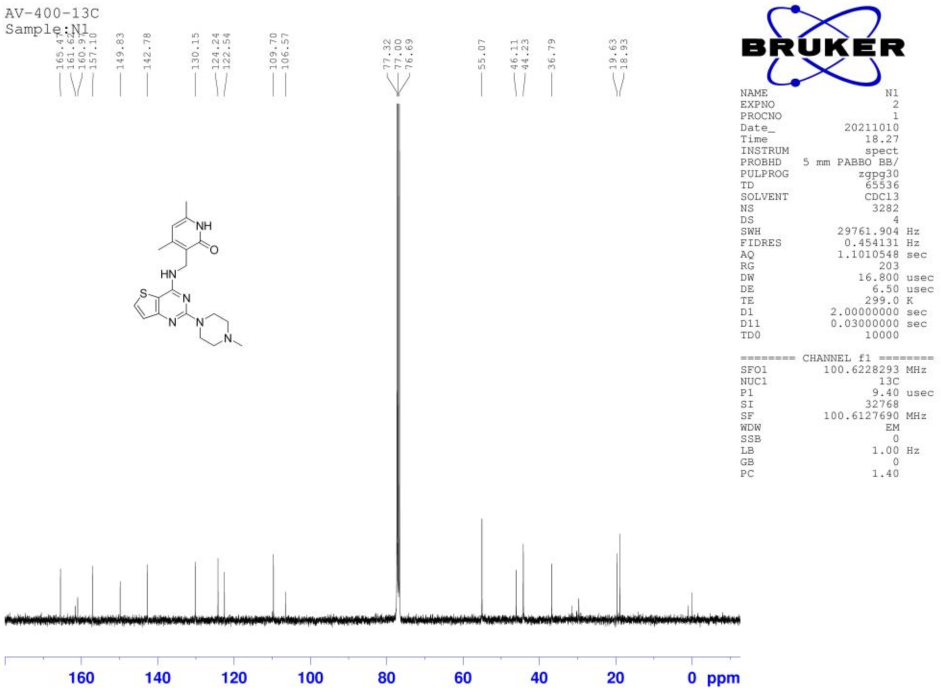

^{13}C NMR Spectrum of N1

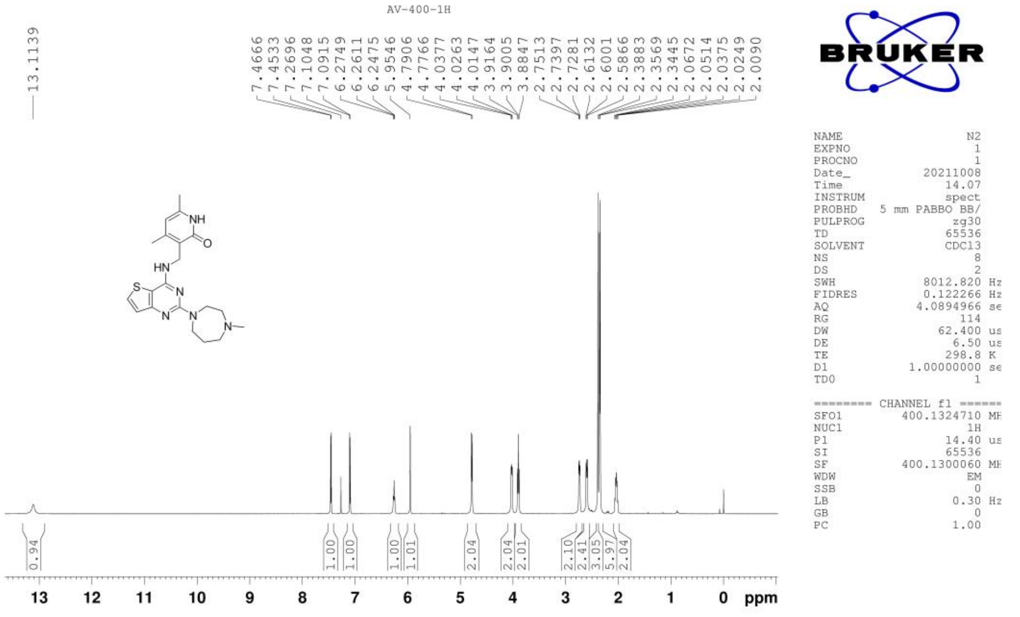

^{1}H NMR Spectrum of N2

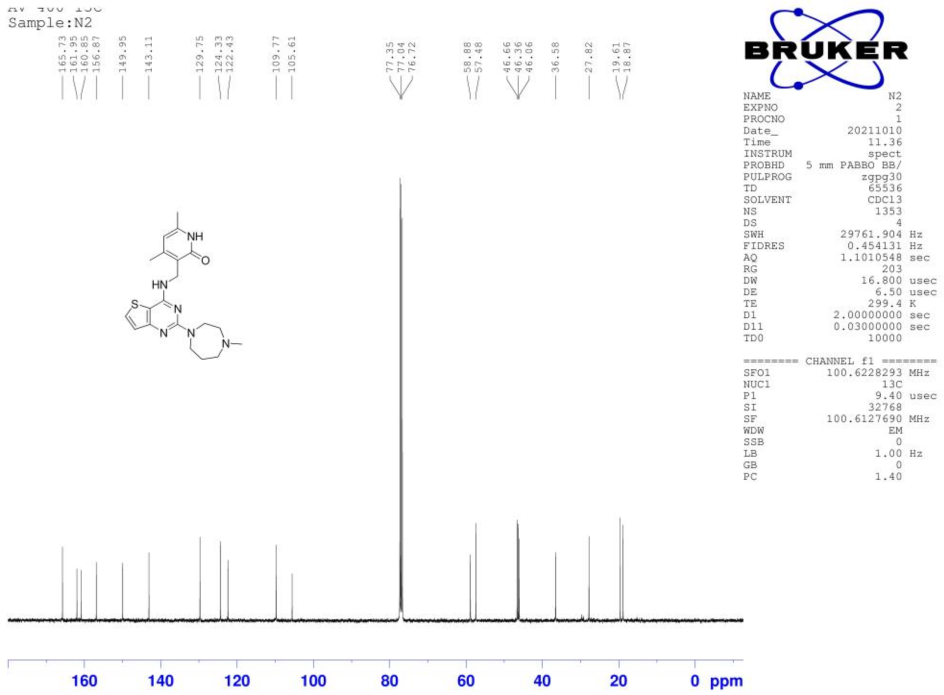

^{13}C NMR Spectrum of N2

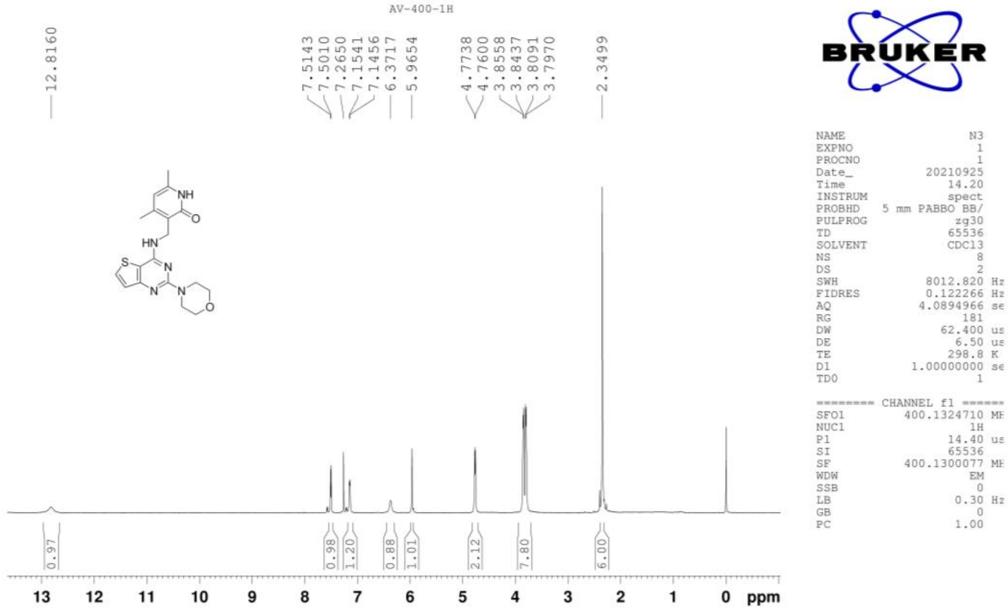

^{1}H NMR Spectrum of N3

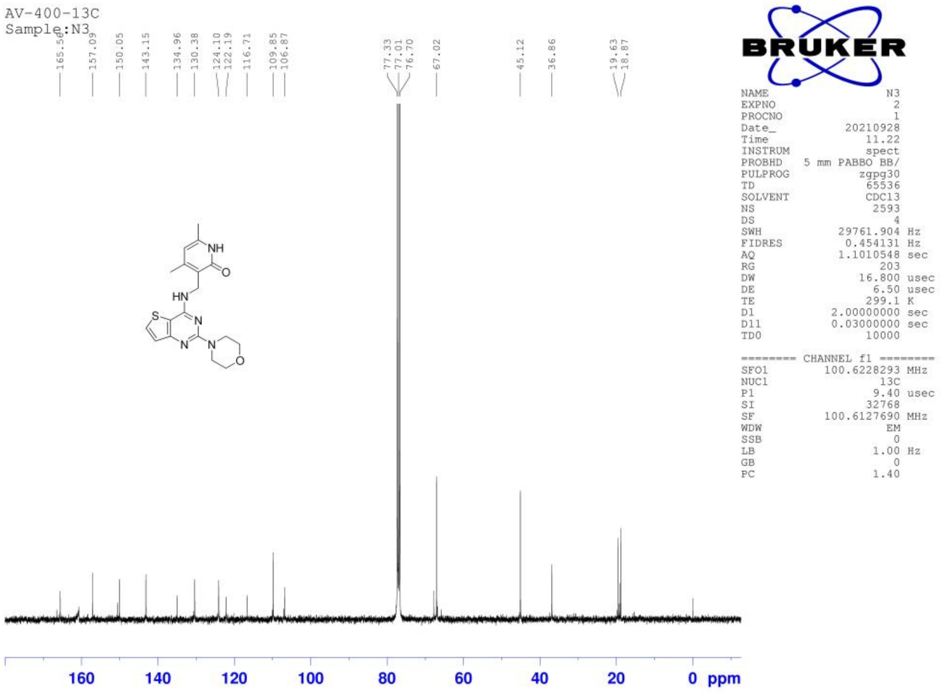

^{13}C NMR Spectrum of N3

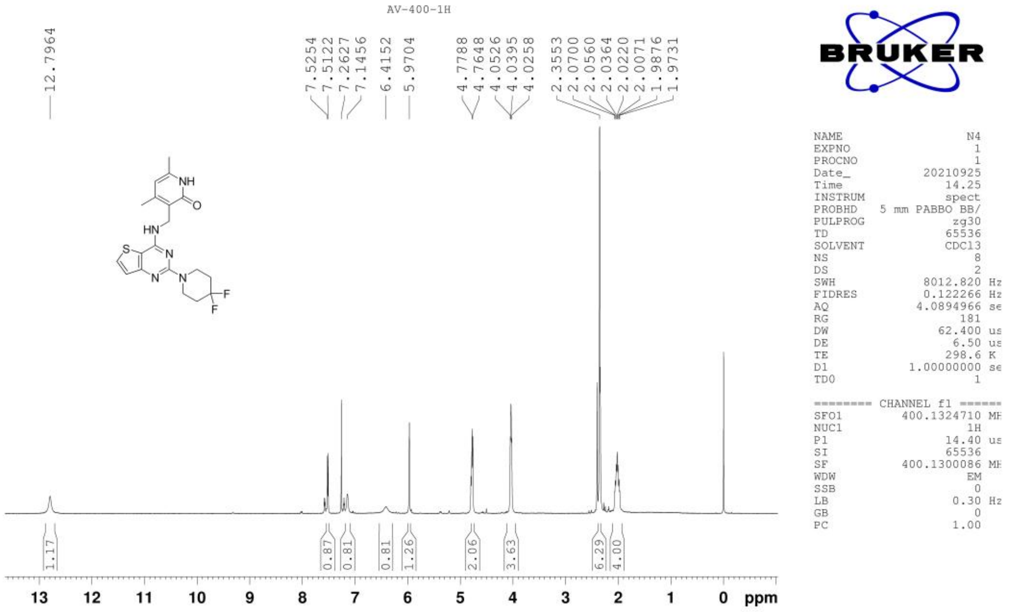

^{1}H NMR Spectrum of N4

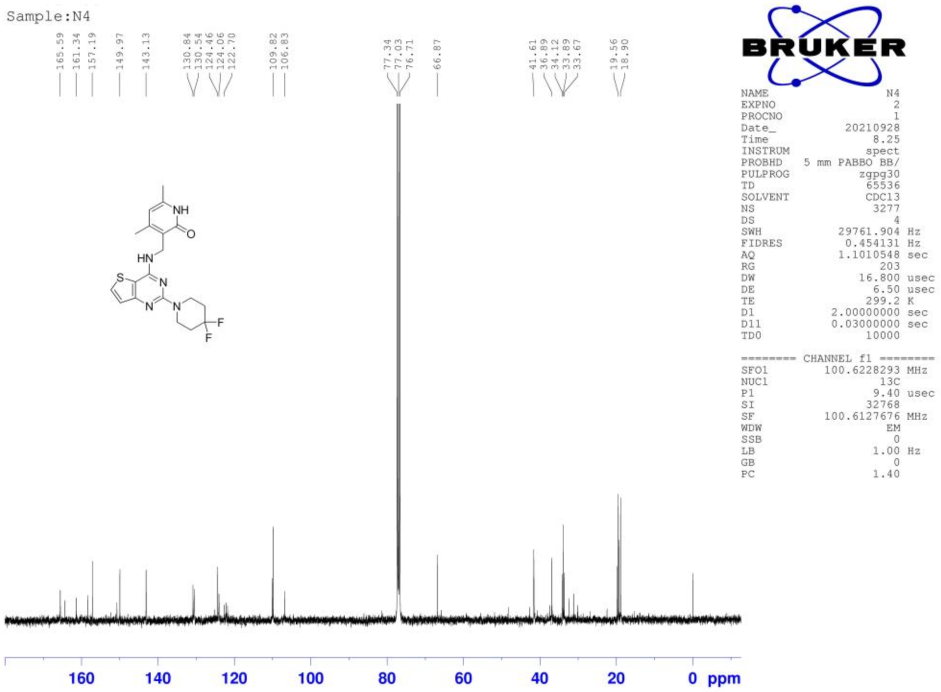

^{13}C NMR Spectrum of N4

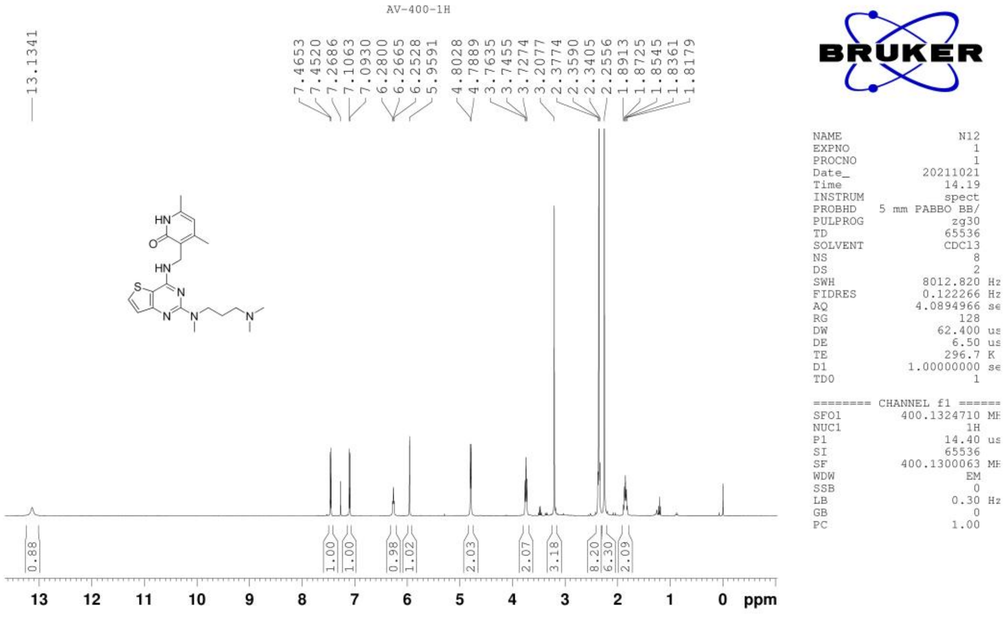

^{1}H NMR Spectrum of N5

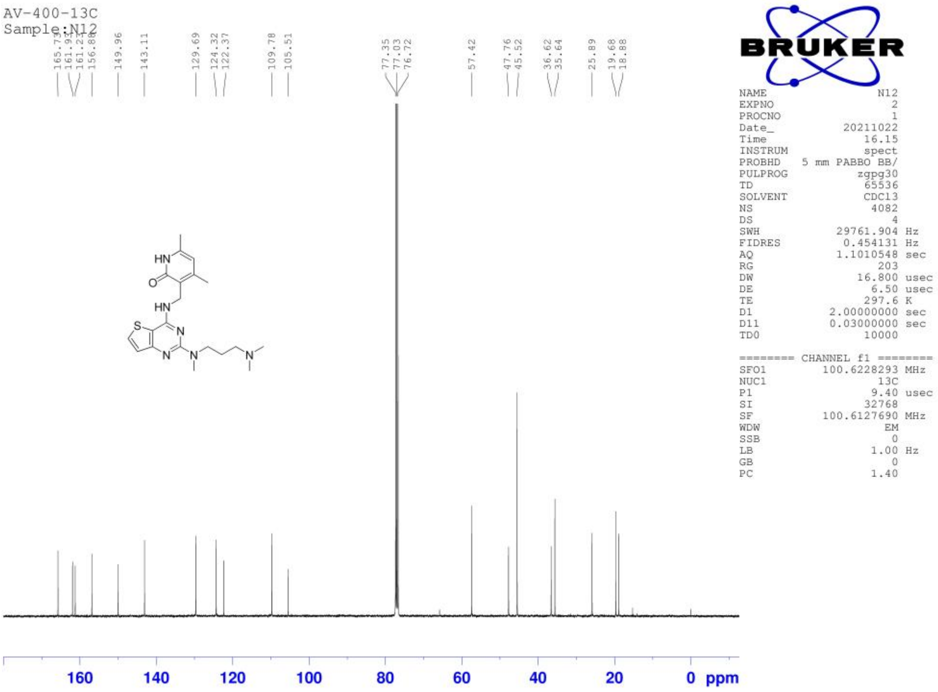

^{13}C NMR Spectrum of N5

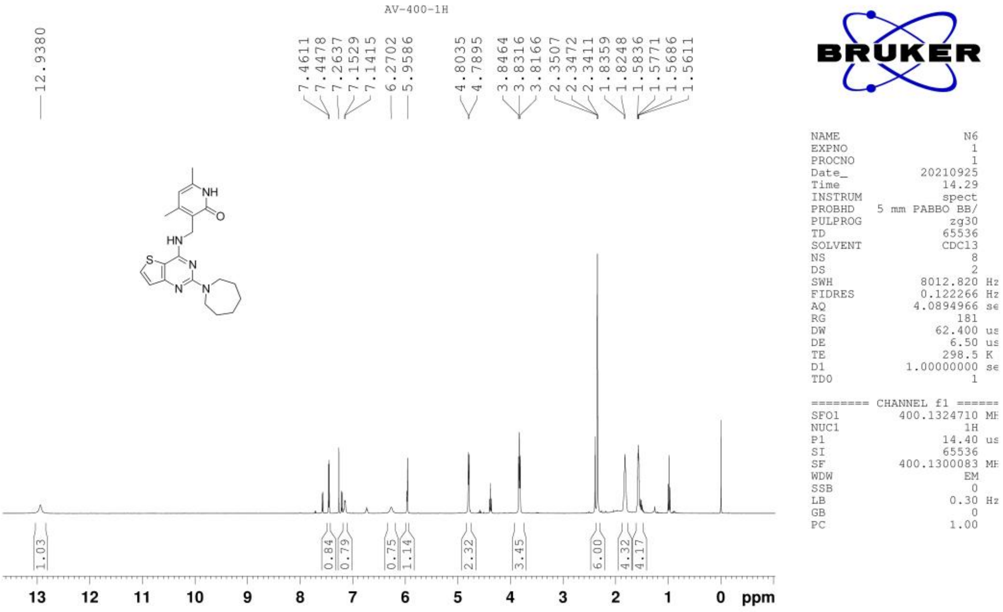

^{1}H NMR Spectrum of N6

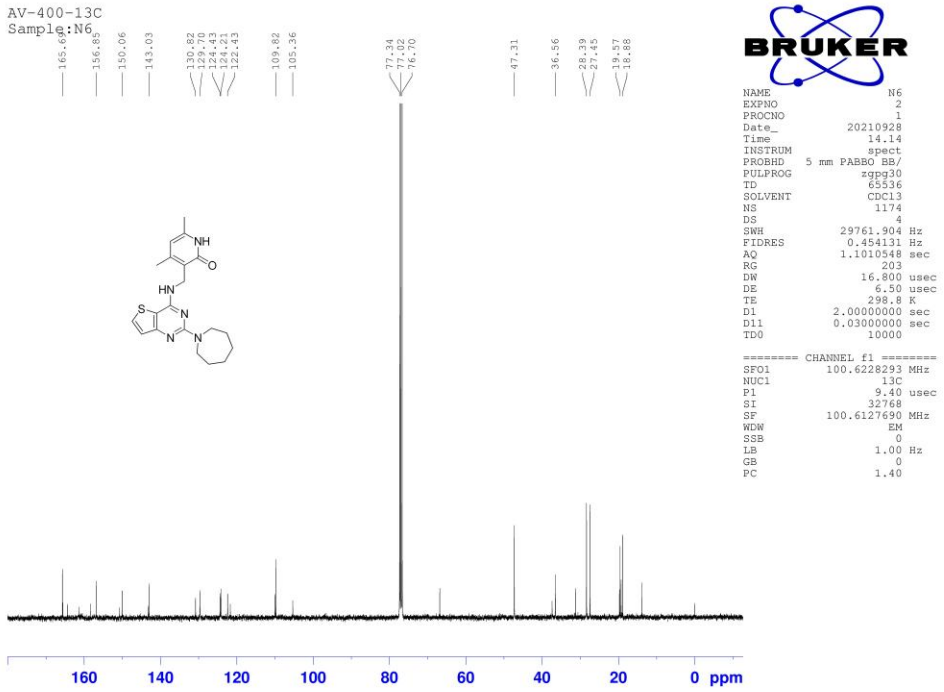

^{13}C NMR Spectrum of N6

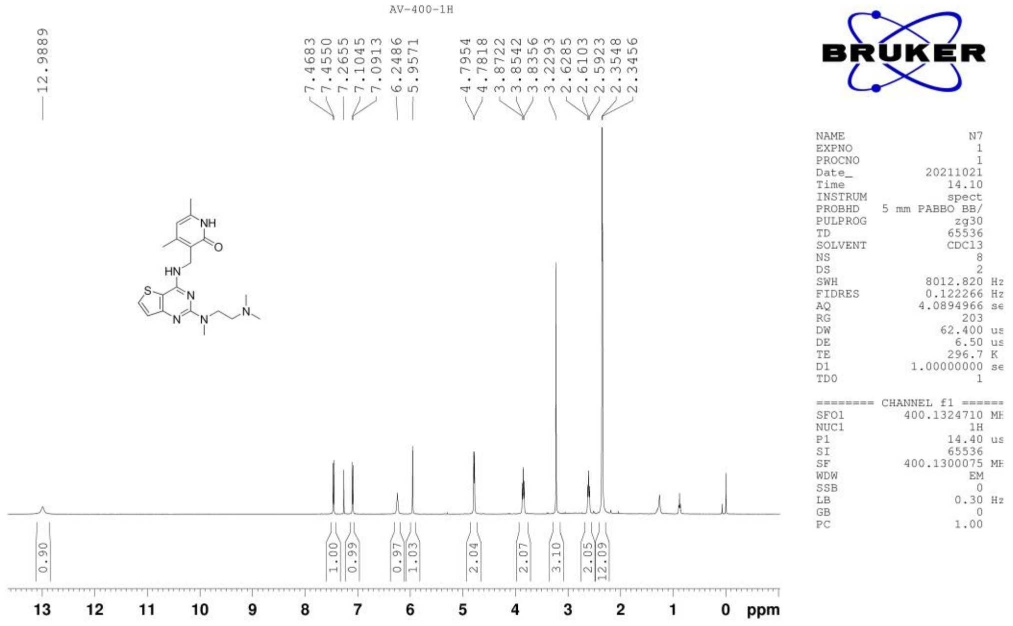

^{1}H NMR Spectrum of N7

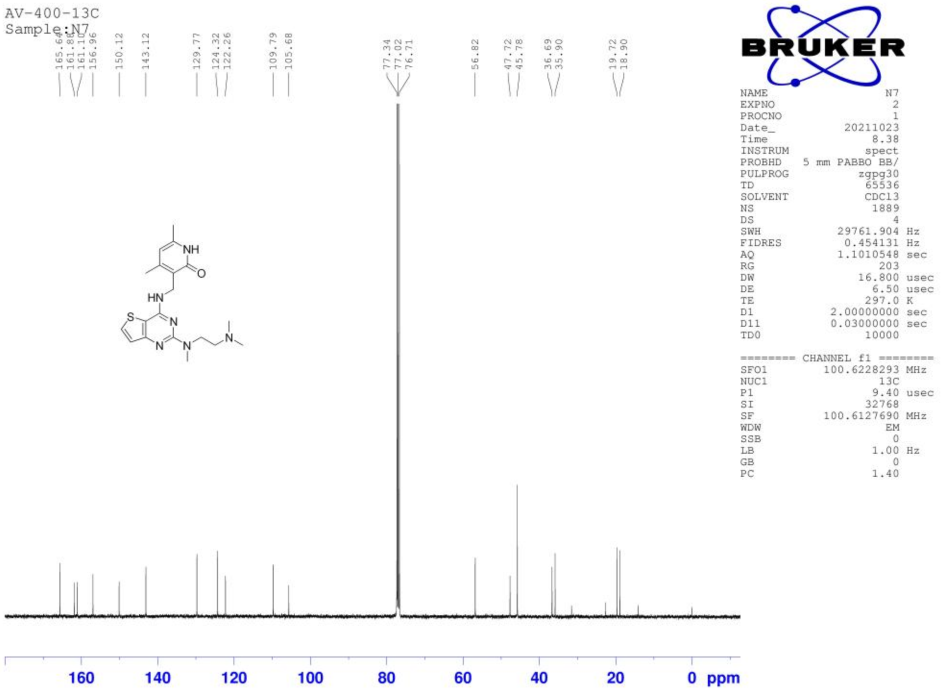

^{13}C NMR Spectrum of N7

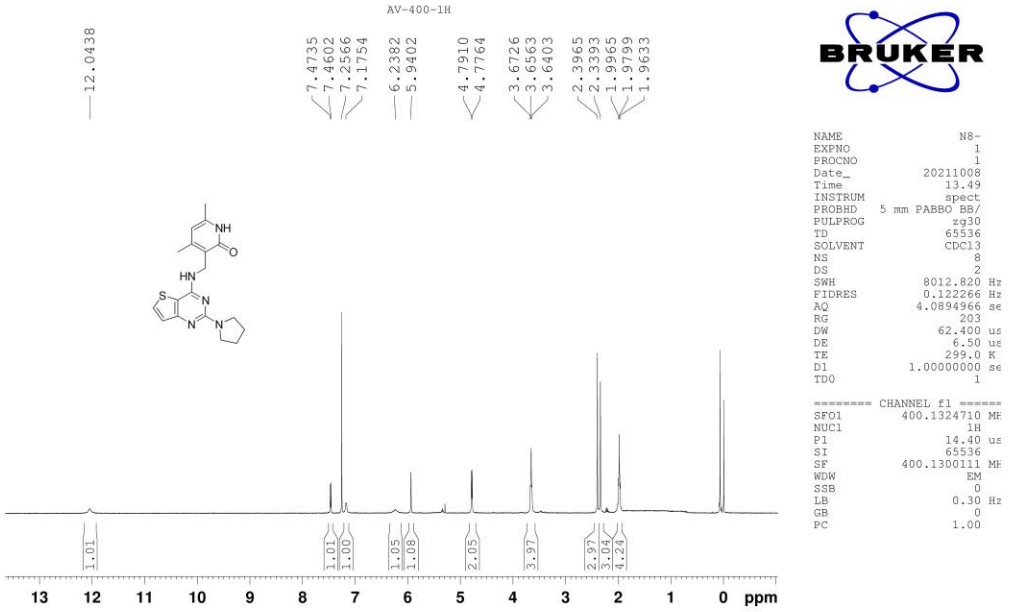

^{1}H NMR Spectrum of N8

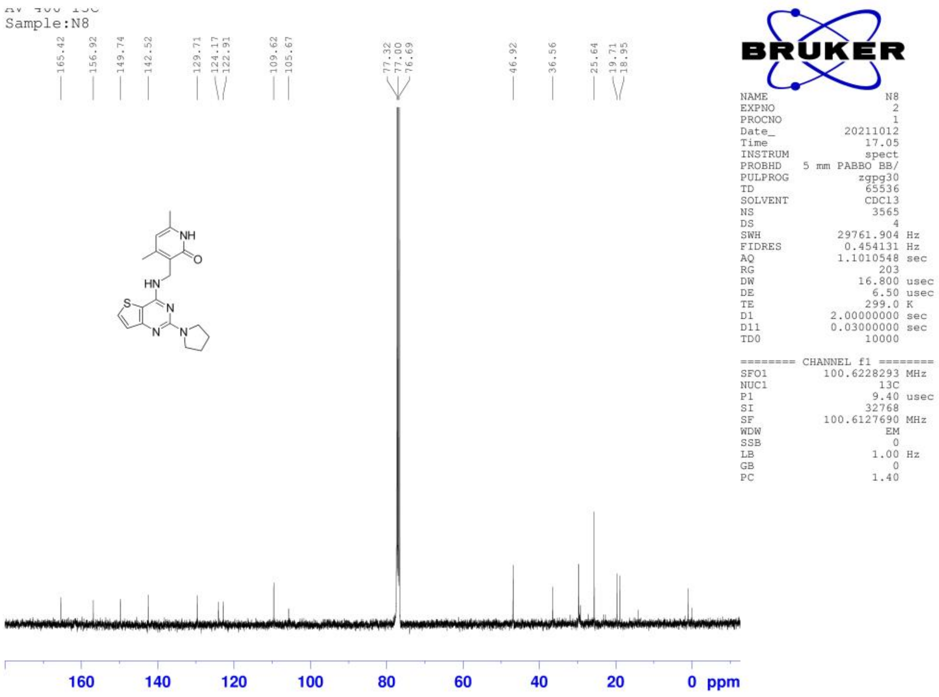

^{13}C NMR Spectrum of N8

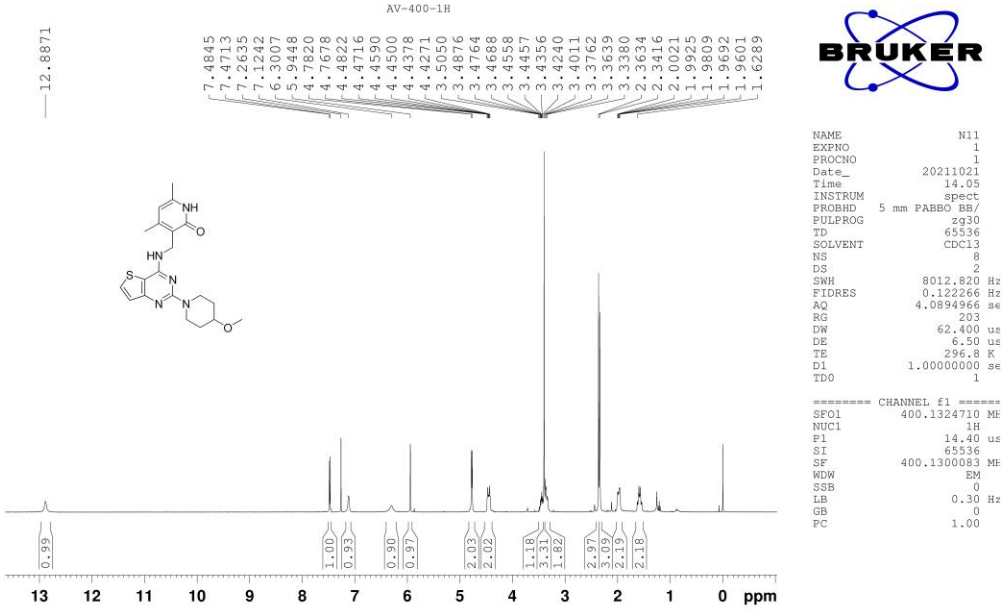

^{1}H NMR Spectrum of N9

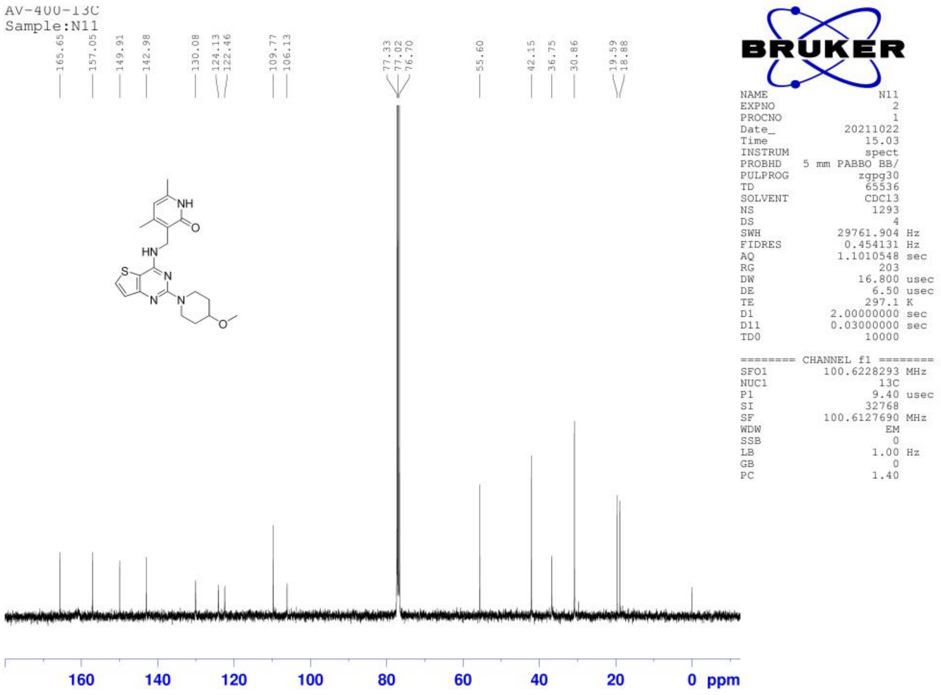

^{13}C NMR Spectrum of N9

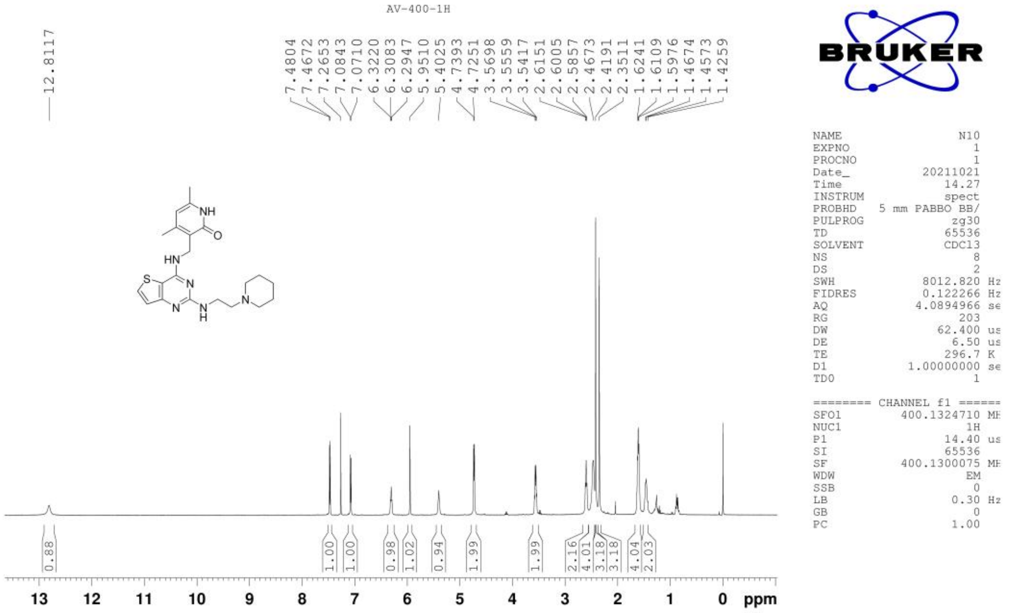

^{1}H NMR Spectrum of N10

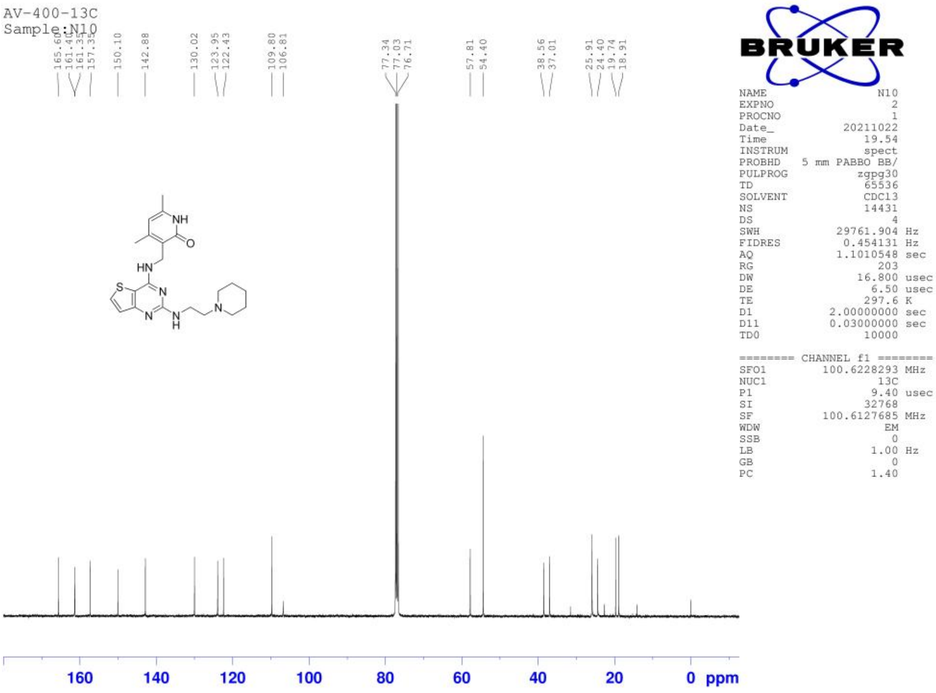

^{13}C NMR Spectrum of N10

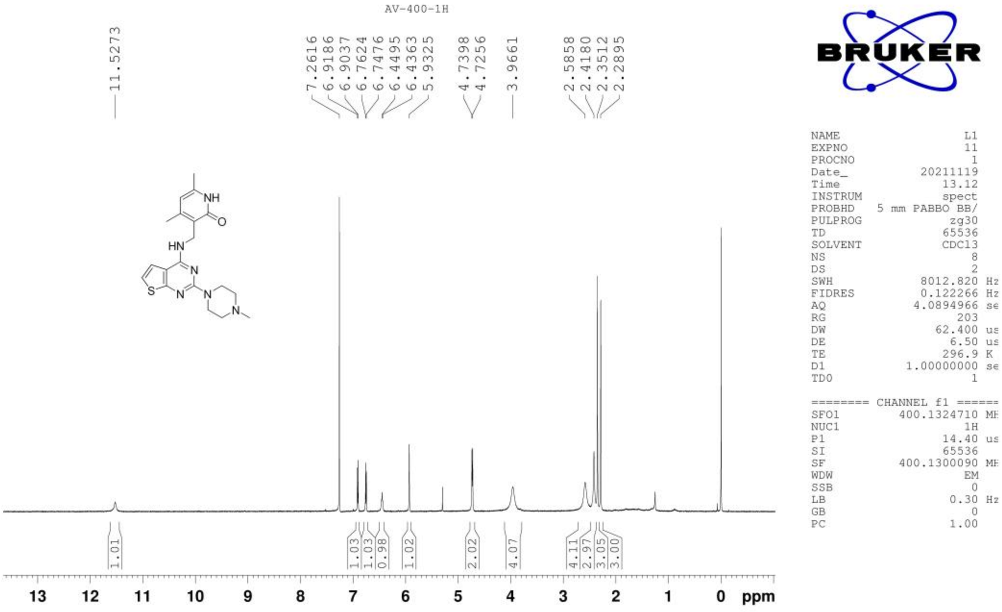

^{1}H NMR Spectrum of L1

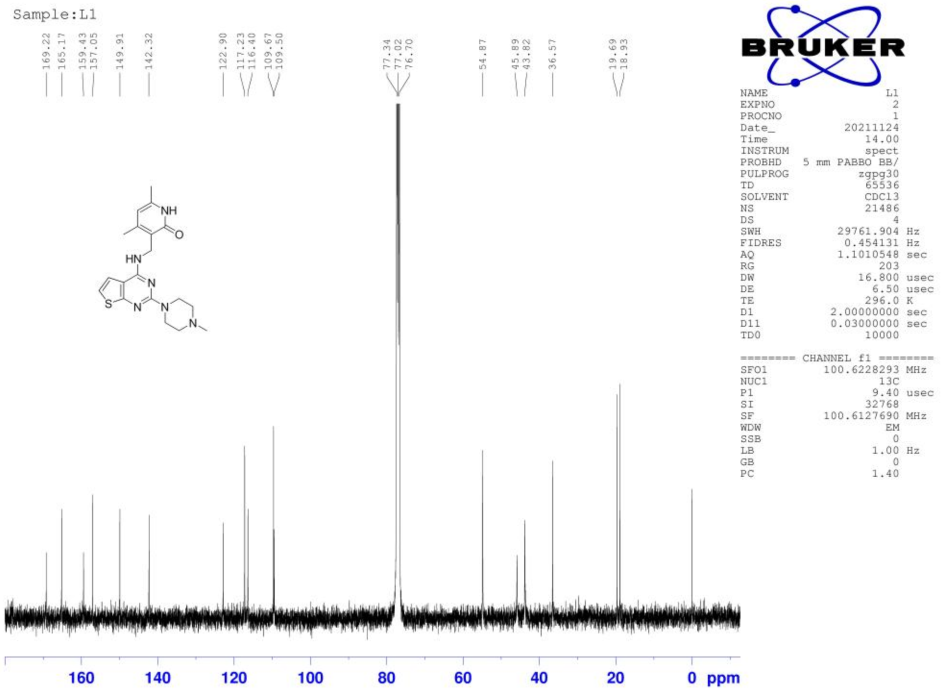

^{13}C NMR Spectrum of L1

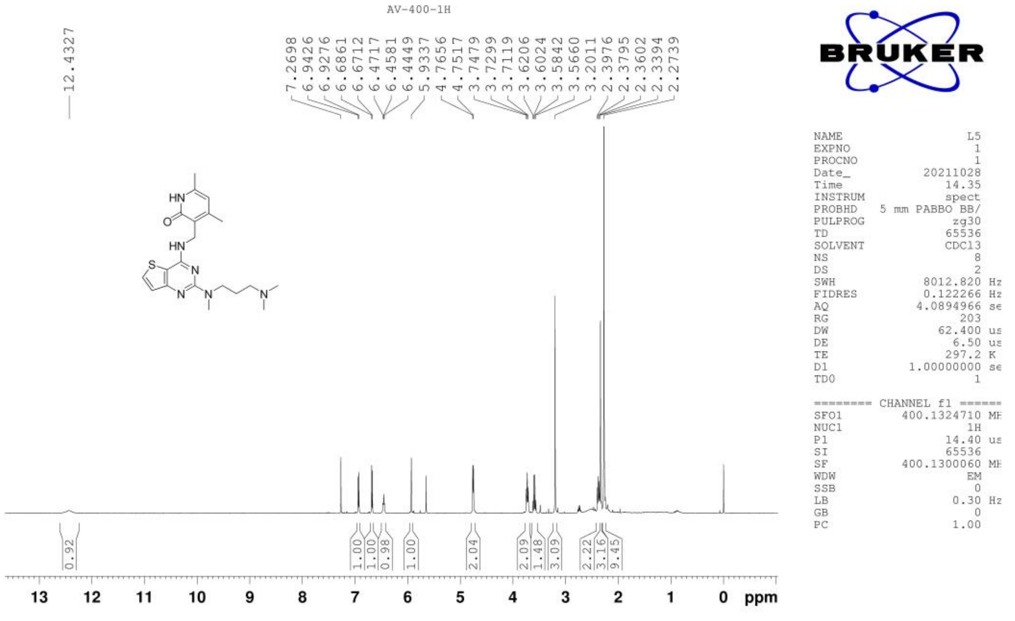

^{1}H NMR Spectrum of L2

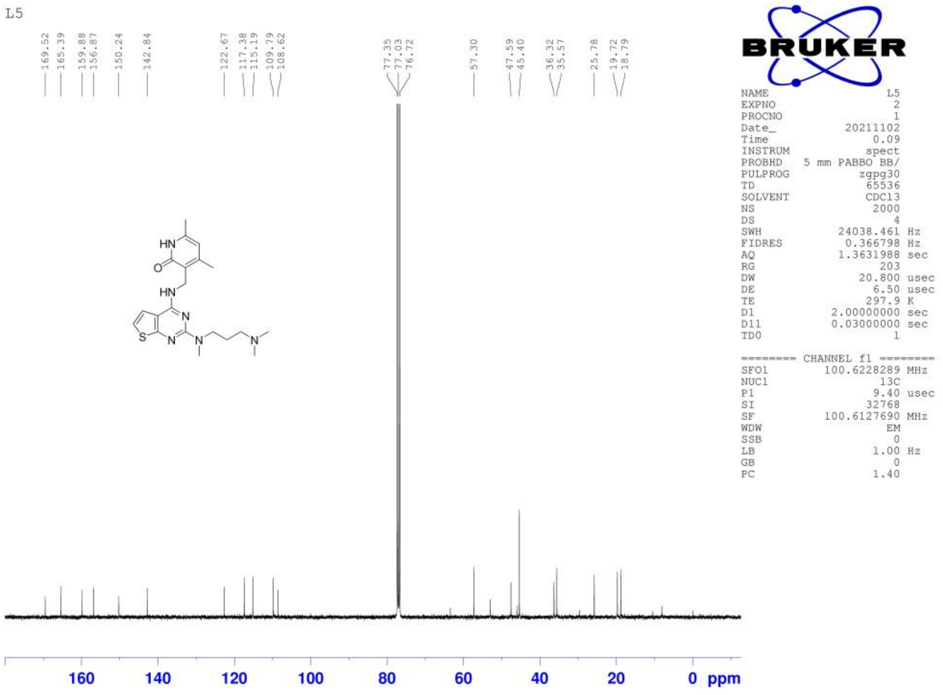

^{13}C NMR Spectrum of L2

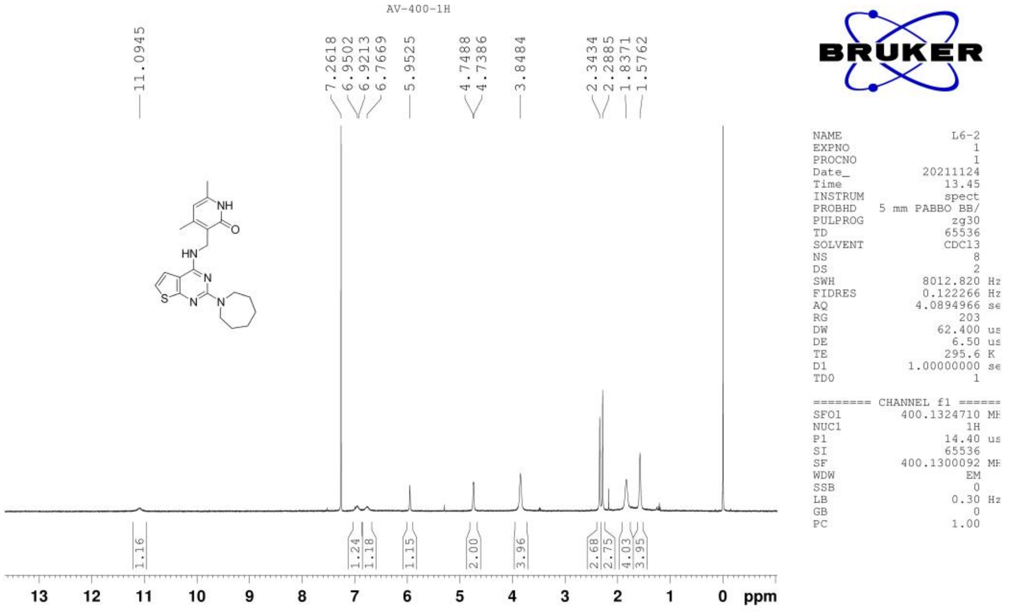

^{1}H NMR Spectrum of L3

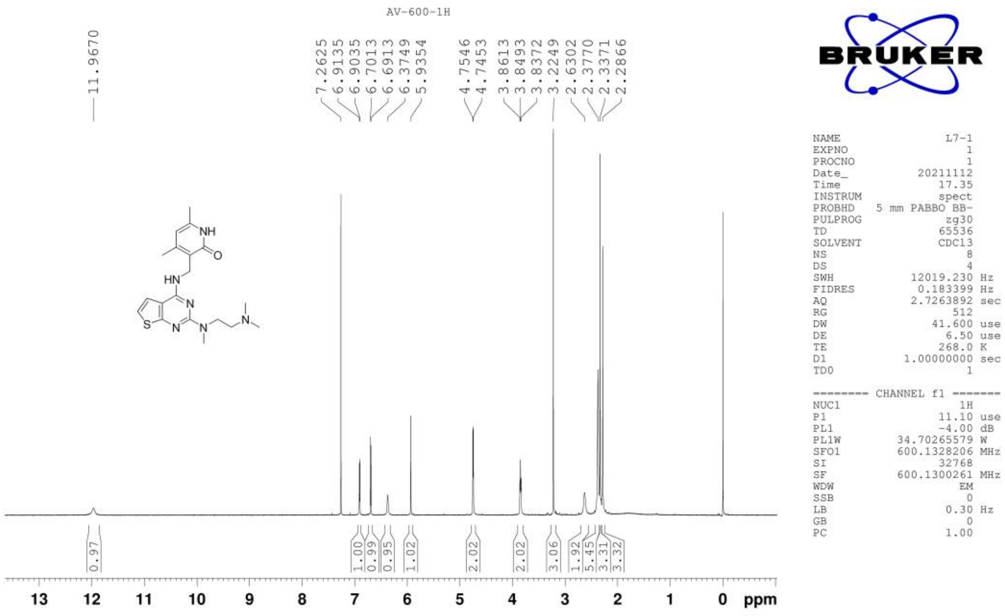

^{1}H NMR Spectrum of L4

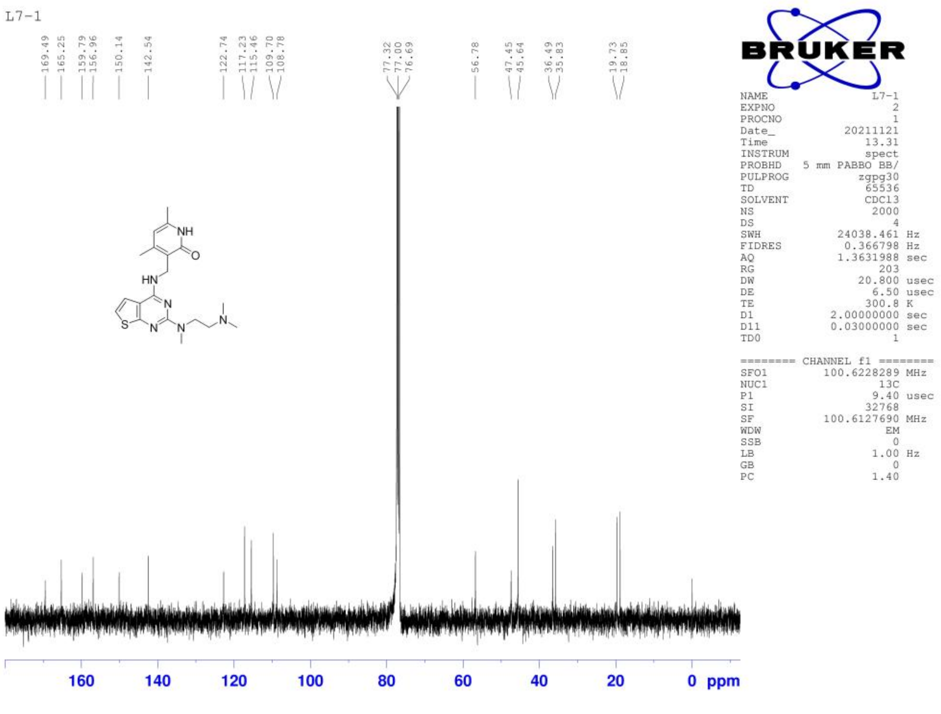

^{13}C NMR Spectrum of L4

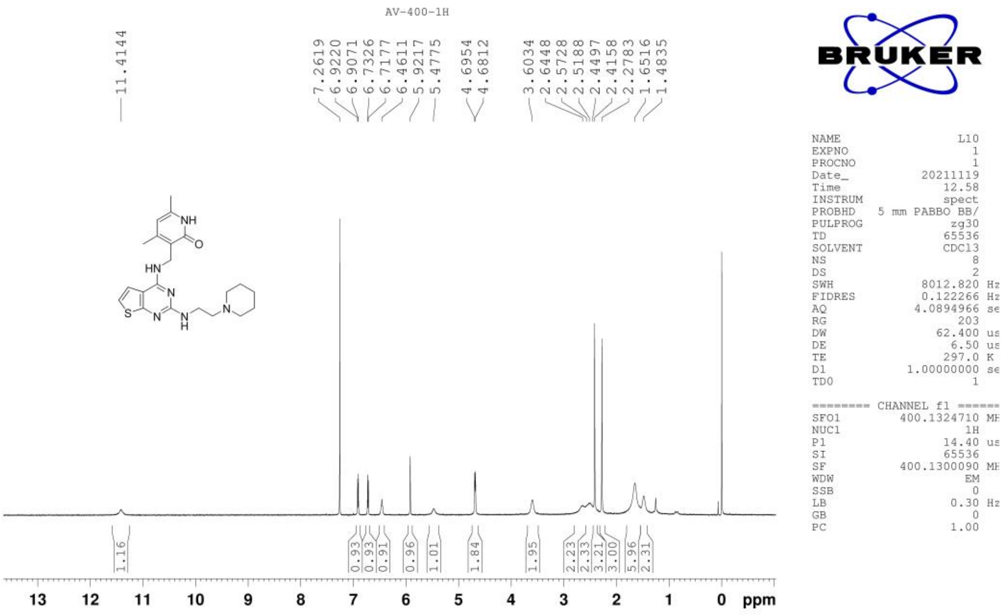

^{1}H NMR Spectrum of L5

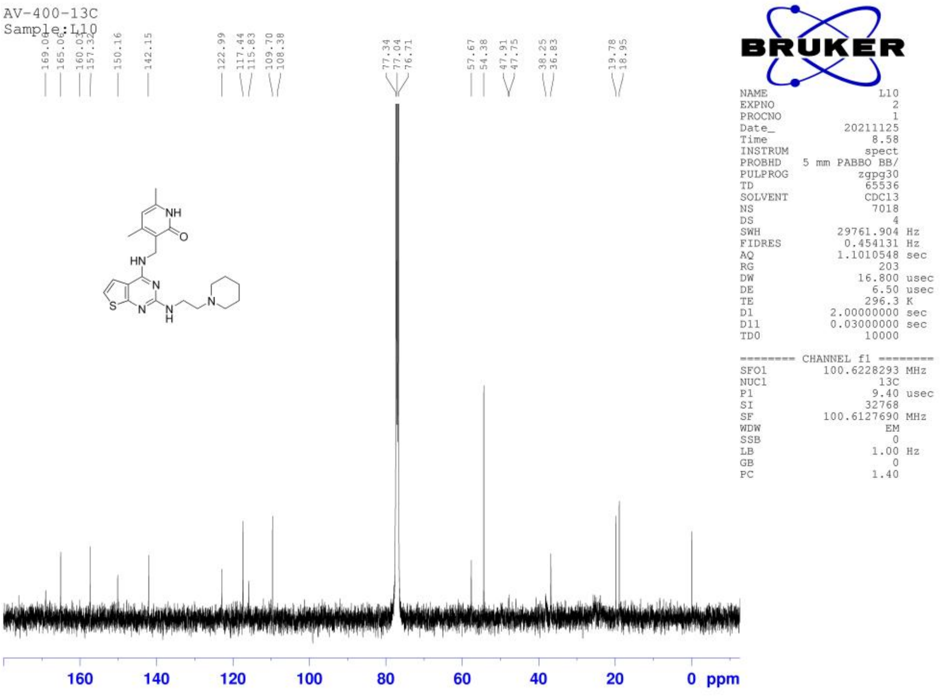

^{13}C NMR Spectrum of L5

S8. MS Spectra of compounds

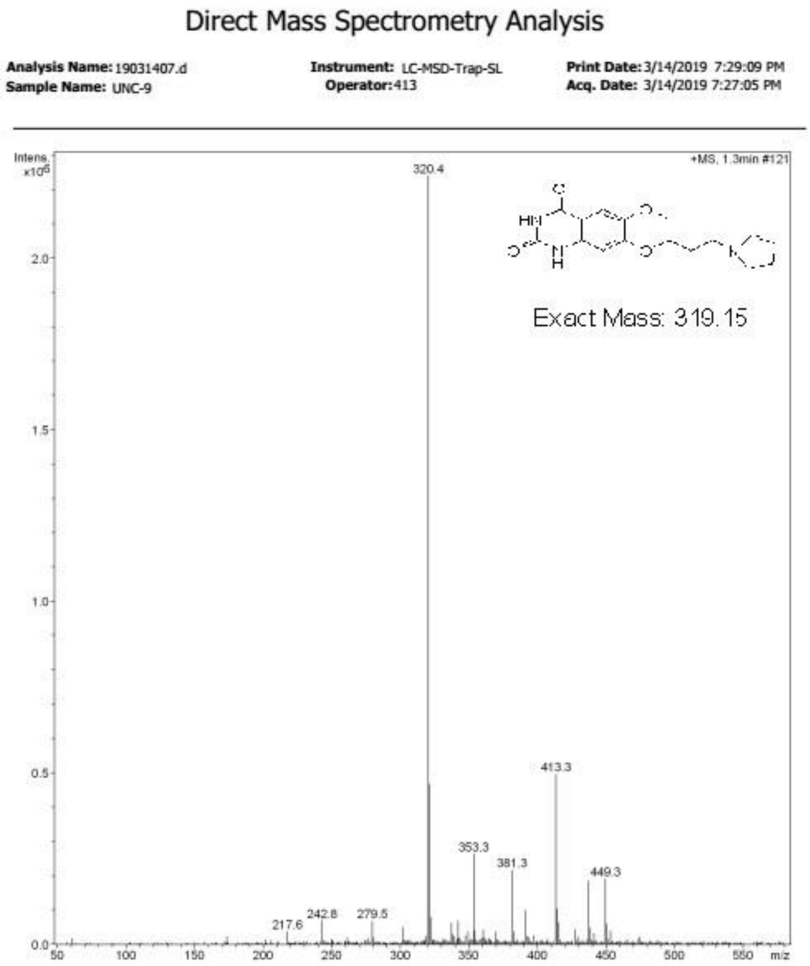

ESI-MS Spectrum of 7

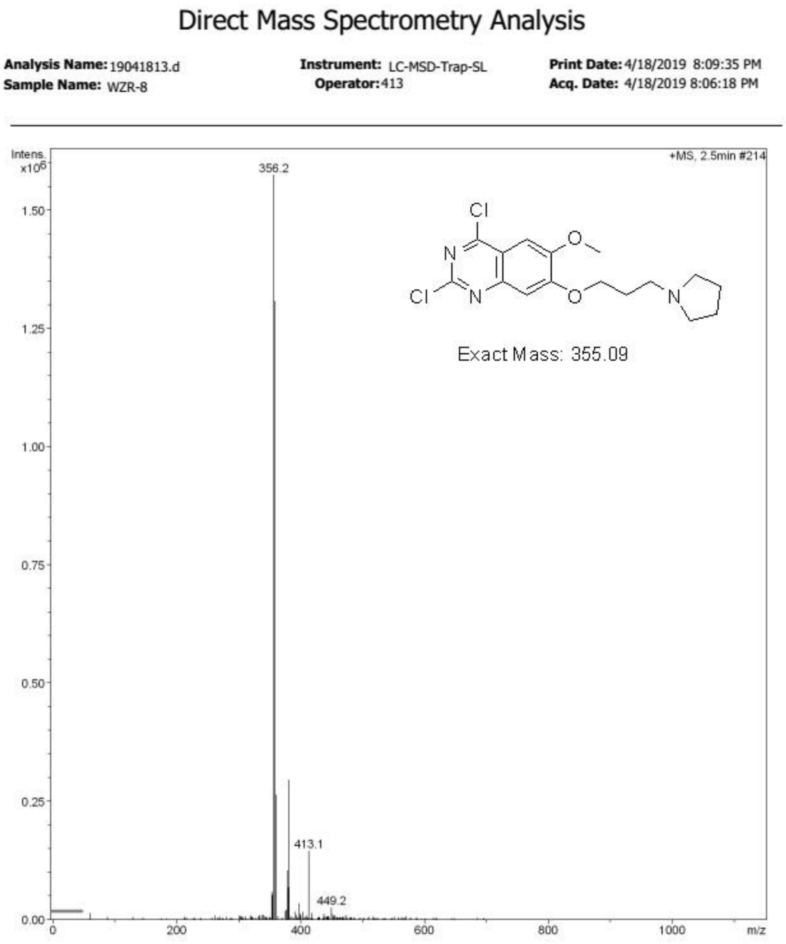

ESI-MS Spectrum of 8

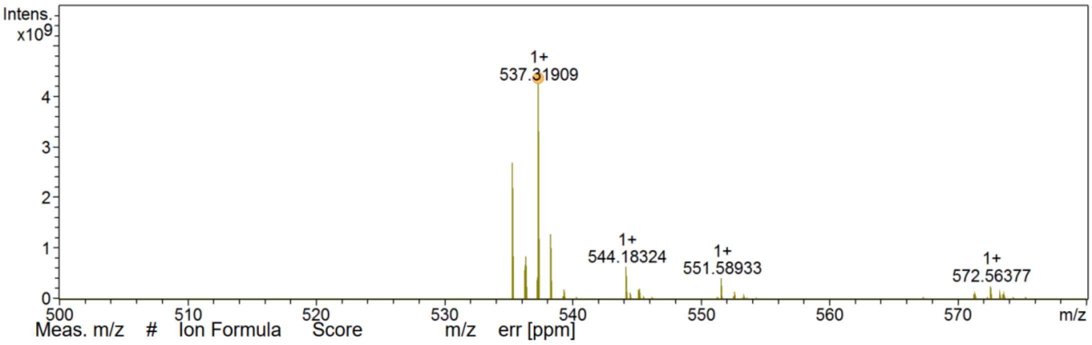

Meas. m/z	#	Ion Formula	Score	m/z	err [ppm]
537.319088	1	C29H41N6O4	100.00	537.318380	-1.3

ESI-HRMS Spectrum of SU101

Direct Mass Spectrometry Analysis

Analysis Name: 23032501.d
Sample Name: SU102
Instrument: LC-MSD-Trap-SL
Operator: 21953830E000H12U
Print Date: 3/25/2023 10:14:06 AM
Acq. Date: 3/25/2023 10:12:54 AM

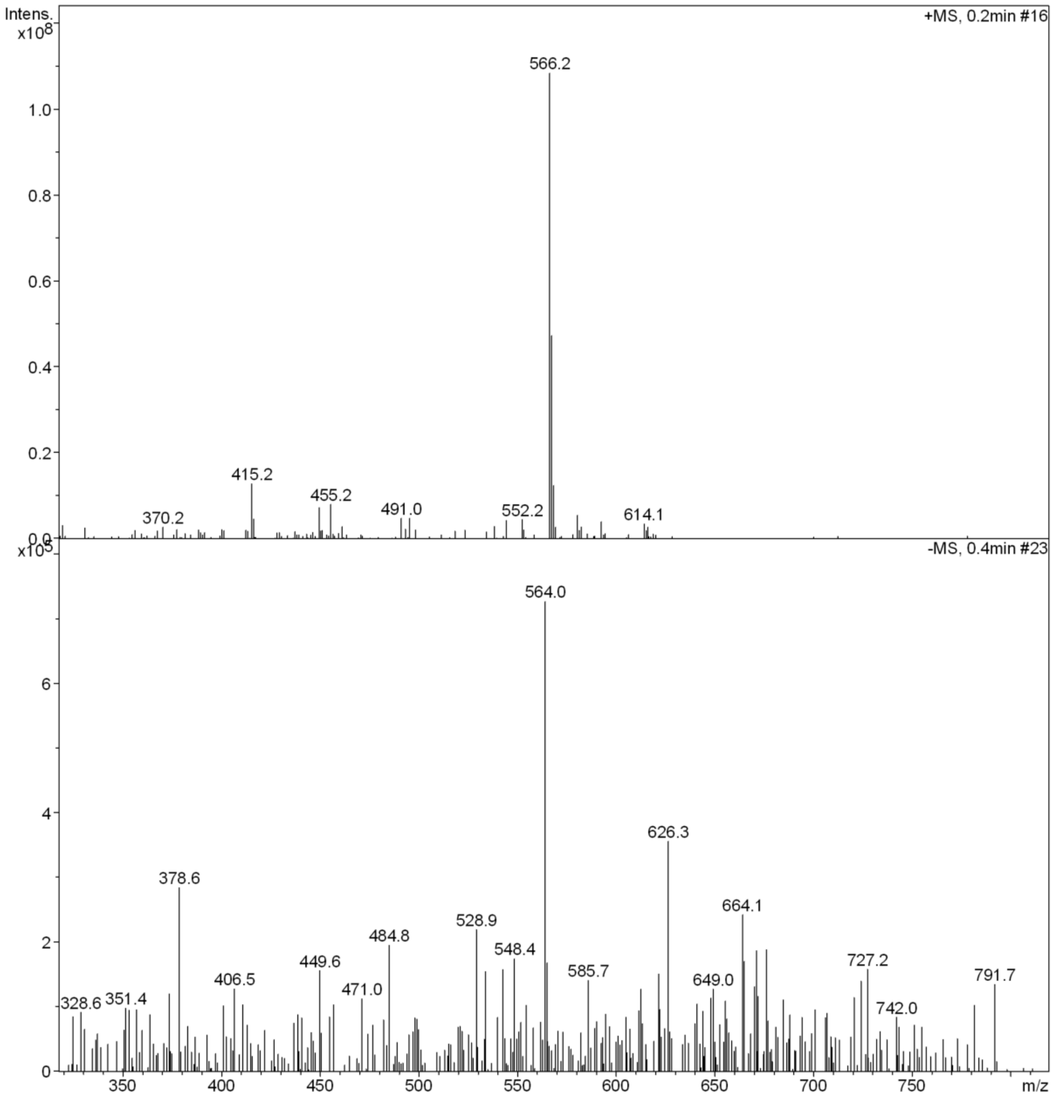

ESI-MS Spectrum of SU102

Direct Mass Spectrometry Analysis

Analysis Name: 23032506.d
Sample Name: SU103

Instrument: LC-MSD-Trap-SL
Operator: 21953830E000H12U

Print Date: 3/25/2023 10:29:40 AM
Acq. Date: 3/25/2023 10:26:06 AM

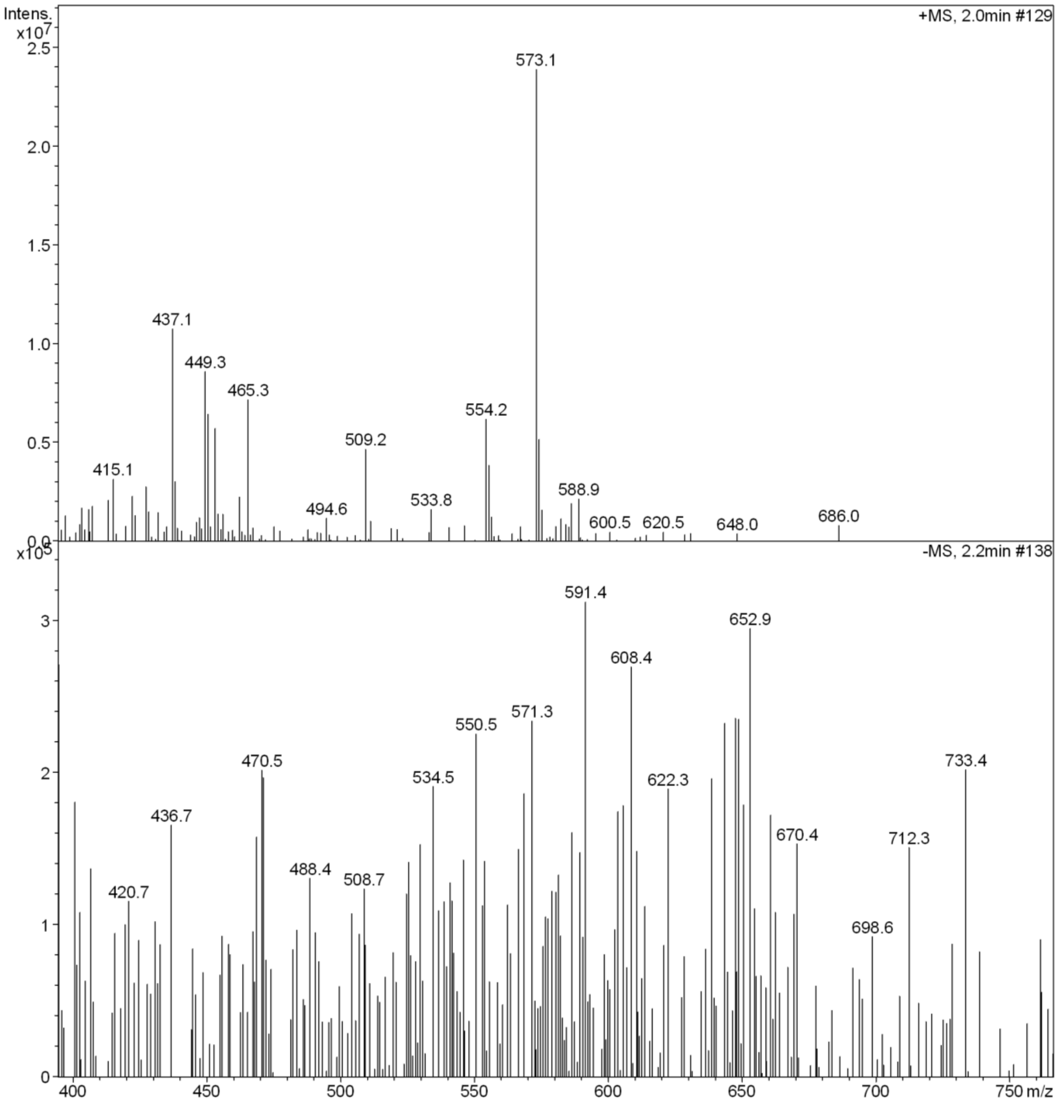

ESI-MS Spectrum of SU103

Direct Mass Spectrometry Analysis

Analysis Name: 23032508.d **Instrument:** LC-MSD-Trap-SL **Print Date:** 3/25/2023 10:35:41 AM
Sample Name: SU104 **Operator:** 21953830E000H12U **Acq. Date:** 3/25/2023 10:34:35 AM

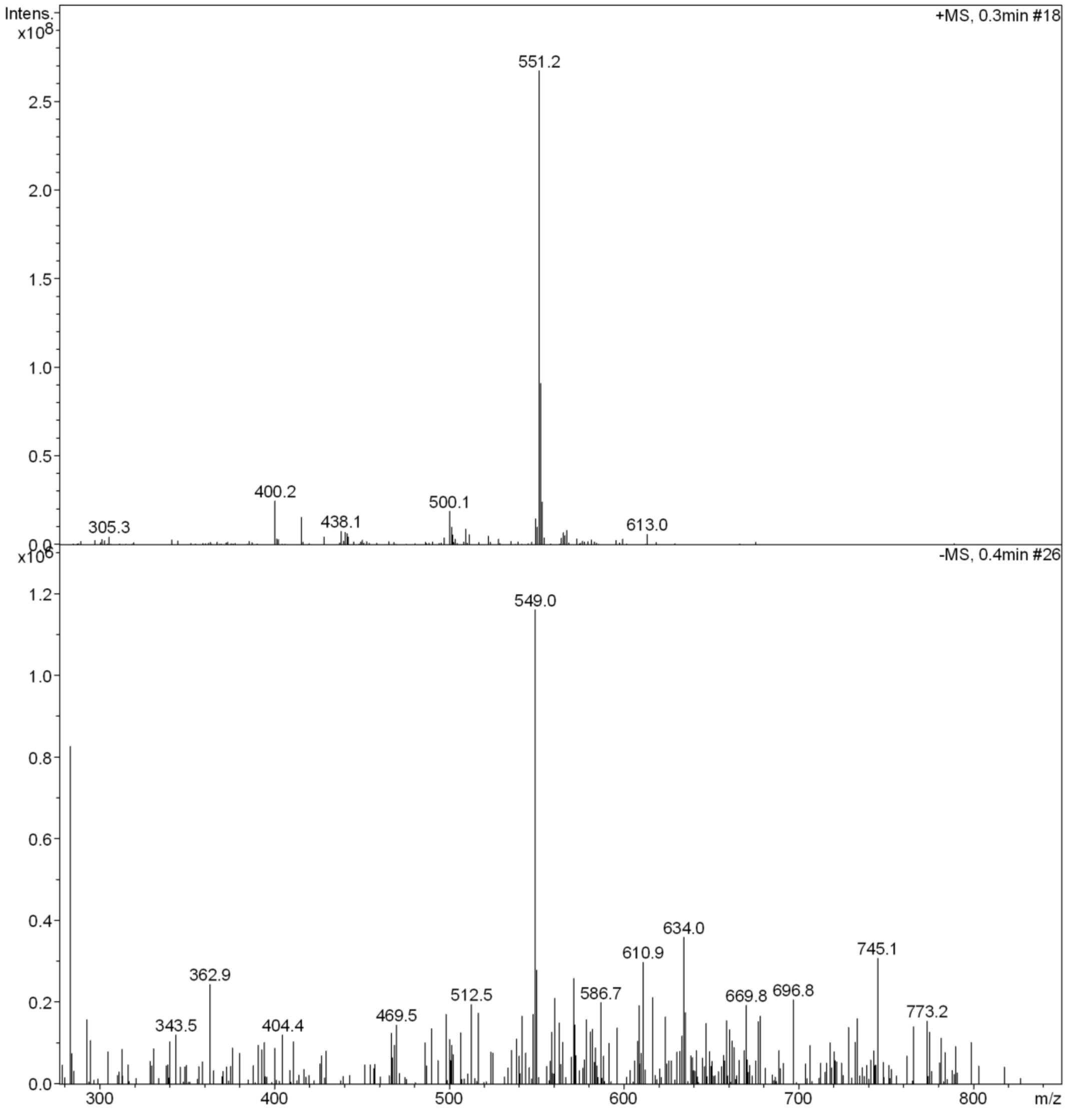

ESI-MS Spectrum of SU104

Direct Mass Spectrometry Analysis

Analysis Name: 23032505.d
Sample Name: SU105
Instrument: LC-MSD-Trap-SL
Operator: 21953830E000H12U
Print Date: 3/25/2023 10:25:17 AM
Acq. Date: 3/25/2023 10:23:52 AM

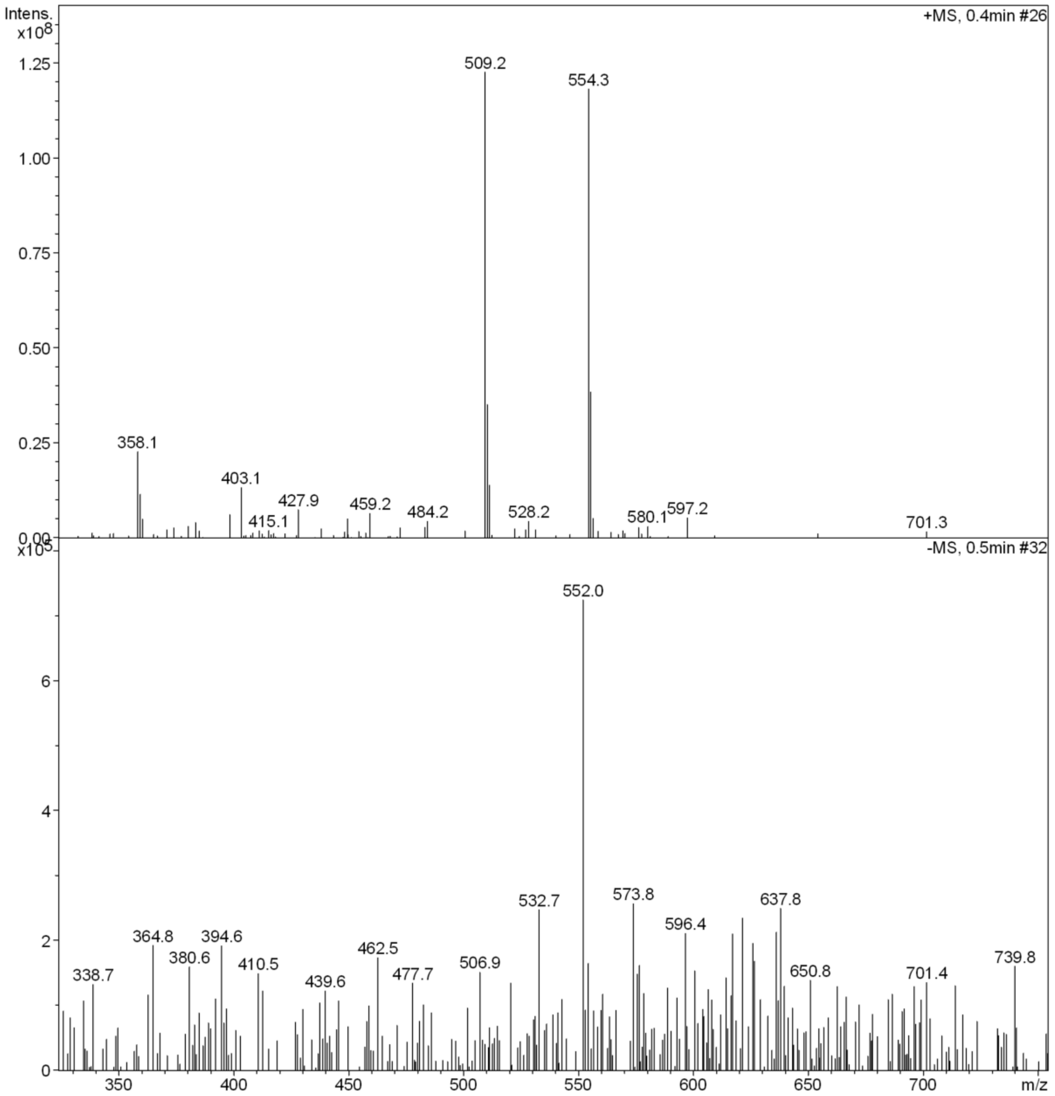

ESI-MS Spectrum of SU105

Direct Mass Spectrometry Analysis

Analysis Name: 23032507.d
Sample Name: SU203
Instrument: LC-MSD-Trap-SL
Operator: 21953830E000H12U
Print Date: 3/25/2023 10:33:20 AM
Acq. Date: 3/25/2023 10:31:56 AM

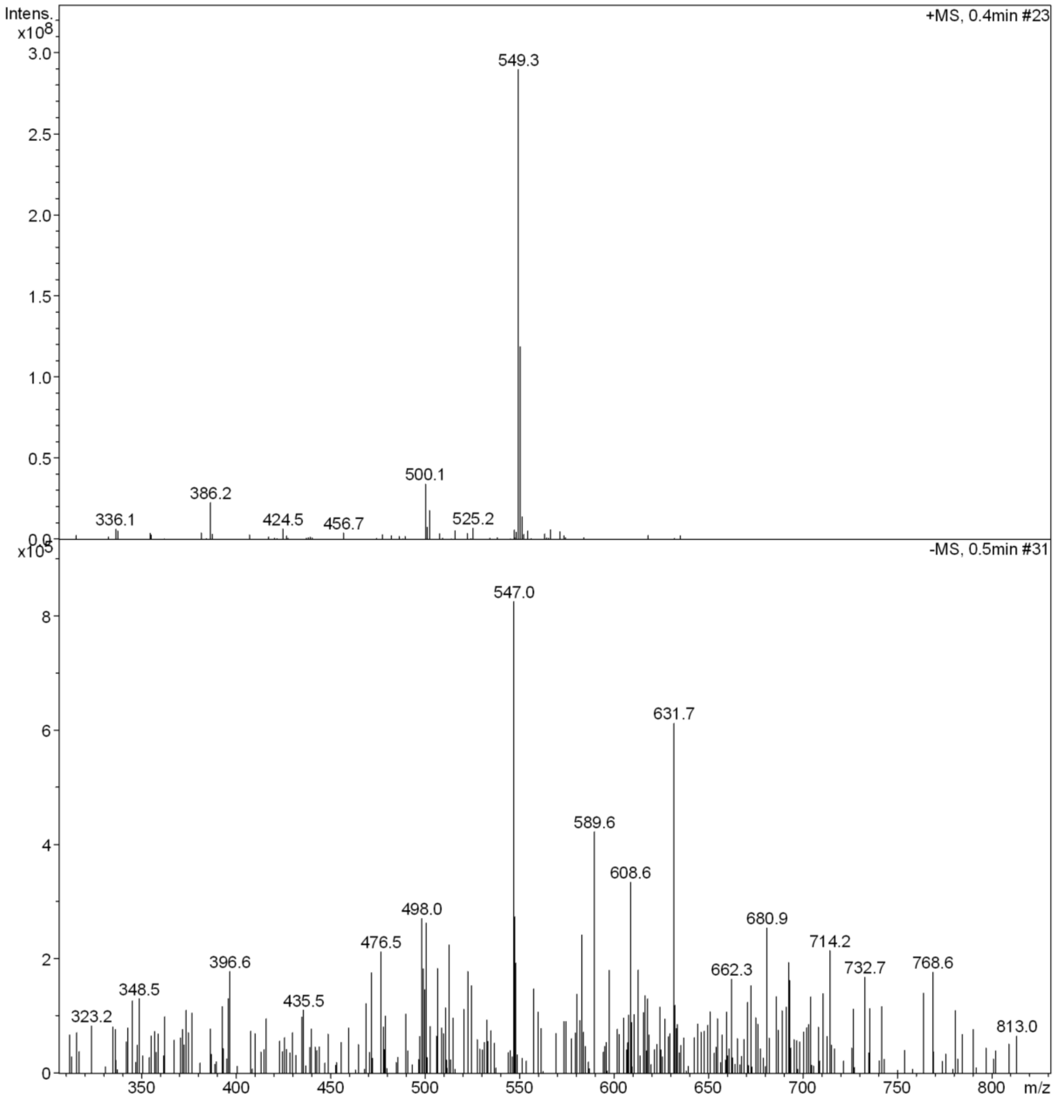

ESI-MS Spectrum of SU201

Direct Mass Spectrometry Analysis

Analysis Name: 23032509.d
Sample Name: SU202
Instrument: LC-MSD-Trap-SL
Operator: 21953830E000H12U
Print Date: 3/25/2023 10:38:46 AM
Acq. Date: 3/25/2023 10:36:42 AM

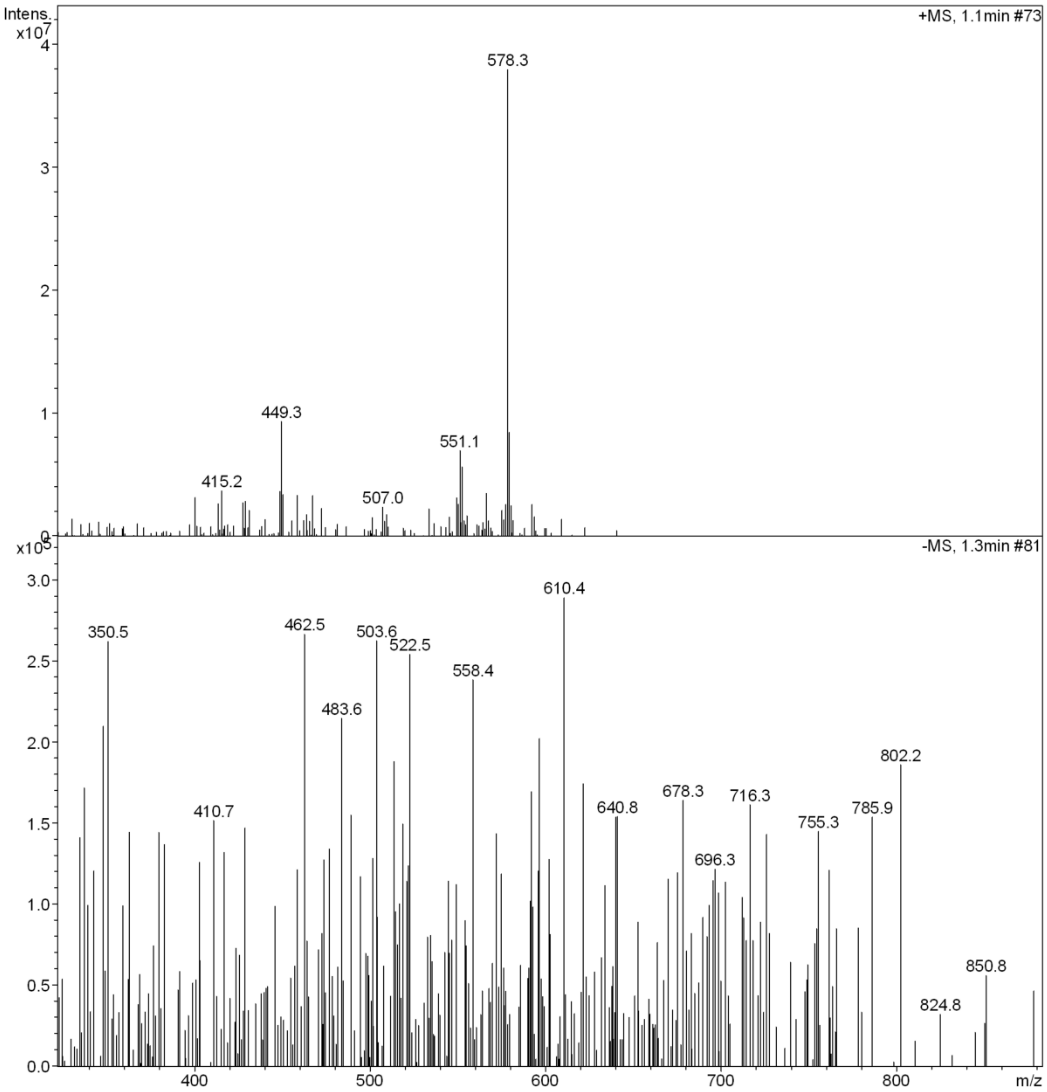

ESI-MS Spectrum of SU202

Direct Mass Spectrometry Analysis

Analysis Name: 23032503.d **Instrument:** LC-MSD-Trap-SL **Print Date:** 3/25/2023 10:20:25 AM
Sample Name: SU201 **Operator:** 21953830E000H12U **Acq. Date:** 3/25/2023 10:18:50 AM

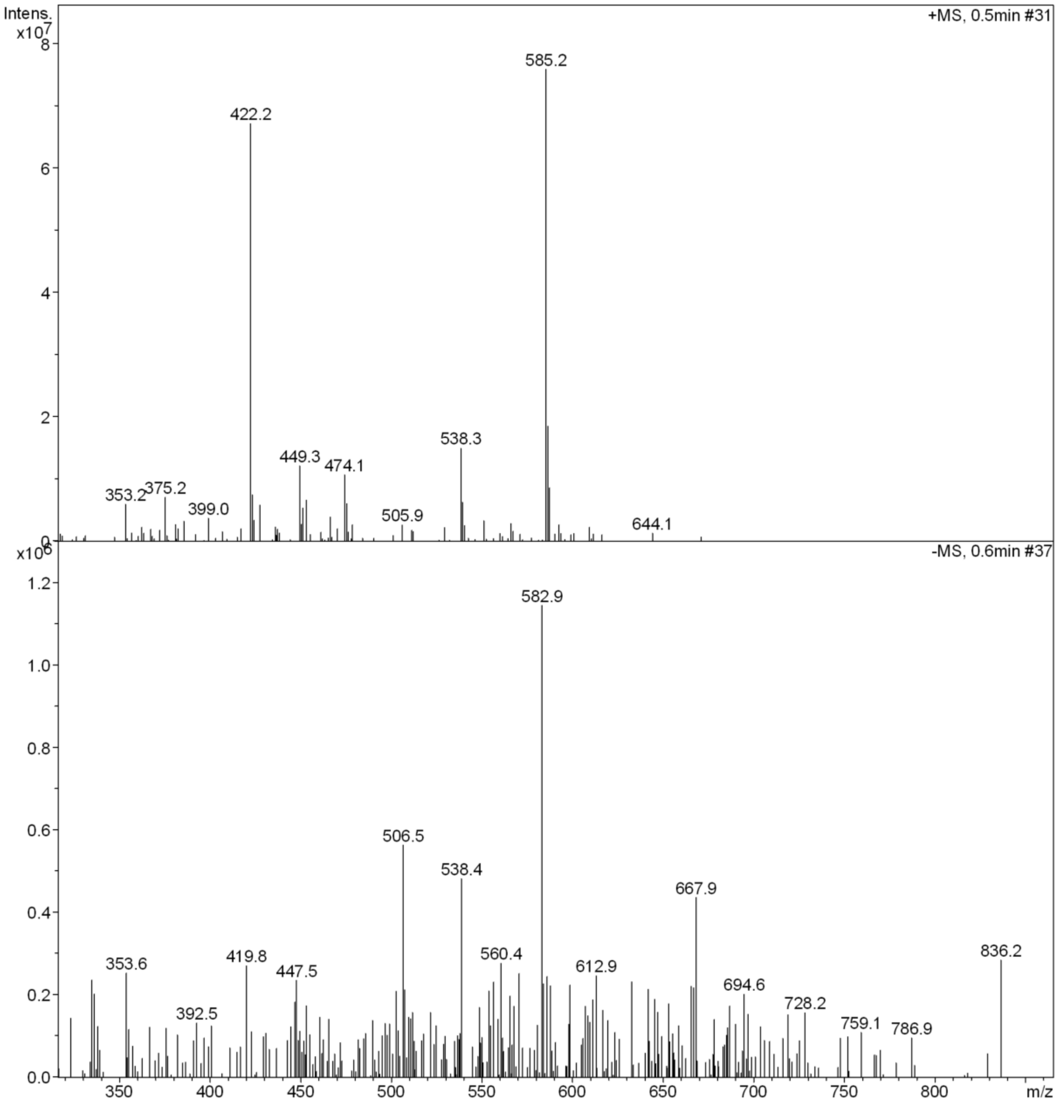

ESI-MS Spectrum of SU203

Direct Mass Spectrometry Analysis

Analysis Name: 23032511.d
Sample Name: SU204
Instrument: LC-MSD-Trap-SL
Operator: 21953830E000H12U
Print Date: 3/25/2023 10:44:15 AM
Acq. Date: 3/25/2023 10:43:09 AM

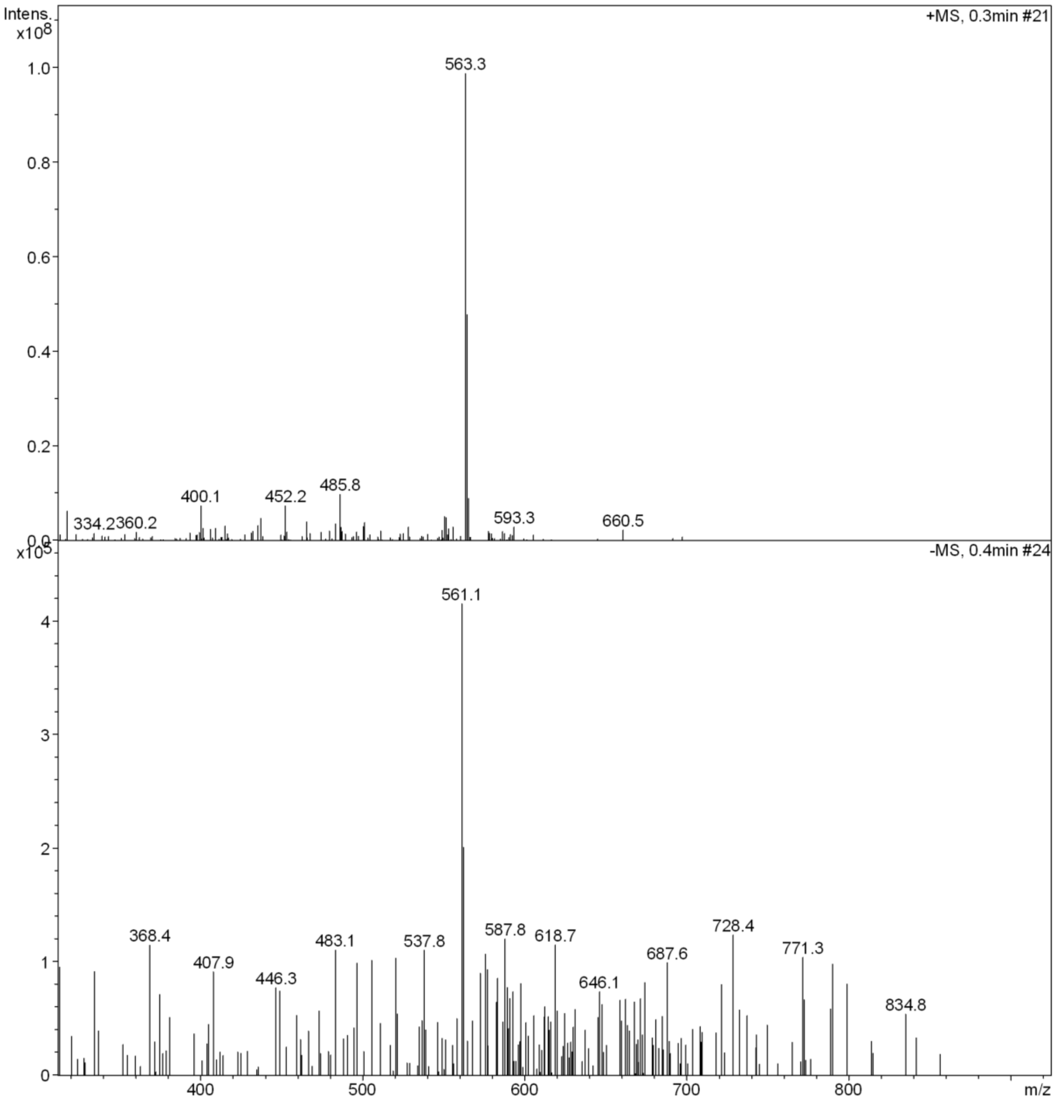

ESI-MS Spectrum of SU204

Direct Mass Spectrometry Analysis

Analysis Name: 23032515.d
Sample Name: SU205
Instrument: LC-MSD-Trap-SL
Operator: 21953830E000H12U
Print Date: 3/25/2023 10:54:18 AM
Acq. Date: 3/25/2023 10:51:55 AM

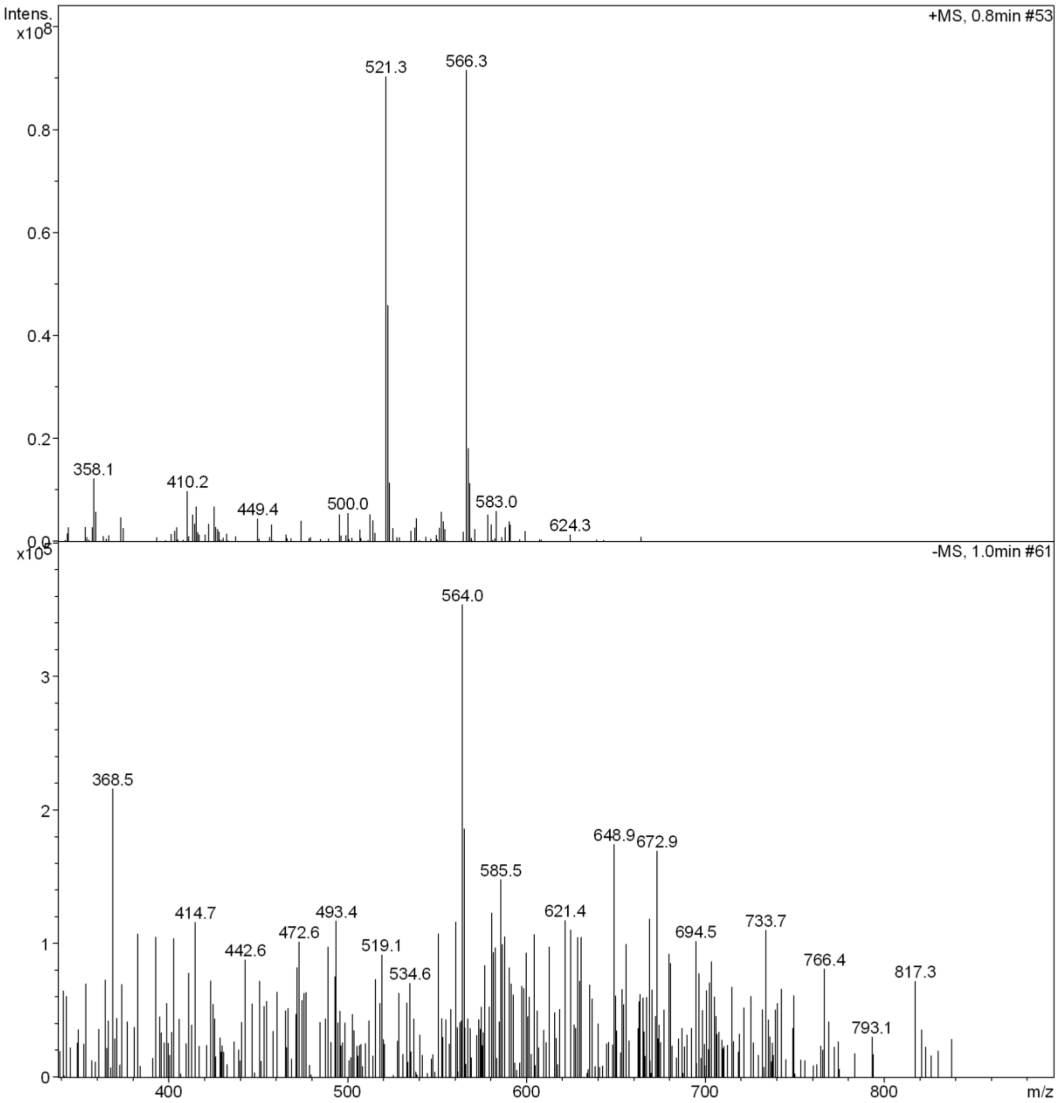

ESI-MS Spectrum of SU205

Direct Mass Spectrometry Analysis

Analysis Name: 23032510.d
Sample Name: SU301
Instrument: LC-MSD-Trap-SL
Operator: 21953830E000H12U
Print Date: 3/25/2023 10:42:19 AM
Acq. Date: 3/25/2023 10:39:36 AM

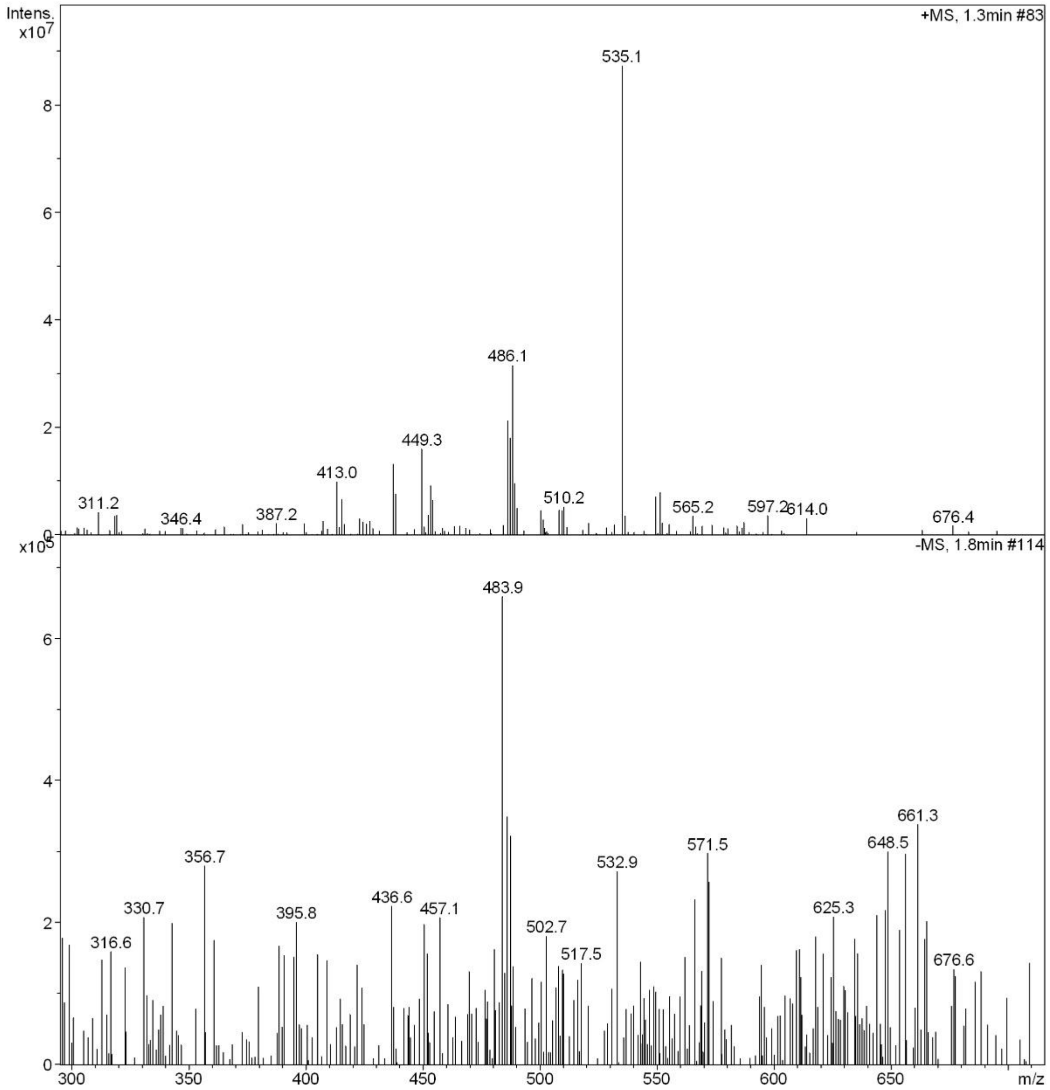

ESI-MS Spectrum of SU301

Direct Mass Spectrometry Analysis

Analysis Name: 23032517.d
Sample Name: SU302
Instrument: LC-MSD-Trap-SL
Operator: 21953830E000H12U
Print Date: 3/25/2023 11:01:17 AM
Acq. Date: 3/25/2023 10:57:10 AM

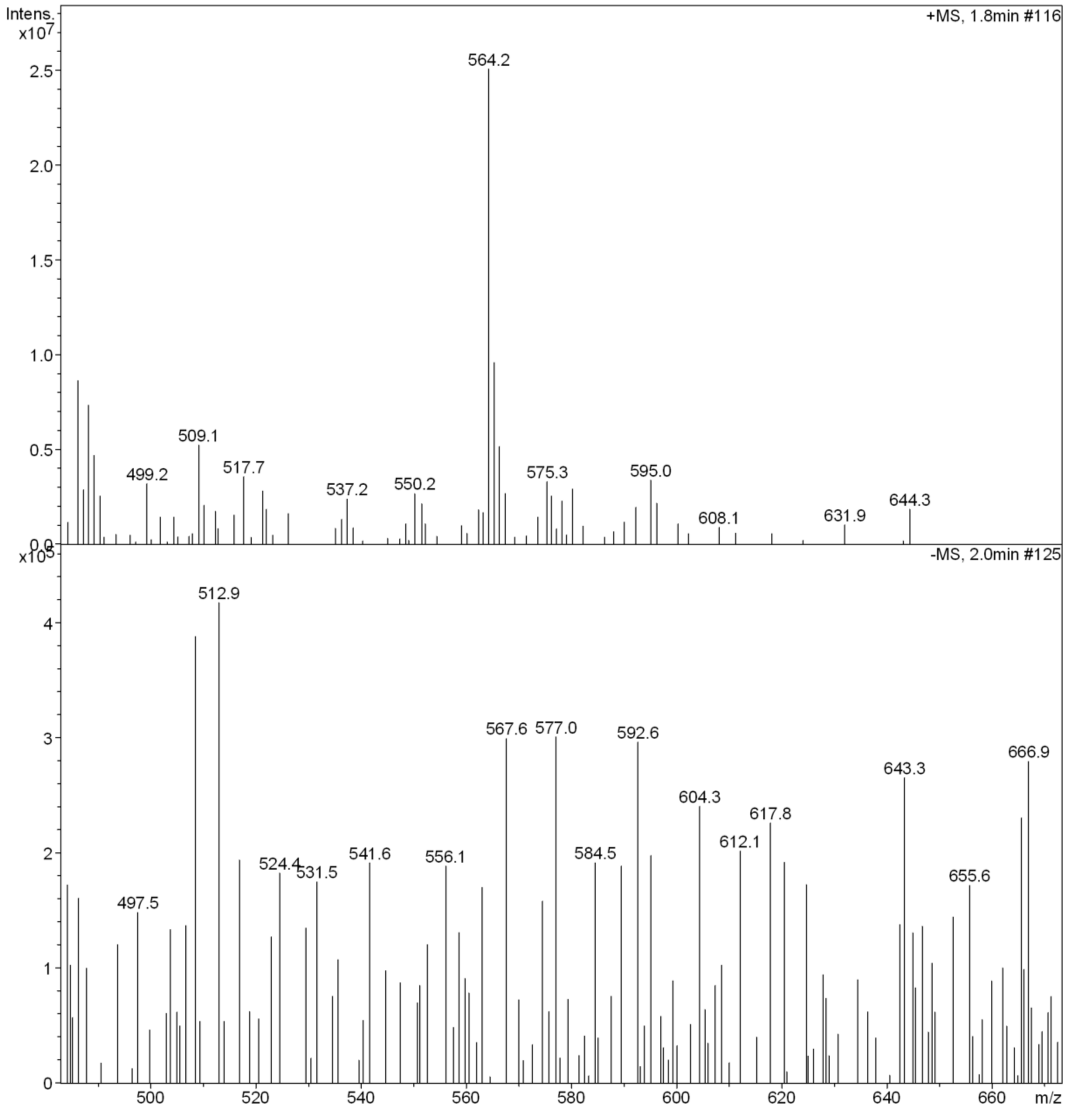

ESI-MS Spectrum of SU302

Direct Mass Spectrometry Analysis

Analysis Name: 23032513.d
Sample Name: SU303
Instrument: LC-MSD-Trap-SL
Operator: 21953830E000H12U
Print Date: 3/25/2023 10:49:14 AM
Acq. Date: 3/25/2023 10:47:39 AM

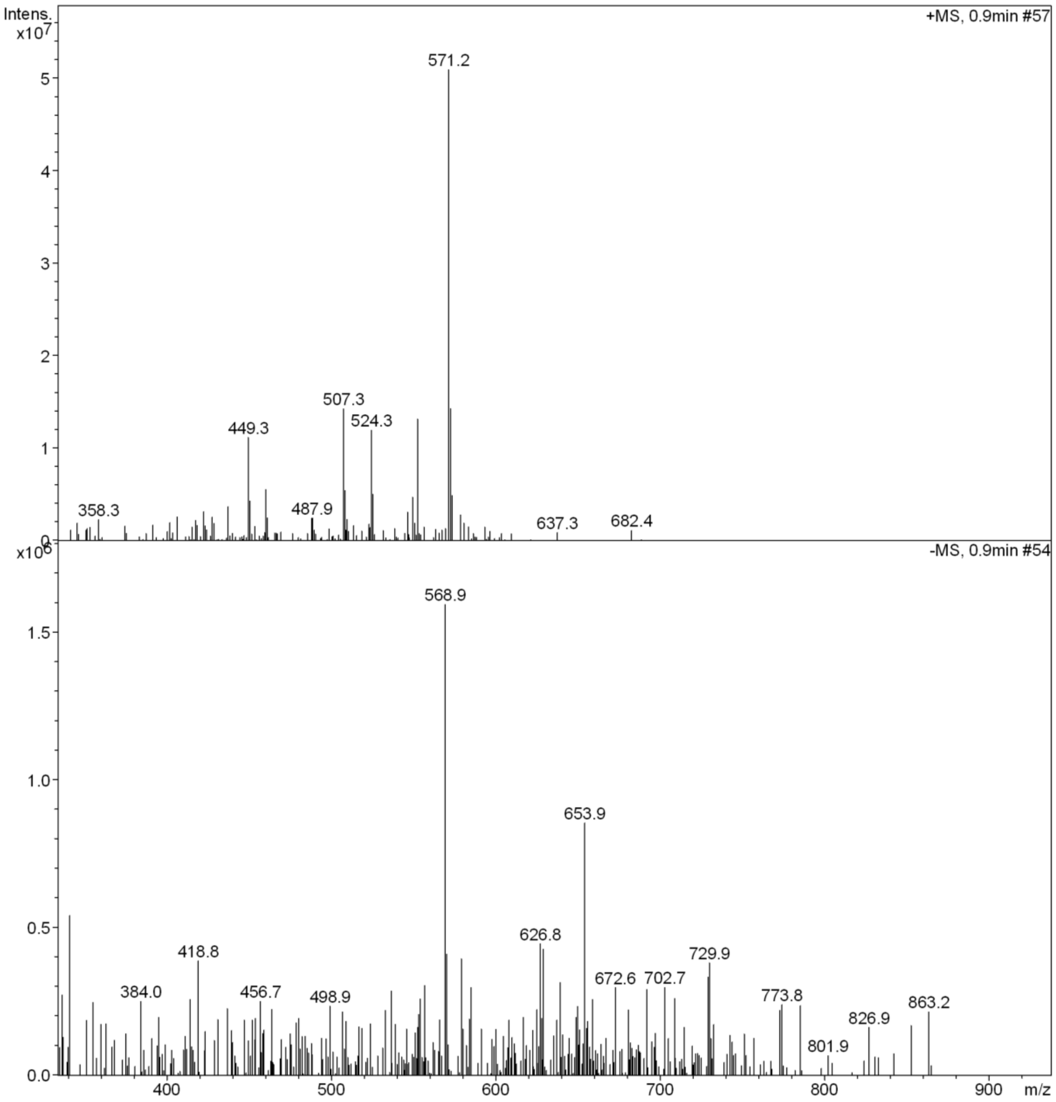

ESI-MS Spectrum of SU303

Direct Mass Spectrometry Analysis

Analysis Name: 23032519.d
Sample Name: SU304
Instrument: LC-MSD-Trap-SL
Operator: 21953830E000H12U
Print Date: 3/25/2023 11:06:52 AM
Acq. Date: 3/25/2023 11:05:38 AM

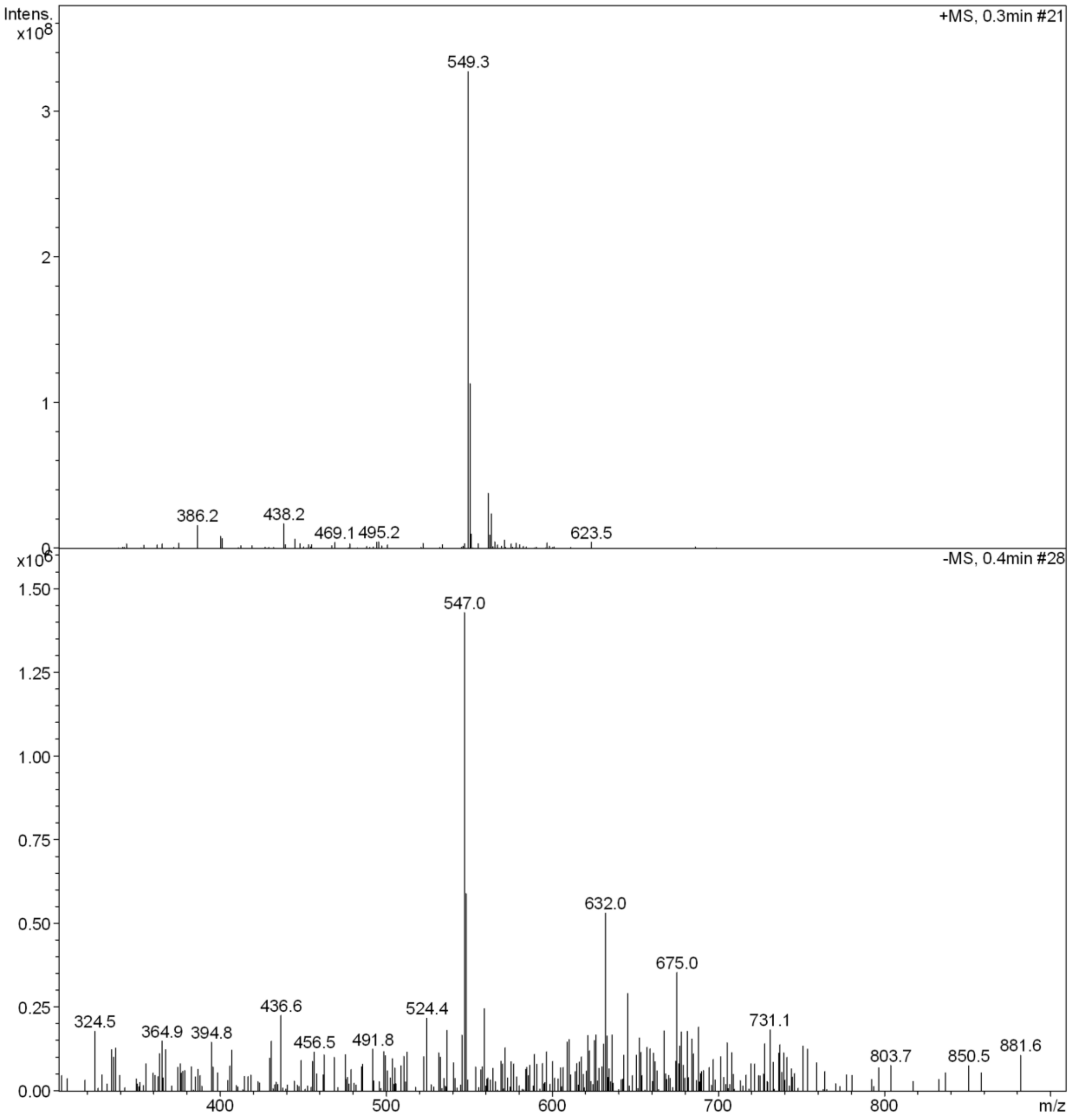

ESI-MS Spectrum of SU304

Direct Mass Spectrometry Analysis

Analysis Name: 23032512.d
Sample Name: SU305
Instrument: LC-MSD-Trap-SL
Operator: 21953830E000H12U
Print Date: 3/25/2023 10:46:52 AM
Acq. Date: 3/25/2023 10:45:05 AM

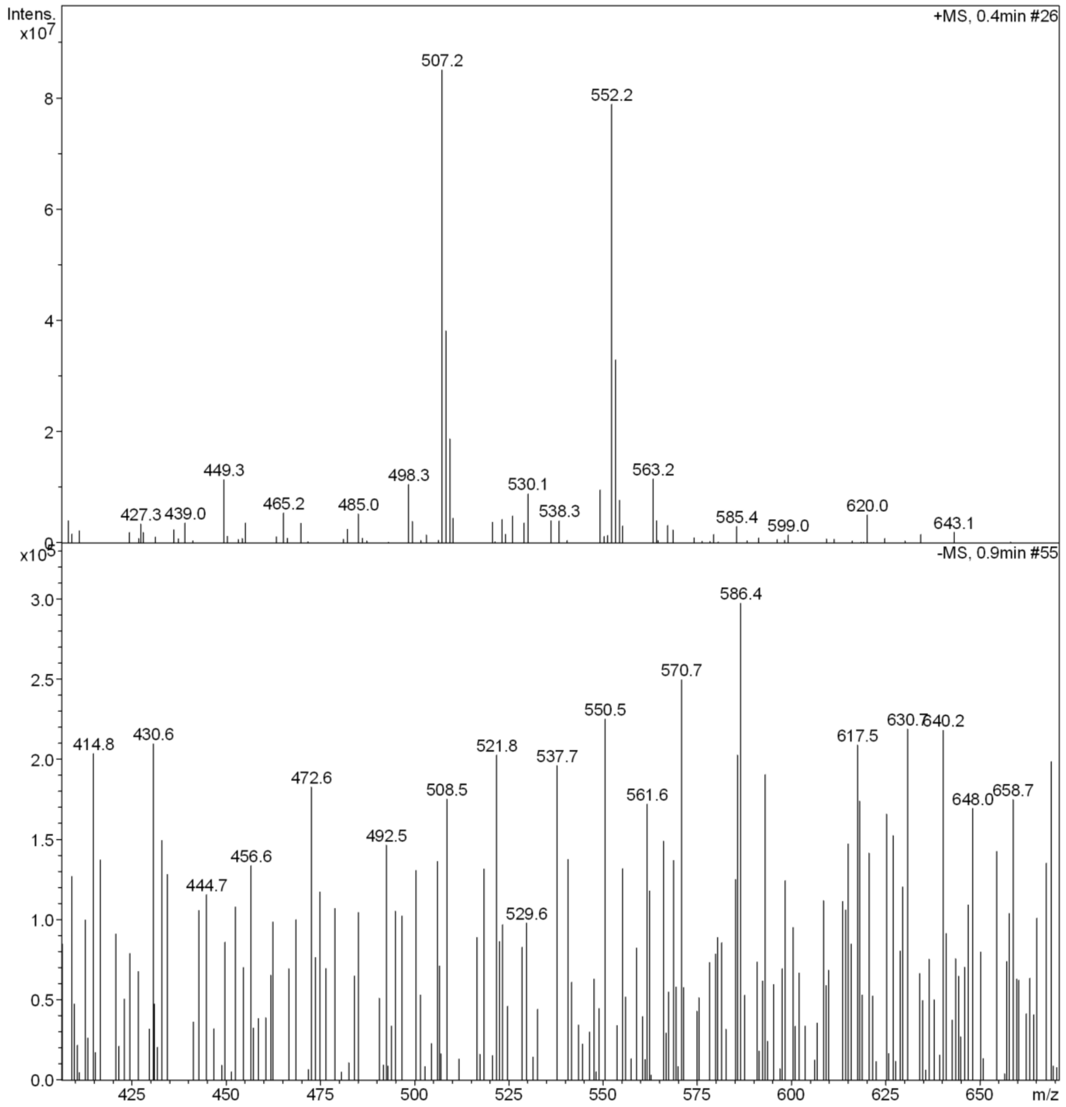

ESI-MS Spectrum of SU305

Direct Mass Spectrometry Analysis

Analysis Name: 23032518.d | **Instrument:** LC-MSD-Trap-SL | **Print Date:** 3/25/2023 11:04:42 AM
Sample Name: SU401 | **Operator:** 21953830E000H12U | **Acq. Date:** 3/25/2023 11:02:08 AM

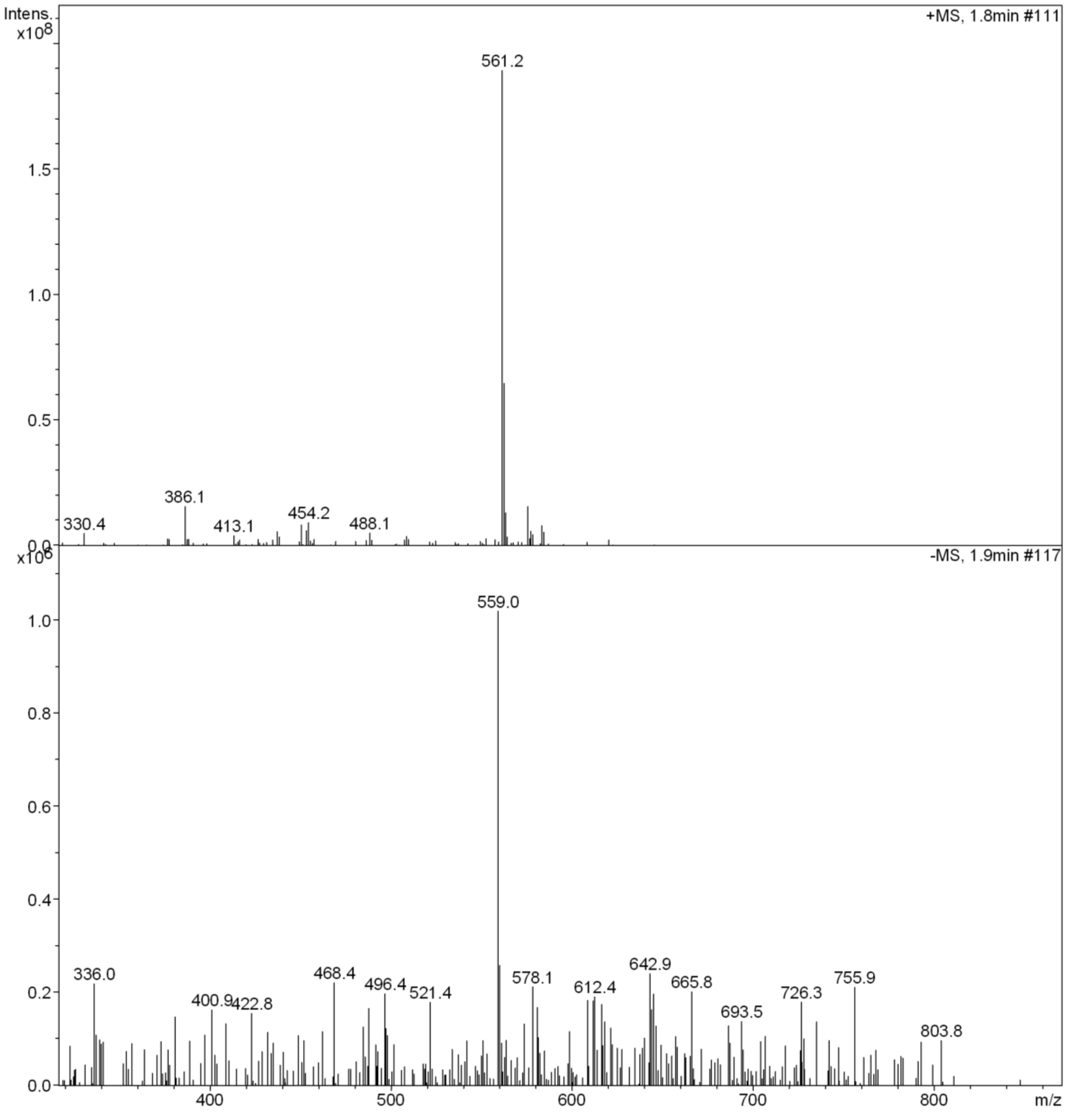

ESI-MS Spectrum of SU401

Direct Mass Spectrometry Analysis

Analysis Name: 23032514.d
Sample Name: SU402
Instrument: LC-MSD-Trap-SL
Operator: 21953830E000H12U
Print Date: 3/25/2023 10:51:04 AM
Acq. Date: 3/25/2023 10:50:03 AM

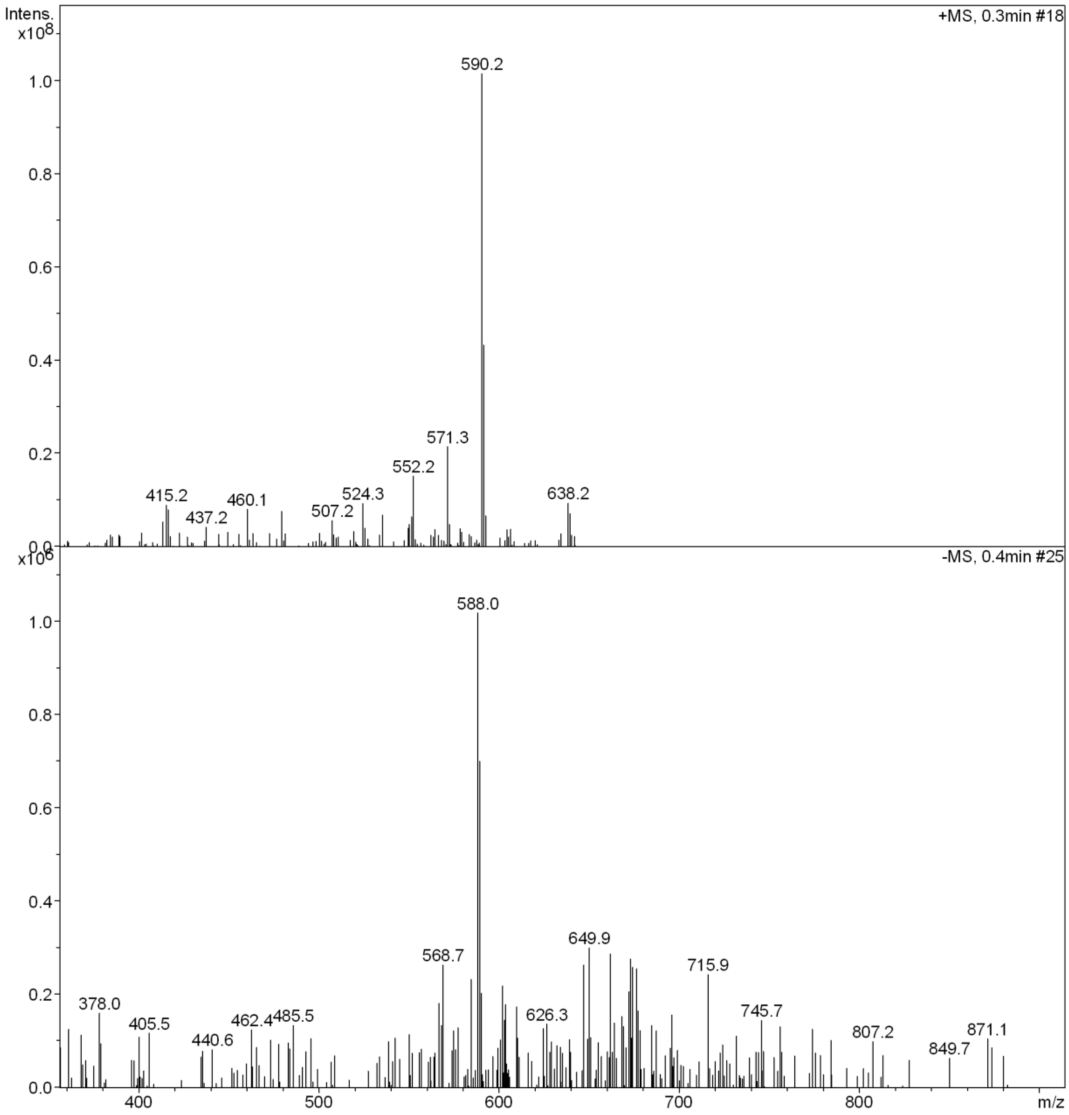

ESI-MS Spectrum of SU402

Direct Mass Spectrometry Analysis

Analysis Name: 23032516.d
Sample Name: SU403
Instrument: LC-MSD-Trap-SL
Operator: 21953830E000H12U
Print Date: 3/25/2023 10:56:22 AM
Acq. Date: 3/25/2023 10:55:08 AM

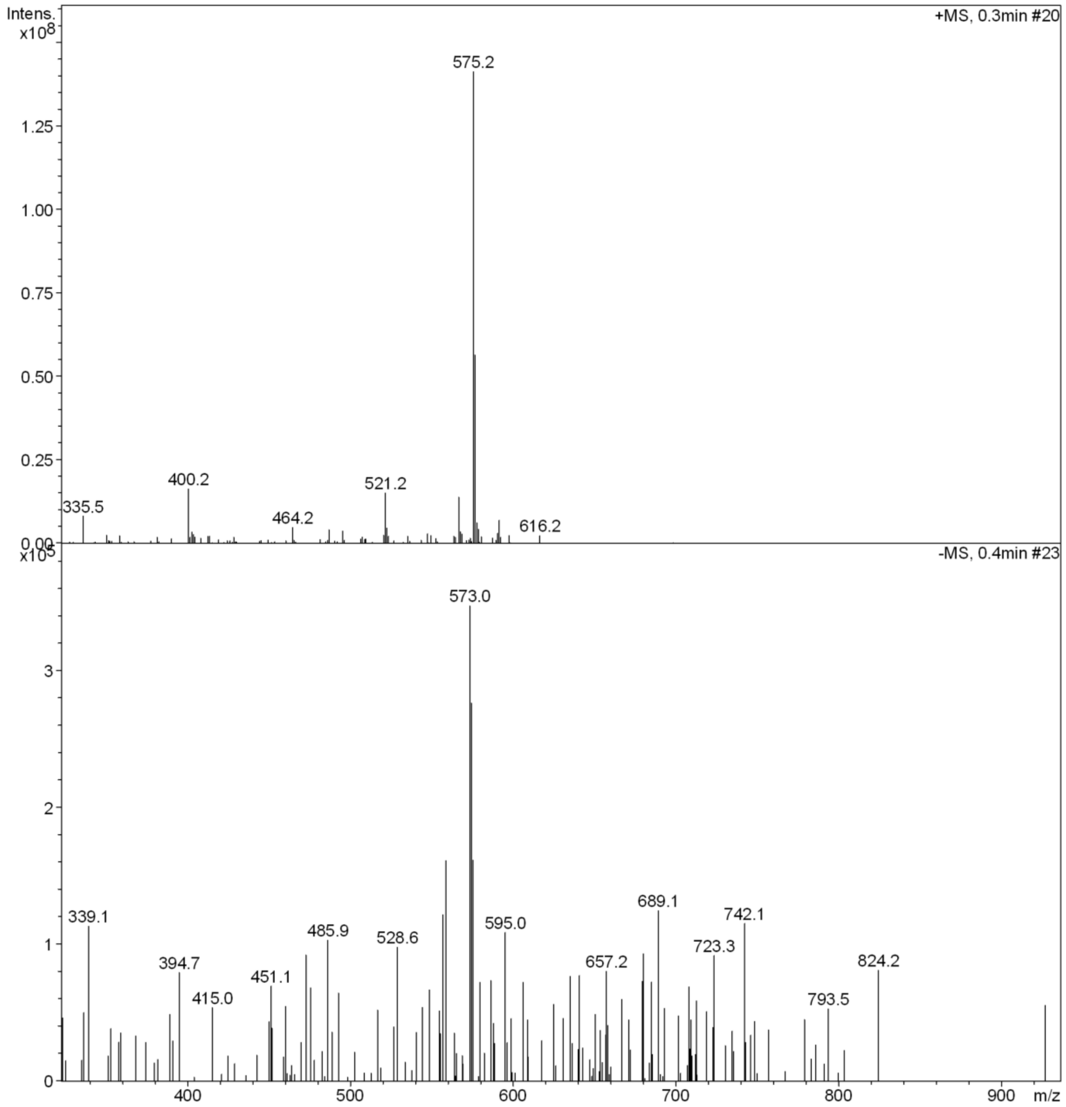

ESI-MS Spectrum of SU403

Direct Mass Spectrometry Analysis

Analysis Name: 23032520.d
Sample Name: SU404
Instrument: LC-MSD-Trap-SL
Operator: 21953830E000H12U
Print Date: 3/25/2023 11:09:31 AM
Acq. Date: 3/25/2023 11:07:34 AM

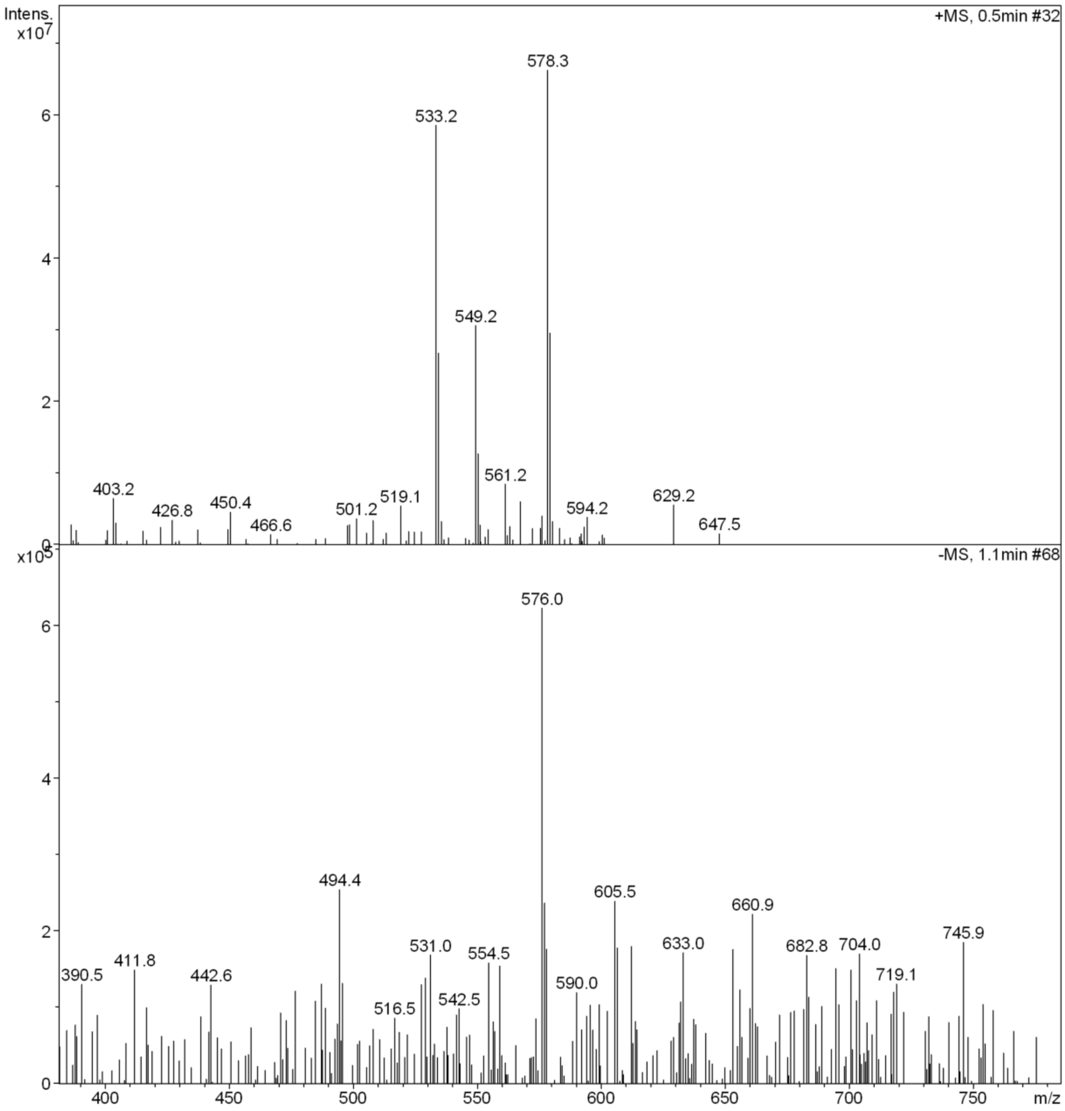

ESI-MS Spectrum of SU404

Print Window : MS Spectrum
Data : E:\DATA\DEF_LC1 2021-11-03 21-21-15\020-0201.D
Sample Name : UI-1

Operator	:	Sequence Line	: 2
Instrument	: Instrument 1	Position	: 20
Injection Date	: 2021/11/3 21:30:38	Injection Count	: 1
		Injection Volume	: 5.000 µl

Acquisition Method : E:\DATA\DEF_LC1 2021-11-03 21-21-15\100to1000P_AND_N.M
Last Modified : 2021/4/17 14:18:09
Analysis Method : C:\CHEM32\1\METHODS\100TO500_POS_AND_NEG.M
Last Modified : 2021/11/4 8:42:18

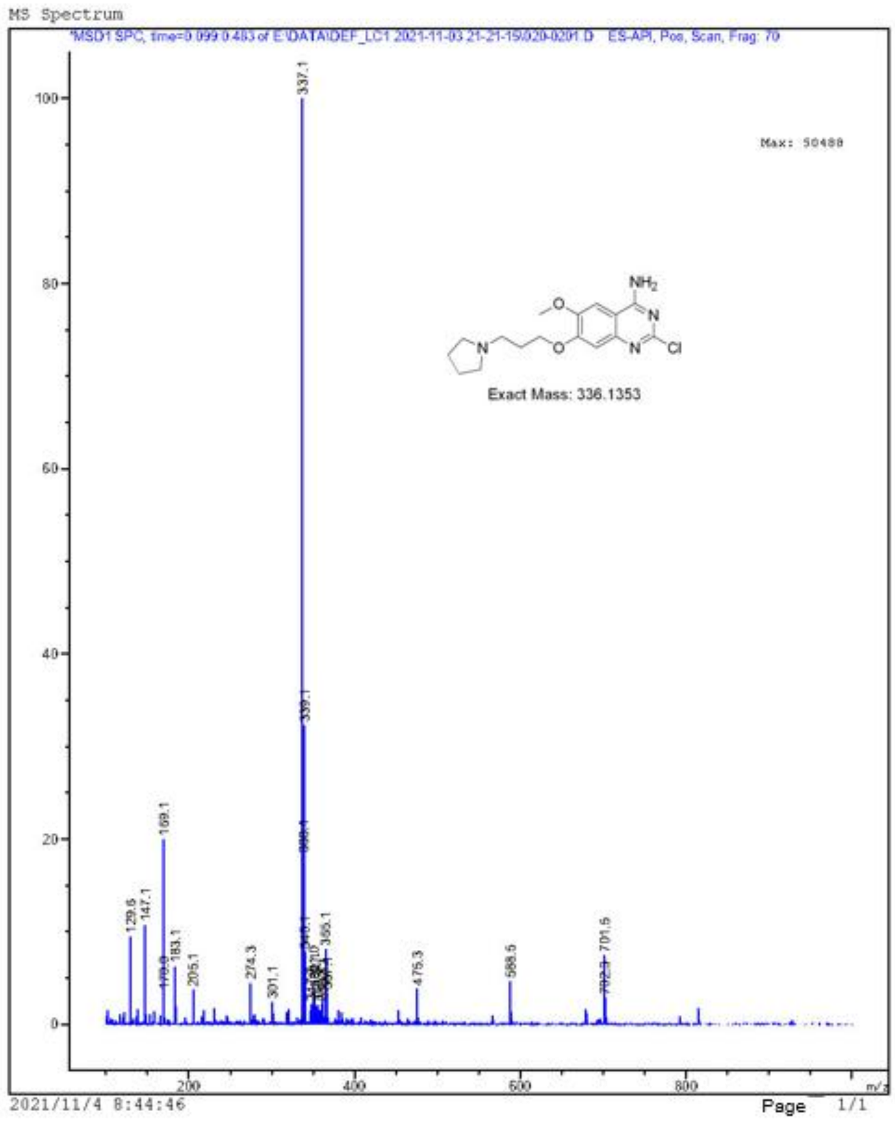

ESI-MS Spectrum of 11

Print Window : MS Spectrum
Data : E:\DATA\DEF_LC1 2022-03-24 22-07-27\014-0401.D
Sample Name : UA-F

==

Operator	:	Sequence Line	: 4
Instrument	Instrument 1	Position	: 14
Injection Date	: 2022/3/24 22:31:42	Injection Count	: 1
		Injection Volume	: 5.000 µl

Acquisition Method : E:\DATA\DEF_LC1 2022-03-24 22-07-27\100TO1000P_AND_N.M
Last Modified : 2021/4/17 14:18:09
Analysis Method : C:\CHEM32\1\METHODS\100TO500_POS_AND_NEG.M
Last Modified : 2022/3/24 8:55:08

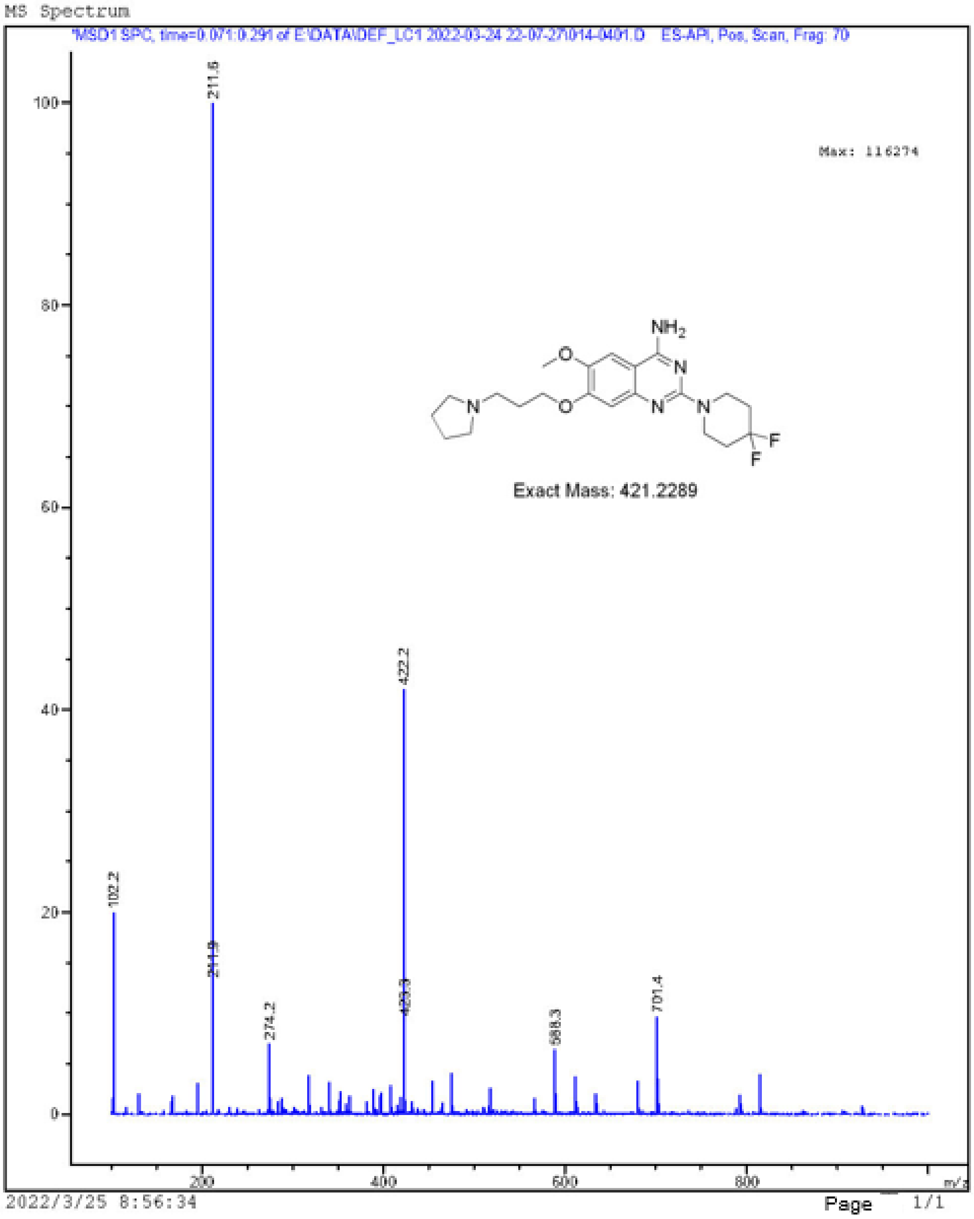

2022/3/25 8:56:34 Page 1/1

ESI-MS Spectrum of 12b

Direct Mass Spectrometry Analysis

Analysis Name: 20221146.d
Sample Name: PN-2
Instrument: LC-MSD-Trap-SL
Operator: 21953830E000H12U
Print Date: 11/16/202 7:43:01 PM
Acq. Date: 21/16/2022 7:41:13 PM

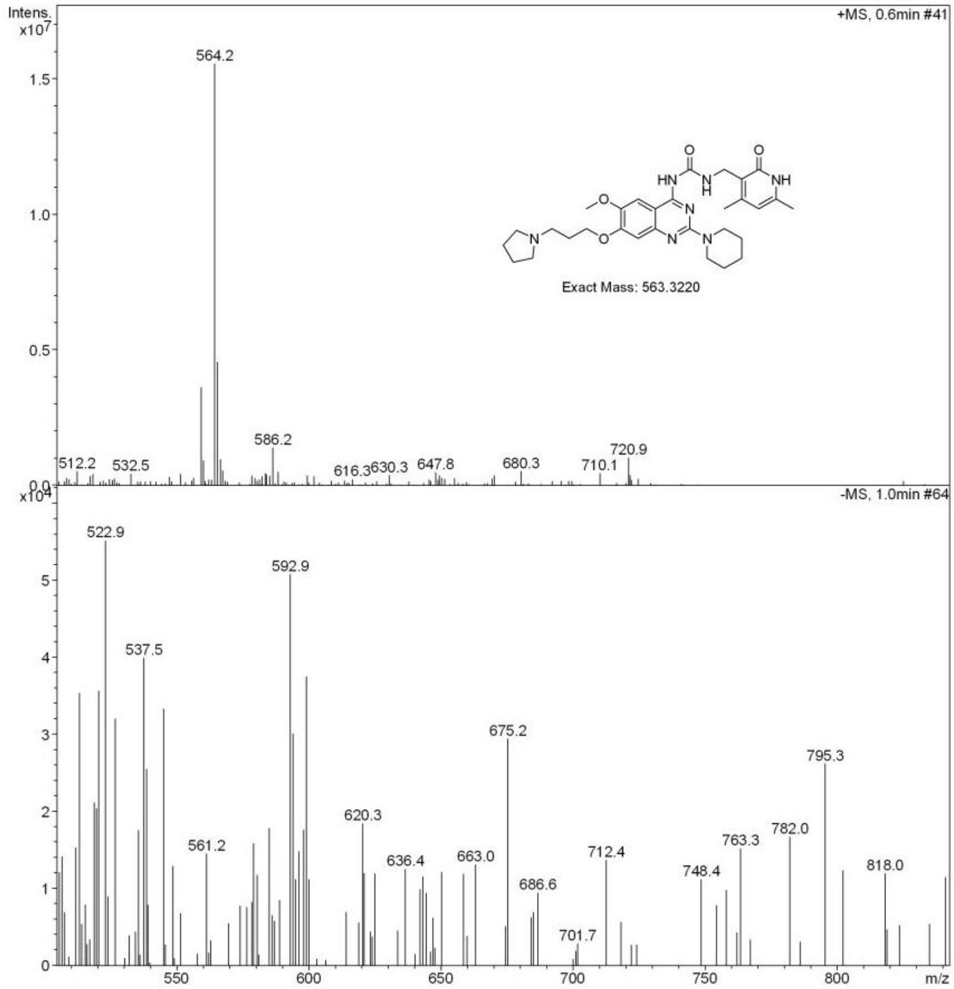

ESI-MS Spectrum of UA01

Direct Mass Spectrometry Analysis

Analysis Name: 23032491.d
Sample Name: UA02
Instrument: LC-MSD-Trap-SL
Operator: 21953830E000H12U
Print Date: 3/24/2023 8:31:07 PM
Acq. Date: 3/24/2023 8:29:44 PM

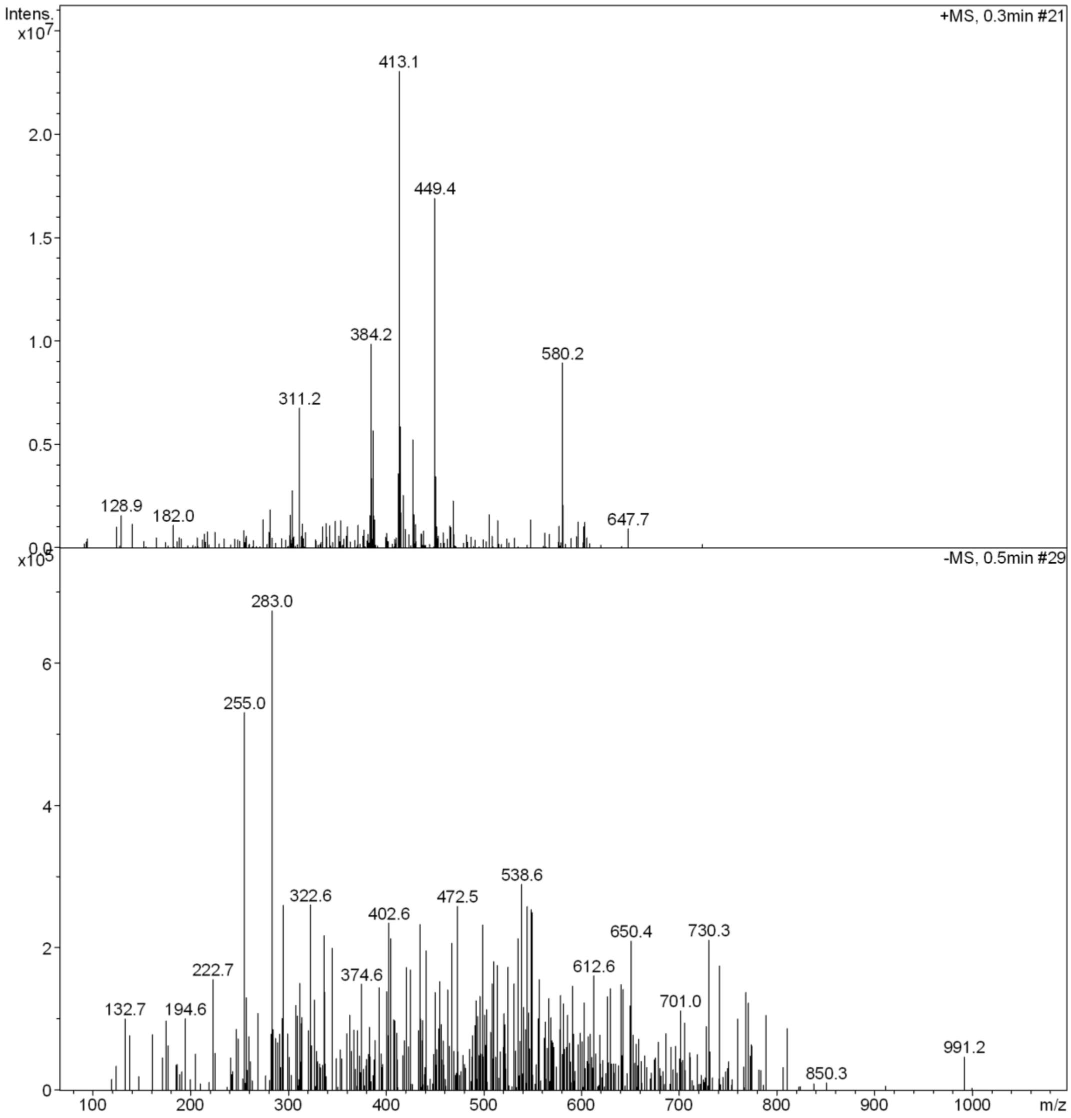

ESI-MS Spectrum of UA02

Direct Mass Spectrometry Analysis

Analysis Name: 23032492.d
Sample Name: UA03
Instrument: LC-MSD-Trap-SL
Operator: 21953830E000H12U
Print Date: 3/24/2023 8:32:45 PM
Acq. Date: 3/24/2023 8:31:35 PM

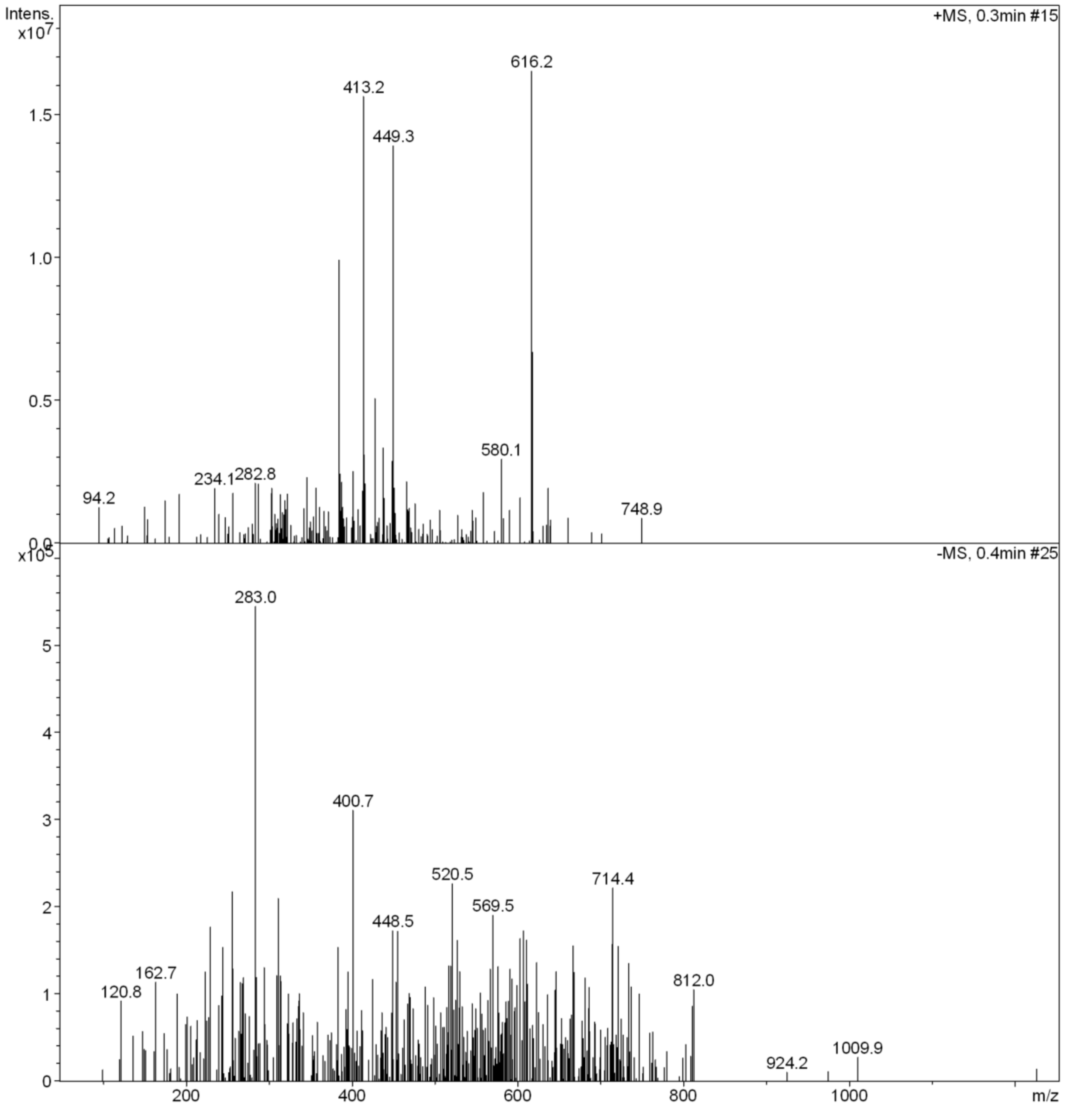

ESI-MS Spectrum of UA03

Print Window : MS Spectrum
Data : E:\DATA\DEF_LC1 2022-04-25 21-20-05\041-3101.D
Sample Name : DBO

Operator :
Instrument : Instrument 1
Injection Date : 2022/4/26 0:39:15
Sequence Line : 31
Position : 41
Injection Count : 1
Injection Volume : 5.000 µl
Acquisition Method : E:\DATA\DEF_LC1 2022-04-25 21-2[illegible]1000P_AND_N.M
Last Modified : 2021/4/17 14:18:09
Analysis Method : C:\CHEM32\1\METHODS\100TO500_POS_AND_NEG.M
Last Modified : 2022/4/23 15:52:39

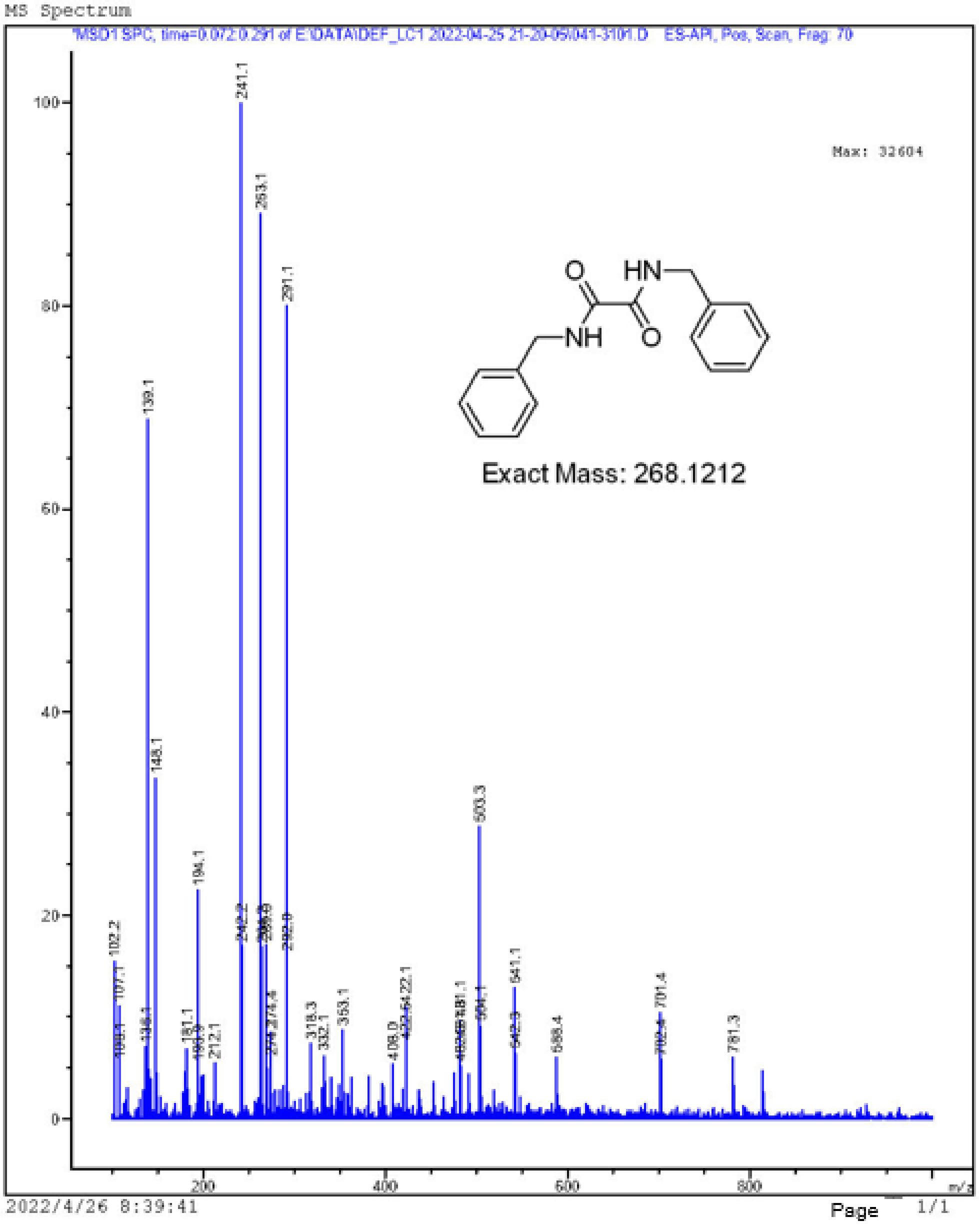

ESI-MS Spectrum of DBO

Print Window : MS Spectrum
Data : E:\DATA\DEF_LC1 2022-02-22 11-02-02\097-4501.D
Sample Name : UII-1

Operator :		Sequence Line :	45
Instrument :	Instrument 1	Position :	97
Injection Date :	2022/2/22 15:53:01	Injection Count :	1
		Injection Volume :	5.000 µl

Acquisition Method : E:\DATA\DEF_LC1 2022-02-22 11-02-02\100TO1000P_AND_N.M
Last Modified : 2021/4/17 14:18:09
Analysis Method : C:\CHEM32\1\METHODS\100TO500_POS_AND_NEG.M
Last Modified : 2022/2/22 17:35:39

MS Spectrum

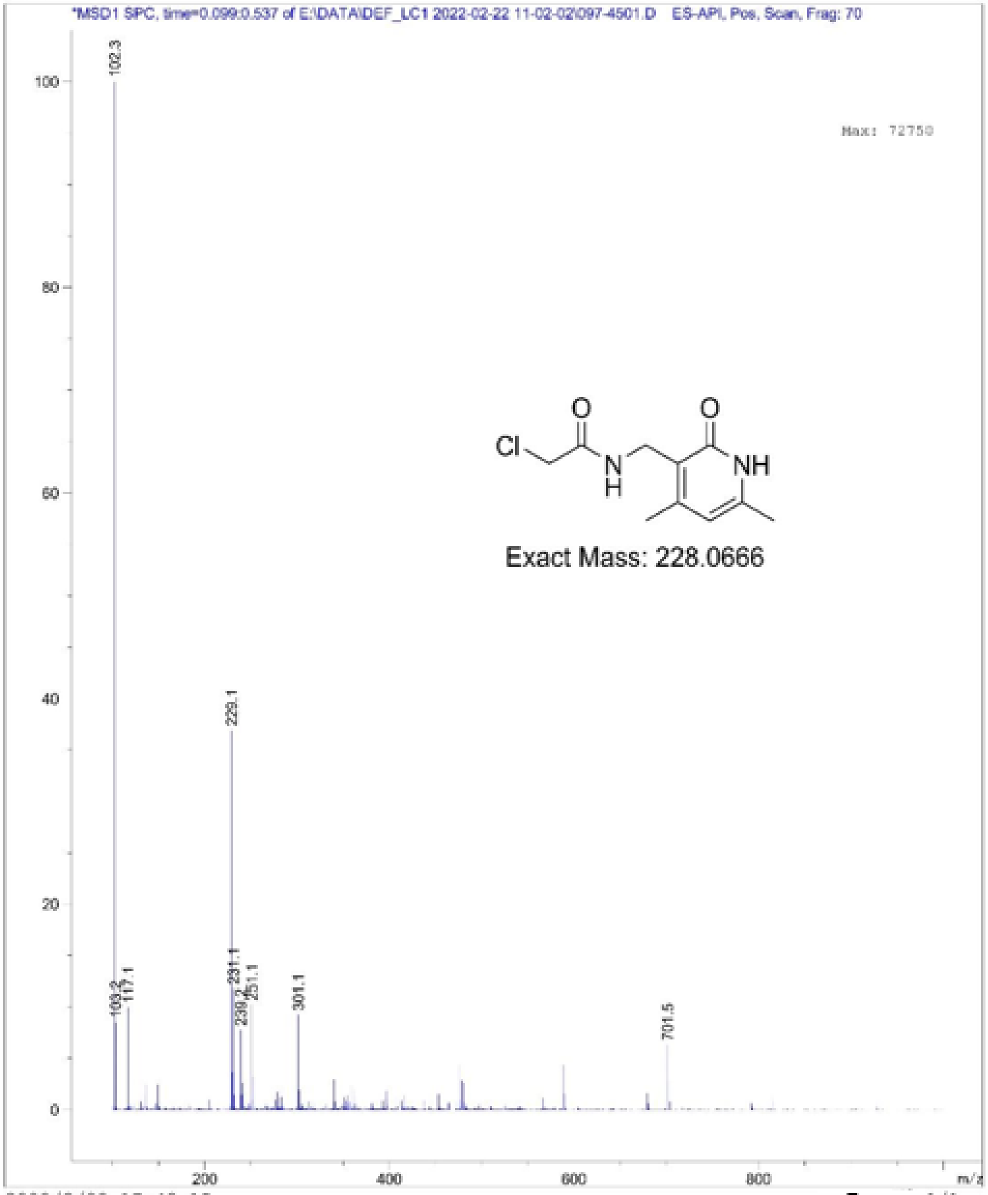

2022/2/22 17:42:15 Page 1/1

ESI-MS Spectrum of 16

Print Window : MS Spectrum
Data : E:\DATA\DEF_LC1 2022-05-19 00-56-53\019-0901.D
Sample Name : UB-3
===

Operator	:	Sequence Line	: 9
Instrument	: Instrument 1	Position	: 19
Injection Date	: 2022/5/19 1:52:07	Injection Count	: 1
		Injection Volume	: 5.000 µl

Acquisition Method : E:\DATA\DEF_LC1 2022-05-19 00-56-53\100TO1000P_AND_N.M
Last Modified : 2021/4/17 14:18:09
Analysis Method : C:\CHEM32\1\METHODS\100TO500_POS_AND_NEG.M
Last Modified : 2022/5/18 9:29:55

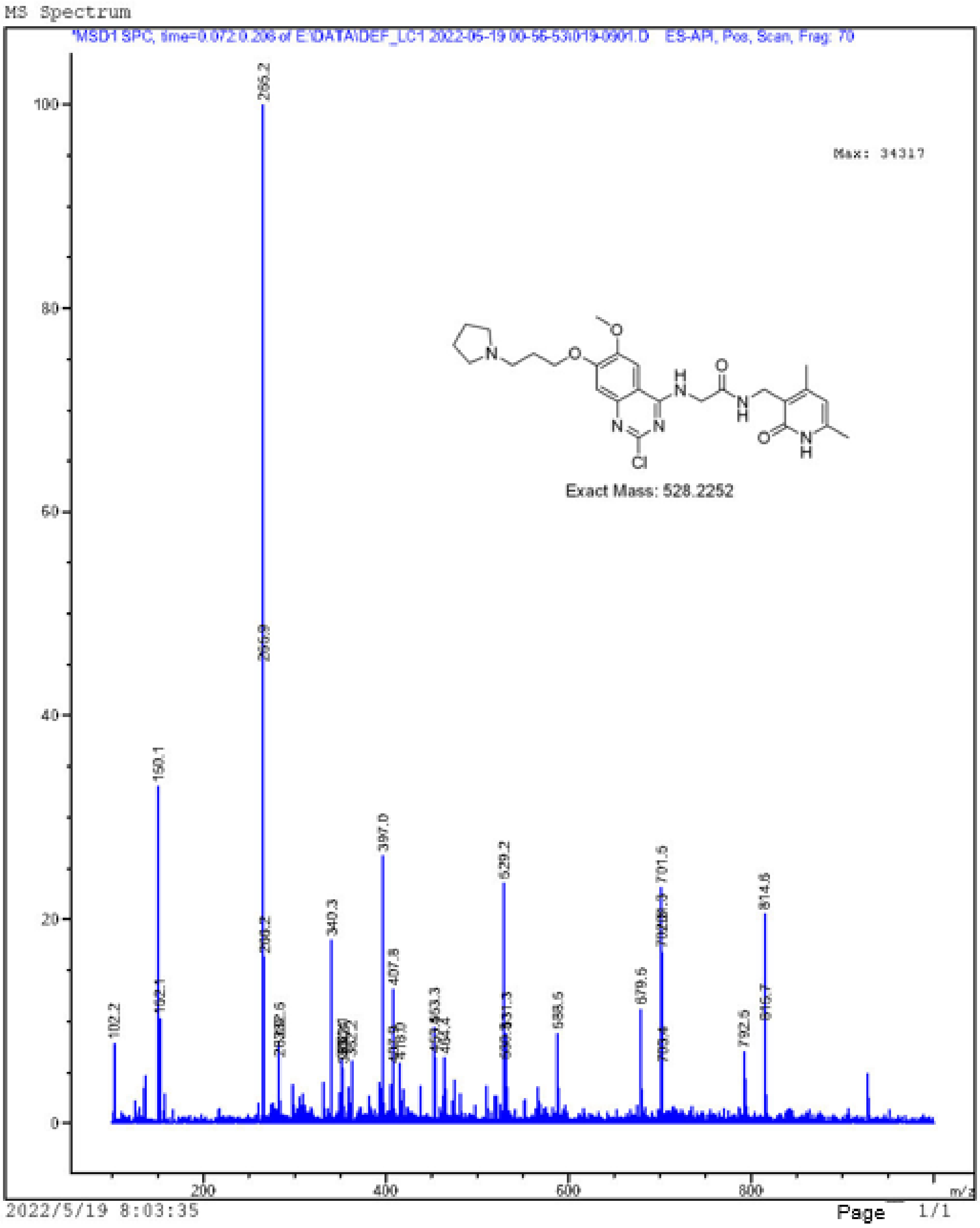

2022/5/19 8:03:35 Page 1/1

ESI-MS Spectrum of 17

Direct Mass Spectrometry Analysis

Analysis Name: 23032493.d
Sample Name: UB01
Instrument: LC-MSD-Trap-SL
Operator: 21953830E000H12U
Print Date: 3/24/2023 8:35:20 PM
Acq. Date: 3/24/2023 8:33:37 PM

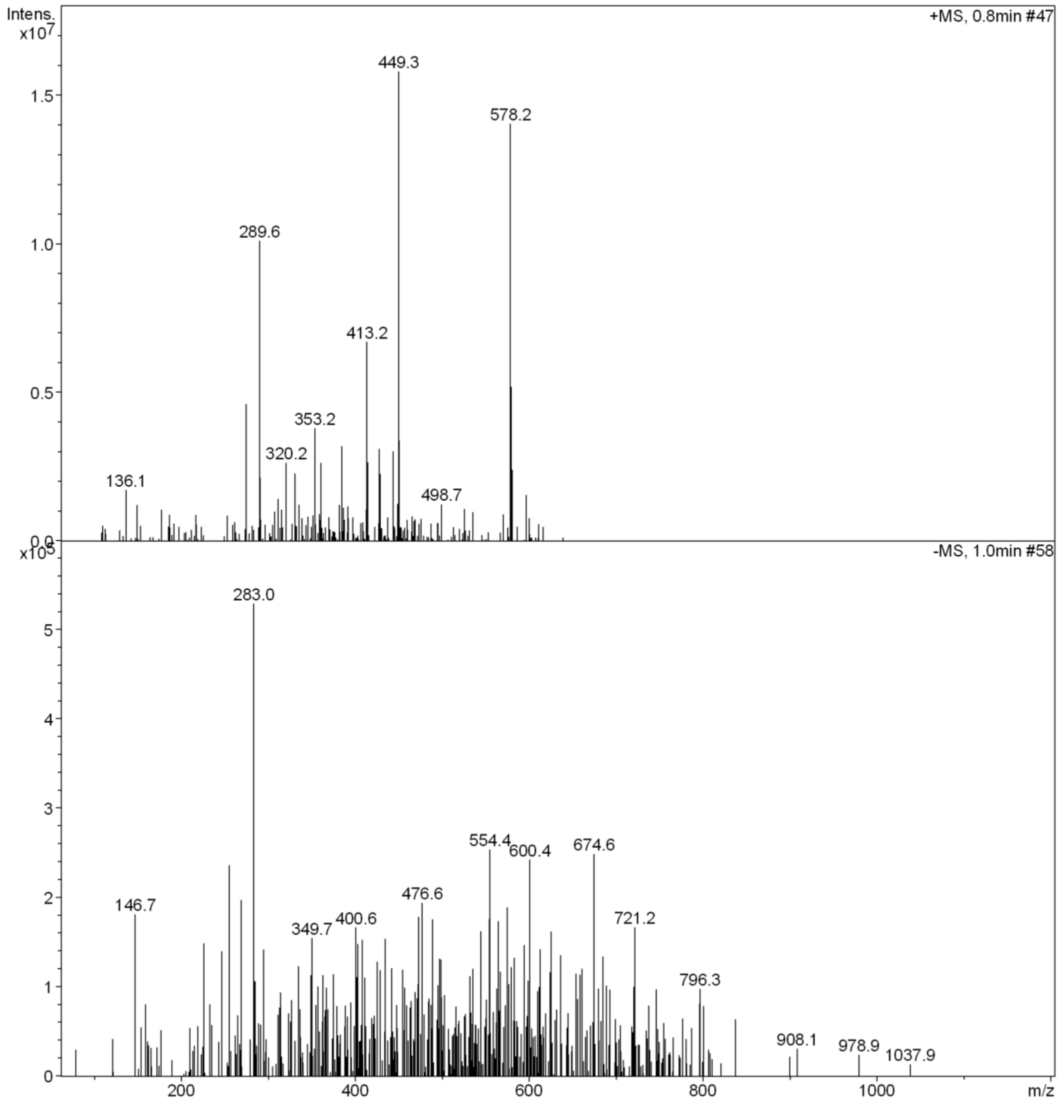

ESI-MS Spectrum of UB01

Direct Mass Spectrometry Analysis

Analysis Name: 23032494.d
Sample Name: UB02
Instrument: LC-MSD-Trap-SL
Operator: 21953830E000H12U
Print Date: 3/24/2023 8:37:27 PM
Acq. Date: 3/24/2023 8:35:46 PM

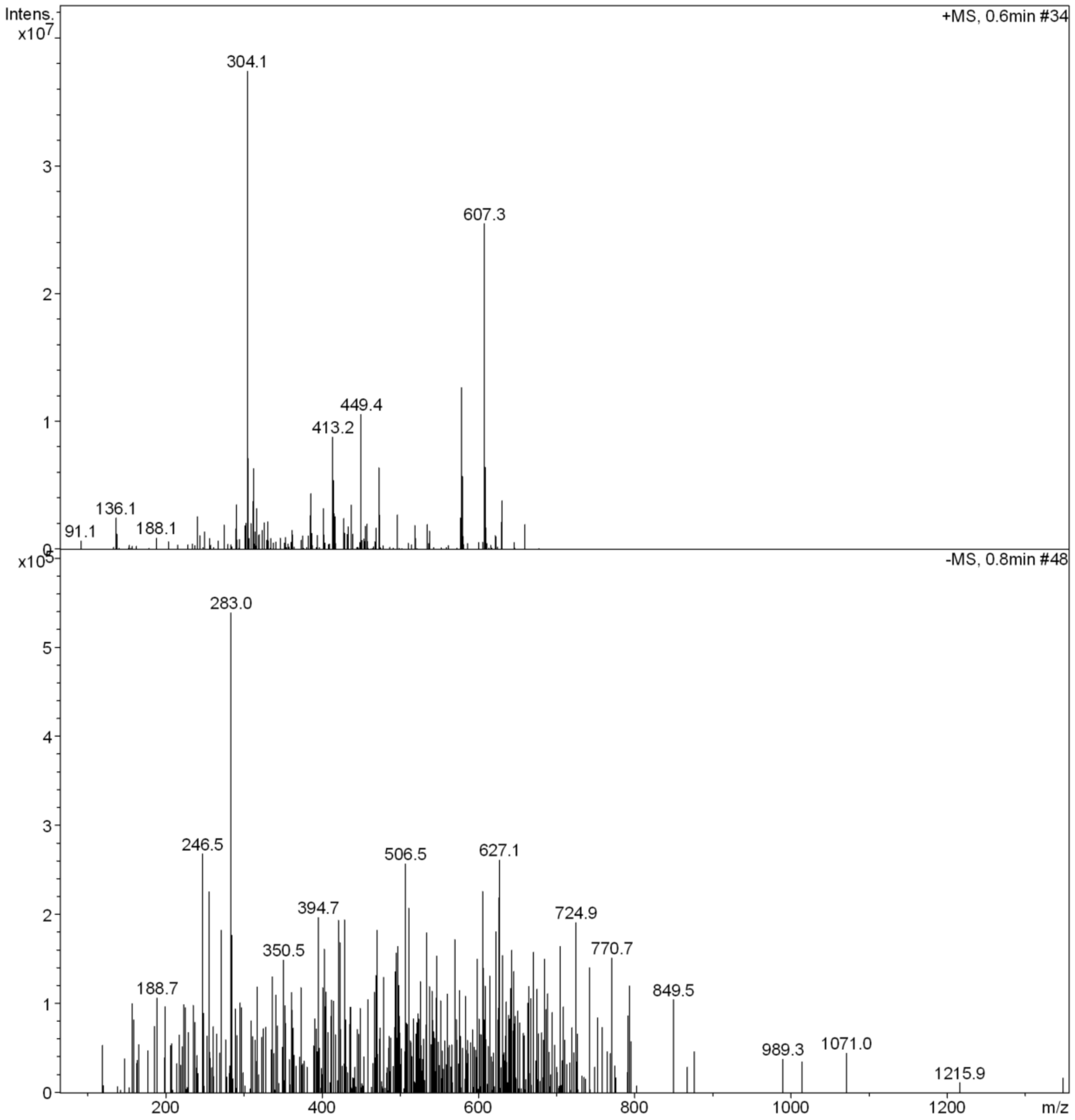

ESI-MS Spectrum of UB02

Direct Mass Spectrometry Analysis

Analysis Name: 23032495.d
Sample Name: UB03
Instrument: LC-MSD-Trap-SL
Operator: 21953830E000H12U
Print Date: 3/24/2023 8:39:29 PM
Acq. Date: 3/24/2023 8:37:54 PM

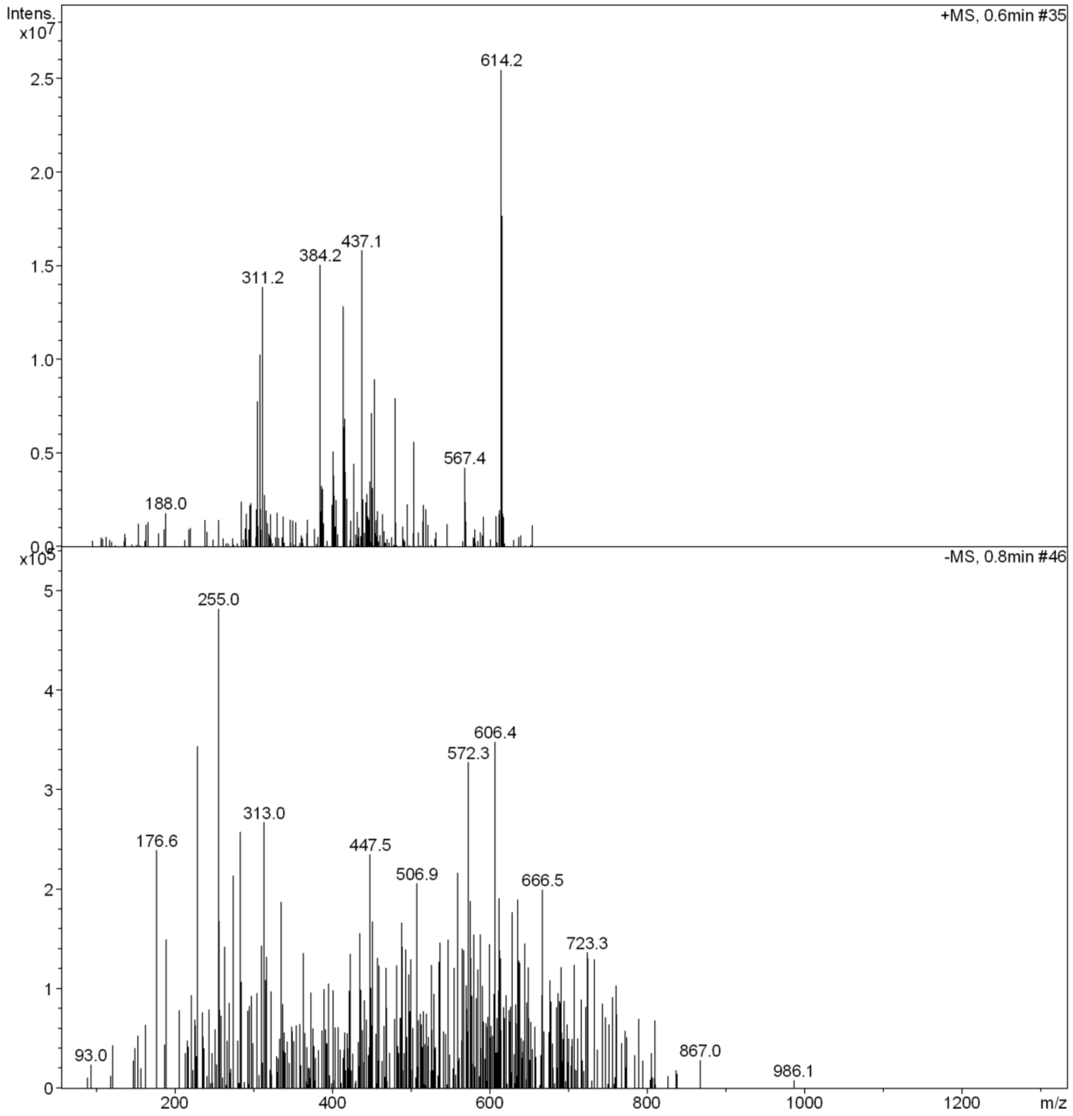

ESI-MS Spectrum of UB03

Direct Mass Spectrometry Analysis

Analysis Name: 23032500.d **Instrument:** LC-MSD-Trap-SL **Print Date:** 3/25/2023 10:11:54 AM
Sample Name: UB04 **Operator:** 21953830E000H12U **Acq. Date:** 3/25/2023 10:09:47 AM

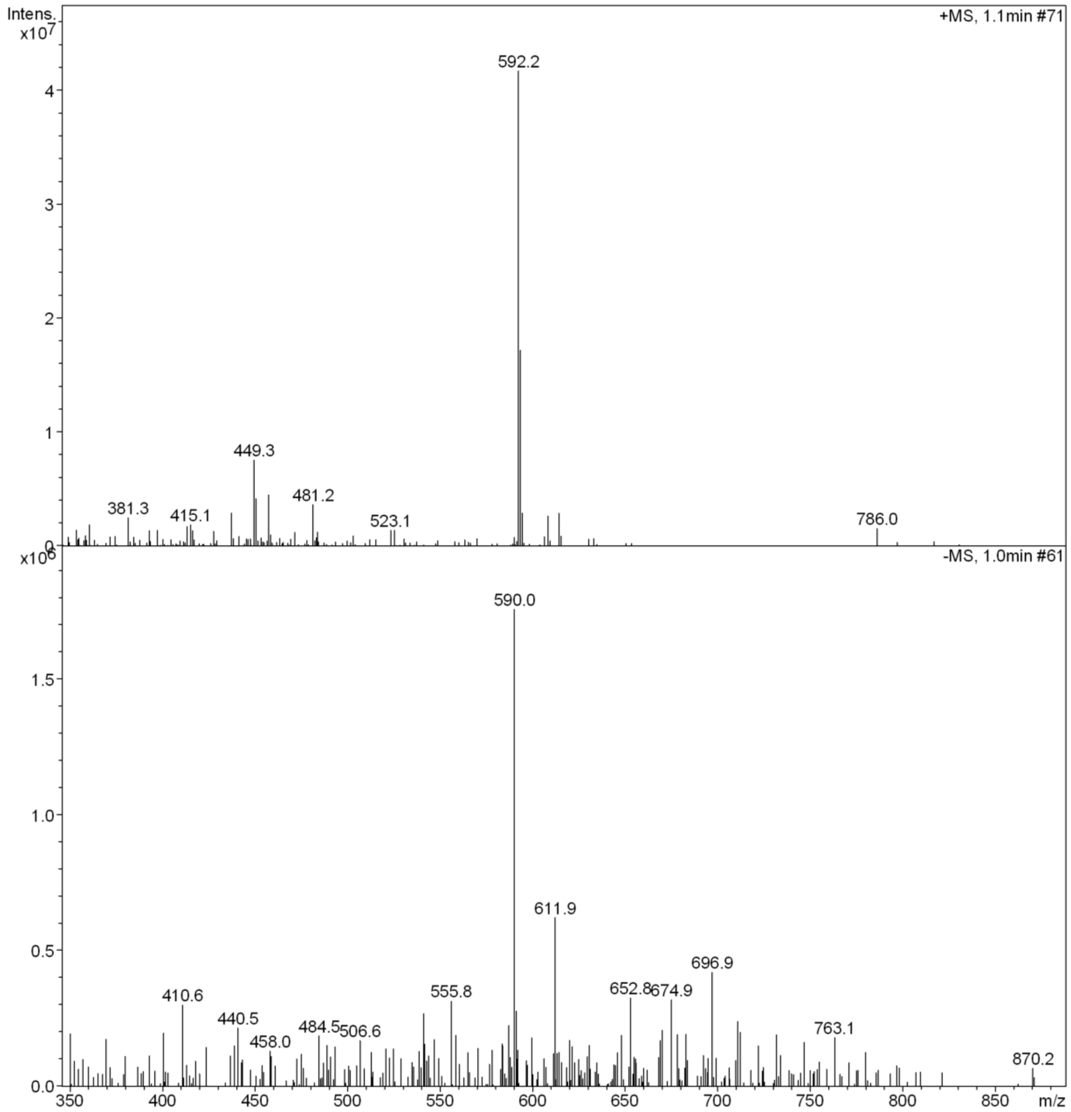

ESI-MS Spectrum of UB04

Direct Mass Spectrometry Analysis

Analysis Name: 23032504.d
Sample Name: UB05
Instrument: LC-MSD-Trap-SL
Operator: 21953830E000H12U
Print Date: 3/25/2023 10:22:56 AM
Acq. Date: 3/25/2023 10:21:24 AM

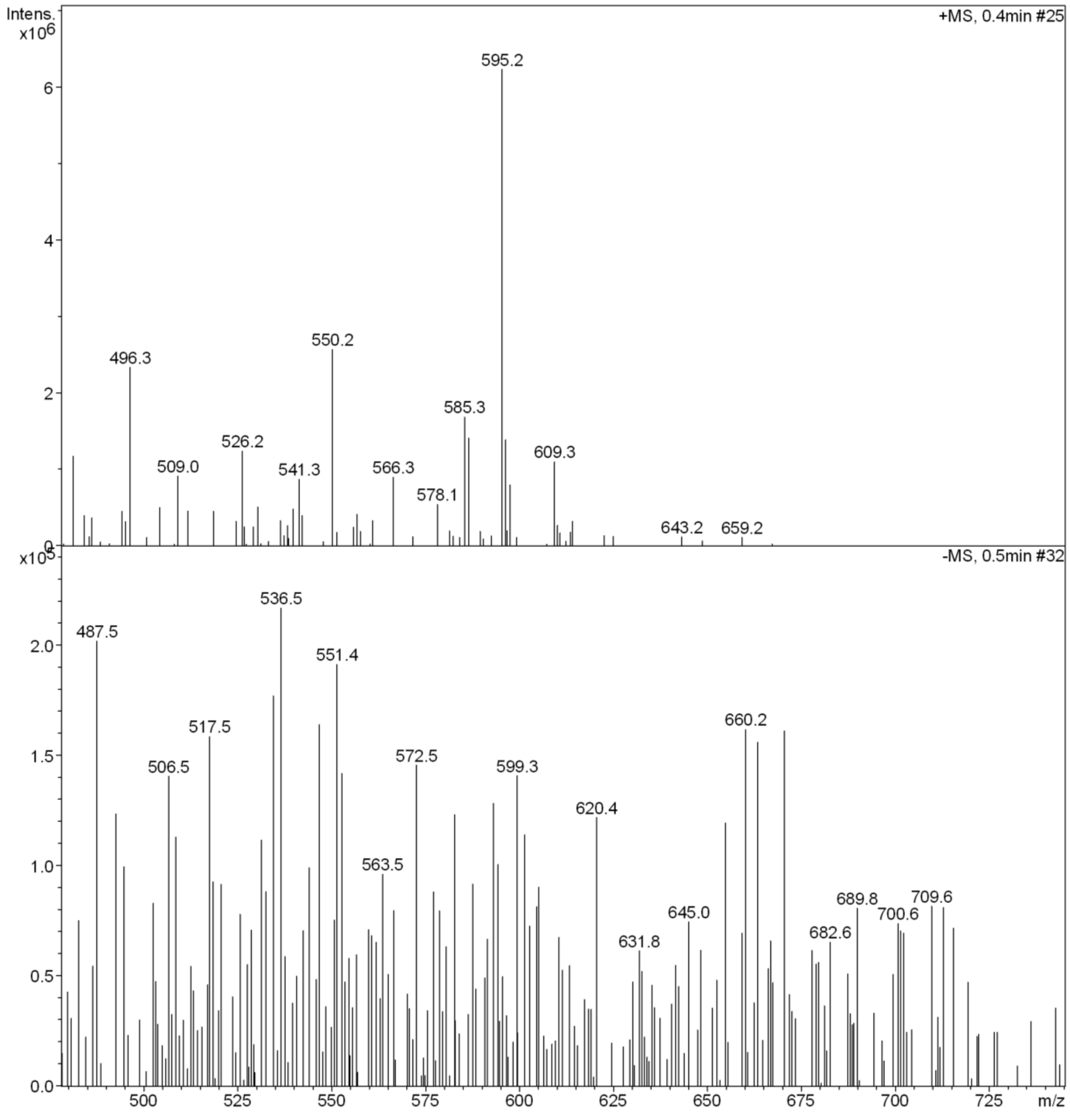

ESI-MS Spectrum of UB05

Direct Mass Spectrometry Analysis

Analysis Name: 23032502.d
Sample Name: UB06
Instrument: LC-MSD-Trap-SL
Operator: 21953830E000H12U
Print Date: 3/25/2023 10:16:52 AM
Acq. Date: 3/25/2023 10:14:58 AM

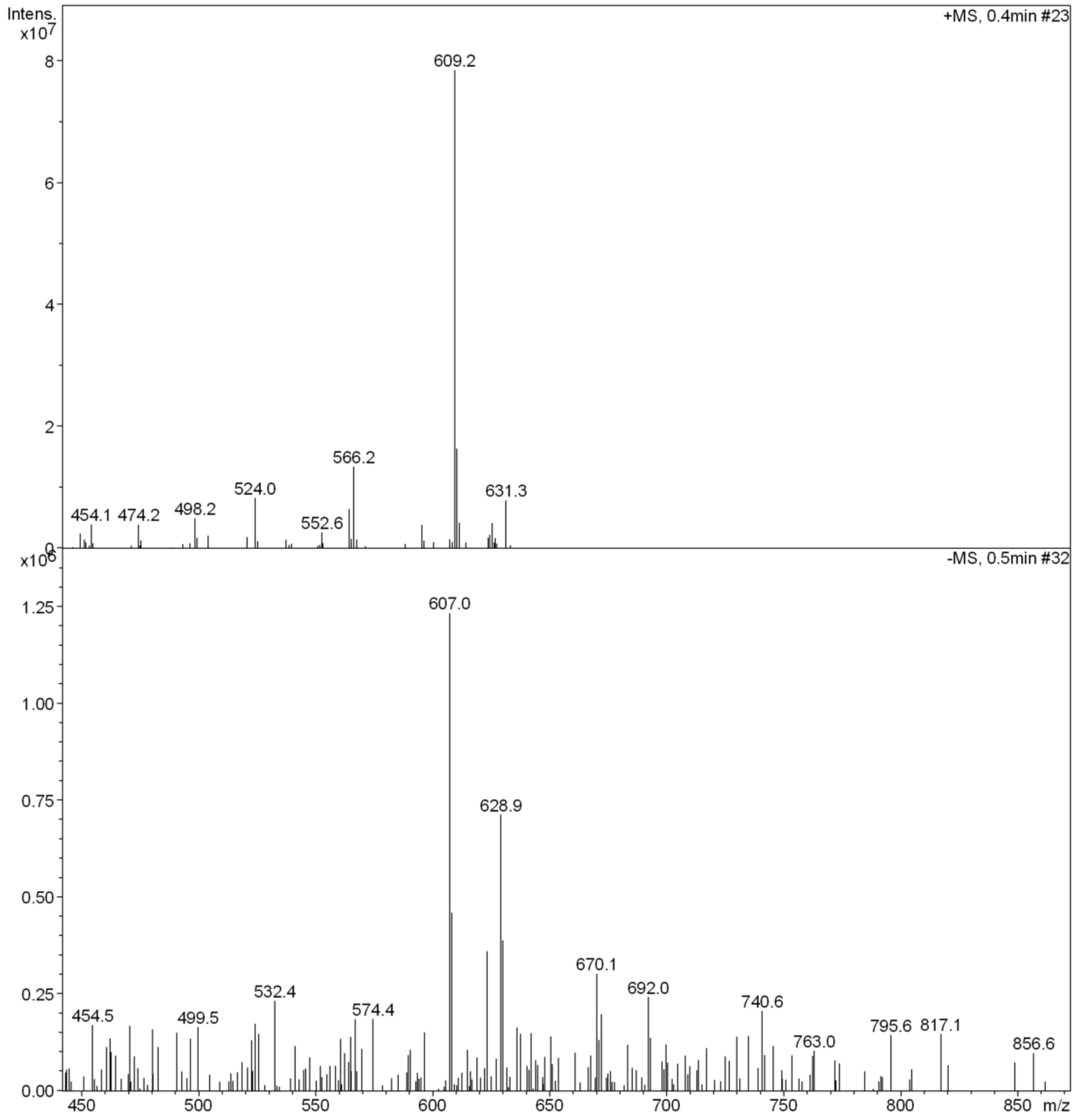

ESI-MS Spectrum of UB06

Print Window : MS Spectrum
Data : E:\DATA\DEF_LC1 2021-05-14 21-33-57\019-0901.D
Sample Name : SN-4

Operator	:	Sequence Line	: 9
Instrument	: Instrument 1	Position	: 19
Injection Date	: 2021/5/14 22:35:15	Injection Count	: 1
		Injection Volume	: 5.000 µl

Acquisition Method : E:\DATA\DEF_LC1 2021-05-14 21-33-57\100TO1000P_AND_N.M
Last Modified : 2021/4/17 14:18:09
Analysis Method : C:\CHEM32\1\METHODS\100TO1000P_AND_N.M
Last Modified : 2021/5/15 8:53:29

Method : 2080F

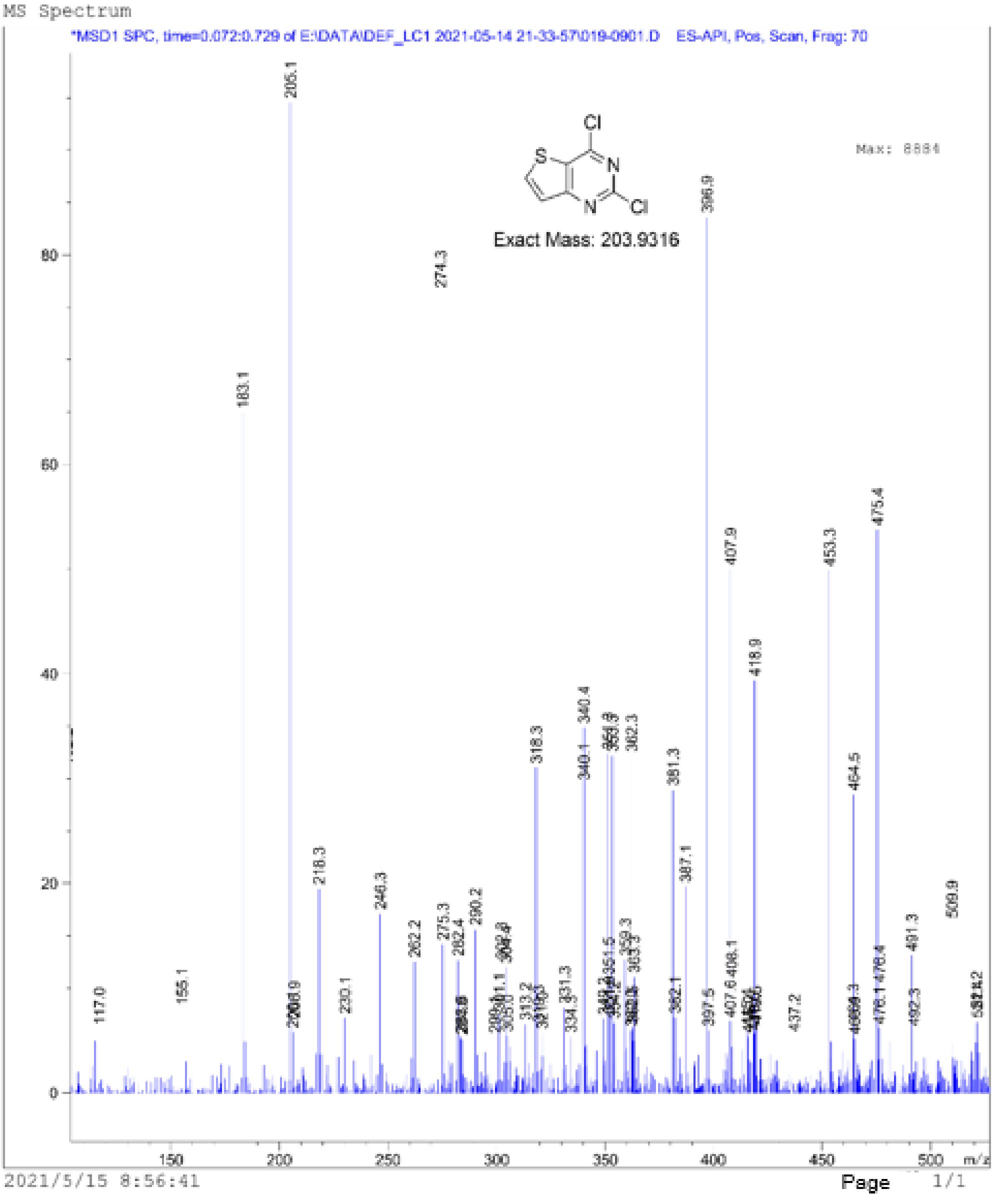

2021/5/15 8:56:41 Page 1/1

ESI-MS Spectrum of 20

Print Window : MS Spectrum

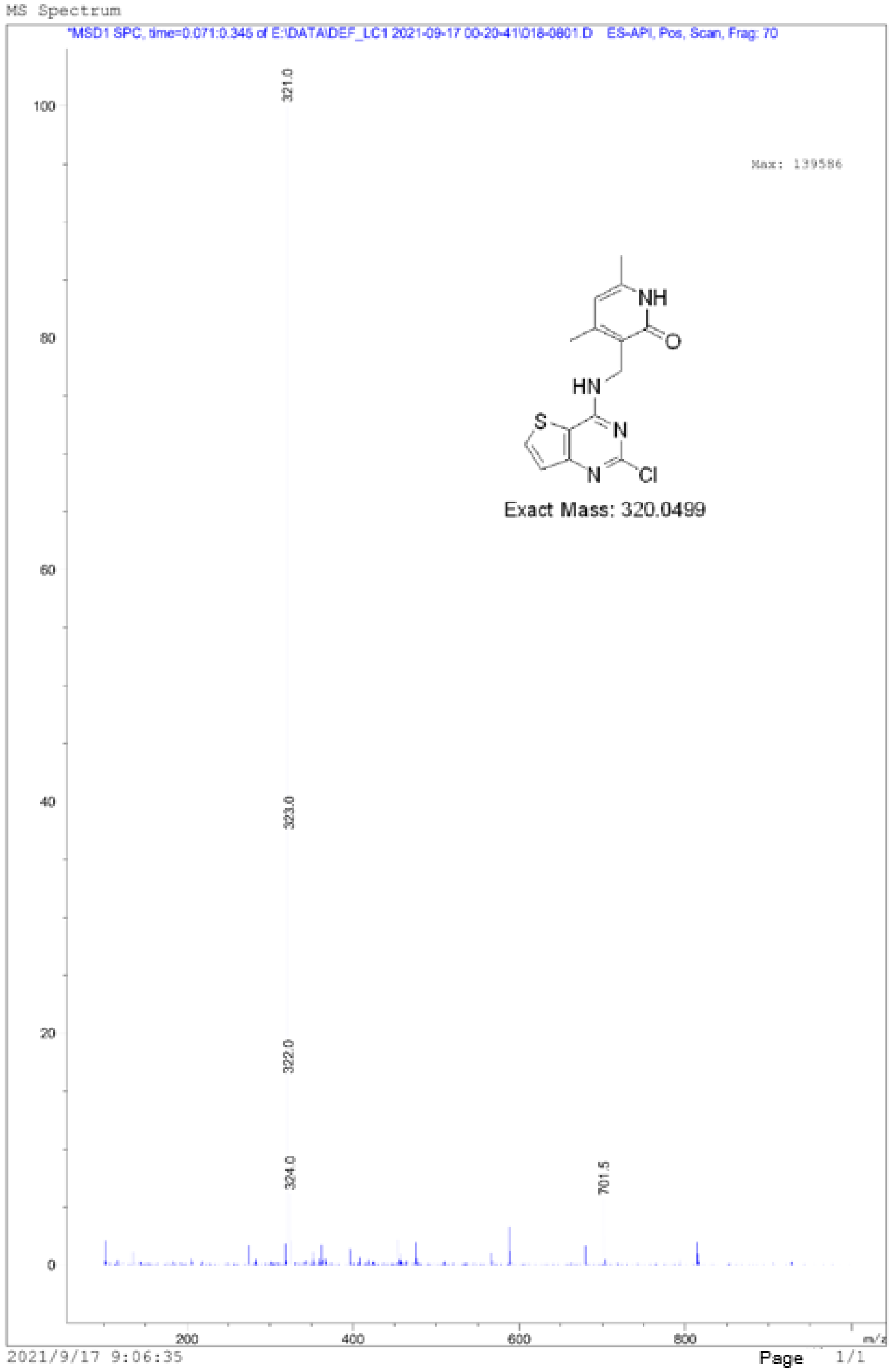

ESI-MS Spectrum of 21

Direct Mass Spectrometry Analysis

Analysis Name: 23032470.d
Sample Name: N1
Instrument: LC-MSD-Trap-SL
Operator: 21953830E000H12U
Print Date: 3/24/2023 7:42:10 PM
Acq. Date: 3/24/2023 7:40:37 PM

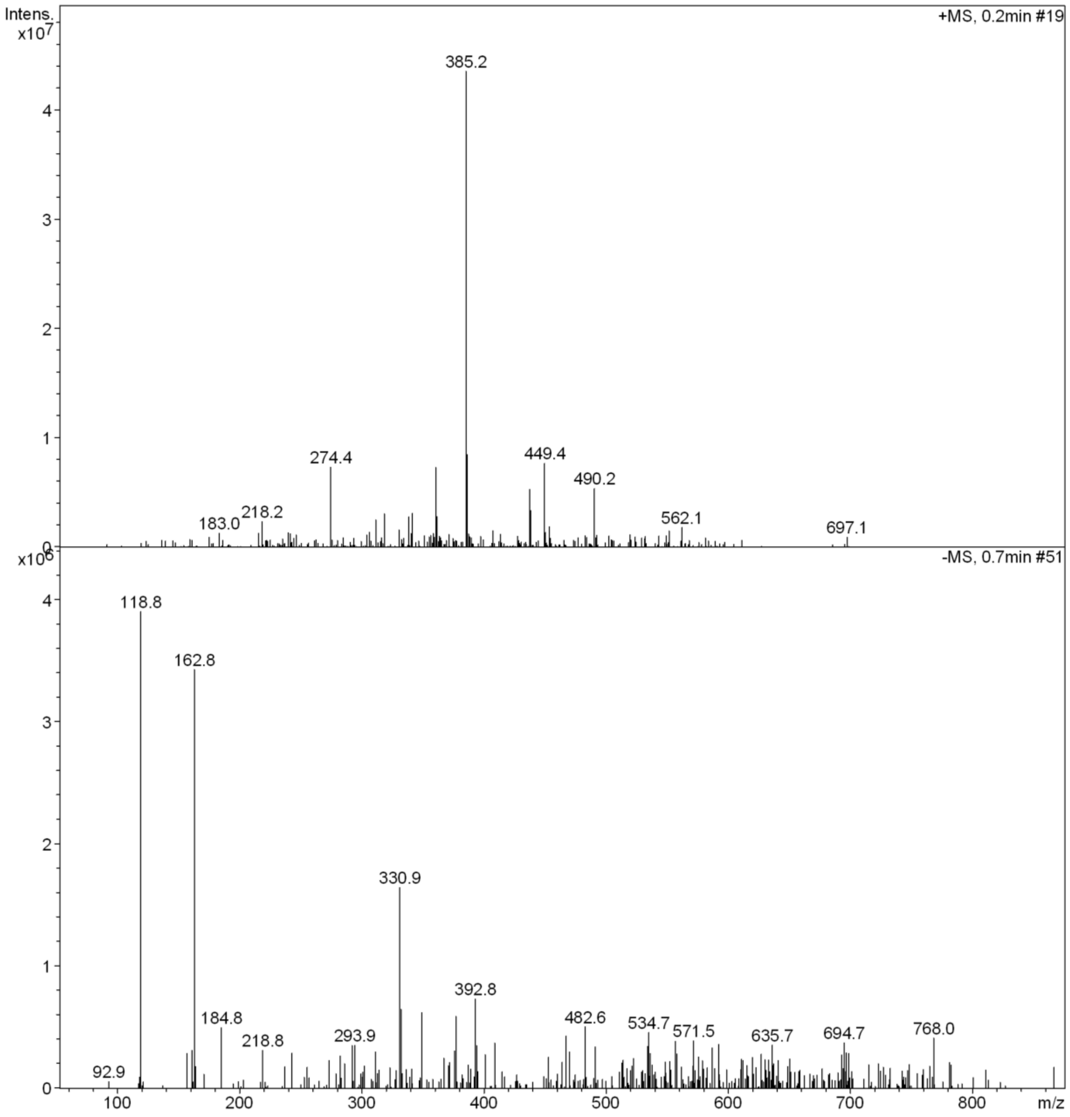

ESI-MS Spectrum of N1

Direct Mass Spectrometry Analysis

Analysis Name: 23032473.d
Sample Name: N2
Instrument: LC-MSD-Trap-SL
Operator: 21953830E000H12U
Print Date: 3/24/2023 7:50:03 PM
Acq. Date: 3/24/2023 7:48:35 PM

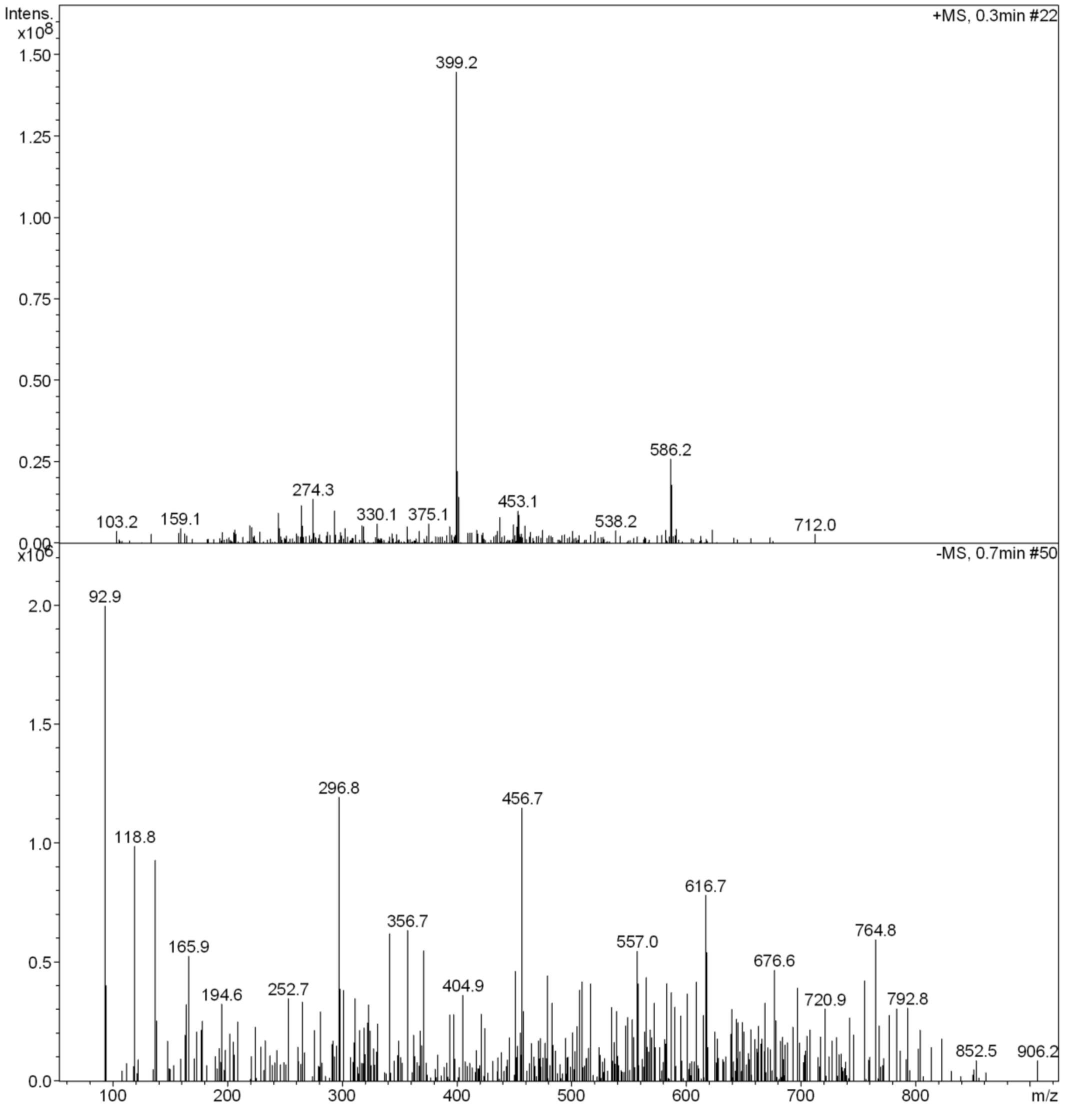

ESI-MS Spectrum of N2

Direct Mass Spectrometry Analysis

Analysis Name: 23032475.d
Sample Name: N3
Instrument: LC-MSD-Trap-SL
Operator: 21953830E000H12U
Print Date: 3/24/2023 7:55:43 PM
Acq. Date: 3/24/2023 7:53:45 PM

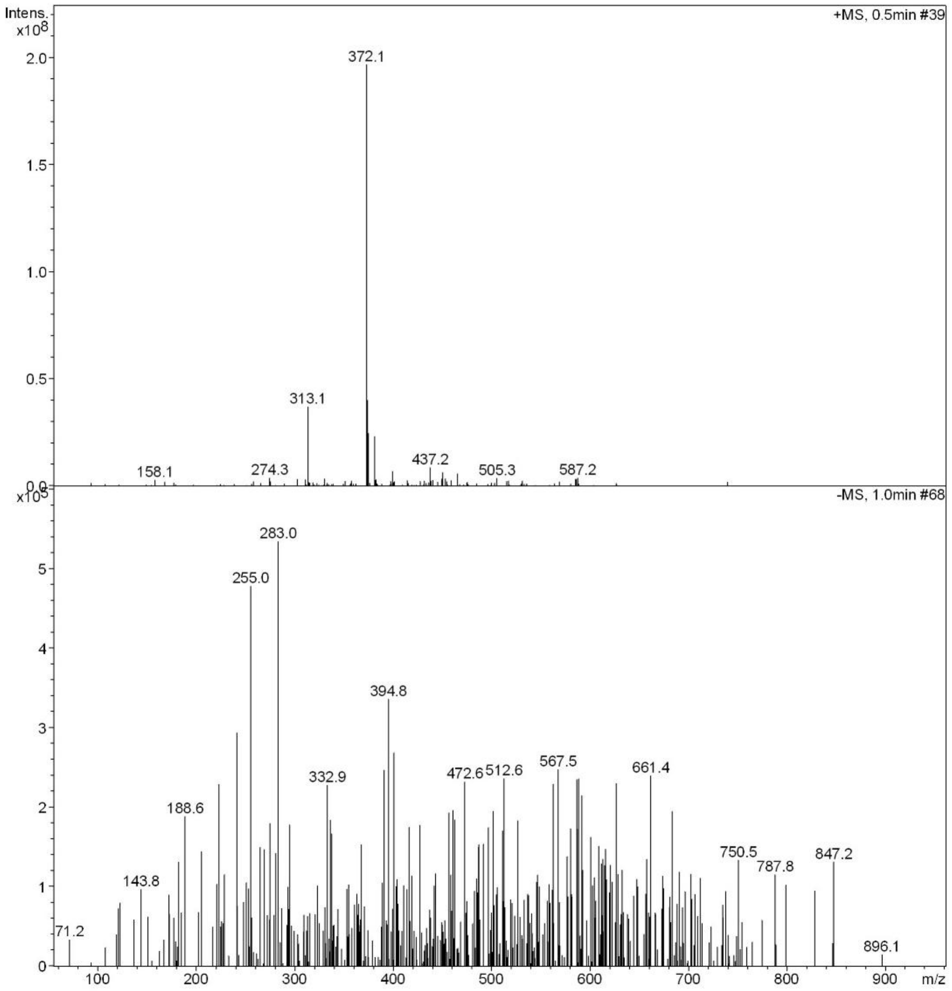

ESI-MS Spectrum of N3

Direct Mass Spectrometry Analysis

Analysis Name: 23032476.d
Sample Name: N4
Instrument: LC-MSD-Trap-SL
Operator: 21953830E000H12U
Print Date: 3/24/2023 7:58:06 PM
Acq. Date: 3/24/2023 7:56:39 PM

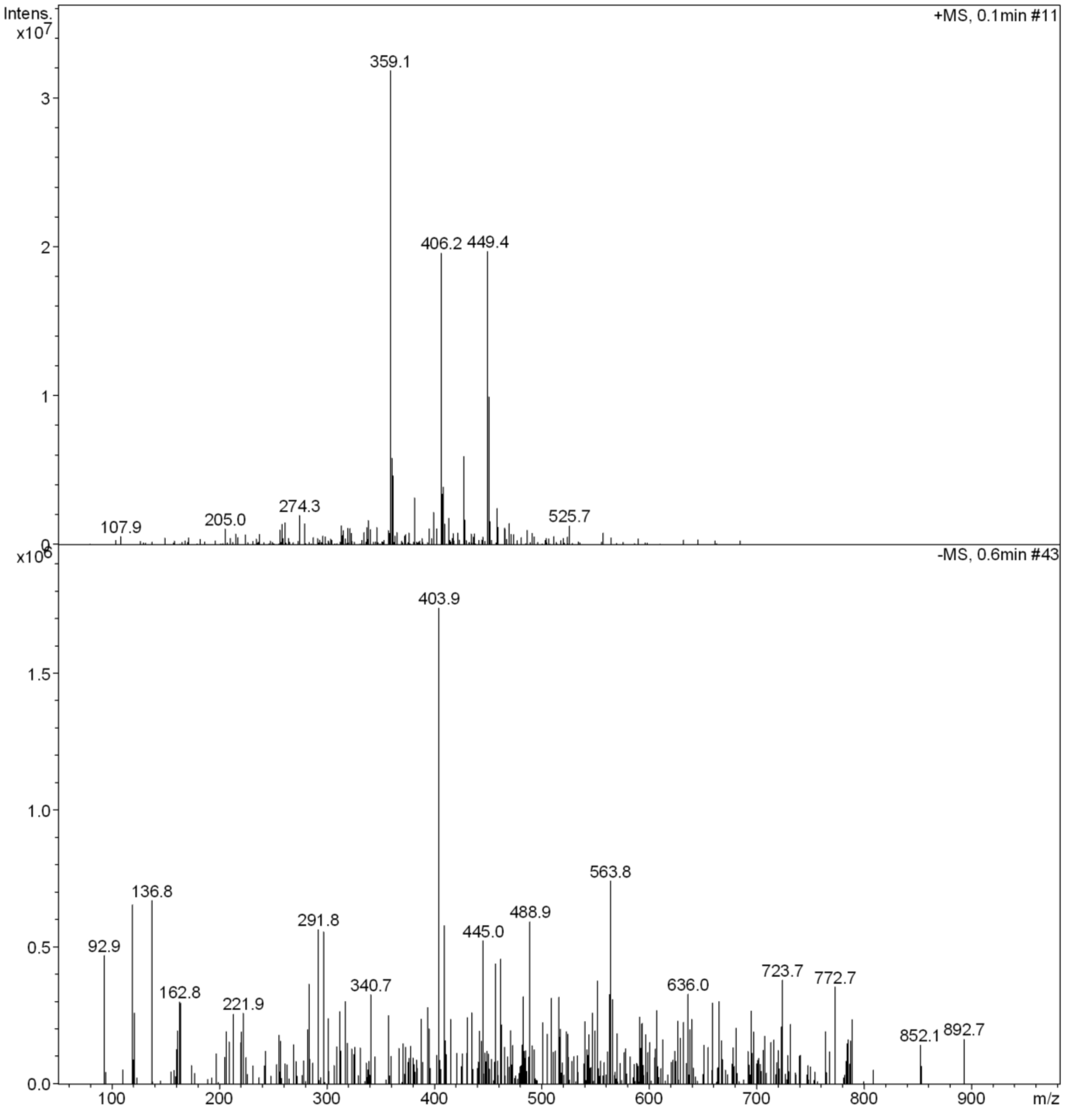

ESI-MS Spectrum of N4

Direct Mass Spectrometry Analysis

Analysis Name: 23032480.d
Sample Name: N5

Instrument: LC-MSD-Trap-SL
Operator: 21953830E000H12U

Print Date: 3/24/2023 8:09:45 PM
Acq. Date: 3/24/2023 8:08:02 PM

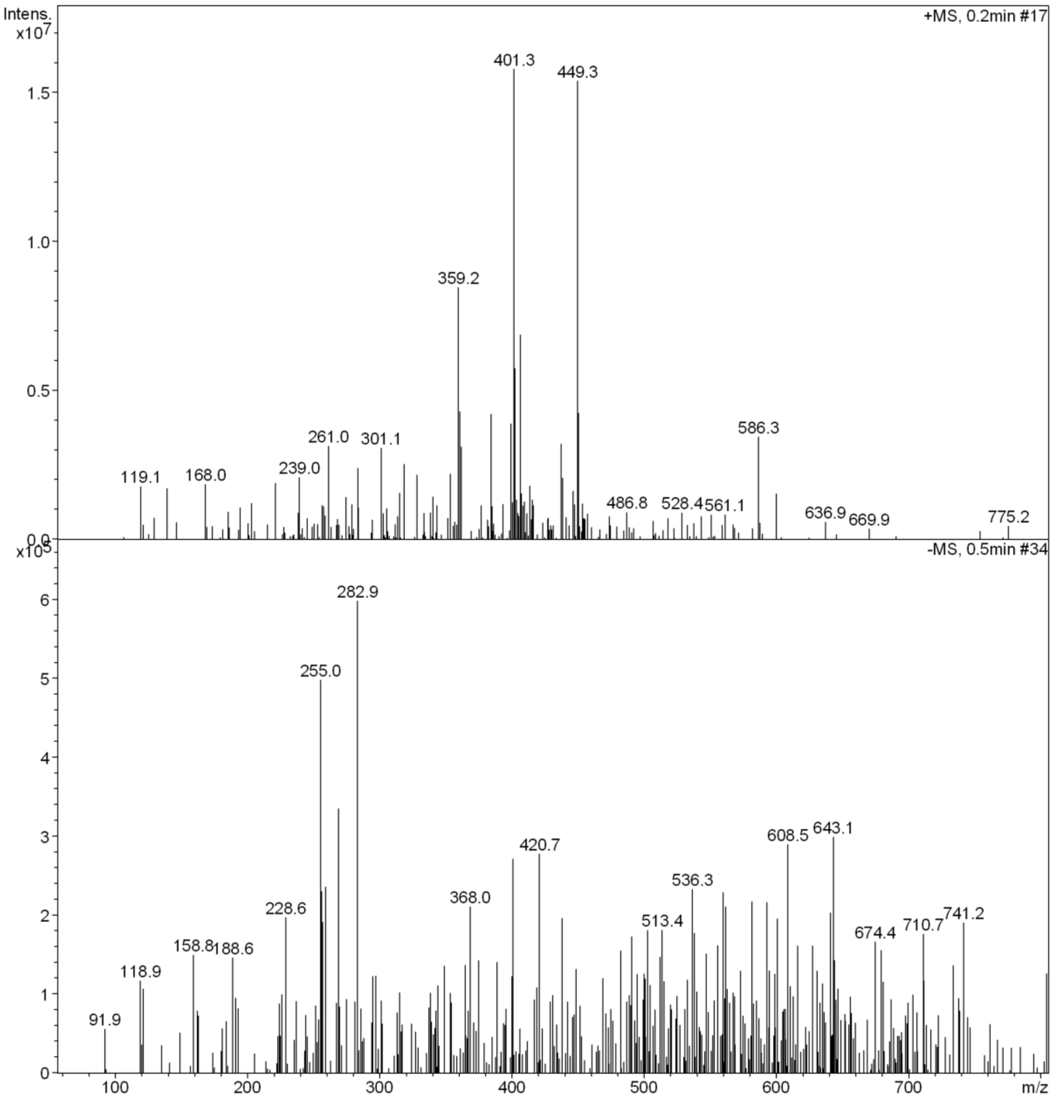

ESI-MS Spectrum of N5

Direct Mass Spectrometry Analysis

Analysis Name: 23032477.d
Sample Name: N6

Instrument: LC-MSD-Trap-SL
Operator: 21953830E000H12U

Print Date: 3/24/2023 8:00:21 PM
Acq. Date: 3/24/2023 7:58:50 PM

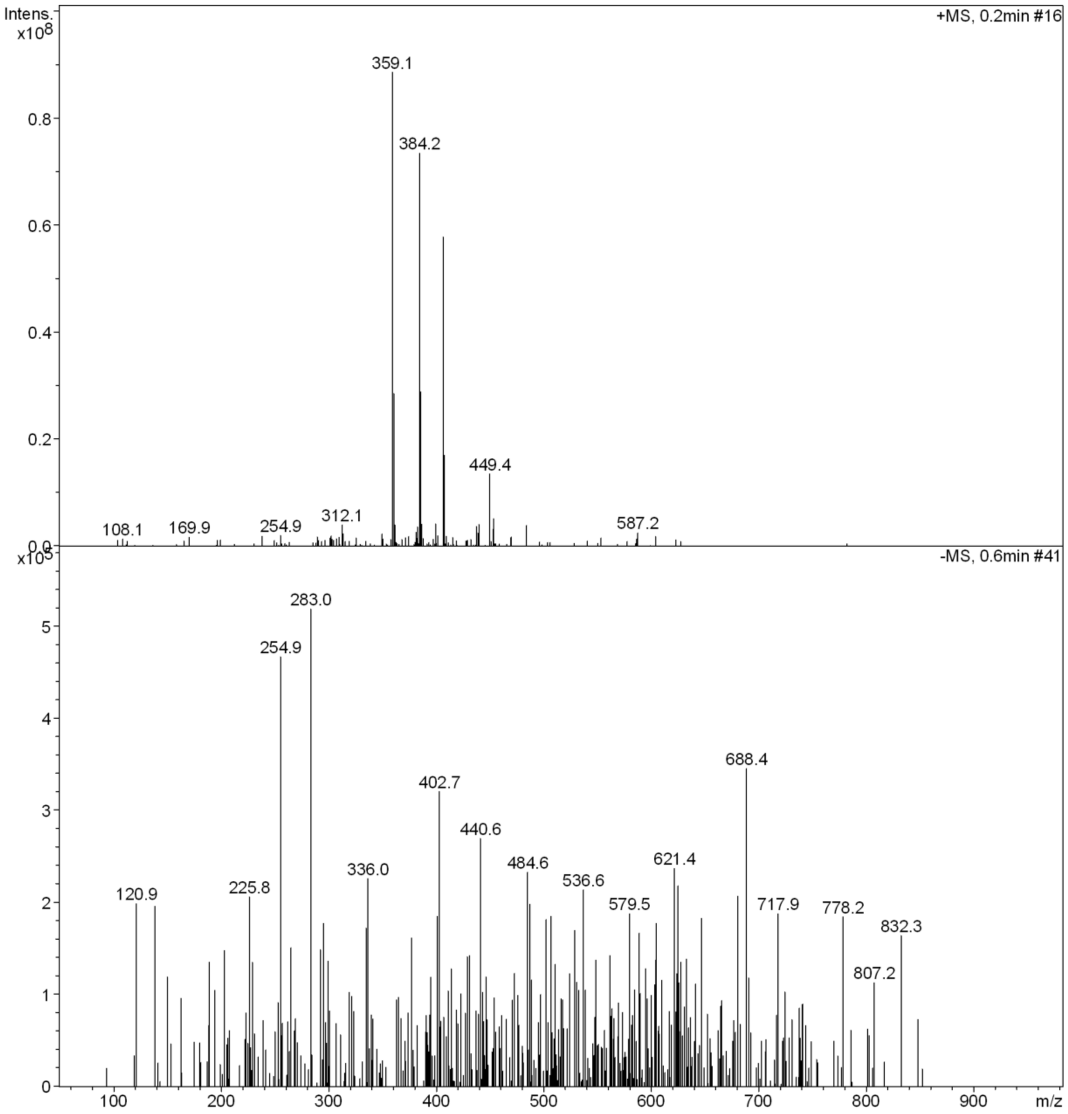

ESI-MS Spectrum of N6

Direct Mass Spectrometry Analysis

Analysis Name: 23032482.d
Sample Name: N7
Instrument: LC-MSD-Trap-SL
Operator: 21953830E000H12U
Print Date: 3/24/2023 8:14:18 PM
Acq. Date: 3/24/2023 8:13:06 PM

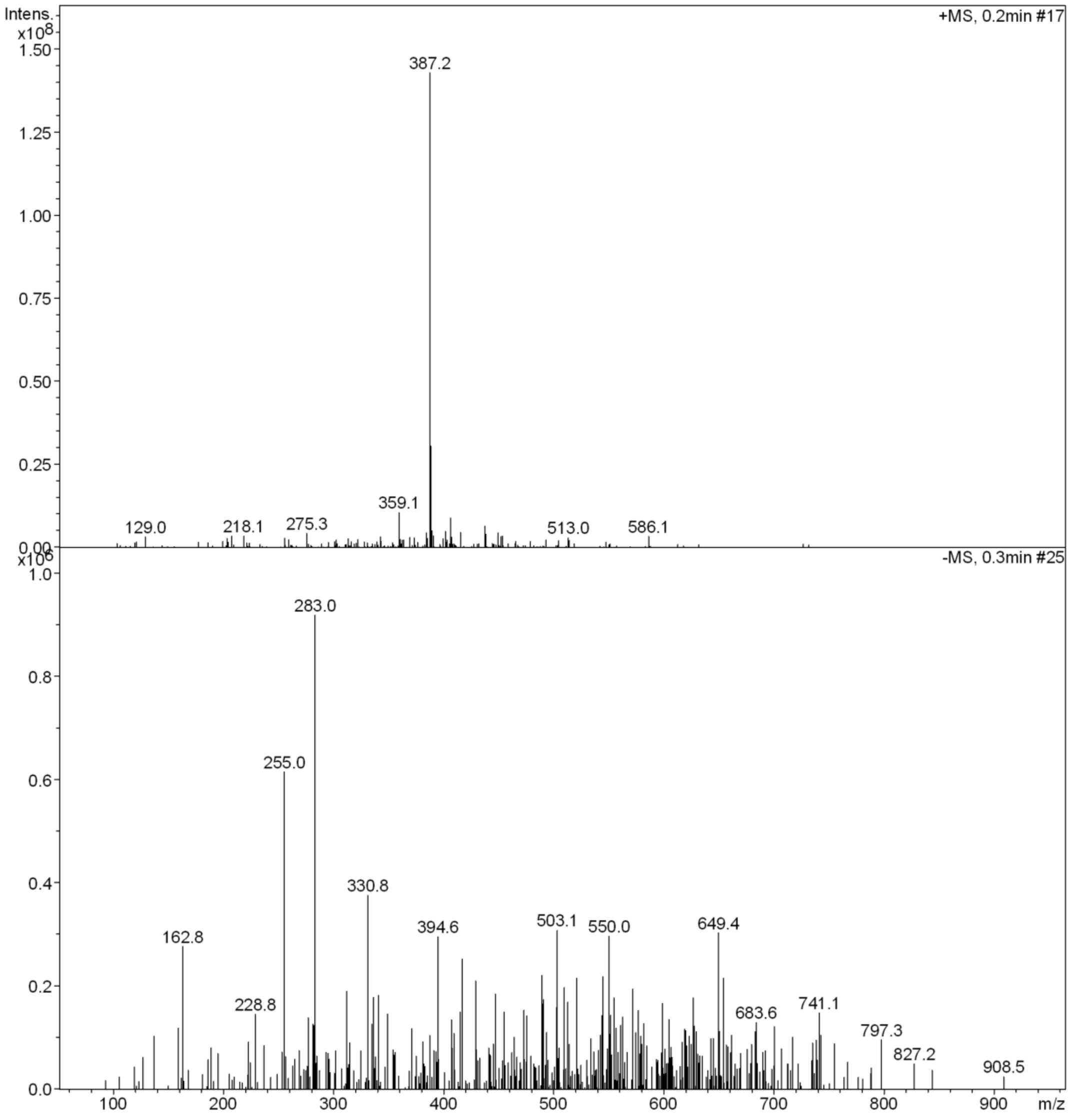

ESI-MS Spectrum of N7

Direct Mass Spectrometry Analysis

Analysis Name: 23032483.d **Instrument:** LC-MSD-Trap-SL **Print Date:** 3/24/2023 8:16:45 PM
Sample Name: N8 **Operator:** 21953830E000H12U **Acq. Date:** 3/24/2023 8:14:43 PM

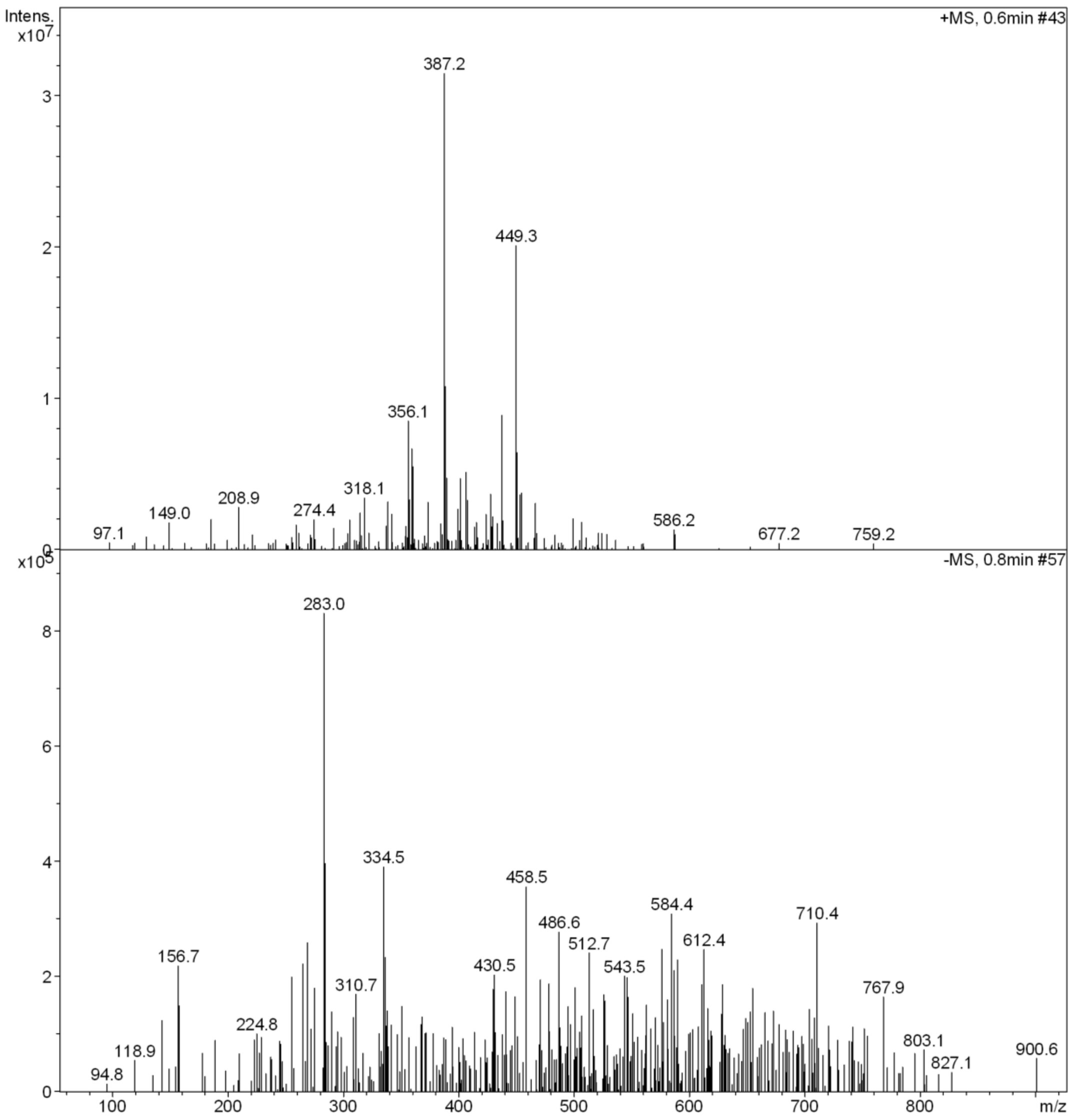

ESI-MS Spectrum of N8

Direct Mass Spectrometry Analysis

Analysis Name: 23032484.d
Sample Name: N9
Instrument: LC-MSD-Trap-SL
Operator: 21953830E000H12U
Print Date: 3/24/2023 8:18:29 PM
Acq. Date: 3/24/2023 8:17:12 PM

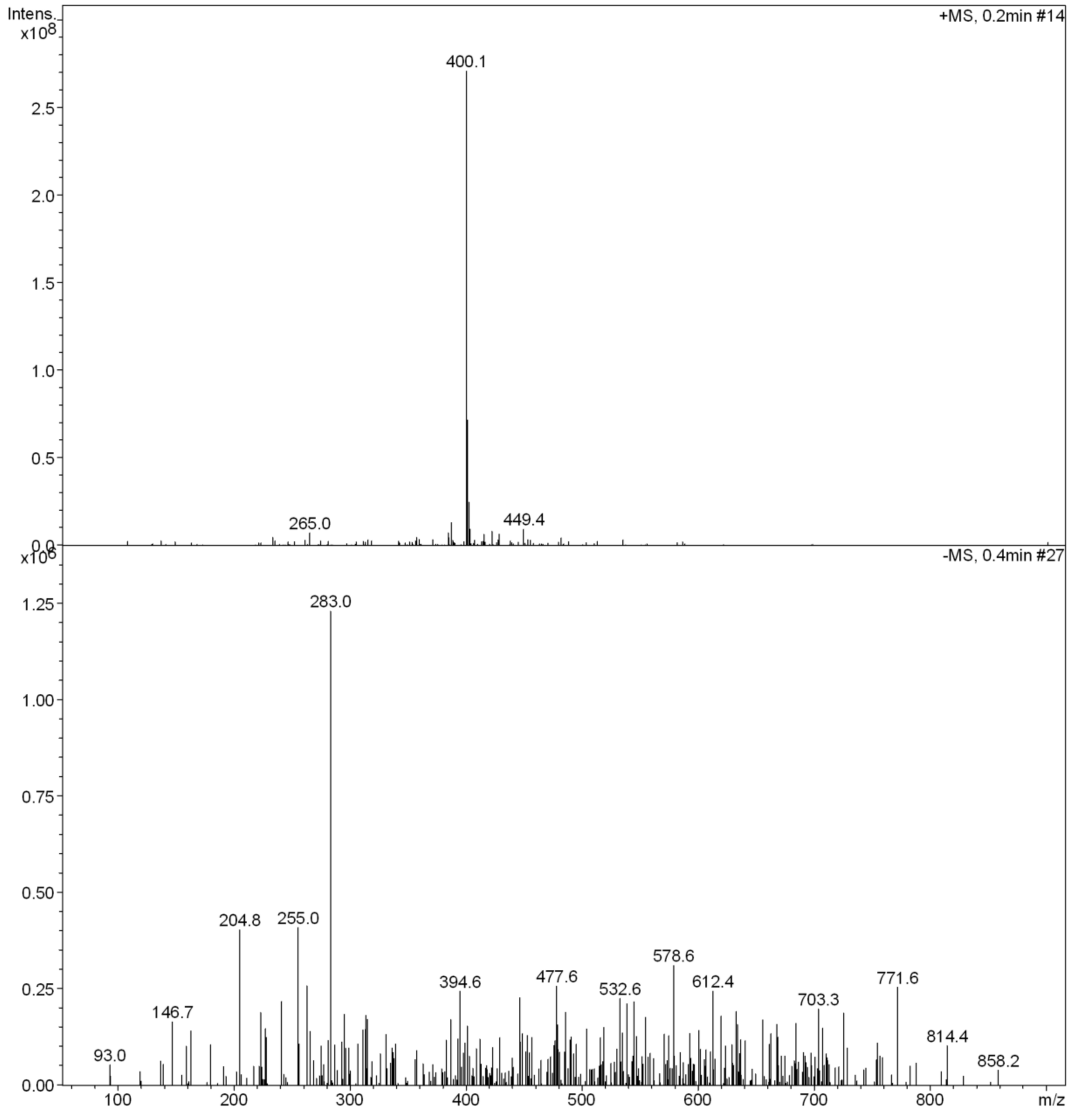

ESI-MS Spectrum of N9

Direct Mass Spectrometry Analysis

Analysis Name: 23032485.d **Instrument:** LC-MSD-Trap-SL **Print Date:** 3/24/2023 8:20:35 PM
Sample Name: N10 **Operator:** 21953830E000H12U **Acq. Date:** 3/24/2023 8:19:06 PM

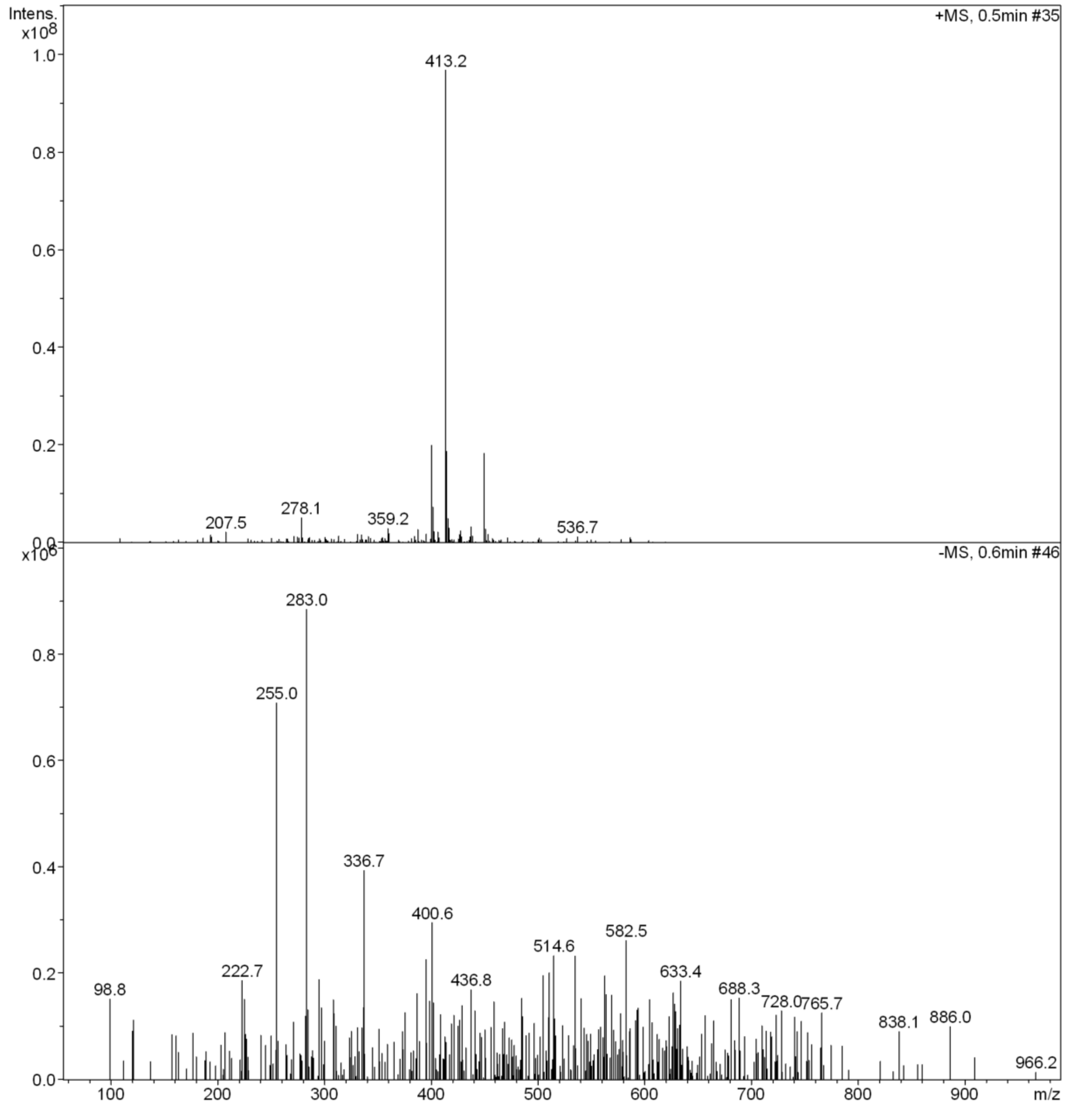

ESI-MS Spectrum of N10

Direct Mass Spectrometry Analysis

Analysis Name: 23032486.d
Sample Name: L1
Instrument: LC-MSD-Trap-SL
Operator: 21953830E000H12U
Print Date: 3/24/2023 8:22:30 PM
Acq. Date: 3/24/2023 8:21:02 PM

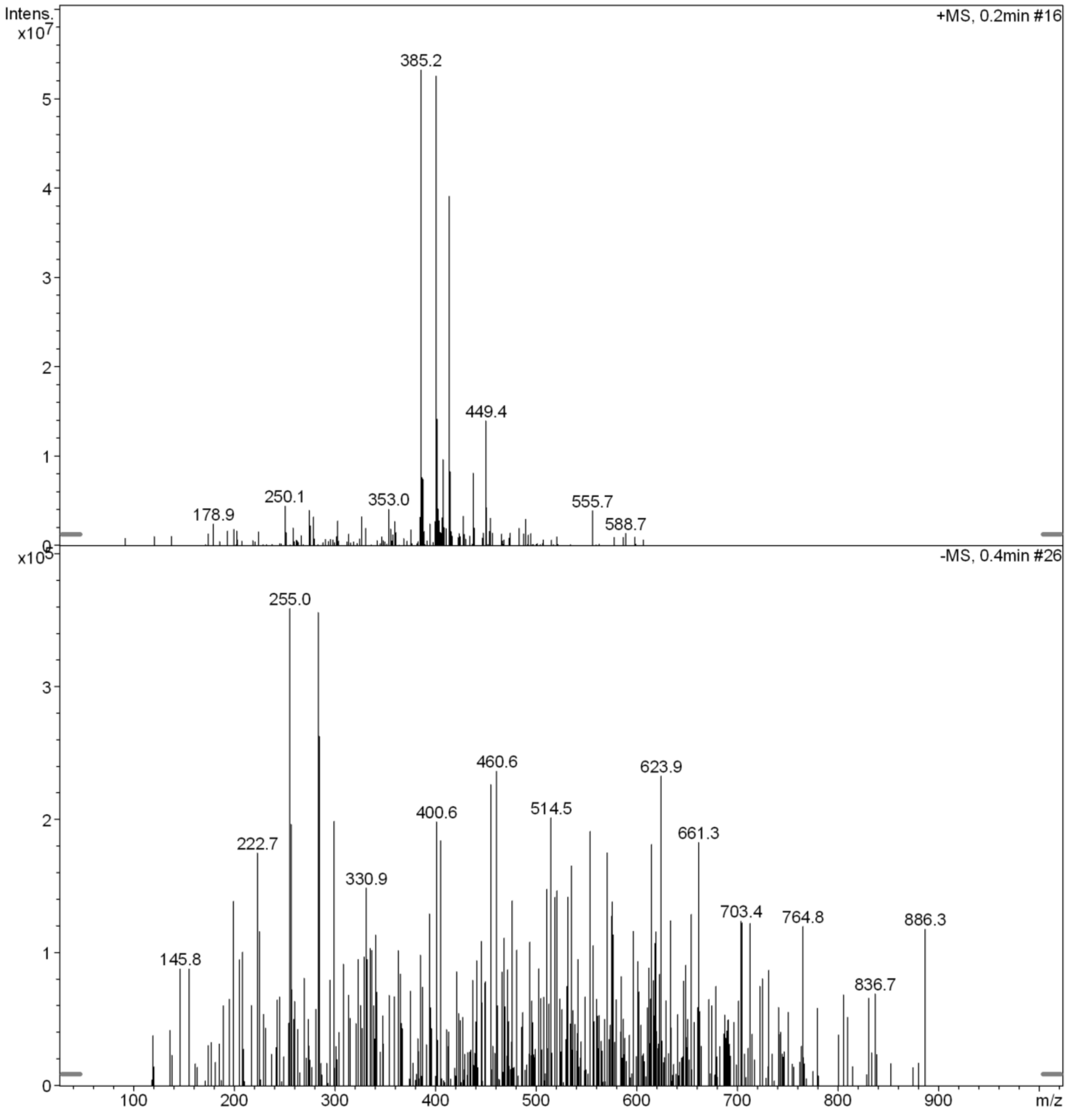

ESI-MS Spectrum of L1

Direct Mass Spectrometry Analysis

**Analysis Name:**	23032487.d	**Instrument:**	LC-MSD-Trap-SL	**Print Date:**	3/24/2023 8:24:20 PM
**Sample Name:**	L2	**Operator:**	21953830E000H12U	**Acq. Date:**	3/24/2023 8:22:53 PM

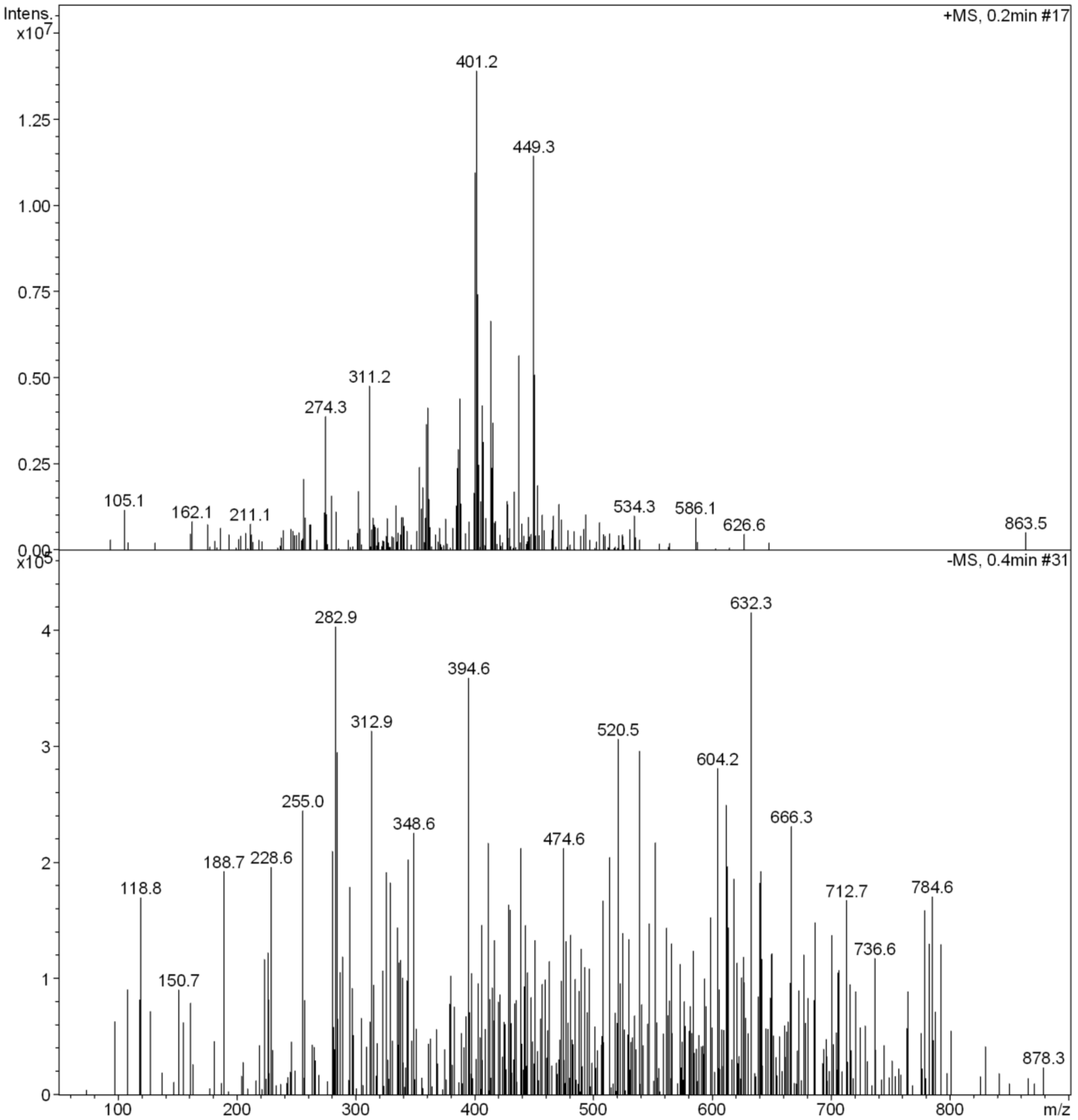

ESI-MS Spectrum of L2

Direct Mass Spectrometry Analysis

Analysis Name: 23032488.d
Sample Name: L3
Instrument: LC-MSD-Trap-SL
Operator: 21953830E000H12U
Print Date: 3/24/2023 8:26:08 PM
Acq. Date: 3/24/2023 8:24:49 PM

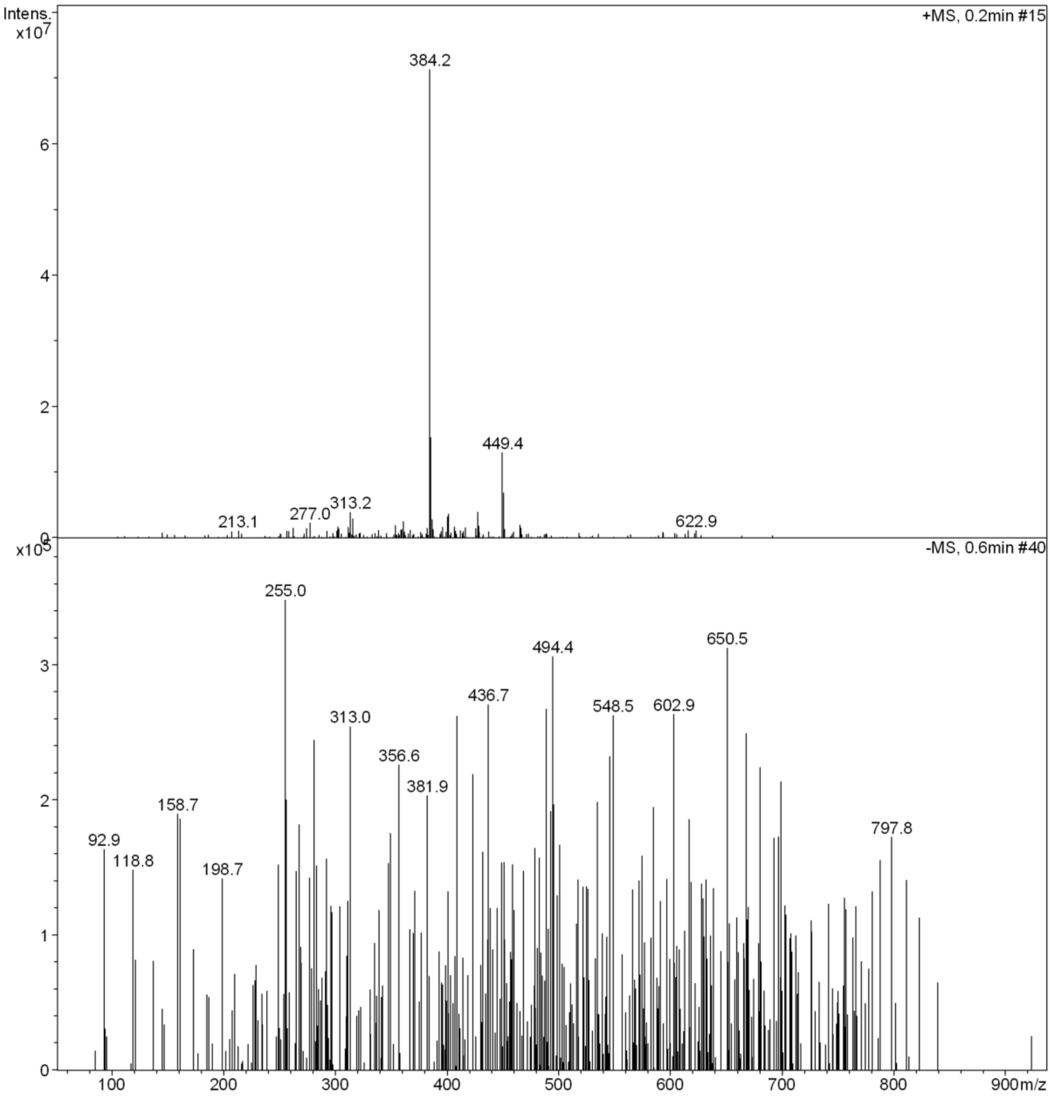

ESI-MS Spectrum of L3

Direct Mass Spectrometry Analysis

Analysis Name: 23032489.d **Instrument:** LC-MSD-Trap-SL **Print Date:** 3/24/2023 8:27:49 PM
Sample Name: L4 **Operator:** 21953830E000H12U **Acq. Date:** 3/24/2023 8:26:40 PM

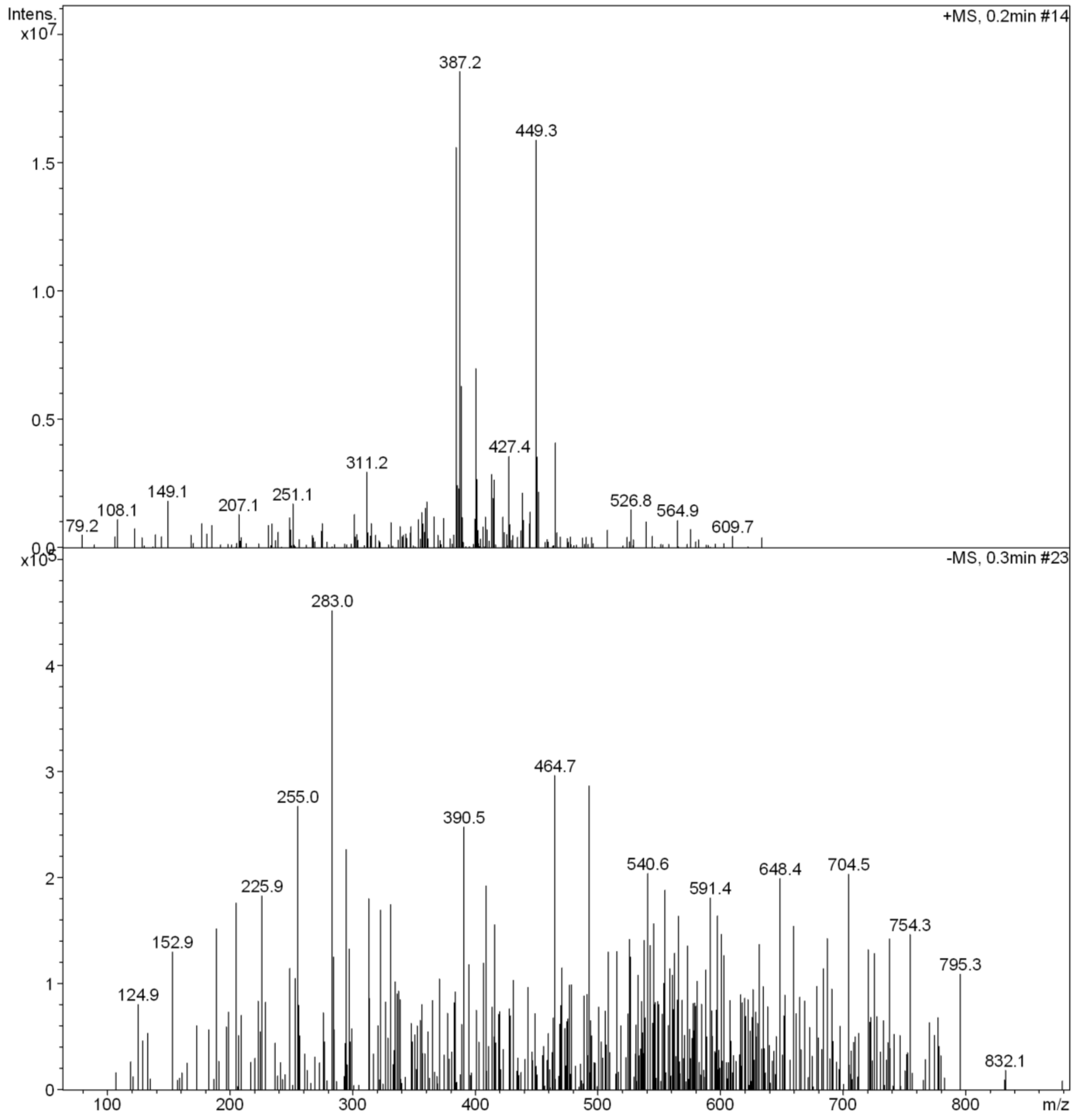

ESI-MS Spectrum of L4

Direct Mass Spectrometry Analysis

Analysis Name: 23032490.d
Sample Name: L5
Instrument: LC-MSD-Trap-SL
Operator: 21953830E000H12U
Print Date: 3/24/2023 8:29:14 PM
Acq. Date: 3/24/2023 8:28:15 PM

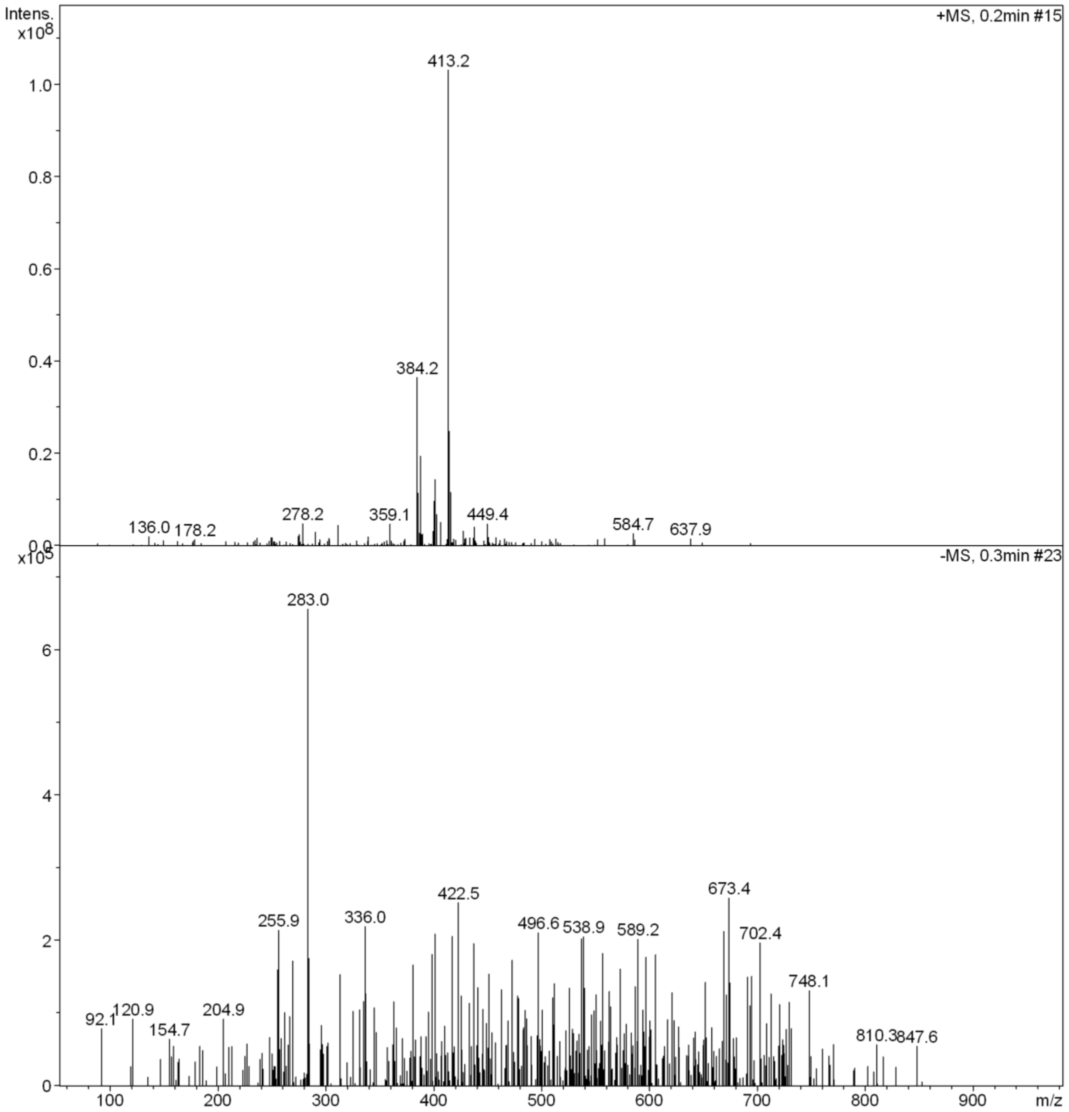

ESI-MS Spectrum of L5

References

1. *Encyclopedia of Reagents for Organic Synthesis;* John Wiley & Sons, Ltd: Chichester, UK, 2001, ISBN 0471936235.

2. Wang, P.-X.; Wang, Y.-N.; Lin, Z.-Y.; Li, G.; Huang, H.-H. Facile and efficient synthesis of quinazoline-2,4(1 H ,3 H)-diones through sequential hydrogenation condensation. *Synthetic Communications* **2018**, *48*, 1183–1189, doi:10.1080/00397911.2018.1439173.

3. Sundriyal, S.; Chen, P.B.; Lubin, A.S.; Lueg, G.A.; Li, F.; White, A.J.P.; Malmquist, N.A.; Vedadi, M.; Scherf, A.; Fuchter, M.J. Histone lysine methyltransferase structure activity relationships that allow for segregation of G9a inhibition and anti-Plasmodium activity. *Medchemcomm* **2017**, *8*, 1069–1092, doi:10.1039/c7md00052a.

4. Bhunia, S.; De, S.; Ma, D. Room Temperature Cu-Catalyzed N-Arylation of Oxazolidinones and Amides with (Hetero)Aryl Iodides. *Org. Lett.* **2022**, *24*, 1253–1257, doi:10.1021/acs.orglett.2c00122.

5. Gavade, S.N.; Balaskar, R.S.; Mane, M.S.; Pabrekar, P.N.; Shingare, M.S.; Mane, D.V. An efficient method for the N-arylation of phenylurea via copper catalyzed amidation. *Chinese Chemical Letters* **2011**, *22*, 675–678, doi:10.1016/j.cclet.2010.12.018.

6. Zhou, W.; Fan, M.; Yin, J.; Jiang, Y.; Ma, D. CuI/Oxalic Diamide Catalyzed Coupling Reaction of (Hetero)Aryl Chlorides and Amines. *J. Am. Chem. Soc.* **2015**, *137*, 11942–11945, doi:10.1021/jacs.5b08411.

7. Deng, J.; Peng, L.; Zhang, G.; Lan, X.; Li, C.; Chen, F.; Zhou, Y.; Lin, Z.; Chen, L.; Dai, R.; et al. The highly potent and selective dipeptidyl peptidase IV inhibitors bearing a thienopyrimidine scaffold effectively treat type 2 diabetes. *Eur. J. Med. Chem.* **2011**, *46*, 71–76, doi:10.1016/j.ejmech.2010.10.016.

8. Bedford, S.T.; Benwell, K.R.; Brooks, T.; Chen, I.; Comer, M.; Dugdale, S.; Haymes, T.; Jordan, A.M.; Kennett, G.A.; Knight, A.R.; et al. Discovery and optimization of potent and selective functional antagonists of the human adenosine A2B receptor. *Bioorg. Med. Chem. Lett.* **2009**, *19*, 5945–5949, doi:10.1016/j.bmcl.2009.08.040.
